# Synthesis of highly substituted allenylsilanes by alkylidenation of silylketenes

**DOI:** 10.1186/1860-5397-1-5

**Published:** 2005-08-26

**Authors:** Stephen P Marsden, Pascal C Ducept

**Affiliations:** 1 School of Chemistry, University of Leeds, Leeds LS2 9JT, UK; 2 Department of Chemistry, Imperial College London, London SW7 2AY, UK

## Abstract

**Background:**

Allenylsilanes are useful intermediates in organic synthesis. An attractive, convergent but little used approach for their synthesis is the alkylidenation of stable silylketenes. Reactions thus far have been limited to the use of unsubstituted silylketenes (or equivalents) with stabilised or semi-stabilised ylides only. The current study explores the reactions of substituted ketenes prepared through rhodium(II)-mediated rearrangement of silylated diazoketones.

**Results:**

A range of novel 1,3-disubstituted and 1,3,3-trisubstituted allenylsilanes were prepared using stabilised and semi-stabilised ylides. Alkylidenation with non-stabilised phosphorus ylides was not viable, but the use of titanium-based methylenating reagents was successful, allowing access to 1-substituted allenylsilanes.

**Conclusion:**

Many novel allenylsilanes may be accessed by alkylidenation of substituted silylketenes. Importantly, for the first time, simple methylenation of silylketenes has been achieved using titanium carbenoid-based reagents.

## Introduction

Allenylsilanes are versatile intermediates for organic synthesis.[1–2] They have two main modes of reactivity: firstly, as propargyl anion equivalents in thermal [3–4] or Lewis acid-mediated [5–6] addition to carbonyls, acetals and imines, and secondly as three-carbon partners in [3+2] annulation reactions. Thus, reaction with aldehydes,[7] imines/iminiums,[7–8] enones [9–11] and nitrosyl cations [12] leads to dihydrofurans, dihydropyrroles, cyclopentenes and isoxazoles respectively.[13] In most cases the silicon is retained in the final product and can be used as a handle for further synthetic elaboration.

**Figure 1:**
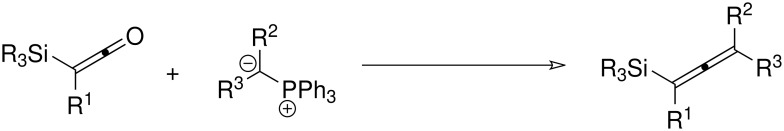
Alkylidenation approach to the synthesis of allenylsilanes.

Amongst the myriad methods to prepare allenylsilanes,[1,14] an attractive disconnection is to consider a Wittig-type alkylidenation of a silylketene, Figure 1.

This convergent approach potentially allows for tremendous variation in substitution pattern at both termini of the allenylsilane, yet has been little exploited thus far. Ruden first demonstrated that the stabilised phosphorane carbethoxymethylenetriphenylphosphorane underwent Wittig condensation with trimethylsilylketene at sub-ambient temperature in high yield, but found that non-stabilised phosphoranes led to complex mixtures of products.[15] Other workers later extended this chemistry to include a wider range of stabilised phosphoranes, but attempts to promote the reaction with semi-stabilised ylides such as benzylidenetriphenylphosphorane were unsuccessful unless bis(trialkylsilyl)ketenes were used as substrates.[16–17] Thus, only 3-substituted and 3,3-disubstituted allenylsilanes have thus far been accessed by alkylidenation of silylketenes, whilst no reports of the successful introduction of non-stabilised ylide equivalents have been forthcoming.

A second impediment to the generalisation of the approach has been the paucity of methods for preparing substituted silylketenes.[18–19] Methods based upon the thermolysis of siloxyalkynes [20] and dehydrohalogenation of substituted α-silyl acid halides [21–22] have not found general application. We recently reported a mild and functional group tolerant approach to substituted silylketenes based upon a rhodium-mediated formal Wolff rearrangement of silylated diazoketones.[23] Related photolytic approaches also hold some promise.[24–27] These methods allow access for the first time to a wide range of substituted silylketenes which will allow the chemistry of these fascinating persistent ketenes [18] to be better delineated.

In this paper we outline the synthesis of 1,3-disubstituted and 1,3,3-trisubstituted allenylsilanes by the condensation of stabilised and semi-stabilised ylides with substituted silylketenes, and report for the first time the methylenation of silylketenes to give 1-substituted allenylsilanes using titanium-based methodology.

## Results and Discussion

**Scheme 1:**
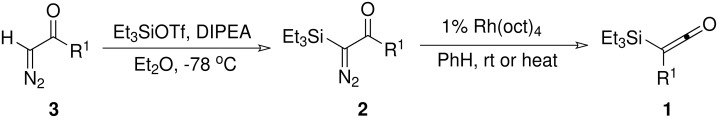
Synthesis of substituted silylketenes **1**

Our investigations began with the preparation of substituted silylketenes **1** as substrates for the alkylidenation chemistry. This was carried out under our previously reported conditions for rhodium(II) octanoate-mediated rearrangement of silyl diazoketones **2**,[23] which in turn were prepared by *C*-silylation of the parent diazoketones **3** with triethylsilyl triflate,[28] Scheme 1, Table 1. It should be noted that while the alkyl-substituted silylketenes are relatively stable and show little decomposition at room temperature over several days, the (hetero)aromatic-substituted silylketenes are much less robust and should be used quickly or stored in a freezer.

**Table 1:** Synthesis of substituted silylketenes 1

**Entry**	**R^1^**	**Yield (2)**	**Yield (1)**

a	CH_3_	82%	50%
b	C_7_H_15_	95%	65%
c	PhCH_2_	90%	72%
d	*c*C_6_H_11_	94%	77%
e	Ph	80%	43%
f	4-MeOPh	35%	24%
g	4-BrPh	90%	40%
h	2-furyl	74%	40%
i	2-thienyl	72%	27%
j	3-(*N*-Boc-indolyl)	46%	51%

**Scheme 2:**
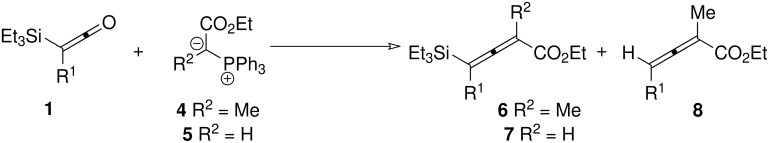
Reaction of substituted silylketenes with ester-stabilised phosphoranes

With the requisite silylketenes in hand, attention turned to their reaction with the carboethoxy-stabilised phosphoranes **4** and **5**. At the outset, it was by no means certain that these would react efficiently with substituted silylketenes **1** since it is well documented that nucleophiles attack silylketenes *anti* to the silicon, [29]*ie* the phosphoranes would be approaching from the same side as the R^1^-substituent. Since in all previous examples this substituent has been a hydrogen atom, the extension to bulkier substituents could not be taken for granted. In the event, however, we were pleased to find that in nearly all cases the desired allenylsilanes were formed in moderate to excellent yield, Scheme 2, Table 2.

**Table 2:** Reaction of substituted silylketenes with ester-stabilised phosphoranes

**Entry**	**Ketene**	**Ylide**	**Temp (°C)**	**t (h)**	**Solvent**	**Yield 6/7 (8)**

1	**1a**	**4**	80	24	PhH	54%
2	**1a**	**5**	rt	3	CH_2_Cl_2_	60%
3	**1b**	**4**	110	24	toluene	45%
4	**1b**	**5**	reflux	24	CH_2_Cl_2_	77%
5	**1c**	**4**	80	24	PhH	60%
6	**1c**	**5**	rt	6	CH_2_Cl_2_	81%
7	**1d**	**4**	110	48	toluene	22%^a^
8	**1d**	**5**	80	48	toluene	78%
9	**1e**	**4**	80	24	PhH	55% (7%)
10	**1f**	**4**	60	5	CH_2_Cl_2_	44% (3%)
11	**1h**	**4**	rt	6	CH_2_Cl_2_	0% (57%)
12	**1h**	**4**	50	1	CH_2_Cl_2_	7% (23%)
13	**1i**	**4**	rt	10	CH_2_Cl_2_	0% (67%)
14	**1i**	**5**	rt	2	CH_2_Cl_2_	98%
15	**1j**	**4**	80	12	PhH	74% (19%)

^a^ 60% of starting material recovered

As expected, reactions with the more substituted ylide **4** were significantly slower than those with the parent ylide **5** (compare reaction temperatures and times, entries 1, 3 and 5 versus entries 2, 4 and 6). Increasing the steric bulk of the ketene substituent also slows the reaction, in line with our expectations, exemplified by the reactions of cyclohexyl-substituted ketene **1d** (entries 7 and 8, compared with entries 1–6). In almost all cases, if reactions were left for extended periods beyond consumption of starting material, small quantities of desilylated allenes were observed. With alkyl-substituted ketenes this could be avoided by careful reaction monitoring, but was unavoidable in the (somewhat sluggish) reaction of ylie **4** with aromatic substituted ketenes **1e**/**f**, while in the case of furyl and thiophenyl-substituted ketenes **1h** and **1i**, these were the sole or predominant products of reaction. It appears that the presence of the (hetero)aryl group labilises the silicon to cleavage under the reaction conditions, likely reflecting the silyl group acting as an electrofuge with the (hetero)aryl substituent stabilising incipient anionic character at the adjacent carbon.

**Scheme 3:**
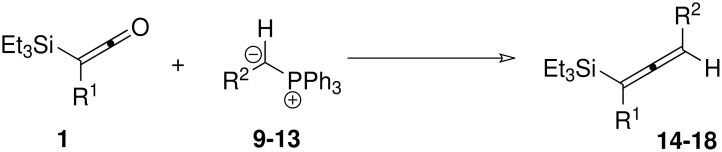
Reaction of silylketenes with various ylides

We next investigated the reaction of silylketenes **1** with other ylides (Scheme 3). Pleasingly, ylides stabilised by alternative electron-withdrawing groups such as cyano (**9**), acetyl (**10**) and benzoyl (**11**) were also successfully employed in allenylsilane formation. Likewise, the semi-stabilised aromatic-substituted ylides **12** and **13** also gave good yields of the allenylsilanes. The incorporation of the 4-bromophenyl group from **13** is particularly interesting since the bromide provides a handle for further functionalisation either of the allenylsilane or of the products of its subsequent reaction.

**Table 3:** Reaction of silylketenes with various ylides

**Entry**	**Ketene**	**Ylide**	**R^2^**	**Temp (°C)**	**t (h)**	**Solvent**	**Allene**	**Yield**

1	**1a**	**9**	CN	rt	1	CH_2_Cl_2_	**14a**	53%
2	**1b**	**9**	CN	rt	16	CH_2_Cl_2_	**14b**	94%
3	**1a**	**10**	COMe	rt	8	CH_2_Cl_2_	**15a**	52%
4	**1a**	**11**	COPh	50	5	CH_2_Cl_2_	**16a**	62%
5	**1b**	**11**	COPh	60	24	CH_2_Cl_2_	**16b**	62%
6	**1a**	**12**	Ph	rt	0.5	THF	**17a**	65%
7	**1b**	**12**	Ph	rt	0.5	THF	**17b**	68%
8	**1g**	**12**	Ph	rt	0.5	THF	**17g**	58%
9	**1b**	**13**	4-BrPh	rt	0.5	THF	**18b**	53%

^a^ the TBDMS ketene was employed in place of the TES ketene

Finally, the reactions of the non-stabilised ylides Ph_3_P=CHSPh, Ph_3_P=CHOMe and Ph_3_P=CH_2_ were examined. In all cases, rapid decomposition of the silylketenes to complex mixtures of unidentified products was observed, even at sub-ambient temperatures. One possible mechanism proposed for the decomposition of unsubstituted silylketenes with reactive ylides involves deprotonation by the basic reagent to generate ynolate anions. Clearly, since such a pathway cannot operate with the substituted ketenes used here other mechanisms are at play, which may include nucleophilic desilylation or ketene oligomerisation initiated by nucleophilic addition to the ketene carbonyl group. If the former pathway were occurring, then increasing the size of the silyl substituent ought to retard this pathway, but reaction of the *tert*-butyldimethylsilyl analogue of **1b** with Ph_3_P=CH_2_ also gave no observable allenylsilane product. The lack of identifiable products from these reactions precludes further speculation on the mechanism of decomposition at this stage.

**Scheme 4:**
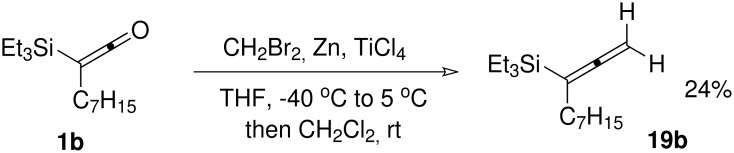
Methylenation of silylketene **1b** with the Lombardo reagent

The confirmation that non-stabilised ylides such as methylenetriphenylphosphorane were unsuitable reaction partners for condensation with even substituted silylketenes was disappointing, since the 1-substituted allenylsilanes **19** that would be thus formed are useful synthetic intermediates. Our attention therefore turned to the application of milder (and specifically less basic/nucleophilic) methylenating reagents. We first investigated the Lombardo reagent,[30] and were pleased to find that a modest yield of allenylsilane **19b** was obtained from this reaction (Scheme 4).

**Scheme 5:**
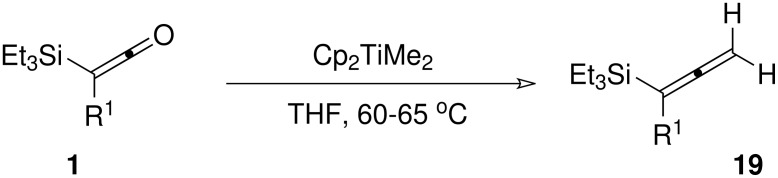
Methylenation of silylketenes with the Petasis reagent

Although the Lombardo reaction had several disadvantages (including the lengthy preparation of the reagent and the heterogeneous nature of the reaction medium) which led us to discount it as a long term solution to the problem, it gave us the encouragement to screen other titanium carbenoid-based methylenating reagents. The Petasis reagent (dimethyltitanocene) [31–32] was our next choice, and we were pleased to find that it afforded modest to excellent yields of the allenylsilanes in reaction with most of the ketene substrates examined (Scheme 5, Table 4).

**Table 4:** Methylenation of silylketenes with the Petasis reagent

**Entry**	**Ketene**	**Time (h)**	**Yield**

1	**1b**	6	78%
2	1b*	2	79%
3	**1c**	5	74%
4	**1d**	26	82%
5	**1e**	5	38%

^a^ the TBDMS ketene was employed in place of the TES ketene

The low yield with phenyl-substituted ketene **1e** is likely attributable to the instability of the ketene at the elevated temperatures for the Petasis reaction. Consistent with this, attempted reaction of the heteroaryl-substituted ketenes **1h**/**j** failed to return any allenylsilane product. We have not as yet examined the use of the more reactive Tebbe reagent which may function effectively at lower temperatures and hence offer a solution to this current limitation. Additionally, it will be of future interest to investigate the chromium-based Takai procedures for olefination with higher alkylidene reagents,[33] since this has the potential to deliver alkyl-substituted allenylsilanes.

In summary, we have further developed the reaction of stabilised and semi-stabilised ylides with silylketenes to include, for the first time, carbon-substituted silylketenes leading to a range of novel 1,3-disubstituted and 1,3,3-trisubstituted allenylsilanes. Additionally, we have demonstrated for the first time that titanium carbenoids can be used to methylenate silylketenes, providing the first access to 1-substituted allenylsilanes from these substrates. Given the broad range of useful chemistry undertaken by allenylsilanes, we expect that these new highly functionalised members of the class will find broad synthetic utility. Our own studies on their application in heterocyclic synthesis will be disclosed in due course.

## Description of Supporting Information

Supporting Information features copies of ^1^H nmr spectra of silylated diazoketones **2** and silylketenes **1**, plus ^1^H and ^13^C nmr spectra of allenylsilanes **6**, **7**, and **14**–**19**.

## Supporting Information

File 1 Experimental details

File 2 NMR spectra

File 3 NMR spectra
