# Supplementary material for: Synthesis of highly substituted allenylsilanes by alkylidenation of silylketenes
Source: Beilstein J Org Chem. 2005 Aug 26;1:5. doi: 10.1186/1860-5397-1-5 (PMC1399453; doi:10.1186/1860-5397-1-5)
Supplement: File 1 — Experimental details [file Beilstein_J_Org_Chem-01-05-s001.pdf]

Synthesis of Highly Substituted Allenylsilanes by Alkylidenation of Silylketenes

Stephen P. Marsden* and Pascal C. Ducept

Experimental section

This document contains full experimental protocols for the preparation of silyldiazoketones **2**, silylketenes **1** and all allenylsilanes **6**, **7**, and **14-19**.

Experimental

General procedure for the preparation of silyldiazoketones 2

1-Diazo-1-triethylsilylpropan-2-one 2a

Triethylsilyl trifluoromethanesulfonate (0.35 ml, 1.56 mmol) was added dropwise to a stirred solution of 1-diazopropan-2-one (0.094 g, 1.12 mmol) and dry DIPEA (0.39 ml, 2.24 mmol) in diethyl ether (5 ml) at 0°C. After 30 min, the reaction mixture was allowed to warm to rt, diluted with ether (10 ml) and washed successively with a saturated aqueous sodium hydrogen carbonate solution (2 ml), a saturated ammonium chloride solution (2 x 10 ml) and finally with brine (15 ml) and the combined organics dried ($MgSO_4$), filtered and evaporated under reduced pressure. The crude product was purified by flash chromatography (90:10 petroleum ether:diethyl ether) to yield 1-diazo-1-triethylsilylpropan-2-one **2a** as a yellow liquid (0.181 g, 82 %).

ν_{max} (KBr/film) 2961 s, 2878 s, 2066 s (C=N_2), 1732 s, 1643 s (C=O), 1464 m, 1359 m, 1270 s, 1203 s, 1018 s, 725 s cm^{-1}; δ_H (300 MHz, $CDCl_3$) 0.74 (6 H, q, J = 8 Hz, Si$\underline{CH_2}CH_3$), 0.95 (9 H, t, J = 8 Hz, $SiCH_2\underline{CH_3}$), 2.23 (3 H, s, CH_3); δ_C (75 MHz, $CDCl_3$) 2.7 Si$\underline{CH_2}CH_3$, 6.8 $SiCH_2\underline{CH_3}$, 26.4 CH_3, 53.3 C=N_2, 195.3 C=O; *m/z* (CI+, NH_3) 199 ($[M+H]^+$, 100), 102 (33), 85 ($[M-SiEt_3+2H]^+$, 35); HRMS (CI+) Found $[M+H]^+$ 199.1267 $C_9H_{19}N_2OSi$, requires 199.1267

1-Diazo-1-triethylsilylnonan-2-one 2b

Carried out by the general procedure above, with 1-diazononan-2-one (1.36 g, 8.08 mmol), triethylsilyl trifluoromethanesulfonate (2.2 ml, 9.7 mmol) and DIPEA (2.1 ml, 12.1 mmol) in diethyl ether (40 ml). The crude product was purified by flash chromatography (95:5 petroleum ether:diethyl ether) to yield 1-diazo-1-triethylsilylnonan-2-one **2b** as a yellow liquid (2.116 g, 93 %).

ν_{max} (KBr/film) 2956 s, 2064 s (C=N_2), 1642 s (C=O), 1463 w, 1181 w, 1007 m, 725 w cm^{-1}; δ_H (270 MHz, $CDCl_3$) 0.74 (6 H, q, J = 8 Hz, Si$\underline{CH_2}CH_3$), 0.85 (3 H, t, $J_{9\text{-}8}$ = 7 Hz, H_9), 0.96 (9 H, t, J = 8 Hz, $SiCH_2\underline{CH_3}$), 1.20-1.36 (8 H, m, $H_{5,6,7,8}$), 1.61 (2 H, p, J = 7.5 Hz, H_4), 2.48 (2 H,

t, $J_{3\text{-}4}$ = 7 Hz, H_3); δ_C (75 MHz, $CDCl_3$) 3.0 Si$\underline{CH_2}CH_3$, 7.1 $SiCH_2\underline{CH_3}$, 14.0 C_9, 22.6 C_8, 24.7, 29.0, 29.2 $C_{5,6,7}$, 31.7 C_4, 39.2 C_3, 55.0 C=N_2, 198.8 C=O; *m/z* (CI+, NH_3) 283 ($[M+H]^+$, 15), 255 ($[M-N_2+H]^+$, 100), 188 (30), 132 ($[SiEt_3+NH_3]^+$, 61); HRMS (CI+) Found $[M+H]^+$ 283.2252 $C_{15}H_{31}N_2OSi$, requires 283.2206

1-Diazo-3-phenyl-1-triethylsilylpropan-2-one 2c

Carried out by the general procedure above, with 1-diazo-3-phenylpropan-2-one (0.8 g, 5 mmol), triethylsilyl trifluoromethanesulfonate (1.35 ml, 6 mmol) and DIPEA (1.3 ml, 7.5 mmol) in diethyl ether (20 ml). The crude product was purified by flash chromatography (95:5 petroleum ether:diethyl ether) to yield 1-diazo-3-phenyl-1-triethylsilylpropan-2-one **2c** as a yellow liquid (2.116 g, 93 %).

ν_{max} (KBr/film) 2955 s, 2910 m, 2876 m, 2066 s (C=N_2), 1637 s (C=O), 1454 w, 1414 w, 1280 m, 1200 m, 1005 m, 725 s, 701 s cm^{-1}; δ_H (300 MHz, $CDCl_3$) 0.72 (6 H, q, *J* = 8 Hz, Si$\underline{CH_2}CH_3$), 0.91 (9 H, t, *J* = 8 Hz, $SiCH_2\underline{CH_3}$), 3.82 (2 H, s, H_3), 7.20-7.34 (5 H, m, Ph); δ_C (75 MHz, $CDCl_3$) 2.9 Si$\underline{CH_2}CH_3$, 7.0 $SiCH_2\underline{CH_3}$, 46.4 Ph$\underline{C}H_2$, 52.6 C=N_2, 126.9 $C_{4'}$, 128.6 $C_{2'}$ or $C_{3'}$, 129.1 $C_{3'}$ or $C_{2'}$, 134.4 $C_{1'}$, 195.6 C=O; *m/z* (CI+, NH_3) 275 ($[M+H]^+$, 92), 247 ($[M-N_2+H]^+$, 100), 161 ($[M-SiEt_3+H]^+$, 49), 132 ($[SiEt_3+NH_3]^+$, 55), 104 (40), 91 ($[PhCH_2]^+$, 43); HRMS (CI+) Found $[M+H]^+$ 275.1575 $C_{15}H_{23}N_2OSi$, requires 275.1579

1-Cyclohexyl-2-diazo-2-triethylsilylethan-1-one 2d

Carried out by the general procedure above with 1-cyclohexyl-2-diazoethan-1-one (1.05 g, 6.9 mmol), triethylsilyl trifluoromethanesulfonate (1.9 ml, 8.28 mmol) and DIPEA (1.8 ml, 10.35 mmol) in diethyl ether (30 ml). The crude product was purified by flash chromatography (95:5 petroleum ether:diethyl ether) to yield 1-cyclohexyl-2-diazo-2-triethylsilylethan-1-one **2d** as a yellow liquid (1.74 g, 94 %).

ν_{max} (KBr/film) 2933 s, 2875 m, 2856 m, 2061 s (C=N_2), 1641 s (C=O), 1452 w, 1251 m, 1214 m, 1187 m, 1172 m, 1006 w, 727 m, 700 w cm^{-1}; δ_H (300 MHz, $CDCl_3$) 0.71 (6 H, q, *J* = 8 Hz, Si$\underline{CH_2}CH_3$), 0.92 (9 H, t, *J* = 8 Hz, $SiCH_2\underline{CH_3}$), 1.13-1.48 (6 H, m, $H_{3',4'}$), 1.62-1.78 (4 H, m,

$H_{2'}$), 2.57 (1 H, tt, *J* = 11 Hz, $H_{1'}$); δ_C (75 MHz, $CDCl_3$) 2.9 Si<u>CH</u>$_2$CH$_3$, 7.0 SiCH$_2$<u>CH</u>$_3$, 25.6, 29.0 $C_{2',3'}$, 25.7 $C_{4'}$, 47.2 $C_{1'}$, 50.6 C=N_2, 202.0 C=O; *m/z* (CI+, NH_3) 267 ($[M+H]^+$, 100), 256 ($[M-N_2+NH_4]^+$, 20), 239 ($[M-N_2+H]^+$, 85), 132 ($[SiEt_3+NH_3]^+$, 31), 119 (24); HRMS (CI+) Found $[M+H]^+$ 267.1880 $C_{14}H_{27}N_2OSi$, requires 267.1892

2-Diazo-1-phenyl-2-triethylsilylethan-1-one 2e

Carried out by the general procedure above with 2-diazo-1-phenyl-ethan-1-one (0.67 g, 4.58 mmol), triethylsilyl trifluoromethanesulfonate (1.24 ml, 5.5 mmol) and DIPEA (1.2 ml, 6.86 mmol) in diethyl ether (20 ml). The crude product was purified by flash chromatography (95:5 petroleum ether:diethyl ether) to yield 2-diazo-1-phenyl-2-triethylsilyl-ethan-1-one **2e** as a yellow liquid (0.96 g, 80 %).

ν_{max} (KBr/film) 3064 w, 2955 m, 2910 w, 2876 m, 2067 s (C=N2), 1621 s (C=O), 1577 m, 1316 m, 1298 m, 1284 m, 1241 w, 1207 w, 1113 w, 1079 w, 1007 w cm^{-1}; δ_H (300 MHz, $CDCl_3$) 0.84 (6 H, q, *J* = 8 Hz, Si<u>CH</u>$_2$CH$_3$), 1.00 (9 H, t, *J* = 8 Hz, SiCH$_2$<u>CH</u>$_3$), 7.38-7.53 (3 H, m, $H_{3',4'}$), 7.57-7.60 (2 H, m, $H_{2'}$); δ_C (75 MHz, $CDCl_3$) 3.0 Si<u>CH</u>$_2$CH$_3$, 7.1 SiCH$_2$<u>CH</u>$_3$, 53.0 C=N_2, 127.0, 128.5 $C_{2',3'}$, 131.3 $C_{4'}$, 138.6 $C_{1'}$, 193.6 C=O; *m/z* (CI+, NH_3) 261 ($[M+H]^+$, 30), 250 (11), 234 ($[M-N_2+H]^+$, 58), 132 ($[SiEt_3+NH_3]^+$, 100); HRMS (CI+) Found $[M+H]^+$ 261.1421 $C_{14}H_{21}N_2OSi$, requires 261.1423

2-Diazo-1-(4-methoxyphenyl)-2-triethylsilylethan-1-one 2f

Carried out by the general procedure above with 2-diazo-1-(4-methoxyphenyl)-ethan-1-one (1.057 g, 6 mmol), triethylsilyl trifluoromethanesulfonate (2 ml, 9 mmol) and DIPEA (2.1 ml, 12 mmol) in diethyl ether (30 ml). The crude product was purified by flash chromatography (90:10 petroleum ether:diethyl ether) to yield 2-diazo-1-(4-methoxyphenyl)-2-triethylsilyl ethan-1-one **2f** as a yellow liquid (0.608 g, 35 %).

ν_{max} (KBr/film) 2956 s, 2910 m, 2875 m, 2065 s (C=N_2), 1616 s (C=O), 1514 m, 1313 m, 1290 s, 1172 m cm^{-1}; δ_H (270 MHz, $CDCl_3$) 0.80 (6 H, q, *J* = 8 Hz, Si<u>CH</u>$_2$CH$_3$), 0.99 (9 H, t, *J* = 8 Hz, SiCH$_2$<u>CH</u>$_3$), 3.83 (3 H, s, OCH_3), 6.90 (2 H, d, $J_{3'-2'}$ = 9 Hz, $H_{3'}$), 7.60 (2 H, d, $J_{2'-3'}$ = 9

Hz, $H_{2'}$); δ_C (75 MHz, $CDCl_3$) 3.0 Si<u>CH</u>$_2$CH$_3$, 7.1 SiCH$_2$<u>CH</u>$_3$, 51.1 C=N$_2$, 55.4 OCH$_3$, 113.7 $C_{3'}$, 129.2 $C_{2'}$, 131.3 $C_{1'}$, 162.1 $C_{4'}$, 192.3 C=O; *m/z* (CI+, NH_3) 291 ($[M+H]^+$, 20), 263 ($[M-N_2+H]^+$, 100), 252 (42), 235 (52), 132 ($[SiEt_3+NH_3]^+$, 59); HRMS (CI+) Found $[M+H]^+$ 291.1522 $C_{15}H_{23}N_2O_2Si$, requires 291.1529

2-Diazo-1-(4-bromophenyl)-2-triethylsilylethan-1-one 2g

Carried out by the general procedure above with 2-diazo-1-(4-bromophenyl)-ethan-1-one (0.675 g, 3 mmol), triethylsilyl trifluoromethanesulfonate (0.95 ml, 3.6 mmol) and DIPEA (0.78 ml, 4.5 mmol) in diethyl ether (10 ml). The crude product was purified by flash chromatography (95:5 petroleum ether:diethyl ether) to yield 2-diazo-1-(4-bromo phenyl)-2-triethylsilylethan-1-one **2g** as a yellow liquid (0.92 g, 90 %).

ν_{max} (KBr/film) 2954 s, 2910 m, 2875 s, 2069 s (C=N$_2$), 1614 s (C=O), 1587 m, 1303 s, 1292 s, 1205 m, 1105 m, 1070 m, 1010 s, 742 s cm^{-1}; δ_H (270 MHz, $CDCl_3$) 0.79 (6 H, q, *J* = 8 Hz, Si<u>CH$_2$</u>CH$_3$), 0.99 (9 H, t, *J* = 8 Hz, SiCH$_2$<u>CH$_3$</u>), 7.46 (2 H, d, $J_{3'-2'}$ = 8.5 Hz, $H_{3'}$), 7.55 (2 H, d, $H_{2'}$, $J_{2'-3'}$ = 8.5 Hz); δ_C (67.5 MHz, $CDCl_3$) 2.9 Si<u>CH</u>$_2$CH$_3$, 7.1 SiCH$_2$<u>CH</u>$_3$, 52.3 C=N$_2$, 125.8 $C_{4'}$, 128.6, 131.7 $C_{2',3'}$, 137.2 $C_{1'}$, 192.2 C=O; *m/z* (CI+, NH_3) 341 ($[M^{81}Br+H]^+$, 100), 339 ($[M^{79}Br+H]^+$, 98), 313 ($[M^{81}Br-N_2+H]^+$, 41), 311 ($[M^{79}Br-N_2+H]^+$, 42), 244 ($[M^{81}Br-SiEt_3+NH_4]^+$, 84), 242 ($[M^{79}Br-SiEt_3+NH_4]^+$, 89), 227 (68), 225 (67), 132 ($[SiEt_3+NH_3]^+$, 78), 106 (41), 89 (72); HRMS (CI+) Found $[M+H]^+$ 341.0503 $C_{14}H_{20}{}^{81}BrN_2OSi$, requires 341.0501

2-Diazo-1-(2-furyl)-2-triethylsilylethan-1-one 2h

Carried out by the general procedure above with 2-diazo-1-(2-furyl)-ethan-1-one (1.088 g, 8 mmol), triethylsilyl trifluoromethanesulfonate (2.7 ml, 12 mmol) and DIPEA (2.8 ml, 16 mmol) in diethyl ether (40 ml). The crude product was purified by flash chromatography (90:10 petroleum ether:diethyl ether) to yield 2-diazo-1-(2-furyl)-2-triethylsilylethan-1-one **2h** as a yellow liquid (1.486 g, 74 %).

ν_{max} (KBr/film) 3115 w, 2949 m, 2899 m, 2870 m, 2074 s (C=N$_2$), 1587 s (C=O), 1468 m, 1326 m, 1217 m, 1160 m cm^{-1}; δ_H (300 MHz, $CDCl_3$) 0.81 (6 H, q, *J* = 8 Hz, Si<u>CH$_2$</u>CH$_3$), 0.98

(9 H, t, *J* = 8 Hz, $SiCH_2\underline{CH_3}$), 6.50 (1 H, dd, $J_{4'-3'}$ = 3.5 Hz, $J_{4'-5'}$ = 1.5 Hz, $H_{4'}$), 7.07 (1 H, dd, $J_{3'-4'}$ = 3.5 Hz, $J_{3'-5'}$ = 1 Hz, $H_{3'}$), 7.47 (1 H, dd, $J_{5'-4'}$ = 1.5 Hz, $J_{5'-3'}$ = 1 Hz, $H_{5'}$); δ_C (75 MHz, $CDCl_3$) 3.0 $Si\underline{CH_2}CH_3$, 7.1 $SiCH_2\underline{CH_3}$, 51.1 C=N_2, 111.9, 114.8 $C_{3',4'}$, 143.9 $C_{5'}$, 153.3 $C_{2'}$, 178.6 C=O; *m/z* (CI+, NH_3) 251 ($[M+H]^+$, 73), 240 (42), 223 ($[M-N_2+H]^+$, 100), 195 (55), 132 ($[SiEt_3+NH_3]^+$, 54), 104 (22); HRMS (CI+) Found $[M+H]^+$ 251.1222 $C_{12}H_{19}N_2O_2Si$, requires 251.1216

2-Diazo-1-(2-thiophenyl)-2-triethylsilylethan-1-one 2i

The experiment was carried out by the general procedure for the preparation of silyl diazoketones with 2-diazo-1-(2-thiophenyl)-ethan-1-one (0.43 g, 2.85 mmol), triethylsilyl trifluoromethanesulfonate (0.77 ml, 3.42 mmol) and DIPEA (0.75 ml, 4.275 mmol) in diethyl ether (20 ml). The crude product was purified by flash chromatography (90:10 petroleum ether:diethyl ether) to yield 2-diazo-1-(2-thiophenyl)-2-triethylsilylethan-1-one **2i** as a yellow liquid (0.554 g, 73 %).

ν_{max} (KBr/film) 2956 m, 2909 w, 2876 w, 2064 s (C=N_2), 1594 s (C=O), 1416 s, 1302 s, 1240 w, 1203 w, 1005 w, 723 s cm^{-1}; δ_H (270 MHz, $CDCl_3$) 0.81 (6 H, q, *J* = 8 Hz, $Si\underline{CH_2}CH_3$), 0.99 (9 H, t, *J* = 8 Hz, $SiCH_2\underline{CH_3}$), 7.07 (1 H, dd, $J_{4'-3'}$ = 5 Hz, $J_{4'-5'}$ = 4 Hz, $H_{4'}$), 7.52 (1 H, dd, $J_{3'-4'}$ = 5 Hz, $J_{3'-5'}$ = 1 Hz, $H_{3'}$), 7.55 (1 H, dd, $J_{5'-4'}$ = 4 Hz, $J_{5'-3'}$ = 1 Hz, $H_{5'}$); δ_C (67.5 MHz, $CDCl_3$) 2.8 $Si\underline{CH_2}CH_3$, 7.1 $SiCH_2\underline{CH_3}$, 50.6 C=N_2, 127.5, 129.1 $C_{3',4'}$, 131.2 $C_{5'}$, 143.7 $C_{2'}$, 182.6 C=O; *m/z* (CI+, NH_3) 284 ($[M+NH_4]^+$, 10), 267 ($[M+H]^+$, 100), 256 (36), 239 ($[M-N_2+H]^+$, 80), 238 (48), 228 (34), 211 (42), 132 ($[SiEt_3+NH_3]^+$, 48); HRMS (CI+) Found $[M+H]^+$ 267.0989 $C_{12}H_{19}N_2OSSi$, requires 267.0987

1-(3-(1-*tert*-Butoxycarbonyl)indolyl)-2-diazo-2-triethylsilylethan-1-one 2j

Carried out by the general procedure above with 1-(3-(1-*tert*-butoxycarbonyl)indolyl)-2-diazo-1-ethanone (0.335 g, 1.235 mmol), triethylsilyl trifluoromethanesulfonate (0.34 ml, 1.48 mmol) and DIPEA (0.32 ml, 1.85 mmol) in diethyl ether (10 ml). The crude product was purified by flash chromatography (95:5 petroleum ether:diethyl ether) to yield 1-(3-(1-*tert*-

butoxycarbonyl)indolyl)-2-diazo-2-triethylsilylethan-1-one **2j** as a yellow liquid (0.229 g, 46 %).

ν_{max} (KBr/film) 2956 m, 2936 m, 2911 m, 2877 m, 2062 s (C=N_2), 1742 s (C=O Boc), 1608 m (C=O diazo), 1544 m, 1479 m, 1451 s, 1373 m, 1337 w, 1309 w, 1276 m, 1253 m, 1198 m, 1154 s, 1018 s cm^{-1}; δ_H (270 MHz, $CDCl_3$) 0.88 (6 H, q, *J* = 8 Hz, Si<u>CH</u>CH_3), 1.02 (9 H, t, *J* = 8 Hz, $SiCH_2\underline{CH_3}$), 1.67 (9 H, s, C($\underline{CH_3}$)), 7.27-7.38 (2 H, m, *Ind*H), 8.00-8.13 (2 H, m, *Ind*H), 8.00 (1 H, s, $H_{2'}$); δ_C (75 MHz, $CDCl_3$) 3.1 $Si\underline{CH_2}CH_3$, 7.2 $SiCH_2\underline{CH_3}$, 28.1 C($\underline{C}H_3$), 51.8 C=N_2, 85.0 $\underline{C}(CH_3)$, 114.9, 122.1, 123.9, 125.3, 128.1 $C_{2',4',5',6',7'}$, 119.7, 127.8, 134.9 $C_{3',3a',7a'}$, 149.0 C=O (Boc), 181.6 C=O; *m/z* (CI+, NH_3) 400 ($[M+H]^+$, 6), 389 ($[M-N_2+NH_4]^+$, 52), 371 ($[M-N_2]^+$, 70), 344 (56), 315 (76), 233 (100), 132 ($[SiEt_3+NH_3]^+$, 79), 119 (65); HRMS (CI+) Found $[M+H]^+$ 400.2077 $C_{21}H_{30}N_3O_3Si$, requires 400.2056

General procedure for the preparation of silyl ketenes 1

2-Triethylsilylpropen-2-one 1a

To a stirred solution of 1-diazo-1-triethylsilylpropan-2-one **2a** (1.09 g, 5.5 mmol) in dry benzene (5 ml) was added $Rh_2(octanoate)_4$ (0.043 g, 0.055 mmol). The resulting solution was stirred at rt for 15 min. Afterwards, the reaction mixture was concentrated under reduced pressure and the residual oil purified by flash chromatography (100 % petroleum ether) to yield 2-triethylsilylpropen-1-one **1a** as a colourless liquid (0.463 g, 50 %).

ν_{max} (KBr/film) 2955 s, 2911 m, 2877 s, 2088 s (C=O), 1790 w, 1725 w, 1458 w, 1239 w, 1093 w, 1018 w, 1004 w, 954 w, 738 m, 722 m, 710 m cm^{-1}; δ_H (300 MHz, $CDCl_3$) 0.61 (6 H, q, *J* = 8 Hz, $Si\underline{CH_2}CH_3$), 0.94 (9 H, t, *J* = 8 Hz, $SiCH_2\underline{CH_3}$) 1.54 (3 H, s, CH_3); δ_C (67.5 MHz, $CDCl_3$) 3.3 $Si\underline{CH_2}CH_3$, 7.1 $SiCH_2\underline{CH_3}$, 11.0 C_2, 53.4 C_3, 181.9 C_1; *m/z* (CI+, NH_3) 188 ($[M+NH_4]^+$, 13), 171 ($[M+H]^+$, 100), 132 ($[SiEt_3+NH_3]^+$, 49); HRMS (CI+) Found $[M+H]^+$ 171.1205 $C_9H_{19}OSi$, requires 171.1205

2-Triethylsilylnon-1-en-1-one 1b

Carried out by the general procedure above with 1-diazo-1-triethylsilylnonan-2-one **2b** (7.35 g, 26 mmol) and Rh_2(octanoate)$_4$ (0.2 g, 0.26 mmol) in benzene (50 ml). The crude product was purified by flash chromatography (petroleum ether) to yield 2-triethylsilylnon-1-en-1-one **1b** as a colourless liquid (4.97 g, 75 %).

ν_{max} (KBr/film) 2956 s, 2928 s, 2876 m, 2083 s (C=O), 1573 w, 1462 w, 1005 w, 720 w cm^{-1}; δ_H (300 MHz, $CDCl_3$) 0.60 (6 H, q, *J* = 8 Hz, Si$\underline{CH_2}CH_3$), 0.85 (3 H, t, $J_{9\text{-}8}$ = 7 Hz, H_9), 0.94 (9 H, t, *J* = 8 Hz, $SiCH_2\underline{CH_3}$), 1.20-1.32 (8 H, m, $H_{5,6,7,8}$), 1.46 (2 H, p, *J* = 7.5 Hz, H_4), 1.85 (2 H, t, $J_{3\text{-}4}$ = 7 Hz, H_3); δ_C (75 MHz, $CDCl_3$) 3.6 Si$\underline{CH_2}CH_3$, 7.1 $SiCH_2\underline{CH_3}$, 9.4 C_2, 14.0 C_9, 22.6 C_8, 29.0, 29.2 $C_{5,6,7}$, 31.7, 31.8 $C_{3,4}$, 181.9 C_1; *m/z* (CI+, NH_3) 255 ($[M+H]^+$, 100), 132 ($[SiEt_3+NH_3]^+$, 56); HRMS (CI+) Found $[M+H]^+$ 255.2146 $C_{15}H_{31}OSi$, requires 255.2144

3-Phenyl-2-triethylsilylpropen-1-one 1c

Carried out by the general procedure above with 1-diazo-3-phenyl-1-triethylsilylpropan-2-one **2c** (0.274 g, 1 mmol) and Rh_2(octanoate)$_4$ (0.007 g, 0.01 mmol) in benzene (5 ml). The crude product was purified by flash chromatography (petroleum ether) to yield 3-phenyl-2-triethylsilylpropen-1-one **1c** as a colourless liquid (0.158 g, 64 %).

ν_{max} (KBr/film) 3030 w, 2955 m, 2912 m, 2875 m, 2088 s (C=O), 1494 w, 1454 w, 1414 w, 1239 w, 1007 m, 735 m, 716 m, 700 m cm^{-1}; δ_H (270 MHz, $CDCl_3$) 0.65 (6 H, q, *J* = 8 Hz, Si$\underline{CH_2}CH_3$), 0.99 (9 H, t, *J* = 8 Hz, $SiCH_2\underline{CH_3}$), 3.25 (2 H, s, C_3), 7.20-7.39 (5 H, m, Ph); δ_C (75 MHz, $CDCl_3$) 3.5 $SiCH_2\underline{CH_3}$, 7.1 Si$\underline{CH_2}CH_3$, 11.5 C_2, 28.0 Ph$\underline{C}H_2$, 126.5 $C_{4'}$, 128.0, 128.4 $C_{2',3'}$, 141.1 $C_{1'}$, 181.7 C_1; *m/z* (CI+, NH_3) 247 ($[M+H]^+$, 100), 217 (18), 132 ($[SiEt_3+NH_3]^+$, 22); HRMS (CI+) Found $[M+H]^+$ 247.1510 $C_{15}H_{23}OSi$, requires 247.1518

2-Cyclohexyl-2-triethylsilylethen-1-one 1d

Carried out by the general procedure above with 1-cyclohexyl-2-diazo-2-triethylsilylethan-1-one **2d** (1.74 g, 6.53 mmol) and Rh_2(octanoate)$_4$ (0.051 g, 0.065 mmol) in benzene (15 ml) at 50°C. The crude product was purified by flash chromatography (petroleum ether) to yield 2-cyclohexyl-2-triethylsilylethen-1-one **1d** as a colourless liquid (1.209 g, 77 %).

ν_{max} (KBr/film) 2954 s, 2929 s, 2877 m, 2852 m, 2078 s (C=O), 1448 w, 1515 w, 1236 w, 1006 w, 732 m cm^{-1}; δ_H (300 MHz, $CDCl_3$) 0.62 (6 H, q, *J* = 8 Hz, Si<u>CH$_2$</u>CH$_3$), 0.95 (9 H, t, *J* = 8 Hz, SiCH$_2$<u>CH$_3$</u>), 1.17-1.27 (5 H, m), 1.61-1.88 (6 H, m); δ_C (75 MHz, $CDCl_3$) 4.0 Si<u>CH$_2$</u>CH$_3$, 7.1 SiCH$_2$<u>CH$_3$</u>, 17.2 C_2, 25.9 $C_{4'}$, 26.3 $C_{3'}$, 33.8 $C_{1'}$, 36.4 $C_{2'}$, 182.6 C_1; *m/z* (CI+, NH_3) 239 ($[M+H]^+$, 100), 132 ($[SiEt_3+NH_3]^+$, 17); HRMS (CI+) Found $[M+H]^+$ 239.1841 $C_{14}H_{27}OSi$, requires 239.1831

2-Phenyl-2-triethylsilylethen-1-one 1e

Carried out by the general procedure above with 2-diazo-1-phenyl-2-triethylsilylethan-1-one **2e** (0.96 g, 3.68 mmol) and Rh_2(octanoate)$_4$ (0.029 g, 0.0368 mmol) in benzene (8 ml) at 50°C. The crude product was purified by flash chromatography (petroleum ether) to yield 2-phenyl-2-triethylsilylethen-1-one **1e** as a colourless liquid (0.365 g, 43 %).

ν_{max} (KBr/film) 3059 w, 2957 m, 2877 m, 2082 s (C=O), 1597 m, 1496 m, 1463 w, 1416 w, 1318 w, 1167 w, 1074 w, 1032 w, 1004 m, 920 m, 752 m, 737 m, 723 m, 706 m, 694 m cm^{-1}; δ_H (300 MHz, $CDCl_3$) 0.75 (6 H, q, *J* = 8 Hz, Si<u>CH$_2$</u>CH$_3$), 0.97 (9 H, t, *J* = 8 Hz, SiCH$_2$<u>CH$_3$</u>), 7.07-7.10 (3 H, m, $H_{3',4'}$), 7.21-7.26 (2 H, m, $H_{2'}$); δ_C (75 MHz, $CDCl_3$) 4.0 Si<u>CH$_2$</u>CH$_3$, 7.1 SiCH$_2$<u>CH$_3$</u>, 20.2 C_2, 124.6 $C_{4'}$, 128.1, 128.9 $C_{2',3'}$, 131.8 $C_{1'}$, 181.8 C_1; *m/z* (CI+, NH_3) 250 ($[M+NH_4]^+$, 15), 233 ($[M+H]^+$, 27), 154 (53), 132 ($[SiEt_3+NH_3]^+$, 49), 118 (59); HRMS (CI+) Found $[M+H]^+$ 233.1367 $C_{14}H_{21}OSi$, requires 233.1362

2-(4-Methoxyphenyl)-2-triethylsilylethen-1-one 1f

Carried out by the general above with 2-diazo-1-(4-methoxyphenyl)-2-triethylsilylethan-1-one **2f** (0.157 g, 0.54 mmol) and Rh_2(octanoate)$_4$ (0.004 g, 0.0054 mmol) in benzene (3 ml) at 50°C. The crude product was purified by flash chromatography (petroleum ether) to yield 2-(4-methoxyphenyl)-2-triethylsilylethen-1-one **1f** as a colourless liquid (0.033 g, 23 %).

ν_{max} (KBr/film) 2956 s, 2912 m, 2877 m, 2085 s (C=O), 1606 w, 1576 w, 1510 s, 1465 w, 1282 m, 1245 s, 1180 w, 1038 m, 1007 w, 913 w, 826 m cm^{-1}; δ_H (270 MHz, $CDCl_3$) 0.71 (6 H, q, *J* = 8 Hz, Si<u>CH$_2$</u>CH$_3$), 0.96 (9 H, t, *J* = 8 Hz, SiCH$_2$<u>CH$_3$</u>), 3.76 (3 H, s, OCH_3), 6.81 (2 H,

d, $J_{3'-2'}$ = 9 Hz, $H_{3'}$), 7.01 (2 H, d, $J_{2'-3'}$ = 9 Hz, $H_{2'}$), δ_C (62.5 MHz, $CDCl_3$) 3.9 Si$\underline{CH_2}$$CH_3$, 7.1 $SiCH_2$$\underline{CH_3}$, 18.5 C_2, 55.3 OCH_3, 114.5 $C_{3'}$, 122.7 $C_{1'}$, 129.6 $C_{2'}$, 157.2 $C_{4'}$, 182.1 C_1; *m/z* (CI+, NH_3) 263 ($[M+H]^+$, 45), 262 ($[M]^+$, 100), 234 (52), 132 ($[SiEt_3+NH_3]^+$, 39); HRMS (CI+) Found $[M+H]^+$ 263.1467 $C_{15}H_{23}O_2Si$, requires 263.1472

2-(4-Bromophenyl)-2-triethylsilylethen-1-one 1g

Carried out by the general procedure above with 2-diazo-1-(4-bromophenyl)-2-triethylsilylethan-1-one **2g** (0.9 g, 2.65 mmol) and $Rh_2(octanoate)_4$ (0.021 g, 0.0265 mmol) in benzene (10 ml) at 60°C. The crude product was purified by flash chromatography (petroleum ether) to yield 2-(4-bromophenyl)-2-triethylsilylethen-1-one **1g** as a colourless liquid (0.32 g, 40 %).

ν_{max} (KBr/film) 2956 s, 2910 m, 2977 s, 2088 s (C=O), 1486 s, 1166 m, 1076 m, 1008 s, 914 w, 817 s, 736 s, 723 s cm^{-1}; δ_H (300 MHz, $CDCl_3$) 0.77 (6 H, q, *J* = 8 Hz, Si$\underline{CH_2}$$CH_3$), 0.98 (9 H, t, *J* = 8 Hz, $SiCH_2$$\underline{CH_3}$), 6.96 (2 H, d, $J_{3'-2'}$ = 8.5 Hz, $H_{3'}$), 7.36 (2 H, d, $J_{2'-3'}$ = 8.5 Hz, $H_{2'}$); δ_C (75 MHz, $CDCl_3$) 3.9 Si$\underline{CH_2}$$CH_3$, 7.1 $SiCH_2$$\underline{CH_3}$, 19.9 C_2, 118.0 $C_{4'}$, 129.5, 132.0 $C_{4',2'}$, 131.1 $C_{1'}$, 181.1 C_1; *m/z* (CI+, NH_3) 348 (20), 346 (19), 313 ($[M^{81}Br+H]^+$, 35), 311 ($[M^{79}Br+H]^+$, 33), 244 (14), 242 (15), 233 ($[M-Br+2H]^+$, 12) 227 (20), 225 (19), 132 ($[SiEt_3+NH_3]^+$, 100), 52 (35); HRMS (CI+) Found $[M+H]^+$ 313.0437 $C_{14}H_{20}{}^{81}BrOSi$, requires 313.0446

2-(2-Furyl)-2-triethylsilylethen-1-one 1h

Carried out by the general procedure above with 2-diazo-1-(2-furyl)-2-triethylsilylethan-1-one **2h** (0.375 g, 1.5 mmol) and $Rh_2(octanoate)_4$ (0.011 g, 0.015 mmol) in benzene (5 ml) at 60°C. The crude product was purified by flash chromatography (petroleum ether) to yield 2-(2-furyl)-2-triethylsilylethen-1-one **1h** as a pale yellow liquid (0.132 g, 40 %).

ν_{max} (KBr/film) 2958 m, 2878 m, 2099 s (C=O), 1587 m, 1510 w, 1500 w, 1462 w, 1416 w, 1217 m, 1181 m, 1074 w, 1015 s cm^{-1}; δ_H (300 MHz, $CDCl_3$) 0.74 (6 H, q, *J* = 8 Hz, Si$\underline{CH_2}$$CH_3$), 0.96 (9 H, t, *J* = 8 Hz, $SiCH_2$$\underline{CH_3}$), 5.89 (1 H, dd, $J_{3'-4'}$ = 3 Hz, $J_{3'-5'}$ = 0.5 Hz, $H_{3'}$), 6.31 (1 H, dd, $J_{4'-3'}$ = 3 Hz, $J_{4'-5'}$ = 2 Hz, $H_{4'}$), 7.26 (1 H, dd, $J_{5'-4'}$ = 2 Hz, $J_{5'-3'}$ = 0.5 Hz, $H_{5'}$); δ_C

(75 MHz, $CDCl_3$) 3.8 $Si\underline{CH_2}CH_3$, 7.0 $SiCH_2\underline{CH_3}$, 14.0 C_2, 104.7, 111.2 $C_{3',4'}$, 140.5 $C_{5'}$, 143.4 $C_{2'}$, 182.6 C_1; *m/z* (CI+, NH_3) 223 ($[M+H]^+$, 100), 132 ($[SiEt_3+NH_3]^+$, 26), 104 (40); HRMS (CI+) Found $[M+H]^+$ 223.1174 $C_{12}H_{19}N_2O_2Si$, requires 223.1154

2-(2-Thiophenyl)-2-triethylsilylethen-1-one 1i

Carried out by the general procedure above with 2-diazo-1-(2-thiophenyl)-2-triethylsilylethan-1-one **2i** (0.55 g, 2.06 mmol) and Rh_2(octanoate)$_4$ (0.016 g, 0.02 mmol) in benzene (4 ml) at 60°C. The crude product was purified by flash chromatography (petroleum ether) to yield 2-(2-thiophenyl)-2-triethylsilylethen-1-one **1i** as a pale yellow liquid (0.190 g, 39 %).

ν_{max} (KBr/film) 2956 m, 2911 w, 2877 w, 2089 s (C=O), 1526 w, 1457 w, 1415 w, 1238 w, 1132 w, 1004 m, 864 m, 841 m, 722 s, 687 s cm^{-1}; δ_H (300 MHz, $CDCl_3$) 0.75 (6 H, q, *J* = 8 Hz, $Si\underline{CH_2}CH_3$), 0.99 (9 H, t, *J* = 8 Hz, $SiCH_2\underline{CH_3}$), 6.70 (1 H, dd, $J_{5'-4'}$ = 3.5 Hz, $J_{5'-3'}$ = 1 Hz, $H_{5'}$), 6.95 (1 H, dd, $J_{4'-3'}$ = 5 Hz, $J_{4'-5'}$ = 3.5 Hz, $H_{4'}$), 7.11 (1 H, dd, $J_{3'-4'}$ = 5 Hz, $J_{3'-5'}$ = 1 Hz, $H_{3'}$); δ_C (75 MHz, $CDCl_3$) 3.7 $Si\underline{CH_2}CH_3$, 7.1 $SiCH_2\underline{CH_3}$, 14.4 C_2, 123.1, 124.8 $C_{3',4'}$, 127.3 $C_{5'}$, 131.4 $C_{2'}$, 180.3 C_1; *m/z* (CI+, NH_3) 239 ($[M+H]^+$, 100), 238 ($[M]^+$, 57), 132 ($[SiEt_3+NH_3]^+$, 22); HRMS (CI+) Found $[M+H]^+$ 239.0920 $C_{12}H_{19}OSSi$, requires 239.0926

2-(3-(1-*tert*-Butoxycarbonyl)indolyl)-2-triethylsilylethen-1-one 1j

Carried out by the general procedure above with 1-(3-(1-*tert*-butoxycarbonyl)indolyl)-2-diazo-2-triethylsilylethan-1-one **2j** (0.202 g, 0.5 mmol) and Rh_2(octanoate)$_4$ (0.004 g, 0.005 mmol) in benzene (3 ml) at 40°C. The crude product was purified by flash chromatography (petroleum ether) to yield 2-(3-(1-*tert*-butoxycarbonyl)indolyl)-2-triethylsilylethen-1-one **1j** as a colourless liquid (0.094 g, 51 %).

ν_{max} (KBr/film) 3054 w, 2955 m, 2912 m, 2877 m, 2085 s (C=O), 1733 s (C=O Boc), 1476 m, 1456 m, 1418 m, 1373 m, 1298 m, 1246 m, 1215 m, 1158 m, 1123 w, 1096 m, 1042 w, 1021 m cm^{-1}; δ_H (270 MHz, $CDCl_3$) 0.77 (6 H, q, *J* = 8 Hz, $Si\underline{CH_2}CH_3$), 0.99 (9 H, t, *J* = 8 Hz, $SiCH_2\underline{CH_3}$), 1.66 (9 H, s, $C(\underline{CH_3})$)), 7.21-7.34 (3 H, m, *Ind*H), 7.58 (1 H, dd, *J* = 7 Hz, *J* = 1.5 Hz, *Ind*H), 8.07 (1 H, br d, *J* = 7 Hz, *Ind*H); δ_C (75 MHz, $CDCl_3$) 3.8 $Si\underline{CH_2}CH_3$, 7.1 $SiCH_2\underline{CH_3}$,

9.2 C_2, 28.2 $C(\underline{C}H_3)$, 83.6 $\underline{C}(CH_3)$, 115.3, 119.1, 121.2, 122.5, 124.7 $C_{2',4',5',6',7'}$, 108.7, 130.7, 135.0 $C_{3',3a',7a'}$, 149.6 C=O, Boc, 181.0 C_1; *m/z* (CI+, NH_3) 372 ($[M+H]^+$, 100), 344 ($[M-N_2+H]^+$, 86), 315 (92), 286 (17), 271 (60), 242 (20), 132 ($[SiEt_3+NH_3]^+$, 27), 104 (12); HRMS (CI+) Found $[M+H]^+$ 372.1988 $C_{21}H_{30}NO_3Si$, requires 372.1995

General procedure for the preparation of allenylsilanes from silyl ketenes and stabilised phosphorus ylides

Ethyl 2-methyl-4-triethylsilylpenta-2,3-dienoate 6a

To a solution of 2-triethylsilylpropen-2-one **1a** (0.031 g, 0.18 mmol) in toluene (1.5 ml) under a nitrogen atmosphere was added in one portion (1-ethoxycarbonyl-ethylidene)triphenylphosporane (**4**, 0.073 g, 0.20 mmol). The mixture was then heated to gentle reflux and monitored by TLC for complete consumption of the silylketene. Hexane (10 ml) was then added and the white precipitate removed by filtration. The filtrate was concentrated under reduced pressure and the residual oil purified by flash chromatography (95:5 petroleum ether:diethyl ether) to yield ethyl 2-methyl-4-triethylsilylpenta-2,3-dienoate **6a** as a colourless liquid (0.025 g, 54 %).

ν_{max} (KBr/film) 2957 s, 2936 m, 2876 m, 1932 s (C=C=C), 1705 s (C=O), 1458 w, 1267 s, 1238 w, 1119 m, 1003 w, 737 m, 719 m, 712 m cm^{-1}; δ_H (300 MHz, $CDCl_3$) 0.59 (6 H, q, *J* = 8 Hz, $Si\underline{CH_2}CH_3$), 0.93 (9 H, t, *J* = 8 Hz, $SiCH_2\underline{CH_3}$), 1.21 (3 H, t, *J* = 7 Hz, $CH_2\underline{CH_3}$), 1.73 (3 H, s, CH_3), 1.78 (3 H, s, CH_3), 4.07 (1 H, dq, *J* = 11 Hz, *J* = 7 Hz, $\underline{CH_2}CH_3$), 4.19 (1 H, dq, *J* = 11 Hz, *J* = 7 Hz, $\underline{CH_2}CH_3$); δ_C (67.5 MHz, $CDCl_3$) 2.8 $SiCH_2\underline{CH_3}$, 7.1 $Si\underline{CH_2}CH_3$, 14.3, 14.6, 14.7 $CH_2\underline{CH_3}$, 2-CH_3, C_5, 60.3 $\underline{CH_2}CH_3$, 88.4 C_2, 90.7 C_4, 160.8 C=O, 208.8 C_3; *m/z* (CI+, NH_3) 272 ($[M+NH_4]^+$, 50), 255 ($[M+H]^+$, 100); HRMS (CI+) Found $[M+H]^+$ 255.1781 $C_{14}H_{27}O_2Si$, requires 255.1780

Ethyl 4-triethylsilylpenta-2,3-dienoate 7a

Carried out by the general procedure above with 2-triethylsilylpropen-2-one **1a** (0.064 g, 0.375 mmol) and (ethoxycarbonylmethylene)triphenylphosporane (**5**, 0.157 g, 0.45 mmol) in DCM (2 ml) at rt. The crude product was purified by flash chromatography (95:5 petroleum

ether:diethyl ether) to yield ethyl 4-triethylsilylpenta-2,3-dienoate **7a** as a colourless liquid (0.054 g, 60 %).

ν_{max} (KBr/film) 2956 m, 2914 w, 2877 w, 1933 s (C=C=C), 1714 s (C=O), 1457 w, 1402 w, 1382 w, 1248 s, 1200 s, 1145 s, 1044 w, 1005 w, 817 w, 806 w, 728 m, 723 m cm^{-1}; δ_H (270 MHz, $CDCl_3$) 0.61 (6 H, q, J = 8 Hz, $Si\underline{CH_2}CH_3$), 0.93 (9 H, t, J = 8 Hz, $SiCH_2\underline{CH_3}$), 1.21 (3 H, t, J = 7 Hz, $CH_2\underline{CH_3}$), 1.75 (3 H, d, $J_{5\text{-}2}$ = 2.5 Hz, H_5), 4.06 (1 H, dq, J = 11 Hz, J = 7 Hz, H_a or H_b), 4.16 (1 H, dq, J = 11 Hz, J = 7 Hz, H_b or H_a), 5.16 (1 H, q, $J_{2\text{-}5}$ = 2.5 Hz, H_2); δ_C (75 MHz, $CDCl_3$) 2.6 $Si\underline{CH_2}CH_3$, 7.0 $SiCH_2\underline{CH_3}$, 14.2, 14.4 $CH_2\underline{CH_3}$, CH_3, 60.2 $\underline{CH_2}CH_3$, 81.2 C_2, 91.9 C_4, 168.1 C=O ,209.1 C_3; *m/z* (CI+, NH_3) 258 ($[M+NH_4]^+$, 40), 241 ($[M+H]^+$, 100); HRMS (CI+) Found $[M+H]^+$ 241.1619 $C_{13}H_{25}O_2Si$, requires 241.1624

Ethyl 2-methyl-4-triethylsilylundeca-2,3-dienoate 6b

Carried out by the general procedure above with 2-triethylsilylnon-1-en-1-one **1b** (0.076 g, 0.3 mmol) and (1-ethoxycarbonylethylidene)triphenylphosporane (**4**, 0.114 g, 0.315 mmol) in refluxing toluene (3 ml). The crude product was purified by flash chromatography (95:5 petroleum ether:diethyl ether) to yield ethyl 2-methyl-4-triethylsilylundeca-2,3-dienoate **6b** as a colourless liquid (0.045 g, 45 %).

ν_{max} (KBr/film) 2956 s, 2926 s, 2875 s, 2857 m, 1929 s (C=C=C), 1705 s (C=O), 1458 w, 1267 s, 1236 w, 1118 s, 1004 w, 735 m, 720 m cm^{-1}; δ_H (270 MHz, $CDCl_3$) 0.59 (6 H, q, J = 8 Hz, $Si\underline{CH_2}CH_3$), 0.86 (3 H, t, $J_{11\text{-}10}$ = 6.5 Hz, H_{11}), 0.92 (9 H, t, J = 8 Hz, $SiCH_2\underline{CH_3}$), 1.20 (3 H, t, J = 7 Hz, $CH_2\underline{CH_3}$), 1.20-1.32 (8 H, m, $H_{7,8,9,10}$), 1.44 (2 H, p, J = 7.5 Hz, H_6), 1.78 (3 H, s, 2-CH_3), 2.00 (2 H, t, $J_{5\text{-}6}$ = 6.5 Hz, H_5), 4.09 (1 H, dq, J = 10.5 Hz, J = 7 Hz, $\underline{CH_2}CH_3$), 4.16 (1 H, dq, J = 10.5 Hz, J = 7 Hz, $\underline{CH_2}CH_3$); δ_C (75 MHz, $CDCl_3$) 3.0 $Si\underline{CH_2}CH_3$, 7.1 $SiCH_2\underline{CH_3}$, 14.1, 14.3, 14.5 C_{11}, 2-CH_3, $CH_2\underline{CH_3}$, 22.6, 29.0, 29.1, 29.2 (2 peaks) $C_{6,7,8,9,10}$, 31.8 C_5, 60.2 $\underline{CH_2}CH_3$, 89.8 C_4, 96.2 C_2, 169.9 C=O, 208.6 C_3; *m/z* (CI+, NH_3) 339 ($[M+H]^+$, 100); HRMS (CI+) Found $[M+H]^+$ 339.2714 $C_{20}H_{39}O_2Si$, requires 339.2719

Ethyl 4-triethylsilylundeca-2,3-dienoate 7b

Carried out by the general procedure above with 2-triethylsilylnon-1-en-1-one **1b** (0.382 g, 1.5 mmol) and (ethoxycarbonylmethylene) triphenylphosporane (**5**, 0.627 g, 1.8 mmol) in refluxing DCM (5 ml). The crude product was purified by flash chromatography (90:10 petroleum ether:diethyl ether) to yield ethyl 4-triethylsilylundeca-2,3-dienoate **7b** as a colourless liquid (0.375 g, 77 %).

ν_{max} (KBr/film) 2954 s, 2929 s, 2875 m, 2858 w, 1928 s (C=C=C), 1714 s (C=O), 1463 w, 1405 w, 1380 w, 1245 s, 1193 m, 1143 s, 1045 w, 1004 w, 806 w, 736 m, 723 m cm^{-1}; δ_H (270 MHz, $CDCl_3$) 0.62 (6 H, q, *J* = 8 Hz, Si$\underline{CH_2}CH_3$), 0.85 (3 H, t, J_{11-10} = 6.5 Hz, H_{11}), 0.94 (9 H, t, *J* = 8 Hz, $SiCH_2\underline{CH_3}$), 1.22 (3 H, t, *J* = 7 Hz, $CH_2\underline{CH_3}$), 1.19-1.36 (8 H, m, $H_{7,8,9,10}$), 1.47 (2 H, p, *J* = 7.5 Hz, H_6), 2.00 (2 H, m, H_5), 4.10 (1 H, dq, *J* = 11 Hz, *J* = 7 Hz, $\underline{CH_2}CH_3$), 4.17 (1 H, dq, *J* = 11 Hz, *J* = 7 Hz, $\underline{CH_2}CH_3$), 5.23 (1 H, t, J_{2-5} = 3 Hz, H_2); δ_C (75 MHz, $CDCl_3$) 2.8 $Si\underline{CH_2}CH_3$, 6.9 $SiCH_2\underline{CH_3}$, 13.9, 14.1 C_{11}, $CH_2\underline{CH_3}$, 22.5, 28.5, 28.8, 29.0, 29.1 $C_{6,7,8,9,10}$, 31.7 C_5, 60.0 $\underline{CH_2}CH_3$, 82.6 C_4, 97.2 C_2, 167.9 C=O, 208.7 C_3; *m/z* (CI+, NH_3) 342 ($[M+NH_4]^+$, 24), 325 ($[M+H]^+$, 100), 132 ($[SiEt_3+NH_3]^+$, 12); HRMS (CI+) Found $[M+H]^+$ 325.2567 $C_{19}H_{37}O_2Si$, requires 325.2563

Ethyl 2-methyl-5-phenyl-4-triethylsilylpenta-2,3-dienoate 6c

Carried out by the general procedure above with 3-phenyl-2-triethylsilylpropen-1-one **1c** (0.123 g, 0.5 mmol) and (1-ethoxycarbonyl-ethylidene) triphenylphosporane (**4**, 0.200 g, 0.55 mmol) in refluxing benzene (3 ml). The crude product was purified by flash chromatography (90:10 petroleum ether:diethyl ether) to yield ethyl 2-methyl-5-phenyl-4-triethylsilylpenta-2,3-dienoate **6c** as a colourless liquid (0.099 g, 60 %).

ν_{max} (KBr/film) 3026 w, 2953 m, 2909 m, 2875 m, 1929 s (C=C=C), 1704 s (C=O), 1454 w, 1267 s, 1238 w, 1120 s, 1006 m, 762 w, 736 m, 719 m, 699 m cm^{-1}; δ_H (300 MHz, $CDCl_3$) 0.51 (6 H, q, *J* = 8 Hz, $Si\underline{CH_2}CH_3$), 0.87 (9 H, t, *J* = 8 Hz, $SiCH_2\underline{CH_3}$), 1.25 (3 H, t, *J* = 7 Hz, $CH_2\underline{CH_3}$), 1.72 (3 H, s, CH_3), 3.32 (1 H, d, *J* = 15 Hz, H_5), 3.43 (1 H, d, *J* = 15 Hz, H_5), 4.13 (2 H, q, *J* = 7 Hz, $\underline{CH_2}CH_3$), 7.16-7.25 (5 H, m, Ph); δ_C (75 MHz, $CDCl_3$) 3.0 $Si\underline{CH_2}CH_3$, 7.0 $SiCH_2\underline{CH_3}$, 14.3 $CH_2\underline{CH_3}$ and CH_3 (2 peaks overlapped), 36.1 C_5, 60.3 $\underline{CH_2}CH_3$, 89.8 C_2, 95.9

C_4, 126.2 $C_{4'}$, 128.0, 128.8 $C_{2',3'}$, 139.4 $C_{1'}$, 169.4 C_1, 210.5 C_3; *m/z* (CI+, NH_3) 348 ($[M+NH_4]^+$, 25), 331 ($[M+H]^+$, 100); HRMS (CI+) Found $[M+H]^+$ 331.2102 $C_{20}H_{31}O_2Si$, requires 331.2093

Ethyl 5-phenyl-4-triethylsilylpenta-2,3-dienoate 7c

Carried out by the general procedure above with 3-phenyl-2-triethylsilylpropen-1-one **1c** (0.098 g, 0.4 mmol) and (ethoxycarbonylmethylene)triphenylphosporane (**5**, 0.153 g, 0.44 mmol) in DCM (2 ml) at rt. The crude product was purified by flash chromatography (90:10 petroleum ether:diethyl ether) to yield ethyl 5-phenyl-4-triethylsilylpenta-2,3-dienoate **7c** as a colourless liquid (0.103 g, 81 %).

ν_{max} (KBr/film) 3062 w, 3029 w, 2954 s, 2912 w, 2877 m, 1928 s (C=C=C), 1711 s (C=O), 1454 w, 1247 s, 1172 m, 1140 m, 1043 m, 1044 m, 804 w, 737 s, 723 s, 700 s cm^{-1}; δ_H (300 MHz, $CDCl_3$) 0.56 (6 H, q, *J* = 8 Hz, $Si\underline{CH_2}CH_3$), 0.90 (9 H, t, *J* = 8 Hz, $SiCH_2\underline{CH_3}$), 1.26 (3 H, t, *J* = 7 Hz, $CH_2\underline{CH_3}$), 3.41 (2 H, m, H_5), 4.15 (2 H, q, *J* = 7 Hz, $\underline{CH_2}CH_3$), 5.21 (1 H, t, *J* = 2.5 Hz, H_2), 7.18-7.27 (5 H, m, Ph); δ_C (75 MHz, $CDCl_3$) 2.9 $Si\underline{CH_2}CH_3$, 6.9 $SiCH_2\underline{CH_3}$, 14.2 $CH_2\underline{CH_3}$, 35.7 C_5, 60.2 $\underline{CH_2}CH_3$, 82.6 C_2, 96.9 C_4, 126.4 $C_{4'}$, 128.1, 128.8 $C_{2',3'}$, 138.8 $C_{1'}$, 167.7 C_1, 210.6 C_3; *m/z* (CI+, NH_3) 334 ($[M+NH_4]^+$, 88), 333 (37), 317 ($[M+H]^+$, 100), 316 ($[M]^+$, 64); HRMS (CI+) Found $[M+H]^+$ 317.1937 $C_{19}H_{29}O_2Si$, requires 317.1940

Ethyl 4-cyclohexyl-2-methyl-4-triethylsilylbuta-2,3-dienoate 6d

Carried out by the general procedure above with 2-cyclohexyl-2-triethylsilylethen-1-one **1d** (0.072 g, 0.3 mmol) and (1-ethoxycarbonylethylidene)triphenylphosporane (**4**, 0.120 g, 0.33 mmol) in refluxing toluene (2 ml). After 48 h the crude product was purified by flash chromatography (90:10 petroleum ether:diethyl ether) to yield ethyl 4-cyclohexyl-2-methyl-4-triethylsilylbuta-2,3-dienoate **6d** as a colourless liquid (0.021 g, 22 %) and recovered 2-cyclohexyl-2-triethylsilylethen-1-one **1d** (0.043 g, 60 %).

ν_{max} (KBr/film) 2952 s, 2927 s, 2875 m, 2852 w, 1924 m (C=C=C), 1704 s (C=O), 1448 w, 1267 s, 1240 w, 1118 s, 1006 w, 732 m, 721 m cm^{-1}; δ_H (270 MHz, $CDCl_3$) 0.59 (6 H, q, *J* = 8

Hz, Si$\underline{CH_2}CH_3$), 0.92 (9 H, t, *J* = 8 Hz, $SiCH_2\underline{CH_3}$), 1.11-1.30 (5 H, m), 1.19 (3 H, t, *J* = 7 Hz, $CH_2\underline{CH_3}$), 1.56-1.90 (6 H, m), 1.78 (3 H, s, CH_3), 4.11 (2 H, q, *J* = 7 Hz, $\underline{CH_2}CH_3$); δ_C (75 MHz, $CDCl_3$) 3.3 $Si\underline{CH_2}CH_3$, 7.2 $SiCH_2\underline{CH_3}$, 14.3, 14.5 CH_3, $CH_2\underline{CH_3}$, 26.0, 26.6 $C_{3',4'}$, 34.0, 34.4 $C_{2'a,2'e}$, 60.1 $\underline{CH_2}CH_3$, 90.6 C_2, 101.9 C_4, 169.9 C=O, 209.5 C_3; *m/z* (CI+, NH_3) 323 ($[M+H]^+$, 100), 193 (18); HRMS (CI+) Found $[M+H]^+$ 323.2410 $C_{19}H_{35}O_2Si$, requires 323.2406

Ethyl 4-cyclohexyl-4-triethylsilylbuta-2,3-dienoate 7d

Carried out by the general procedure above with 2-cyclohexyl-2-triethylsilylethen-1-one **1d** (0.072 g, 0.3 mmol) and (ethoxycarbonylmethylene)triphenylphosporane (**5**, 0.115 g, 0.33 mmol) in refluxing benzene (2 ml). The crude product was purified by flash chromatography (90:10 petroleum ether:diethyl ether) to yield ethyl 4-cyclohexyl-4-triethylsilylbuta-2,3-dienoate **7d** as a colourless liquid (0.072 g, 78 %).

ν_{max} (KBr/film) 2952 s, 2929 s, 2875 m, 2852 m, 1920 s (C=C=C), 1712 s (C=O), 1243 s, 1191 s, 1145 s, 1045 m, 1006 m, 734 m, 721 m, 698 w cm^{-1}; δ_H (270 MHz, $CDCl_3$) 0.62 (6 H, q, *J* = 8 Hz, $Si\underline{CH_2}CH_3$), 0.92 (9 H, t, *J* = 8 Hz, $SiCH_2\underline{CH_3}$), 1.10-1.30 (5 H, m), 1.21 (3 H, t, *J* = 7 Hz, $CH_2\underline{CH_3}$), 1.55-1.92 (6 H, m), 4.11 (2 H, app dq, *J* = 2 and 7 Hz $\underline{CH_2}CH_3$), 5.23 (1 H, d, $J_{2-1'}$ = 0.5 Hz, H_2); δ_C (75 MHz, $CDCl_3$) 2.9 $Si\underline{CH_2}CH_3$, 7.1 $SiCH_2\underline{CH_3}$, 14.2 $CH_2\underline{CH_3}$, 25.9 $C_{4'}$, 26.6, 26.6, 34.0, 34.1 $C_{2'3'}$, 38.9 $C_{1'}$, 60.1 $\underline{CH_2}CH_3$, 83.4 C_2, 102.9 C_4, 168.2 C=O, 209.6 C_3; *m/z* (CI+, NH_3) 326 ($[M+NH_4]^+$, 24), 309 ($[M+H]^+$, 100); HRMS (CI+) Found $[M+H]^+$ 309.2245 $C_{18}H_{33}O_2Si$, requires 309.2250

Ethyl 2-methyl-4-phenyl-4-triethylsilylbuta-2,3-dienoate 6e and ethyl 2-methyl-4-phenylbuta-2,3-dienoate 8e

Carried out by the general procedure above with 2-phenyl-2-triethylsilylethen-1-one **1e** (0.097 g, 0.42 mmol) and (1-ethoxycarbonyl-ethylidene)triphenylphosporane (**4**, 0.167 g, 0.46 mmol) in refluxing benzene (3 ml). The crude product was purified by flash chromatography (95:5 petroleum ether:diethyl ether) to yield ethyl 2-methyl-4-phenyl-4-triethylsilylbuta-2,3-dienoate **6e** as a colourless liquid (0.072 g, 55 %) and ethyl 2-methyl-4-phenyl-buta-2,3-dienoate **8e** as a colourless liquid (0.006 g, 7 %).

6e: ν_{max} (KBr/film) 2954 s, 2910 m, 2875 m, 1926 s (C=C=C), 1706 s (C=O), 1596 w, 1490 w, 1267 s, 1240 m, 1118 s, 1004 m, 755 m, 734 m, 721 m, 698 m cm^{-1}; δ_H (300 MHz, $CDCl_3$) 0.73 (6 H, q, *J* = 8 Hz, Si$\underline{CH_2}CH_3$), 0.97 (9 H, t, *J* = 8 Hz, $SiCH_2\underline{CH_3}$), 1.28 (3 H, t, *J* = 7 Hz, $CH_2\underline{CH_3}$), 1.91 (3 H, s, CH_3), 4.15 (1 H, dq, *J* = 11 Hz, *J* = 7 Hz, $\underline{CH_2}CH_3$), 4.22 (1 H, dq, *J* = 11 Hz, *J* = 7 Hz, $\underline{CH_2}CH_3$), 7.22-7.34 (5 H, m, Ph); δ_C (75 MHz, $CDCl_3$) 3.6 $Si\underline{CH_2}CH_3$, 7.1 $SiCH_2\underline{CH_3}$, 14.3, 14.4 CH_3,CH_2CH_3, 60.6 $\underline{CH_2}CH_3$, 90.6 C_2, 99.9 C_4, 126.7 $C_{4'}$, 127.9, 128.6 $C_{3',2'}$, 136.1 $C_{1'}$, 169.0 C_1, 212.3 C_3; *m/z* (CI+, NH_3) 334 ($[M+NH_4]^+$, 100), 317 ($[M+H]^+$, 22); HRMS (CI+) Found $[M+H]^+$ 317.1925 $C_{19}H_{29}O_2Si$, requires 317.1937

8e: ν_{max} (KBr/film) 2983 w, 2960 w, 2931 w, 1947 w (C=C=C), 1710 s (C=O), 1459 w, 1367 w, 1268 s, 1189 w, 1120 w, 1027 w, 765 w, 734 w, 692 m cm^{-1}; δ_H (300 MHz, $CDCl_3$) 1.25 (3 H, t, *J* = 7 Hz, $CH_2\underline{CH_3}$), 1.99 (3 H, d, *J* = 3 Hz, CH_3), 4.20 (2 H, q, *J* = 7 Hz, $\underline{CH_2}CH_3$), 6.46 (1 H, q, *J* = 3 Hz, H_4), 7.21-7.35 (5 H, m, Ph); δ_C (75 MHz, $CDCl_3$) 14.3, 15.1 CH_3, CH_2CH_3, 61.1 $\underline{CH_2}CH_3$, 97.2 C_4, 99.4 C_2, 127.3, 128.8 $C_{2',3'}$, 127.6 $C_{4'}$, 132.5 $C_{1'}$, 167.1 C_1, 212.3 C_3; *m/z* (CI+, NH_3) 220 ($[M+NH_4]^+$, 100), 203 ($[M+H]^+$, 21); HRMS (CI+) Found $[M+H]^+$ 203.1076 $C_{13}H_{15}O_2$, requires 203.1072

Ethyl 4-(4-methoxyphenyl)-2-methyl-4-triethylsilylbuta-2,3-dienoate 6f and ethyl 4-(4-methoxyphenyl)-2-methyl-buta-2,3-dienoate 8f

Carried out by the general procedure above with 2-(4-methoxyphenyl)-2-triethylsilylethen-1-one **1f** (0.118 g, 0.45 mmol) and (1-ethoxycarbonyl-ethylidene)triphenylphosporane (**4**, 0.181 g, 0.66 mmol) in benzene (4 ml) at 50 °C. The crude product was purified by flash chromatography (90:10 petroleum ether:diethyl ether) to yield ethyl 4-(4-methoxyphenyl)-2-methyl-4-triethylsilylbuta-2,3-dienoate **6f** as a colourless liquid (0.068 g, 44 %) and ethyl 4-(4-methoxyphenyl)-2-methyl-buta-2,3-dienoate **8f** as a colourless liquid (0.003 g, 3 %).

6f: ν_{max} (KBr/film) 2955 s, 2910 m, 2875 m, 1924 s (C=C=C), 1703 s (C=O), 1605 m, 1509 s, 1462 m, 1367 w, 1286 s, 1264 s, 1246 s, 1174 m, 1117 s, 1035 m, 1006 m, 829 m, 735 m, 701 m cm^{-1}; δ_H (270 MHz, $CDCl_3$) 0.70 (6 H, q, *J* = 8 Hz, $Si\underline{CH_2}CH_3$), 0.94 (9 H, t, *J* = 8 Hz, $SiCH_2\underline{CH_3}$), 1.22 (3 H, t, *J* = 7 Hz, $CH_2\underline{CH_3}$), 1.88 (3 H, s, CH_3), 3.78 (3 H, s, OCH_3), 4.12 (1

H, dq, J = 11 Hz, J = 7 Hz, $\underline{CH_2}CH_3$), 4.20 (1 H, dq, J = 11 Hz, J = 7 Hz, $\underline{CH_2}CH_3$), 6.84 (2 H, d, $J_{3'-2'}$ = 9 Hz, $H_{3'}$), 7.17 (2 H, d, $J_{2'-3'}$ = 9 Hz, $H_{2'}$); δ_C (75 MHz, $CDCl_3$) 3.6 $Si\underline{CH_2}CH_3$, 7.1 $SiCH_2\underline{CH_3}$, 14.3, 14.5 CH_3, $CH_2\underline{CH_3}$, 55.2 OCH_3, 60.5 $\underline{CH_2}CH_3$, 90.7 C_2, 99.1 C_4, 114.1 $C_{3'}$, 127.9 $C_{1'}$, 128.9 $C_{2'}$, 158.5 $C_{4'}$, 169.1 C_1, 212.2 C_3; *m/z* (CI+, NH_3) 364 ($[M+NH_4]^+$, 26), 347 ($[M+H]^+$, 100); HRMS (CI+) Found $[M+H]^+$ 347.2046 $C_{20}H_{31}O_3Si$, requires 347.2042

Ehyl 4-(2-furyl)-2-methyl-4-triethylsilylbuta-2,3-dienoate 6h and ethyl 4-(2-furyl)-2-methylbuta-2,3-dienoate 8h

Carried out by the general procedure above with 2-(2-furyl)-2-triethylsilylethen-1-one **1h** (0.132 g, 0.6 mmol) and (1-ethoxycarbonyl-ethylidene)triphenylphosporane (**4**, 0.236 g, 0.5 mmol) in benzene (3 ml) at 50 °C. The crude product was purified by flash chromatography (90:10 petroleum ether:diethyl ether) to yield ethyl 4-(2-furyl)-2-methyl-4-triethylsilylbuta-2,3-dienoate **6h** as a colourless liquid (0.012 g, 7 %) and ethyl 4-(2-furyl)-2-methyl-buta-2,3-dienoate **8h** as a colourless liquid (0.026 g, 23 %).

6h:_ν_{max} (KBr/film) 2957 s, 2912 m, 2877 s, 1926 s (C=C=C), 1708 s (C=O), 1462 m, 1368 m, 1265 s, 1118 s, 1019 s, 959 m, 859 w, 761 w, 735 s, 701 m cm^{-1}; δ_H (270 MHz, $CDCl_3$) 0.73 (6 H, q, J = 8 Hz, $Si\underline{CH_2}CH_3$), 0.94 (9 H, t, J = 8 Hz, $SiCH_2\underline{CH_3}$), 1.20 (3 H, t, J = 7 Hz, $CH_2\underline{CH_3}$), 1.90 (3 H, s, CH_3), 4.08 (1 H, dq, J = 11 Hz, J = 7 Hz, $\underline{CH_2}CH_3$), 4.21 (1 H, dq, J = 11 Hz, J = 7 Hz, $\underline{CH_2}CH_3$), 6.23 (1 H, dd, $J_{3'-4'}$ = 3 Hz, $J_{3'-5'}$ = 0.5 Hz, $H_{3'}$), 6.36 (1 H, dd, $J_{4'-3'}$ = 3 Hz, $J_{4'-5'}$ = 1.5 Hz, $H_{4'}$), 7.36 (1 H, dd, $J_{5'-4'}$ = 1.5 Hz, $J_{5'-3'}$ = 0.5 Hz, $H_{5'}$); δ_C (75 MHz, $CDCl_3$) 3.5 $Si\underline{CH_2}CH_3$, 7.1 $SiCH_2\underline{CH_3}$, 14.3, 14.6 CH_3, $CH_2\underline{CH_3}$, 60.7 $\underline{CH_2}CH_3$, 91.1, 92.5 $C_{2,4}$, 107.9, 111.3 $C_{3',4'}$, 142.0 $C_{5'}$, 147.9 $C_{2'}$, 168.4 C_1, 212.7 C_3; *m/z* (CI+, NH_3) 324 ($[M+NH_4]^+$, 35), 307 ($[M+H]^+$, 100); HRMS (CI+) Found $[M+H]^+$ 307.1733 $C_{17}H_{27}O_3Si$, requires 307.1729

8h: ν_{max} (KBr/film) 2983 m, 2930 w, 2907 w, 1948 w (C=C=C), 1711 s (C=O), 1447 w, 1369 m, 1270 s, 1171 m, 1120 s, 1013 m, 926 m, 739 s cm^{-1}; δ_H (270 MHz, $CDCl_3$) 1.24 (3 H, t, J = 7 Hz, $CH_2\underline{CH_3}$), 1.97 (3 H, d, J = 3 Hz, CH_3), 4.16 (1 H, dq, J = 11 Hz, J = 7 Hz, $\underline{CH_2}CH_3$), 4.22 (1 H, dq, J = 11 Hz, J = 7 Hz, $\underline{CH_2}CH_3$), 6.28 (1 H, d, $J_{3'-4'}$ = 3 Hz, $H_{3'}$), 6.38 (1 H, dd, $J_{4'-}$

$_{3'}$ = 3 Hz, $J_{4'-5'}$ = 2 Hz, $H_{4'}$), 6.42 (1 H, q, J = 3 Hz, H_4), 7.37 (1 H, dd, $J_{5'-4'}$ = 2 Hz, $J_{5'-3'}$ = 0.5 Hz, $H_{5'}$); δ_C (75 MHz, $CDCl_3$) 14.2, 15.4 CH_3, $CH_2\underline{CH_3}$, 61.2 $\underline{CH_2}CH_3$, 87.9 C_4, 100.1 C_2, 108.9, 111.5 $C_{3',4'}$, 142.7 $C_{5'}$, 146.1 $C_{2'}$, 166.6 C_1, 211.8 C_3; *m/z* (CI+, NH_3) 193 ($[M+H]^+$, 100), 119 (80); HRMS (CI+) Found $[M+H]^+$ 193.0864 $C_{11}H_{13}O_3$, requires 193.0865

Ethyl 2-methyl-4-(2-thiophenyl)-buta-2,3-dienoate 8i

Carried out by the general procedure above with 2-(2-thiophenyl)-2-triethylsilylethen-1-one **1i** (0.036 g, 0.15 mmol) and (1-ethoxycarbonylethylidene)triphenylphosporane (**4**, 0.060 g, 0.165 mmol) in DCM (1 ml) at rt. The crude product was purified by flash chromatography (90:10 petroleum ether:diethyl ether) to yield ethyl 2-methyl-4-(2-thiophenyl)-buta-2,3-dienoate **8i** as a colourless liquid (0.021 g, 67 %).

ν_{max} (KBr/film) 2983 m, 2930 w, 2907 w, 1948 w (C=C=C), 1711 s (C=O), 1447 w, 1369 m, 1270 s, 1171 m, 1120 s, 1013 m, 926 m, 739 s cm^{-1}; δ_H (270 MHz, $CDCl_3$) 1.24 (3 H, t, J = 7 Hz, $CH_2\underline{CH_3}$), 1.97 (3 H, d, J = 3 Hz, CH_3), 4.19 (2 H, app dq, J_{a-b} = 16 Hz, J = 7 Hz, $\underline{CH_2}CH_3$), 6.28 (1 H, d, $J_{3'-4'}$ = 3 Hz, $H_{3'}$), 6.38 (1 H, dd, $J_{4'-3'}$ = 3 Hz, $J_{4'-5'}$ = 2 Hz, $H_{4'}$), 6.42 (1 H, q, J = 3 Hz, H_4), 7.37 (1 H, dd, $J_{5'-4'}$ = 2 Hz, $J_{5'-3'}$ = 0.5 Hz, $H_{5'}$); δ_C (75 MHz, $CDCl_3$) 14.2, 15.4 CH_3, $CH_2\underline{CH_3}$, 61.2 $\underline{CH_2}CH_3$, 87.9 C_4, 100.1 C_2, 108.9, 111.5 $C_{3',4'}$, 142.7 $C_{5'}$, 146.1 $C_{2'}$, 166.6 C_1, 211.8 C_3; *m/z* (CI+, NH_3) 193 ($[M+H]^+$, 100), 119 (80); HRMS (CI+) Found $[M+H]^+$ 193.0864 $C_{11}H_{13}O_3$, requires 193.0865

Ethyl 4-(2-thiophenyl)-4-triethylsilylbuta-2,3-dienoate 7i

Carried out by the general procedure above with 2-(2-thiophenyl)-2-triethylsilylethen-1-one **1i** (0.036 g, 0.15 mmol) and (ethoxycarbonylmethylene)triphenylphosporane (**5**, 0.058 g, 0.165 mmol) in DCM (1 ml) at rt. The crude product was purified by flash chromatography (90:10 petroleum ether:diethyl ether) to yield ethyl 4-(2-thiophenyl)-4-triethylsilylbuta-2,3-dienoate **7i** as a colourless liquid (0.045 g, 98 %).

ν_{max} (KBr/film) 2983 m, 2930 w, 2907 w, 1948 w (C=C=C), 1711 s (C=O), 1447 w, 1369 m, 1270 s, 1171 m, 1120 s, 1013 m, 926 m, 739 s cm^{-1}; δ_H (300 MHz, $CDCl_3$) 0.78 (6 H, q, J = 8

Hz, Si$\underline{CH_2}$CH$_3$), 0.97 (9 H, t, *J* = 8 Hz, SiCH$_2\underline{CH_3}$), 1.23 (3 H, t, *J* = 7 Hz, CH$_2\underline{CH_3}$), 4.17 (2 H, app dq, $J_{a\text{-}b}$ = 16 Hz, *J* = 7 Hz, $\underline{CH_2}$CH$_3$), 5.57 (1 H, s, H$_2$), 6.92-6.97 (2 H, m, $H_{3',4'}$), 7.20 (1 H, dd, $J_{5'\text{-}4'}$ = 5 Hz, $J_{5'\text{-}3'}$ = 1.5 Hz, $H_{5'}$); δ_C (75 MHz, CDCl$_3$) 3.5 Si$\underline{CH_2}$CH$_3$, 7.1 SiCH$_2\underline{CH_3}$, 14.2 CH$_2\underline{CH_3}$, 60.7 $\underline{CH_2}$CH$_3$, 84.5 C$_2$, 95.3 C$_4$, 125.4 $C_{3',4'}$ (2 peaks), 127.3 $C_{5'}$, 136.2 $C_{2'}$, 166.5 C=O, 213.1 C$_3$; *m/z* (CI+, NH$_3$) 326 ([M+NH$_4$]$^+$, 17), 309 ([M+H]$^+$, 100), 148 (41); HRMS (CI+) Found [M+H]$^+$ 309.1341 $C_{16}H_{25}O_2SSi$, requires 309.1345

Ethyl 2-methyl-4-(3-(1-tert-butoxycarbonyl)indolyl)-4-triethylsilyl buta-2,3-dienoate 6j and ethyl 2-methyl-4-(3-(1-tert-butoxycarbonyl)indolyl)-4-triethylsilylbuta-2,3-dienoate 8j

Carried out by the general procedure above with 2-(3-(1-*tert*-butoxycarbonyl)indolyl)-2-triethylsilylethen-1-one **1j** (0.074 g, 0.2 mmol) and (1-ethoxycarbonylethylidene)triphenylphosporane (0.087 g, 0.24 mmol) in refluxing benzene (2 ml). The crude product was purified by flash chromatography (90:10 petroleum ether:diethyl ether) to yield ethyl 2-methyl-4-(3-(1-*tert*-butoxycarbonyl)indolyl)-4-triethylsilylbuta-2,3-dienoate **6j** as a colourless liquid (0.067 g, 74 %) and ethyl 2-methyl-4-(3-(1-*tert*-butoxycarbonyl)indolyl)-buta-2,3-dienoate **8j** as a colourless liquid (0.013 g, 19 %).

6j: ν_{max} (KBr/film) 2954 m, 2875 w, 1918 m (C=C=C), 1735 s (C=O Boc), 1706 s (C=O ester), 1452 m, 1376 s, 1251 s, 1157 s, 1118 m, 1093 m, 1022 w, 858 w, 763 w, 746 w cm^{-1}; δ_H (300 MHz, CDCl$_3$) 0.77 (6 H, q, *J* = 8 Hz, Si$\underline{CH_2}$CH$_3$), 0.98 (9 H, t, *J* = 8 Hz, SiCH$_2\underline{CH_3}$), 1.25 (3 H, t, *J* = 7 Hz, CH$_2\underline{CH_3}$), 1.67 (9 H, s, C($\underline{CH_3}$)), 1.96 (3 H, s, CH$_3$), 4.22 (1 H, dq, *J* = 11 Hz, *J* = 7 Hz, $\underline{CH_2}$CH$_3$), 4.28 (1 H, dq, *J* = 11 Hz, *J* = 7 Hz, $\underline{CH_2}$CH$_3$), 7.22 (1 H, appar t, *J* = 8 Hz, *Ind*H), 7.32 (1 H, appar t, *J* = 8 Hz, *Ind*H), 7.52 (1 H, s, $H_{2'}$), 7.83 (1 H, d, *J* = 8 Hz, *Ind*H), 8.08 (1 H, br d, *Ind*H); δ_C (75 MHz, CDCl$_3$) 3.5 Si$\underline{CH_2}$CH$_3$, 7.2 SiCH$_2\underline{CH_3}$, 14.3, 15.0 CH$_3$, CH$_2\underline{CH_3}$, 27.9 C($\underline{C}$H$_3$), 60.7 $\underline{CH_2}$CH$_3$, 83.9 $\underline{C}$(CH$_3$), 90.7, 90.9 $C_{2,4}$, 115.0, 120.5, 122.8, 122.9, 124.7 $C_{2',4',5',6',7'}$, 114.2, 130.0, 135.1 $C_{3',3a',7a'}$, 149.7 C=O, Boc, 169.3 C$_1$, 212.3 C$_3$; *m/z* (CI+, NH$_3$) 473 ([M+NH$_4$]$^+$, 25), 456 ([M+H]$^+$, 100), 399 (24), 356 (18), 218 (20); HRMS (CI+) Found [M+H]$^+$ 456.2567 $C_{26}H_{38}NO_4Si$, requires 456.2570

8j: ν_{max} (KBr/film) 2979 w, 2933 w, 1945 w (C=C=C), 1735 s (C=O Boc), 1710 s (C=O ester), 1452 m, 1371 m, 1348 s, 1303 w, 1253 s, 1157 s, 1118 m, 1089 s, 1020 w, 763 w, 748 w cm^{-1}; δ_H (300 MHz, $CDCl_3$) 1.21 (3 H, t, *J* = 7 Hz, $CH_2\underline{CH_3}$), 1.65 (9 H, s, $C(\underline{CH_3})$), 2.04 (3 H, d, J_{CH3-4} = 3 Hz, CH_3), 4.20 (2 H, q, *J* = 7 Hz, $\underline{CH_2}CH_3$), 6.64 (1 H, q, J_{4-CH3} = 3 Hz, H_4), 7.23 (1 H, appar t, *J* = 8 Hz, *Ind*H), 7.33 (1 H, appar t, *J* = 8 Hz, *Ind*H), 7.57 (1 H, s, $H_{2'}$), 7.68 (1 H, d, *J* = 8 Hz, *Ind*H), 8.13 (1 H, br d, *Ind*H); δ_C (75 MHz, $CDCl_3$) 14.3, 15.7 CH_3, $CH_2\underline{CH_3}$, 28.2 $C(\underline{C}H_3)$, 61.2 $\underline{CH_2}CH_3$, 84.0 $\underline{C}(CH_3)$, 88.5 C_4, 98.6 C_2, 115.3, 119.9, 123.0, 124.3, 124.9 $C_{2',4',5',6',7'}$, 112.3, 128.6, 135.9 $C_{3',3a',7a'}$, 149.4 (C=O, Boc), 167.2 C_1, 213.1 C_3; *m/z* (CI+, NH_3) 359 ($[M+NH_4]$+, 25), 342 ($[M+H]^+$, 100), 242 (77), 218 (88), 120 (24); HRMS (CI+) Found $[M+H]^+$ 342.1704 $C_{20}H_{24}NO_4$, requires 342.1705

4-Triethylsilylpenta-2,3-dienylnitrile 14a

Carried out by the general procedure above with 2-triethylsilylpropen-2-one **1a** (0.102 g, 0.60 mmol) and (cyanomethylene)triphenylphosporane (**9**, 0.2 g, 0.66 mmol) in DCM (2 ml) at rt. The crude product was purified by flash chromatography (95:5 petroleum ether:diethyl ether) to yield 4-triethylsilylpenta-2,3-dienylnitrile **14a** as a colourless liquid (0.061 g, 53 %).

ν_{max} (KBr/film) 3012 w, 2956 s, 2912 m, 2877 s, 2218 s (CN), 1934 s (C=C=C), 1457 w, 1414 w, 1238 w, 1004 m, 739 s, 721 s, 710 s cm^{-1}; δ_H (300 MHz, $CDCl_3$) 0.64 (6 H, q, *J* = 8 Hz, $Si\underline{CH_2}CH_3$), 0.93 (9 H, t, *J* = 8 Hz, $SiCH_2\underline{CH_3}$), 1.76 (3 H, d, J_{5-2} = 3 Hz, H_5), 4.74 (1 H, q, J_{2-5} = 3 Hz, H_2); δ_C (75 MHz, $CDCl_3$) 2.5 $SiCH_2\underline{CH_3}$, 7.0 $Si\underline{CH_2}CH_3$, 14.3 C_5, 59.3 C_2, 94.2 C_4, 115.2 CN, 210.5 C_3; *m/z* (CI+, NH_3) 211 ($[M+NH_4]^+$, 100); HRMS (CI+) Found $[M+NH_4]^+$ 211.1627 $C_{11}H_{23}N_2Si$, requires 211.1630

4-Triethylsilylundeca-2,3-dienenitrile 14b

Carried out by the general procedure above with 2-triethylsilylnon-1-en-1-one **1b** (0.050 g, 0.2 mmol) and (cyanomethylene)triphenylphosporane (**9**, 0.066 g, 0.22 mmol) in DCM (2 ml) at rt. The crude product was purified by flash chromatography (95:5 petroleum ether:diethyl

ether) to yield 4-triethylsilylundeca-2,3-diene nitrile **14b** as a colourless liquid (0.052 g, 94 %).

ν_{max} (KBr/film) 2955 s, 2929 s, 2874 s, 2857 s, 2217 w (CN), 1931 w (C=C=C), 1728 m, 1650 w, 1465 w, 1244 w, 1017 w, 737 m, 721 m cm^{-1}; δ_H (300 MHz, $CDCl_3$) 0.65 (6 H, q, *J* = 8 Hz, Si<u>CH$_2$</u>CH$_3$), 0.86 (3H, t, *J* = 7 Hz, H_{11}), 0.94 (9 H, t, *J* = 8 Hz, SiCH$_2$<u>CH$_3$</u>) , 1.16-1.30 (8 H, m, $H_{7,8,9,10}$), 1.46 (2 H, p, *J* = 7.5 Hz, H_6), 2.01 (2 H, m, H_5), 4.80 (1 H, t, $J_{2\text{-}5}$ = 3.5 Hz, H_2); δ_C (75 MHz, $CDCl_3$) 2.8 Si<u>CH$_2$</u>CH$_3$, 7.0 SiCH$_2$<u>CH$_3$</u>, 14.0 C_{11}, 22.6 C_{10}, 28.5, 28.7, 29.0, 29.2 $C_{6,7,8,9}$, 31.7 C_5, 60.8 C_2, 99.8 C_4, 115.4 C_1, 210.2 C_3; *m/z* (CI+, NH_3) 295 ($[M+NH_4]^+$, 100), 131 (28), 103 (27); HRMS (CI+) Found $[M+ NH_4]^+$ 295.2565 $C_{17}H_{35}N_2Si$, requires 295.2569

5-Triethylsilylhexa-3,4-dien-2-one 15a

Carried out by the general procedure above with 2-triethylsilylpropen-2-one **1a** (0.102 g, 0.6 mmol) and acetylmethylenetriphenylphosporane (**10**, 0.21 g, 0.66 mmol) in DCM (2 ml) at rt. The crude product was purified by flash chromatography (90:10 petroleum ether:diethyl ether) to yield 5-triethylsilylhexa-3,4-dien-2-one **15a** as a colourless liquid (0.065 g, 52 %).

ν_{max} (KBr/film) 2955 s, 2912 m, 2876 s, 1919 s (C=C=C), 1677 s (C=O), 1456 w, 1415 w, 1356 w, 1231 s, 1007 m, 939 w, 795 w, 737 m, 722 m, 711 m cm^{-1}; δ_H (270 MHz, $CDCl_3$) 0.64 (6 H, q, *J* = 8 Hz, Si<u>CH$_2$</u>CH$_3$), 0.93 (9 H, t, *J* = 8 Hz, SiCH$_2$<u>CH$_3$</u>), 1.80 (3 H, d, $J_{6\text{-}3}$ = 3 Hz, H_6), 2.15 (3 H, s, H_1), 5.38 (1 H, q, $J_{3\text{-}6}$ = 3 Hz, H_3); δ_C (75 MHz, $CDCl_3$) 2.7 Si<u>CH$_2$</u>CH$_3$, 7.1 SiCH$_2$<u>CH$_3$</u>, 14.6 CH_3, 26.3 C_1, 92.0 C_3, 93.0 C_5, 200.4 C=O, 210.5 C_4; *m/z* (CI+, NH_3) 228 ($[M+NH_4]^+$, 17), 211 ($[M+H]^+$, 100); HRMS (CI+) Found $[M+H]^+$ 211.1526 $C_{12}H_{23}OSi$, requires 211.1518

1-Phenyl-4-triethylsilylpenta-2,3-dien-1-one 16a

Carried out by the general procedure above with 2-triethylsilylpropen-2-one **1a** (0.073 g, 0.43 mmol) and benzoylmethylenetriphenylphosporane (**11**, 0.18 g, 0.47 mmol) in refluxing DCM (2 ml). The crude product was purified by flash chromatography (90:10 petroleum

ether:diethyl ether) to yield 1-phenyl-4-triethylsilylpenta-2,3-dien-1-one **16a** as a colourless liquid (0.073 g, 62 %).

ν_{max} (KBr/film) 2956 s, 2911 m, 2876 m, 1918 s (C=C=C), 1647 s (C=O), 1447 w, 1389 w, 1275 m, 1219 m, 1062 w, 1007 m, 710 m cm^{-1}; δ_H (270 MHz, $CDCl_3$) 0.54 (6 H, q, J = 8 Hz, Si$\underline{CH_2}$$CH_3$), 0.81 (9 H, t, J = 8 Hz, Si$CH_2$$\underline{CH_3}$), 1.83 (3 H, d, $J_{5\text{-}2}$ = 3 Hz, H_5), 5.97 (1 H, q, $J_{2\text{-}5}$ = 3 Hz, H_3), 7.35-7.51 (3 H, m, $H_{3',4'}$), 7.75-7.79 (2 H, m, $H_{2'}$); δ_C (75 MHz, $CDCl_3$) 2.6 Si$\underline{CH_2}$$CH_3$, 6.9 Si$CH_2$$\underline{CH_3}$, 14.4 C_5, 88.2 C_2, 91.6 C_4, 128.0, 128.1 $C_{2',3'}$, 131.9 $C_{4'}$, 138.5 $C_{1'}$, 194.4 C=O, 210.8 C_3; *m/z* (CI+, NH_3) 273 ([M+H]$^+$, 100); HRMS (CI+) Found [M+H]$^+$ 273.1667 $C_{17}H_{25}OSi$, requires 273.1675

1-Phenyl-4-triethylsilylundeca-2,3-dien-1-one 16b

Carried out by the general procedure above with 2-triethylsilylnon-1-en-1-one **1b** (0.382 g, 1.5 mmol) and benzoylmethylenetriphenylphosporane (**11**, 0.628 g, 1.65 mmol) in refluxing DCM (5 ml). The crude product was purified by flash chromatography (90:10 petroleum ether:diethyl ether) to yield 1-phenyl-4-triethylsilylundeca-2,3-dien-1-one **16b** as a colourless liquid (0.333 g, 62 %).

ν_{max} (KBr/film) 2954 s, 2927 s, 2875 m, 1914 s (C=C=C), 1644 s (C=O), 1390 w, 1276 m, 1218 m, 1004 m, 971 w, 842 w, 721 m, 696 m cm^{-1}; δ_H (300 MHz, $CDCl_3$) 0.57 (6 H, q, J = 8 Hz, Si$\underline{CH_2}$$CH_3$), 0.84 (12 H, t, J = 8 Hz, Si$CH_2$$\underline{CH_3}$, H_{11}), 1.22-1.32 (8 H, m, $H_{7,8,9,10}$), 1.52 (2 H, p, J = 7.5 Hz, H_6), 1.94-2.18 (2 H, m, H_5), 6.10 (1 H, t, $J_{2\text{-}5}$ = 3.5 Hz, H_2), 7.35-7.47 (3 H, m, $H_{3',4'}$), 7.79-7.82 (2 H, m, $H_{2'}$); δ_C (75 MHz, $CDCl_3$) 2.9 Si$\underline{CH_2}$$CH_3$, 7.0 Si$CH_2$$\underline{CH_3}$, 14.1 C_{11}, 22.6 C_{10}, 28.9, 29.0, 29.1, 29.3 $C_{6,7,8,9}$, 31.7 C_5, 89.5 C_2, 97.0 C_4, 128.1 $C_{2',3'}$ (2 peaks overlapped), 131.9 $C_{4'}$, 138.6 $C_{1'}$, 193.6 C_1, 210.0 C_3; *m/z* (CI+, NH_3) 357 ([M+H]$^+$, 100); HRMS (CI+) Found [M+H]$^+$ 357.2614 $C_{23}H_{37}OSi$, requires 357.2614

General procedure for the preparation of allenylsilanes from silyl ketenes and semi-stabilised phosphorus ylides

3-Methyl-1-phenyl-3-triethylsilylbuta-1,2-diene 17a

To a suspension of benzyltriphenylphosphonium chloride (0.238 g, 0.55 mmol) in THF (2 ml) at rt was added dropwise *n*-BuLi (0.275 ml of a 2 M solution in pentane, 0.55 mmol). The resulting red-orange solution was stirred at rt for 0.5 h and then a solution of 2-triethylsilylpropen-2-one **1a** (0.085 g, 0.5 mmol) in THF (2ml) was added dropwise. After 30 min, hexane (10 ml) was then added and the white precipitate removed by filtration. The filtrate was concentrated under reduced pressure and the residual oil purified by flash chromatography (100 % petroleum ether) to yield 3-methyl-1-phenyl-3-triethylsilylbuta-1,2-diene **17a** as a colourless liquid (0.079 g, 65 %).

ν_{max} (KBr/film) 3029 w, 2953 s, 2911 m, 2874 s, 1923 s (C=C=C), 1598 w, 1496 w, 1456 m, 1414 w, 1238 w, 1009 m, 931 w, 797 m, 737 s, 711 s, 692 s cm^{-1}; δ_H (270 MHz, $CDCl_3$) 0.70 (6 H, q, *J* = 8 Hz, Si$\underline{CH_2}CH_3$), 1.02 (9 H, t, *J* = 8 Hz, $SiCH_2\underline{CH_3}$), 1.85 (3 H, d, $J_{4\text{-}1}$ = 3 Hz, H_4), 5.84 (1 H, q, $J_{1\text{-}4}$ = 3 Hz, H_1), 7.12-7.18 (1 H, m, $H_{4'}$), 7.24-7.33 (4 H, m, $H_{2',3'}$); δ_C (75 MHz, $CDCl_3$) 3.0 Si$\underline{CH_2}CH_3$, 7.4 $SiCH_2\underline{CH_3}$, 15.8 C_4, 88.0 C_3, 92.6 C_1, 125.7 $C_{4'}$, 125.9, 128.4 $C_{2',3'}$, 136.1 $C_{1'}$, 206.4 C_2; *m/z* (CI+, NH_3) 245 ($[M+H]^+$, 41), 132 ($[SiEt_3+NH_3]^+$, 100); HRMS (CI+) Found $[M+H]^+$ 245.1717 $C_{16}H_{25}Si$, requires 245.1725

1-Phenyl-3-triethylsilyldeca-1,2-diene 17b

Carried out by the general procedure above with 2-triethylsilylnon-1-en-1-one **1b** (0.509 g, 2 mmol), benzyltriphenylphosphonium chloride (0.953 g, 2.2 mmol) in THF (15 ml) and *n*-BuLi (1.15 ml of a 2 M solution in pentane, 2.3 mmol). The crude product was purified by flash chromatography (100 % petroleum ether) to yield 1-phenyl-3-triethylsilyldeca-1,2-diene **17b** as a colourless liquid (0.445 g, 68 %).

ν_{max} (KBr/film) 3028 w, 2953 s, 2927 s, 2873 m, 2854 m, 1918 s (C=C=C), 1599 w, 1495 w, 1456 m, 1414 w, 1377 w, 1005 m, 797 m, 735 s, 718 s, 692 s cm^{-1}; δ_H (300 MHz, $CDCl_3$) 0.61 (6 H, q, *J* = 8 Hz, Si$\underline{CH_2}CH_3$), 0.82 (3 H, t, $J_{10\text{-}9}$ = 7 Hz, H_{10}), 0.93 (9 H, t, *J* = 8 Hz, $SiCH_2\underline{CH_3}$), 1.19-1.31 (8 H, m, $H_{6,7,8,9}$), 1.49 (2 H, p, *J* = 7.5 Hz, H_5), 1.99-2.05 (2 H, m, H_4), 5.83 (1 H, t, $J_{1\text{-}4}$ = 3.5 Hz, H_1), 7.10 (1 H, m, $H_{4'}$), 7.19-7.27 (4 H, m, $H_{2',3'}$); δ_C (75 MHz, $CDCl_3$) 3.2 Si$\underline{CH_2}CH_3$, 7.4 $SiCH_2\underline{CH_3}$, 14.1 C_{10}, 22.6, 27.0, 29.2, 29.5, 29.7 $C_{5,6,7,8}$, 31.9 C_4,

89.7 C_1, 98.3 C_3, 125.6 $C_{4'}$, 125.8, 128.4 $C_{2',3'}$, 136.4 $C_{1'}$, 205.8 C_2; *m/z* (CI+, NH_3) 358 ($[M+NH_4]^+$, 10), 329 ($[M+H]^+$, 31), 132 ($[SiEt_3+NH_3]^+$, 100); HRMS (CI+) Found $[M+H]^+$ 329.2657 $C_{22}H_{37}Si$, requires 329.2665

3-(4-Bromophenyl)-1-phenyl-3-triethylsilylpropa-1,2-diene 18g

Carried out by the general procedure above with 2-triethylsilylnon-1-en-1-one **1g** (0.109 g, 0.35 mmol), benzyltriphenylphosphonium chloride (0.167 g, 0.385 mmol) in THF (2 ml) and *n*-BuLi (0.16 ml of a 2.5 M solution in pentane, 0.402 mmol). The crude product was purified by flash chromatography (100 % petroleum ether) to yield 3-(4-bromophenyl)-1-phenyl-3-triethylsilylpropa-1,2-diene **18g** as a colourless liquid (0.078 g, 58%).

ν_{max} (KBr/film) 3027 w, 2954 s, 2910 s, 2873 s, 1913 s (C=C=C), 1598 w, 1483 s, 1456 m, 1413 w, 1375 w, 1238 w, 1072 m, 1008 s, 896 m, 825 m, 802 m, 734 s, 719r s cm^{-1}; δ_H (300 MHz, $CDCl_3$) 0.83 (6 H, q, *J* = 8 Hz, Si$\underline{CH_2}$$CH_3$), 1.00 (9 H, t, *J* = 8 Hz, $SiCH_2$$\underline{CH_3}$), 6.24 (1 H, s, H_1), 7.25 (3 H, m, inc. d, *J* = 8.5 Hz, $H_{2''}$, $H_{4'}$), 7.33 (4 H, m, $H_{2',3'}$), 7.45 (2H, d, *J* = 8.5 Hz, $H_{3''}$); δ_C (75 MHz, $CDCl_3$) 4.0 Si$\underline{CH_2}$$CH_3$, 7.5 $SiCH_2$$\underline{CH_3}$, 91.5 C_1, 101.0, 120.5, 126.3, 126.6, 128.8, 129.3, 131.7, 134.4, 136.63 (8 x ArC), 210.4 C_2; *m/z* (CI+, NH_3) 387 ($[^{81}BrM+H]^+$, 35), 385 ($[^{79}BrM+H]^+$, 35), 307 (37), 132 ($[SiEt_3+NH_3]^+$, 100); HRMS (CI+) Found $[M+H]^+$ 387.0967 $C_{21}H_{26}{}^{81}BrSi$, requires 387.0967

1-(4-Bromophenyl)-3-triethylsilyldeca-1,2-diene 18b

Carried out by the general procedure above with 2-triethylsilylnon-1-en-1-one **1b** (0.051 g, 0.2 mmol), (4-bromobenzyl)triphenylphosphonium chloride (0.107 g, 0.21 mmol) in THF (15 ml) and lithium hexamethyldisilazide (0.22 ml of a 1 M solution in THF, 2.2 mmol). The crude product was purified by flash chromatography (100 % petroleum ether) to yield 3-(4-bromophenyl)-1-phenyl-3-triethylsilyldeca-1,2-diene **18b** as a colourless liquid (0.043 g, 53 %).

ν_{max} (KBr/film) 2952 s, 2927 s, 2873 s, 2854 m, 1916 s (C=C=C), 1486 m, 1461 w, 1417 w, 1070 w, 1008 m, 833 m, 732 s, 719 s cm^{-1}; δ_H (270 MHz, $CDCl_3$) 0.61 (6 H, q, *J* = 8 Hz), 0.83

(3 H, t, J_{10-9} = 7 Hz), 0.93 (9 H, t, J = 8 Hz), 1.18-1.31 (8 H, m), 1.42-1.51 (2 H, m), 1.99-2.07 (2 H, m), 5.77 (1 H, t, J_{1-4} = 3.5 Hz), 7.06 (2 H, d, $J_{3'-2'}$ = 8.5 Hz), 7.35 (2 H, d, $J_{2'-3'}$ = 8.5 Hz); δ_C (67.5 MHz, $CDCl_3$) 3.2, 7.4, 14.1, 22.6, 29.1 (2 peaks overlapped), 29.5, 29.6, 31.8, 88.9, 98.9, 118.9, 127.3, 131.5, 135.5, 205.6; *m/z* (CI+, NH_3) 409 ($[M^{81}Br+H]^+$, 43), 407 ($[M^{79}Br+H]^+$, 40), 329 ($[M-Br+H]^+$, 40), 193 (69), 132 ($[SiEt_3+NH_3]^+$, 100); HRMS (CI+) Found $[M+H]^+$ 409.1756 $C_{22}H_{36}{}^{81}BrSi$, requires 409.1749

General procedure for the preparation of allenylsilanes from silylketenes and Cp_2TiMe_2

Ethyl 4-triethylsilylundeca-2,3-diene 19b

A solution of Cp_2TiMe_2 (4 ml of a 0.5M solution in THF, 2 mmol) was mixed with 2-triethylsilylnon-1-en-1-one **1b** (0.255 g, 1 mmol). The resulting solution was protected from light and stirred at 65-70 °C with exclusion of moisture and the reaction monitored by TLC for complete consumption of the silylketene. The solution was cooled to rt and diluted with petroleum ether. The resulting precipitate was removed by filtration, the filtrate concentrated under reduced pressure and the residual oil purified by flash chromatography (100 % petroleum ether) to yield 4-triethylsilylundeca-2,3-diene **19b** as a colourless liquid (0.197 g, 78 %).

ν_{max} (KBr/film) 2954 s, 2925 s, 2875 s, 2956 m, 1926 s (C=C=C), 1461 m, 1415 w, 1376 w, 1008 m, 808 m, 719 s cm^{-1}; δ_H (300 MHz, $CDCl_3$) 0.62 (6 H, q, J = 8 Hz, Si$\underline{CH_2}CH_3$), 0.84-0.97 (12 H, m, $SiCH_2\underline{CH_3}$, H_{10}), 1.17-1.24 (8 H, m, $H_{6,7,8,9}$), 1.41 (2 H, p, J = 7.5 Hz, H_5), 1.83-1.89 (2 H, m, H_4), 4.28 (2 H, t, J_{1-4} = 3.5 Hz, H_1); δ_C (75 MHz, $CDCl_3$) 3.1 Si$\underline{CH_2}CH_3$, 7.3 $SiCH_2\underline{CH_3}$, 14.1 C_{10}, 22.7, 28.9 (2 peaks overlapped), 29.0, 29.4 $C_{5,6,7,8,9}$, 31.9 C_4, 68.5 C_1, 91.6 C_3, 208.9 C_2; *m/z* (CI+, NH_3) 253 ($[M+H]^+$, 17), 132 ($[SiEt_3+NH_3]^+$, 100), 119 (40), 105 (27); HRMS (CI+) Found $[M+H]^+$ 253.2350 $C_{16}H_{33}Si$, requires 253.2352

3-*tert*-Butyldimethylsilyldeca-1,2-diene 19b*

Carried out by the general procedure above with Cp_2TiMe_2 (0.6 ml, 0.3 mmol) and 2-*tert*-butyldimethylsilylnon-2-en-1-one **1b*** (0.038 g, 0.15 mmol). The crude product was purified by flash chromatography (100 % petroleum ether) to yield 3-*tert*-butyldimethylsilyldeca-1,2-diene **19b*** as a colourless liquid (0.030 g, 79 %).

ν_{max} (KBr/film) 2956 s, 2928 s, 2857 s, 1925 s (C=C=C), 1464 w, 1250 w, 838 s, 825 s, 809 s, 771 m cm^{-1}; δ_H (270 MHz, $CDCl_3$) 0.03 (6 H; s; Si($\underline{CH}_3$)$_2$), 0.86 (3 H, t, $J_{9\text{-}8}$ = 7 Hz, H_9), 0.88 (9 H, s, SiC($\underline{CH}_3$)$_3$), 1.22-1.33 (8 H, m, $H_{6,7,8,9}$), 1.44 (2 H, p, J = 7.5 Hz, H_5), 1.86-1.94 (2 H, m, H_4), 4.31 (2 H, t, $J_{1\text{-}4}$ = 3.5 Hz, H_1); δ_C (75 MHz, $CDCl_3$) - 6.2 Si($\underline{CH}_3$)$_2$, 14.1 C_{10}, 18.8 Si$\underline{C}$(CH_3)$_3$, 22.7 C_9, 26.7 SiC($\underline{CH}_3$)$_3$, 28.9, 29.2, 29.3, 29.4 $C_{5,6,7,8}$, 31.9 C_4, 69.2 C_1, 92.6 C_3, 209.4 C_2; *m/z* (CI+, NH_3) 270 ($[M+NH_4]^+$, 10), 253 ($[M+H]^+$, 78), 214 (36), 212 (25), 137 (44), 132 ($[TBS+NH_3]^+$, 100), 92 (24), 90 (42); HRMS (CI+) Found $[M+H]^+$ 253.2356 $C_{16}H_{33}Si$, requires 253.2352

1-Phenyl-2-triethylsilylbuta-2,3-diene 19c

Carried out by the general procedure above with Cp_2TiMe_2 (3 ml, 1.5 mmol) and 3-phenyl-2-triethylsilylpropen-1-one **1c** (0.123 g, 0.5 mmol). The crude product was purified by flash chromatography (100 % petroleum ether) to yield 1-phenyl-2-triethylsilylbuta-2,3-diene **19c** as a colourless liquid (0.090 g, 75 %).

ν_{max} (KBr/film) 2952 s, 2910 m, 2875 m, 1926 s (C=C=C), 1494 w, 1454 w, 1415 w, 1238 w, 1006 m, 809 m, 755 w, 734 s, 717 s, 698 s cm^{-1}; δ_H (270 MHz, $CDCl_3$) 0.55 (6 H, q, J = 8 Hz, Si$\underline{CH_2}$$CH_3$), 0.89 (9 H, t, J = 8 Hz, $SiCH_2$$\underline{CH_3}$), 3.28 (2 H, t, $J_{1\text{-}4}$ = 3 Hz, H_1), 4.28 (2 H, t, $J_{4\text{-}1}$ = 3 Hz, H_4), 7.13-7.29 (5 H, m, Ph); δ_C (75 MHz, $CDCl_3$) 3.1 Si$\underline{CH_2}$$CH_3$, 7.2 $SiCH_2$$\underline{CH_3}$, 36.4 C_1, 68.7 C_4, 91.5 C_2, 126.0 $C_{4'}$, 128.0, 128.9 $C_{2',3'}$, 140.5 $C_{1'}$, 210.6 C_3; *m/z* (CI+, NH_3) 245 ($[M+H]^+$, 73), 132 ($[SiEt_3+NH_3]^+$, 100); HRMS (CI+) Found $[M+H]^+$ 245.1718 $C_{16}H_{25}Si$ requires 245.1725

1-Cyclohexyl-1-triethylsilylpropa-1,2-diene 19d

Carried out by the general procedure above with Cp_2TiMe_2 (12 ml, 6 mmol) and 2-cyclohexyl-2-triethylsilylethen-1-one **1d** (0.477 g, 2 mmol). The crude product was purified by flash chromatography (100 % petroleum ether) to yield 1-cyclohexyl-1-triethylsilylpropa-1,2-diene **19d** as a colourless liquid (0.39 g, 82 %).

ν_{max} (KBr/film) 2942 s, 2925 s, 2879 m, 1920 s (C=C=C), 1448 w, 1415 w, 1238 w, 1074 w, 1016 w, 808 m, 730 s, 717 s cm^{-1}; δ_H (270 MHz, $CDCl_3$) 0.59 (6 H, q, J = 8 Hz, Si$\underline{CH_2}$$CH_3$), 0.91 (9 H, t, J = 8 Hz, $SiCH_2$$\underline{CH_3}$), 1.10-1.28 (5 H, m), 1.56-1.80 (6 H, m), 4.30 (2 H, d, $J_{3-1'}$ = 1.5 Hz, H_3); δ_C (75 MHz, $CDCl_3$) 3.4 Si$\underline{CH_2}$$CH_3$, 7.4 $SiCH_2$$\underline{CH_3}$, 26.1 $C_{4'}$, 26.8, 33.9 $C_{2',3'}$, 37.9 $C_{1'}$, 69.3 C_3, 97.2 C_1, 208.9 C_2; *m/z* (CI+, NH_3) 237 ([M+H]$^+$, 52), 226 (14), 172 (14), 132 ([$SiEt_3$+NH_3]$^+$, 100); HRMS (CI+) Found [M+H]$^+$ 237.2032 $C_{15}H_{29}Si$, requires 237.2038

1-Phenyl-1-triethylsilylpropa-1,2-diene 19e

Carried out by the general procedure for the preparation of silyl allenes with Cp_2TiMe_2 (4 ml, 2 mmol) and 2-phenyl-2-triethylsilylethen-1-one **1e** (0.116 g, 0.5 mmol). The crude product was purified by flash chromatography (100 % petroleum ether) to 1-phenyl-1-triethylsilylpropa-1,2-diene **19e** as a colourless liquid (0.044 g, 38 %).

ν_{max} (KBr/film) 2954 s, 2910 m, 2875 s, 1918 s (C=C=C), 1596 w, 1490 w, 1459 w, 1415 w, 1238 w, 1004 m, 813 m, 761 m, 734 s, 721 s, 696 s cm^{-1}; δ_H (300 MHz, $CDCl_3$) 0.73 (6 H, q, J = 8 Hz, Si$\underline{CH_2}$$CH_3$), 0.95 (9 H, t, J = 8 Hz, $SiCH_2$$\underline{CH_3}$), 4.64 (2 H, s, H_3), 7.15-7.19 (1 H, m, $H_{4'}$), 7.24-7.28 (4 H, m, $H_{2',3'}$); δ_C (75 MHz, $CDCl_3$) 3.5 Si$\underline{CH_2}$$CH_3$, 7.3 $SiCH_2$$\underline{CH_3}$, 69.7 C_1, 95.6 C_3, 126.1 $C_{4'}$, 127.6, 128.4 $C_{2',3'}$, 137.6 $C_{1'}$, 211.8 C_2; *m/z* (CI+, NH_3) 231 ([M+H]$^+$, 74), 132 ([$SiEt_3$+NH_3]$^+$, 100); HRMS (CI+) Found [M+H]$^+$ 231.1564 $C_{15}H_{23}Si$, requires 231.1569
