# Supplementary material for: Synthesis of highly substituted allenylsilanes by alkylidenation of silylketenes
Source: Beilstein J Org Chem. 2005 Aug 26;1:5. doi: 10.1186/1860-5397-1-5 (PMC1399453; doi:10.1186/1860-5397-1-5)
Supplement: File 2 — NMR spectra [file Beilstein_J_Org_Chem-01-05-s002.pdf]

Synthesis of Highly Substituted Allenylsilanes by Alkylidenation of Silylketenes

Stephen P. Marsden* and Pascal C. Ducept

Supplementary information 1:

^{1}H nmr spectra of silylated diazoketones **2** and silylketenes **1**

^{1}H and ^{13}C nmr spectra of allenylsilanes **6**, **7**

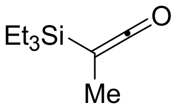

1a

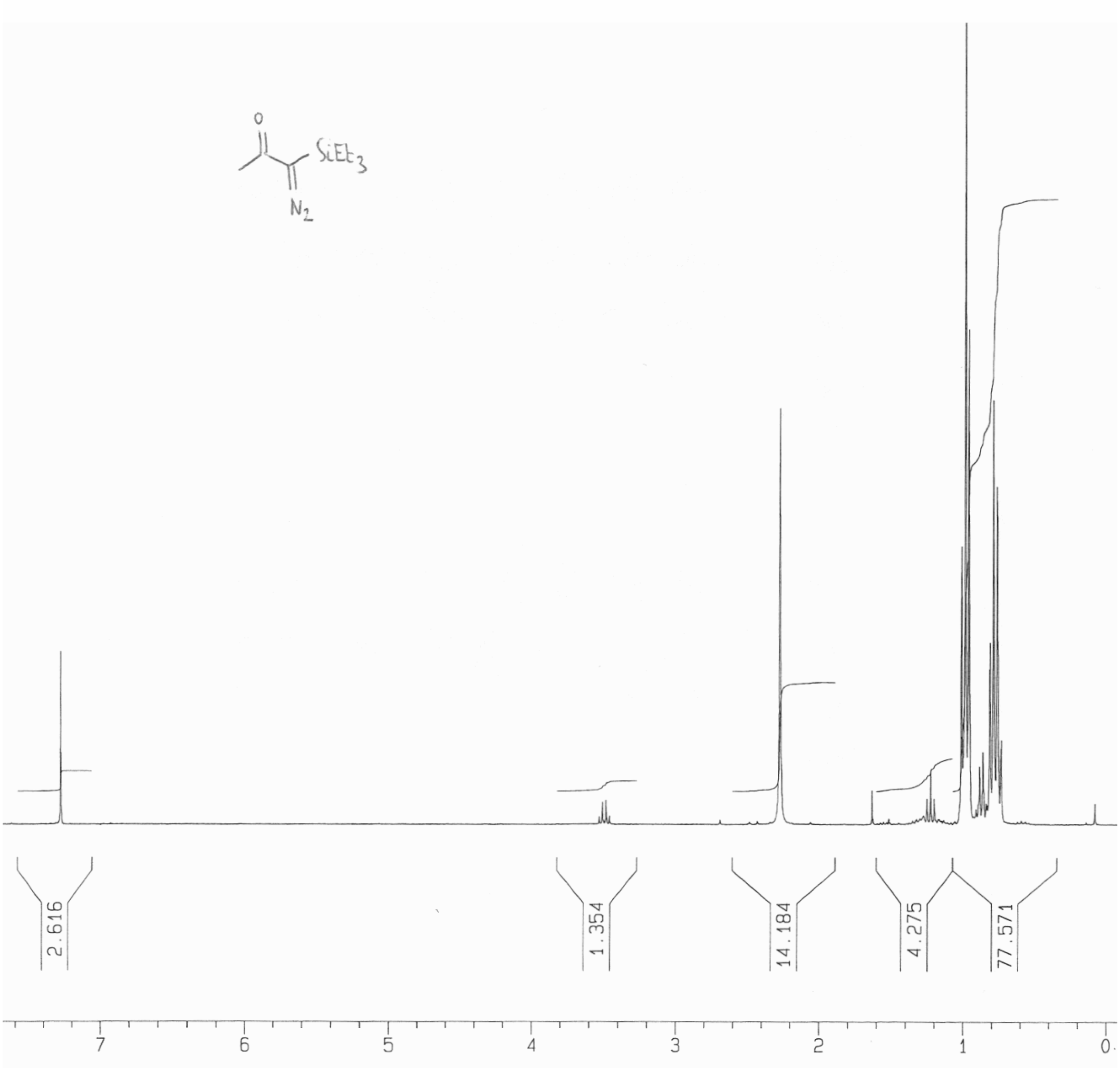

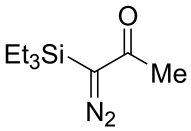

2a

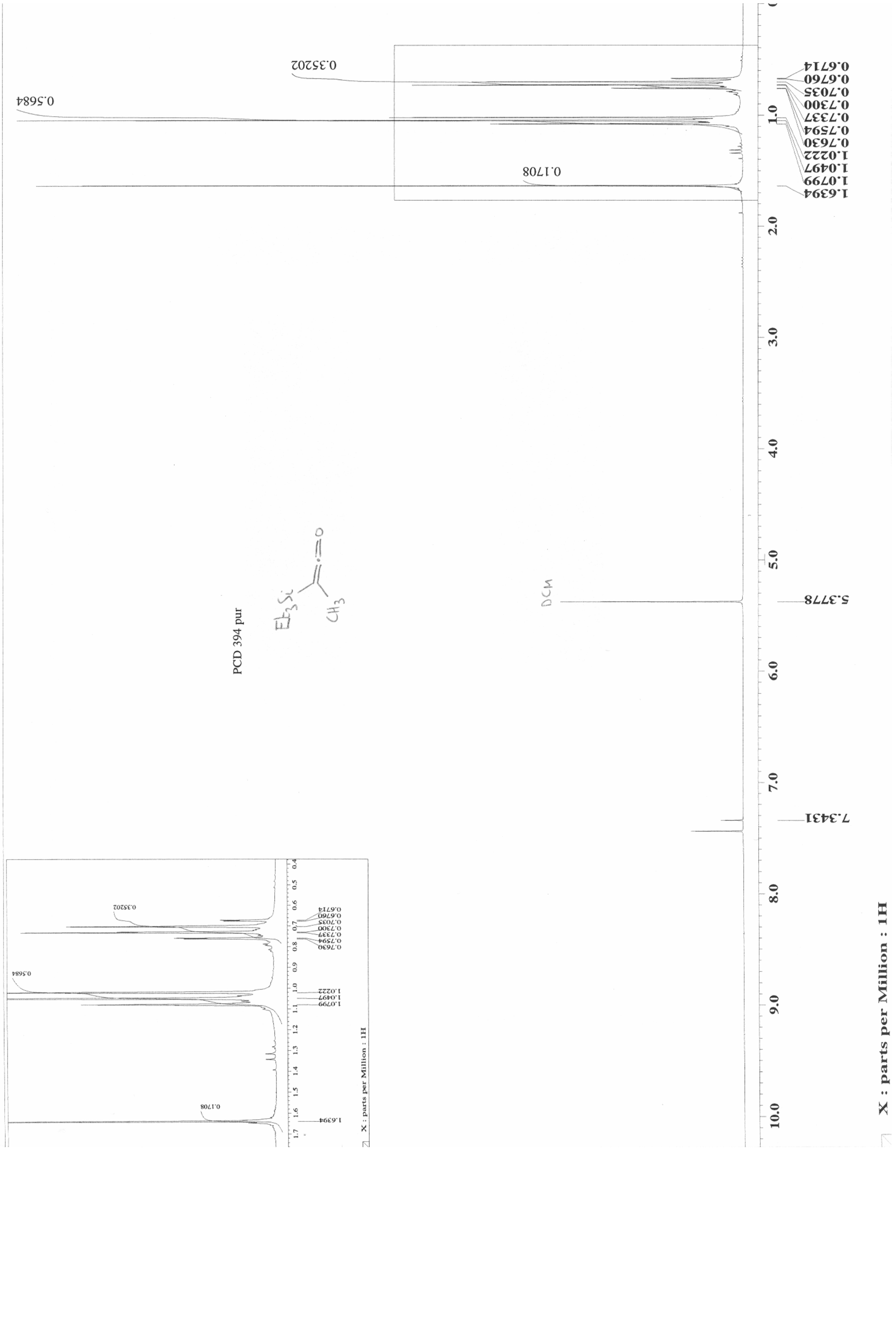
PCD 394 pur
DCM
0.35202
0.5684
0.1708
0.6714
0.6760
0.7035
0.7300
0.7337
0.7594
0.7630
1.0222
1.0497
1.0799
1.6394
5.3778
7.3431
X : parts per Million : 1H

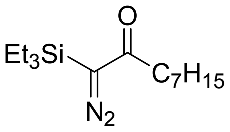
Et3Si
C7H15
N2
O

2b

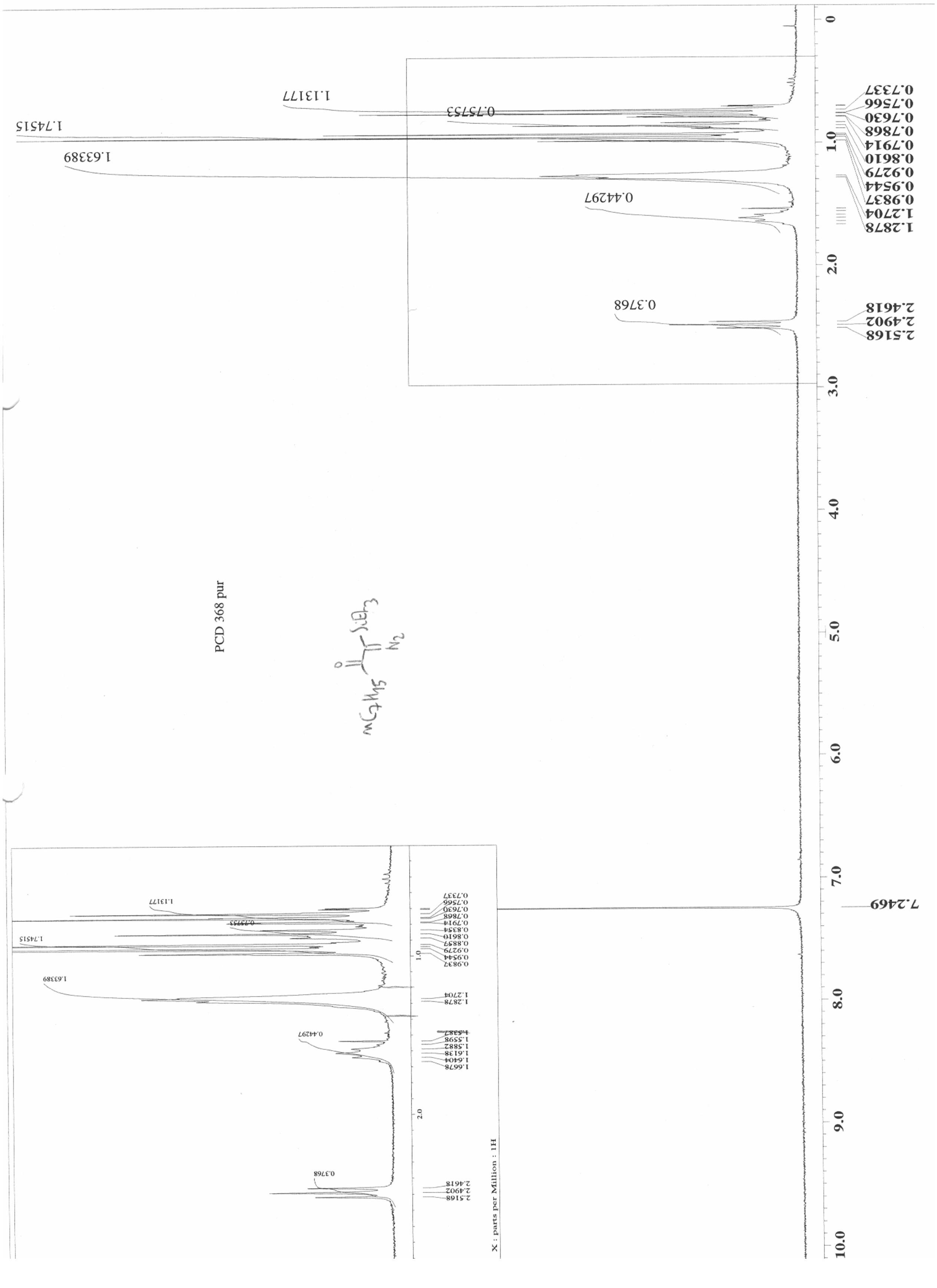
PCD 368 pur
X : parts per Million : 1H
7.2469
2.5168
2.4902
2.4618
1.2878
1.2704
0.9837
0.9544
0.9279
0.8610
0.7914
0.7868
0.7630
0.7566
0.7337
1.74515
1.63389
1.13177
0.75753
0.44297
0.3768

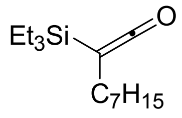

1b

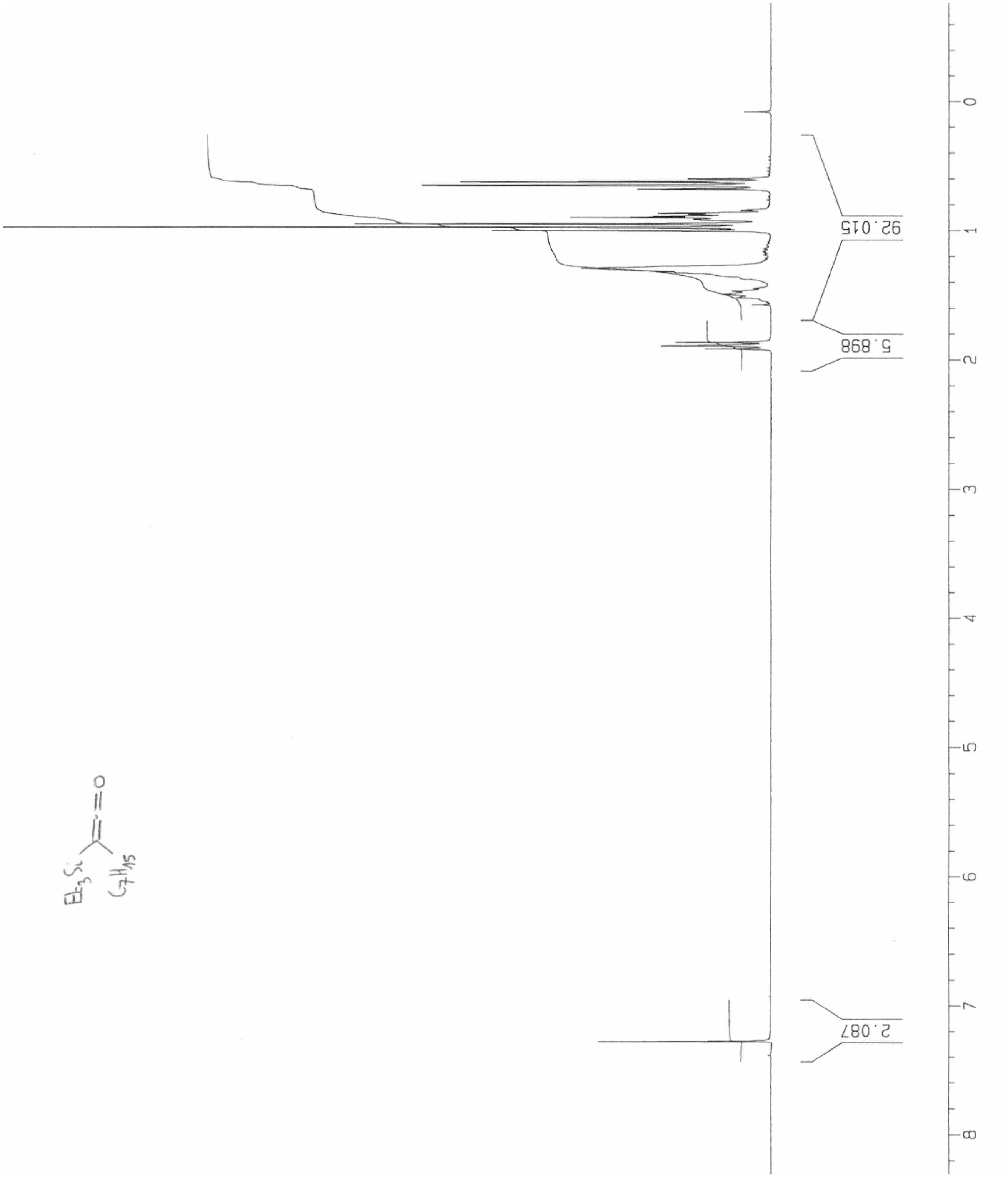

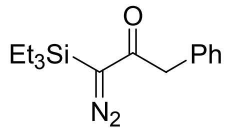

2c

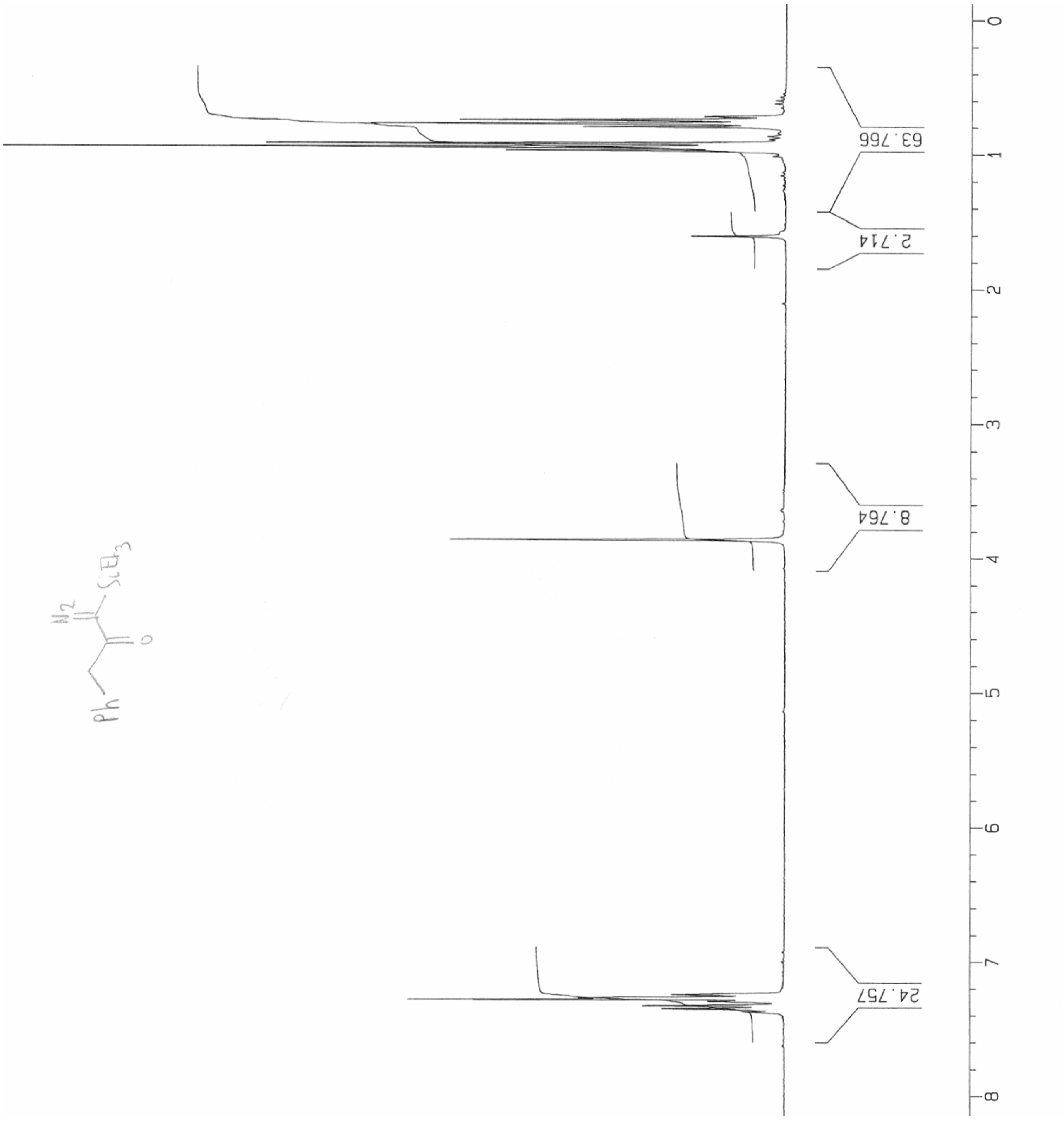

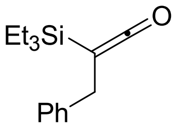
Et3Si
O
Ph

1c

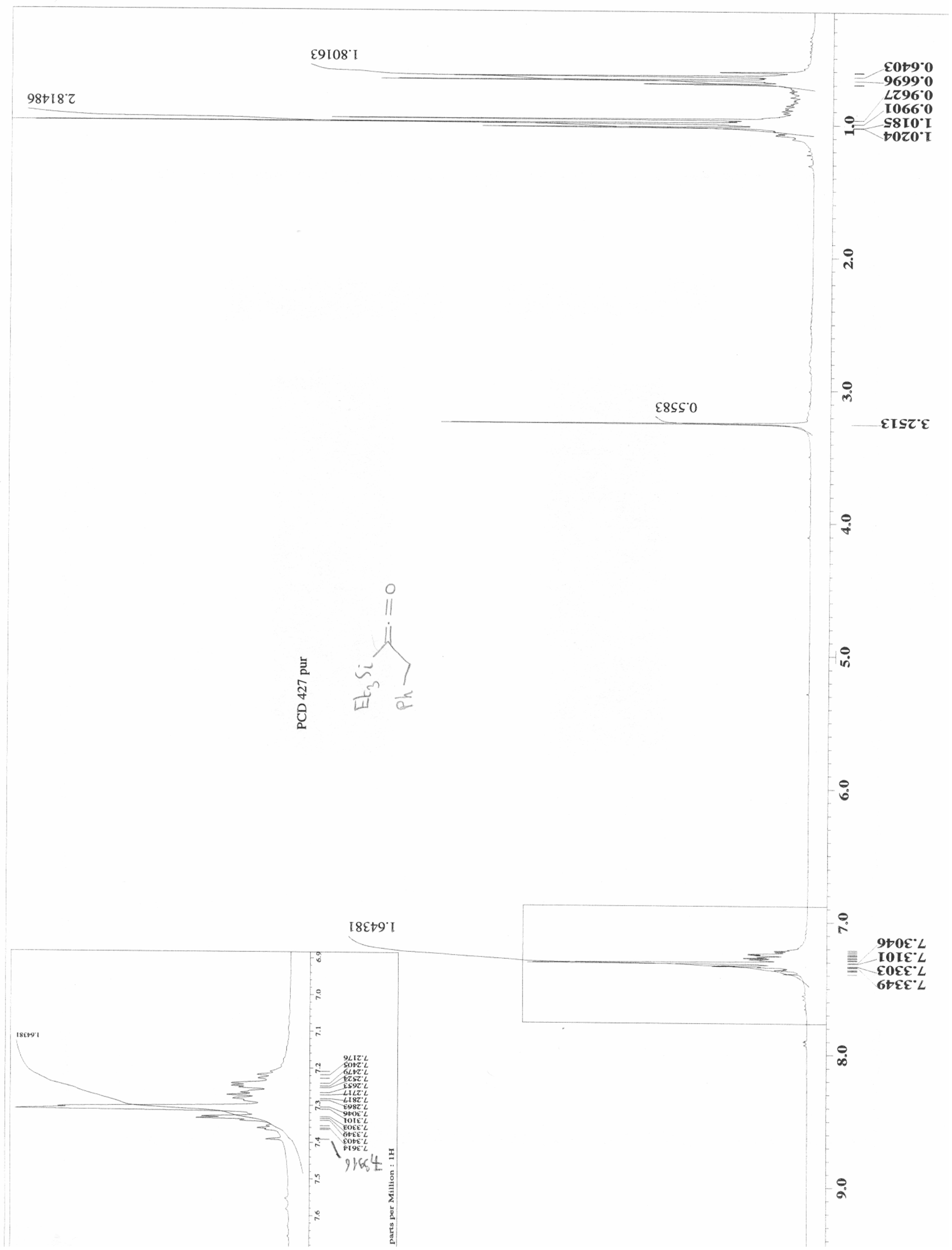
1.80163
2.81486
0.5583
1.64381
0.6403
0.6696
0.9627
0.9901
1.0185
1.0204
3.2513
7.3046
7.3101
7.3303
7.3349
1.0
2.0
3.0
4.0
5.0
6.0
7.0
8.0
9.0
PCD 427 pur
7.2176
7.2405
7.2479
7.2524
7.2653
7.2717
7.2817
7.2863
7.3046
7.3101
7.3303
7.3349
7.3403
7.3614
parts per Million : 1H

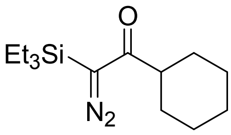

2d

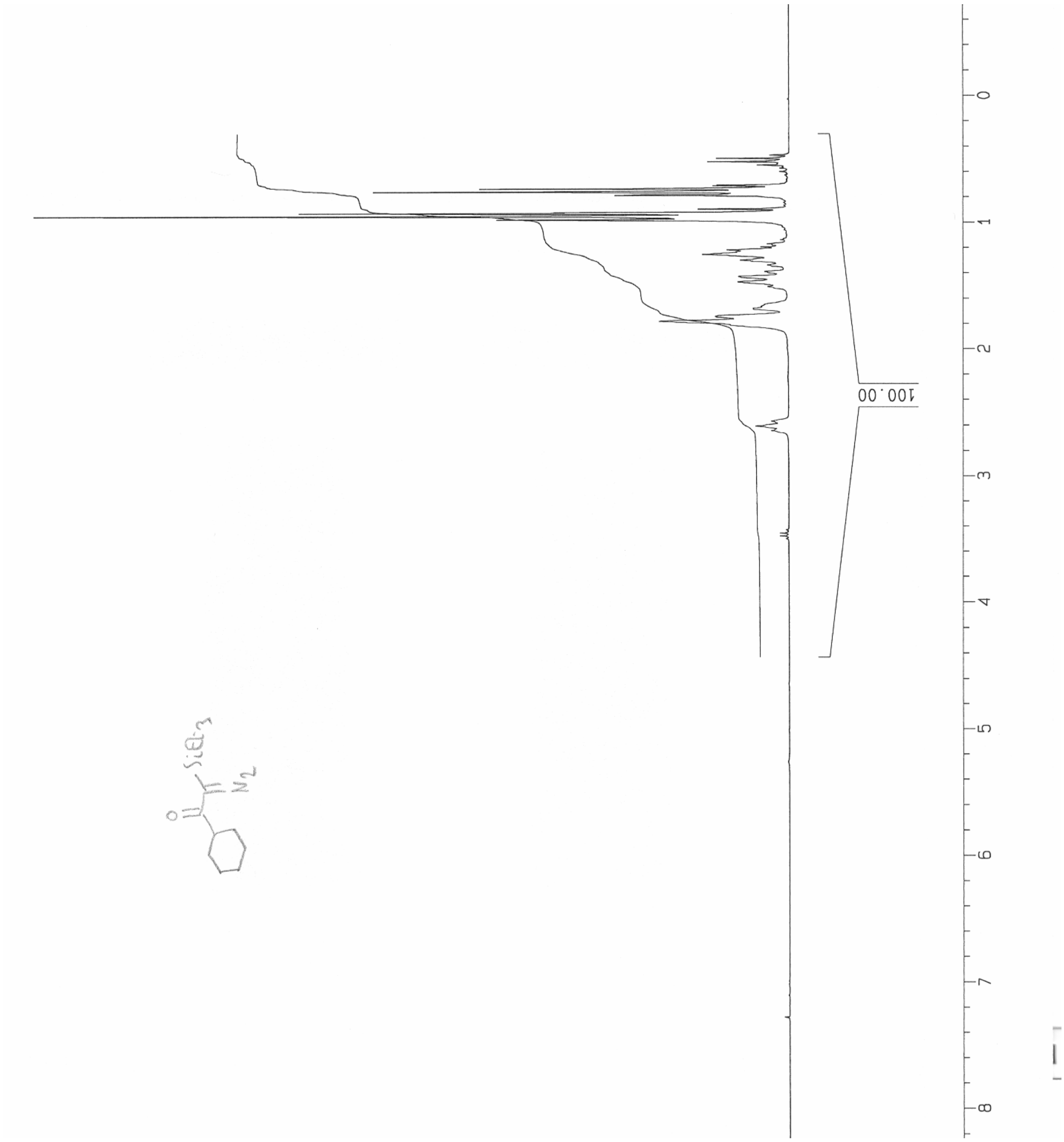

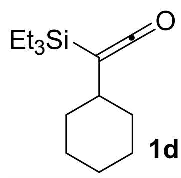

1d

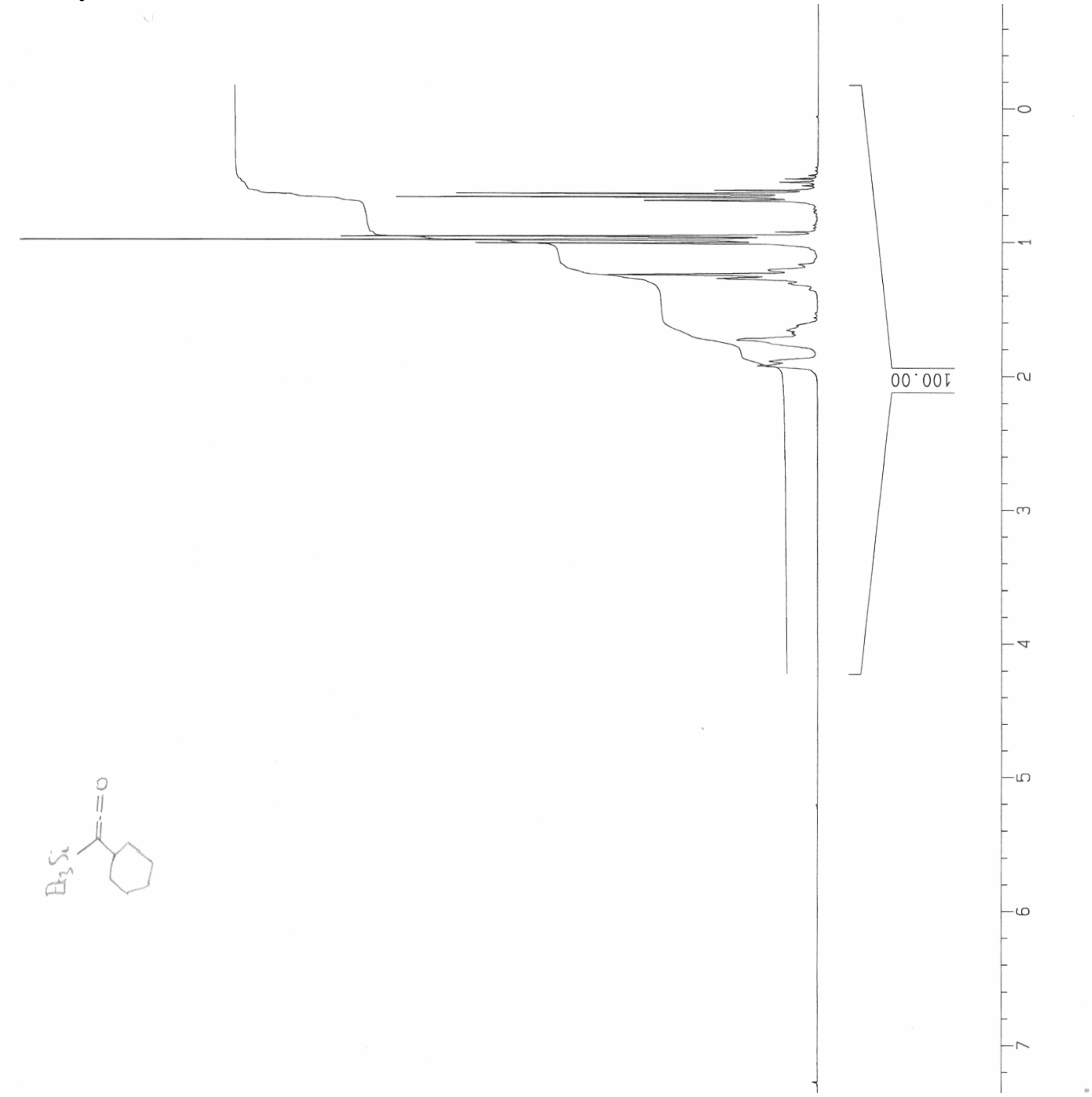

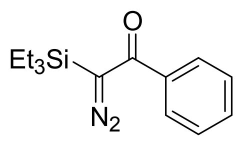

2e

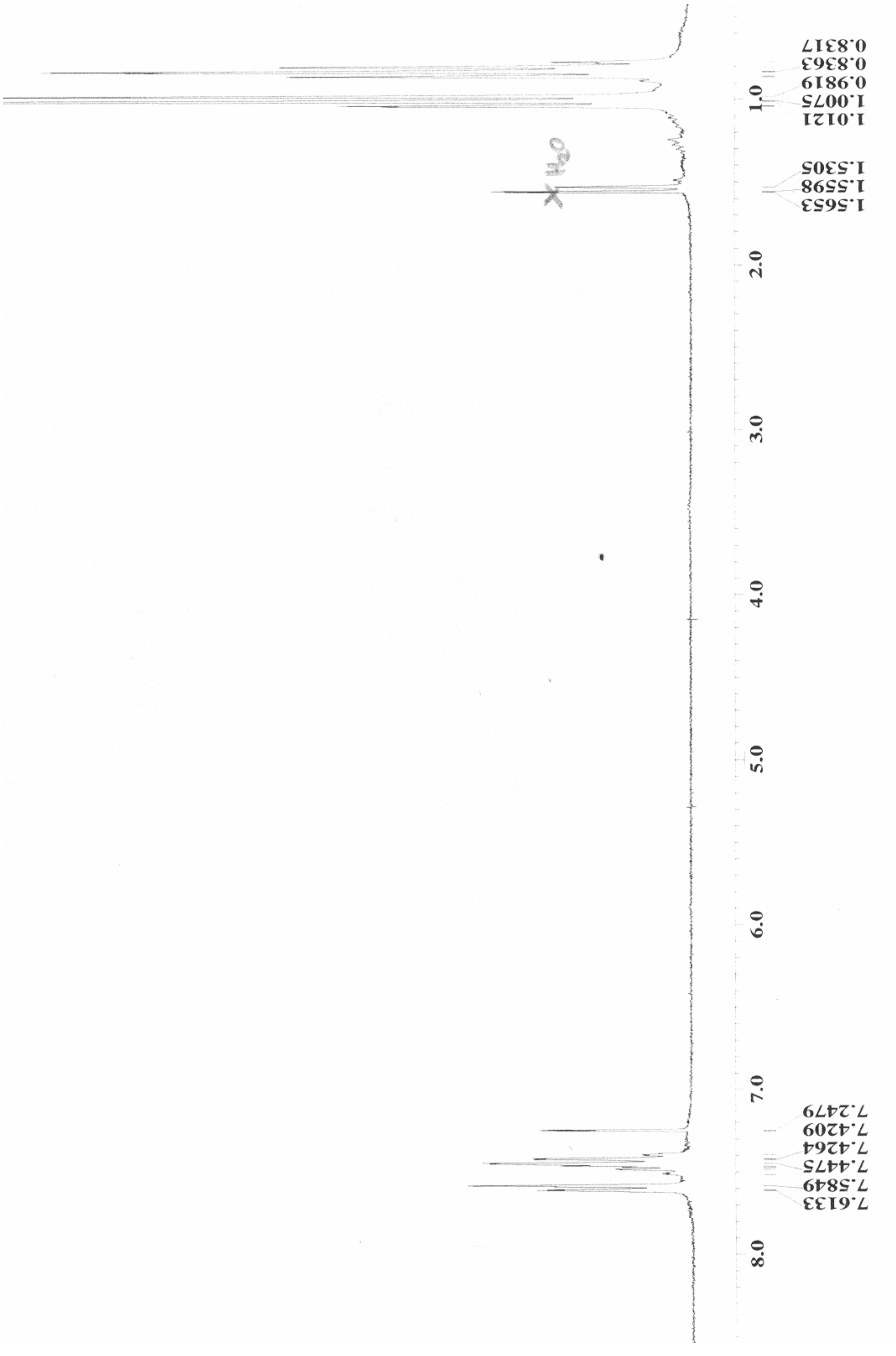

0.8317
0.8363
0.9819
1.0075
1.0121
1.5305
1.5598
1.5653
7.2479
7.4209
7.4264
7.4475
7.5849
7.6133
1.0
2.0
3.0
4.0
5.0
6.0
7.0
8.0
H2O

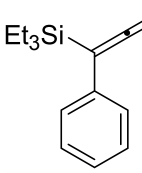

1e

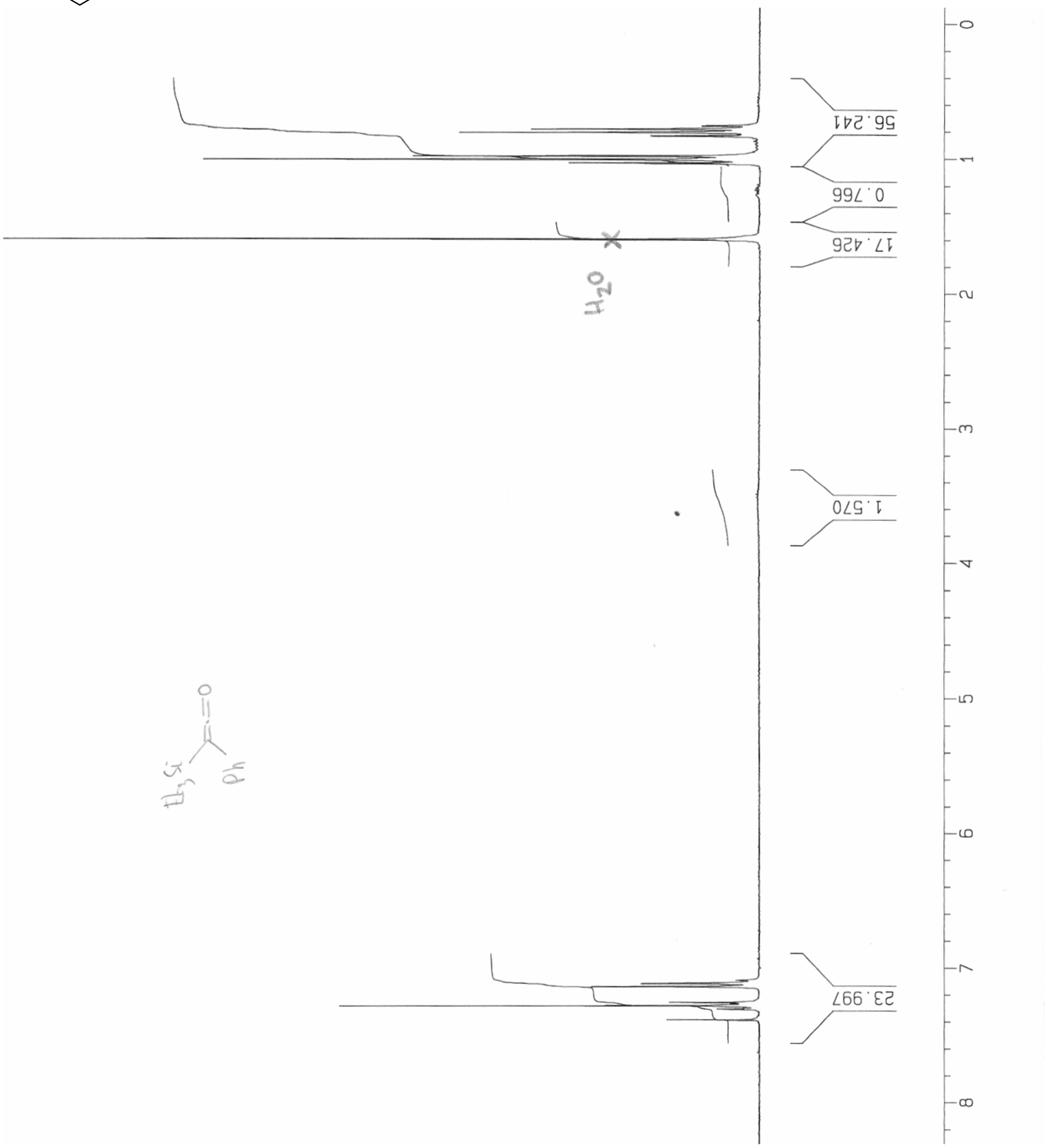

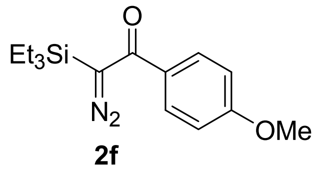

2f

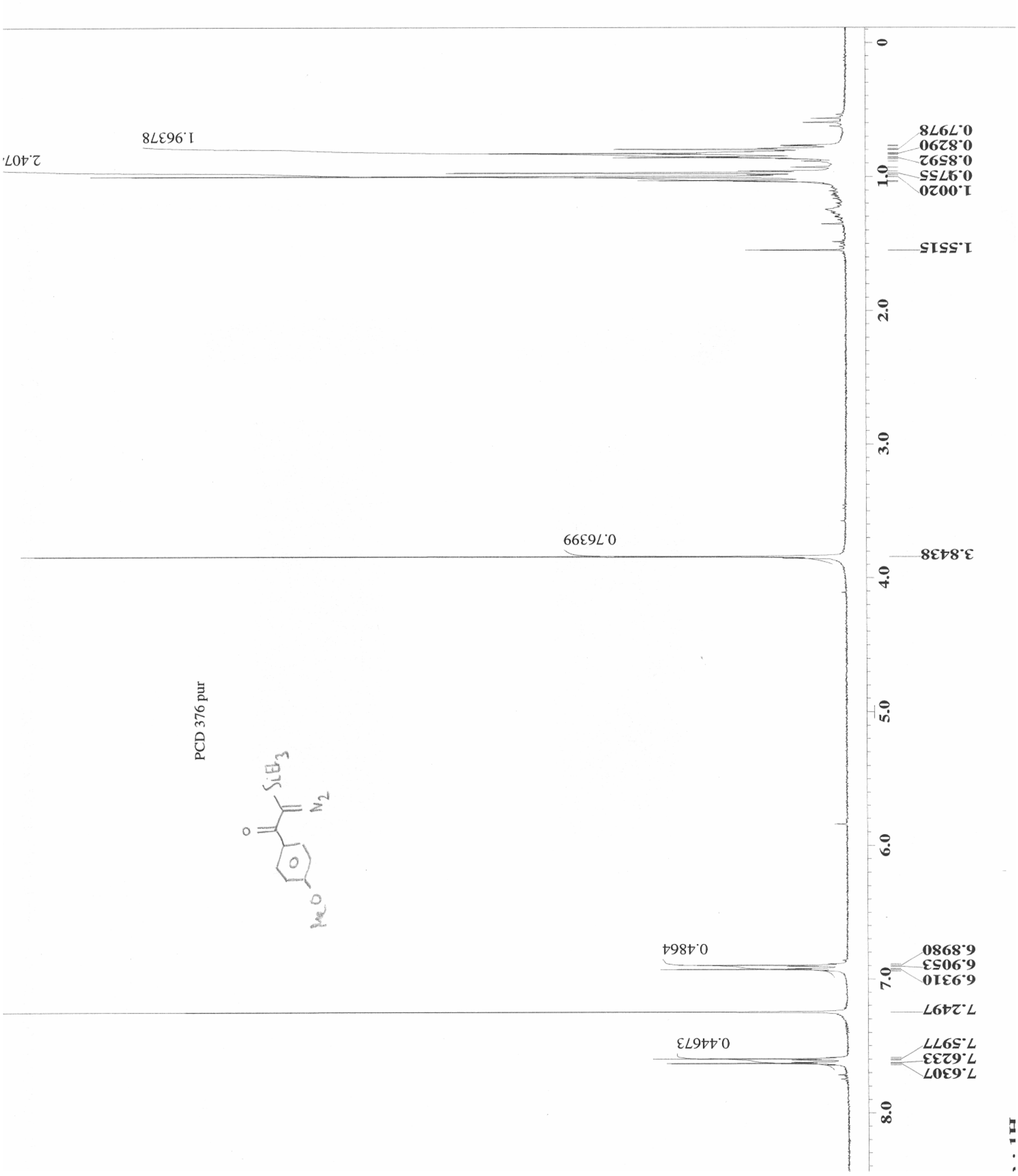
PCD 376 pur
0.7978
0.8290
0.8592
0.9755
1.0020
1.5515
3.8438
6.8980
6.9053
6.9310
7.2497
7.5977
7.6233
7.6307
1.96378
0.76399
0.4864
0.44673

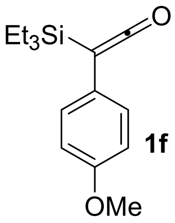

1f

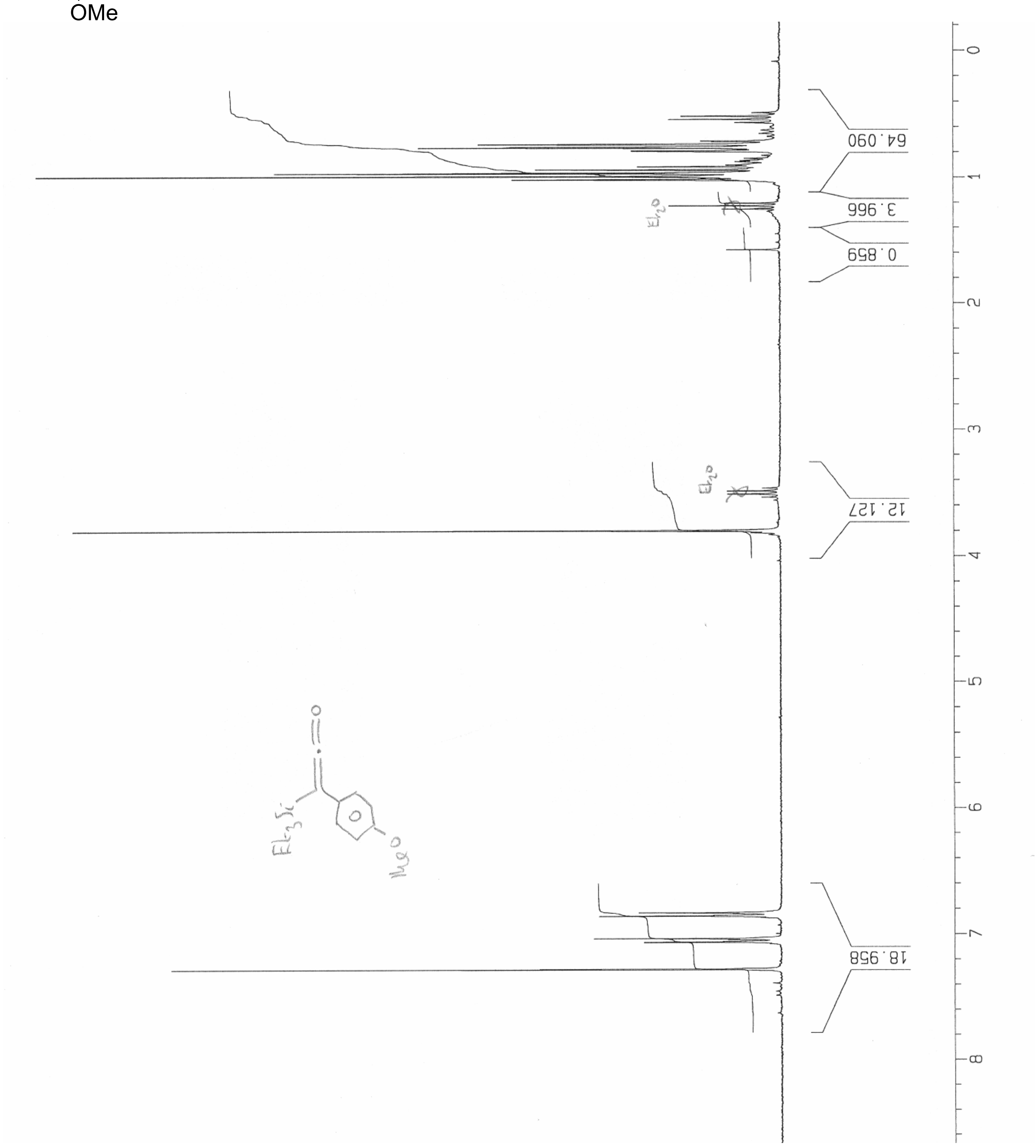

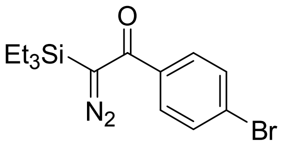

2g

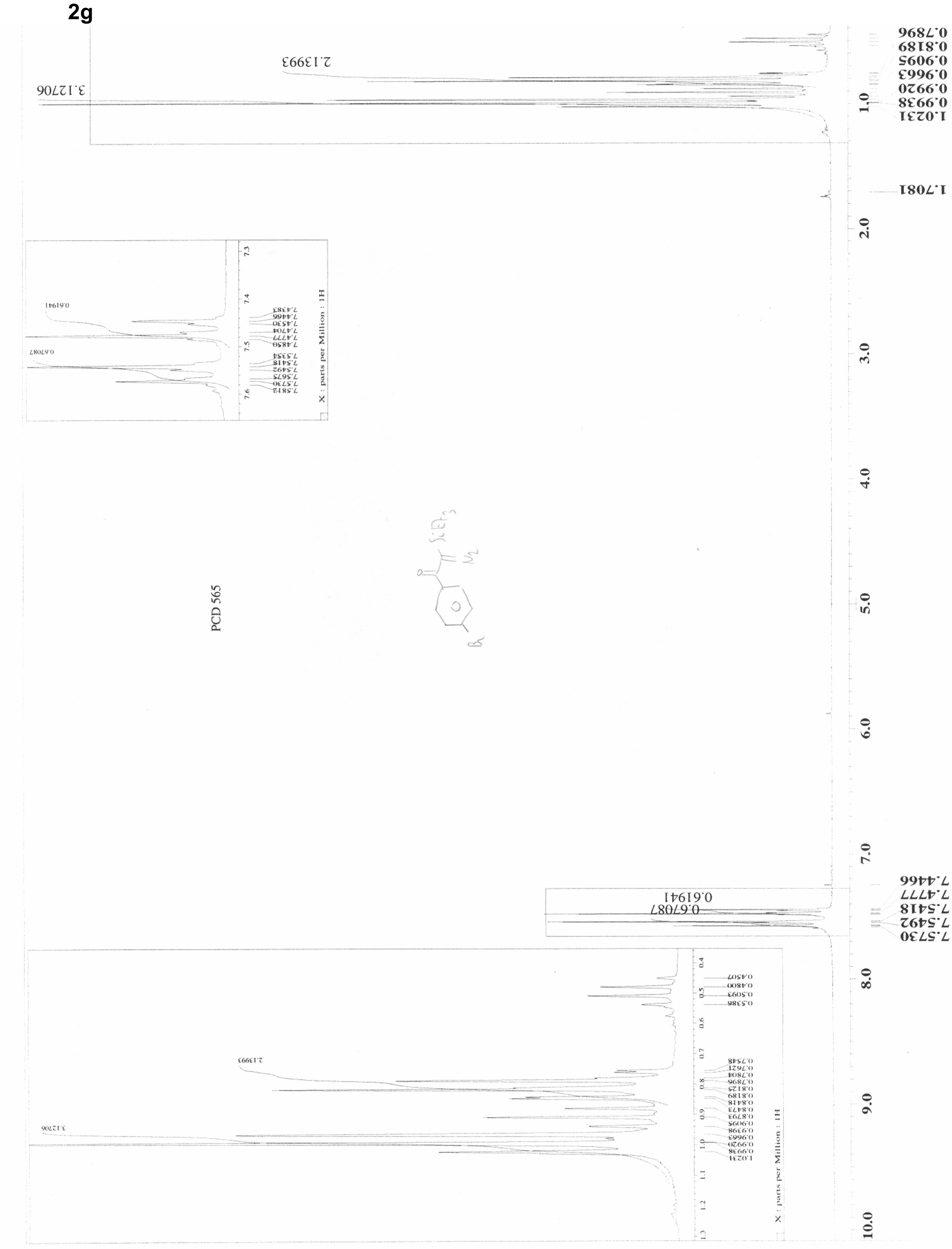

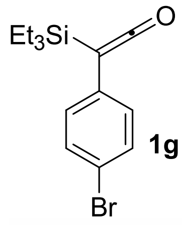

1g

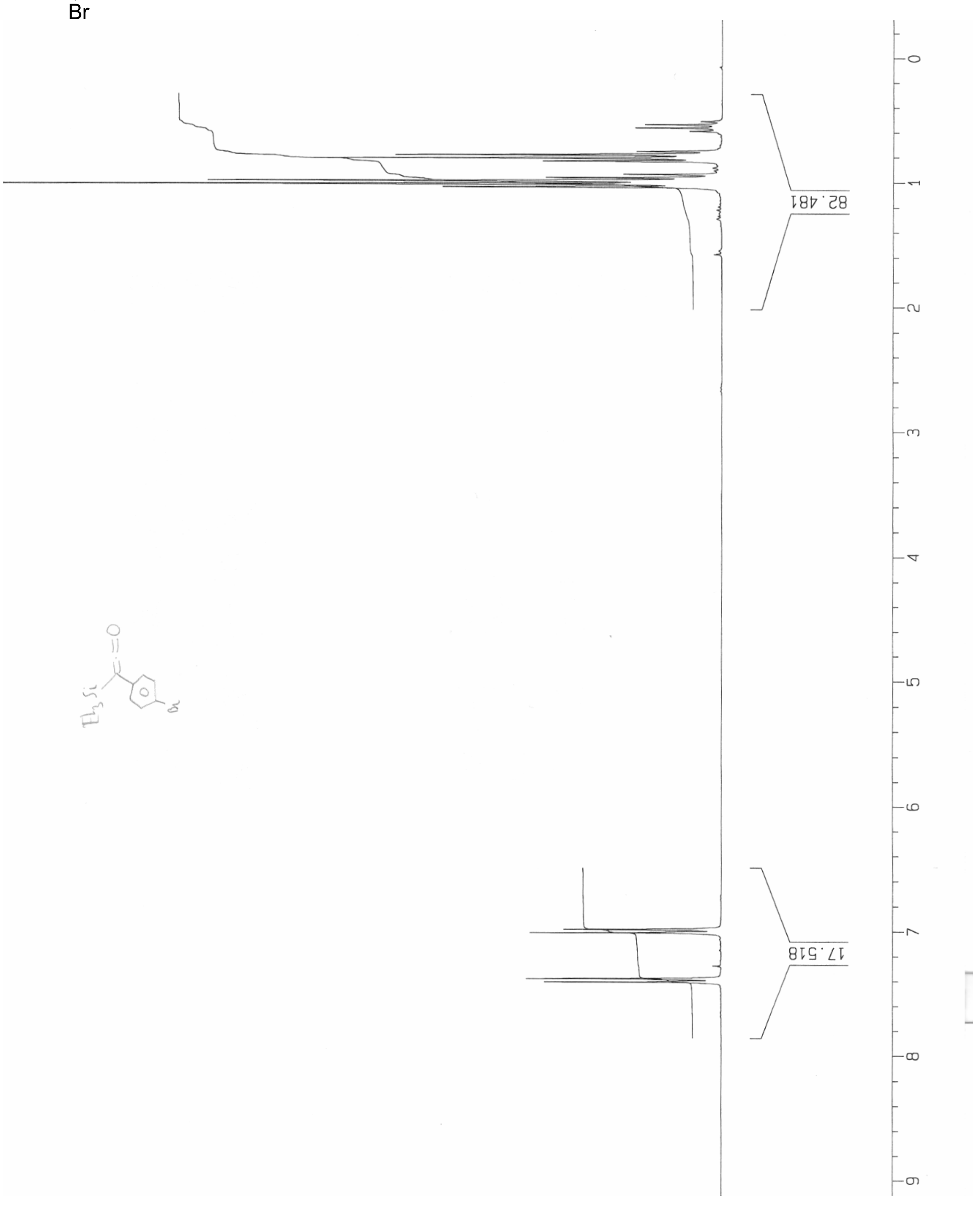

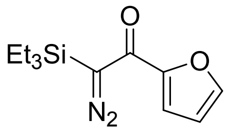

2h

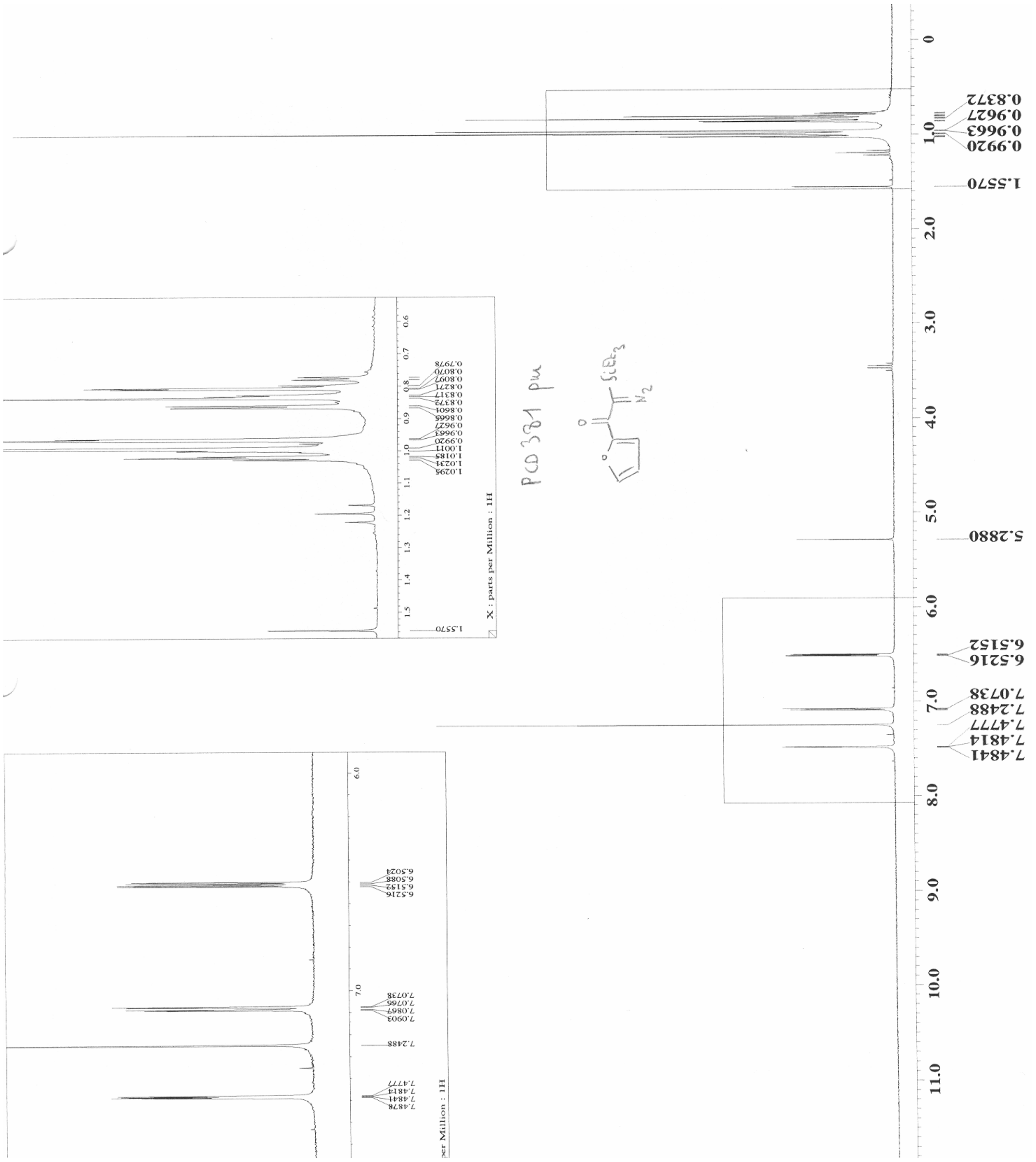

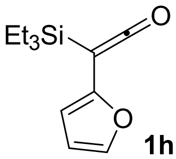

Et3Si
O

1h

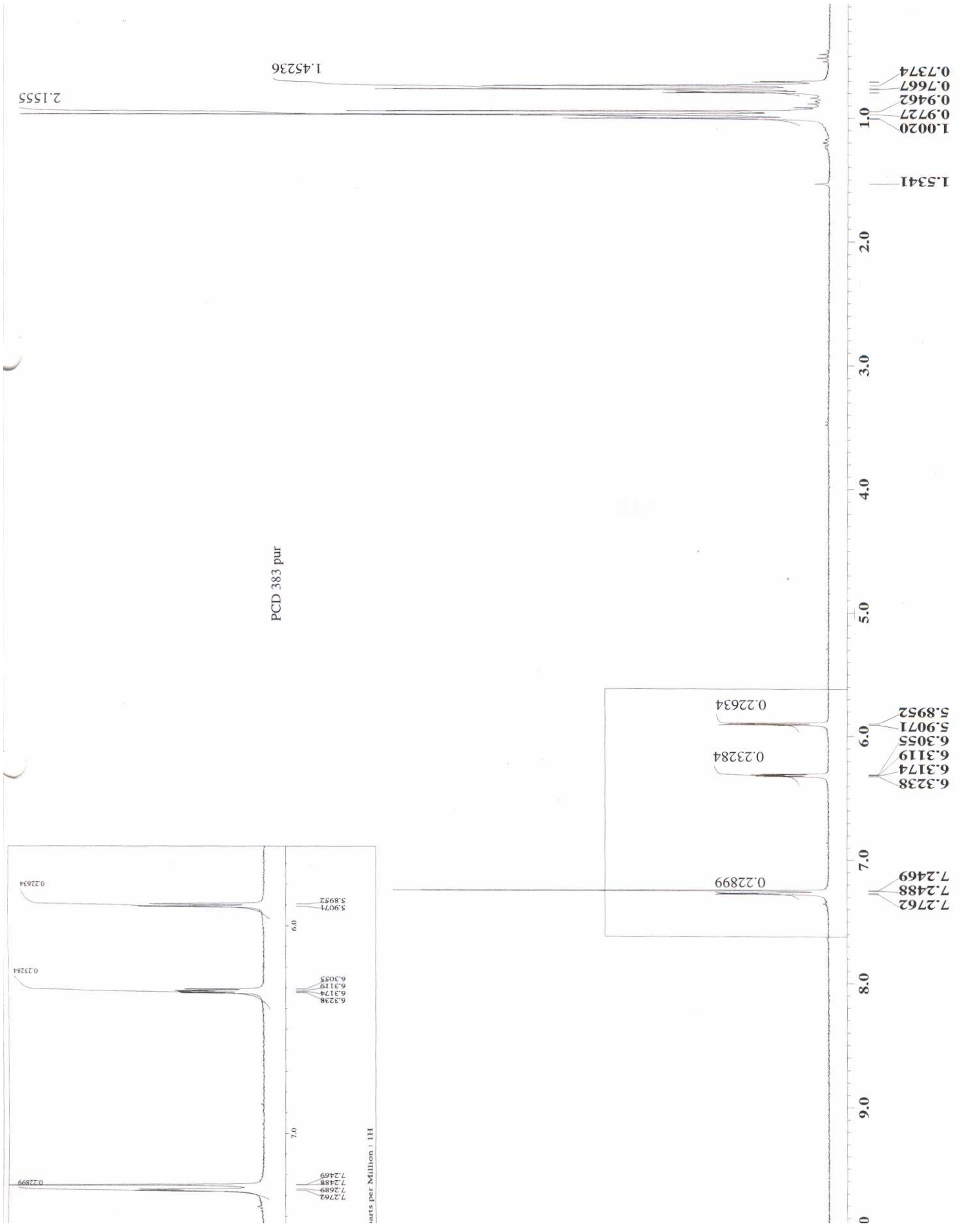

PCD 383 pur
1.45236
2.1555
0.7374
0.7667
0.9462
0.9727
1.0020
1.5341
0.22634
0.23284
0.22899
5.8952
5.9071
6.3055
6.3119
6.3174
6.3238
7.2469
7.2488
7.2762
1.0
2.0
3.0
4.0
5.0
6.0
7.0
8.0
9.0

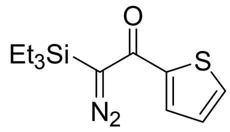

2i

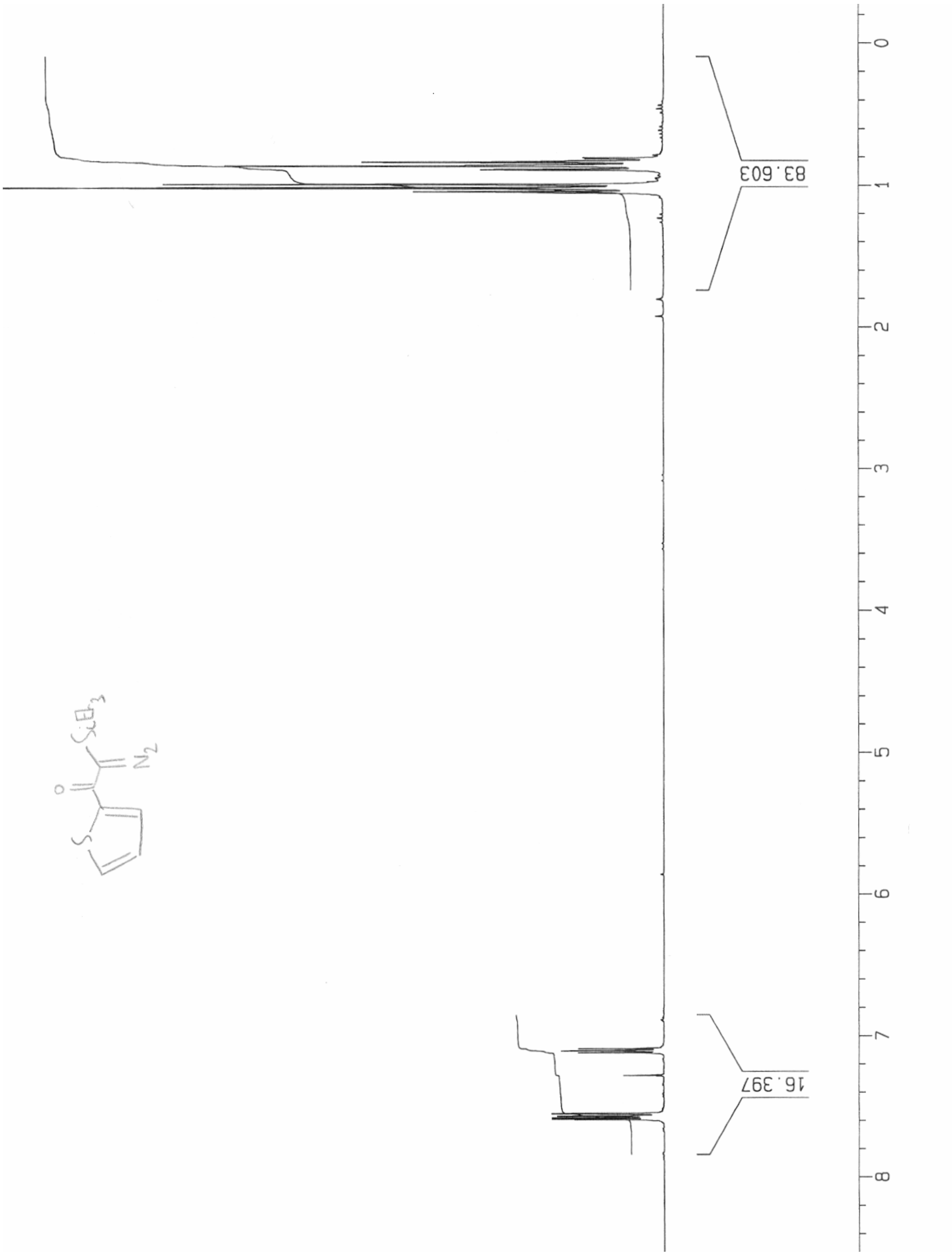

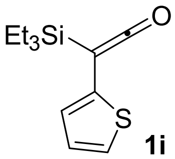

1i

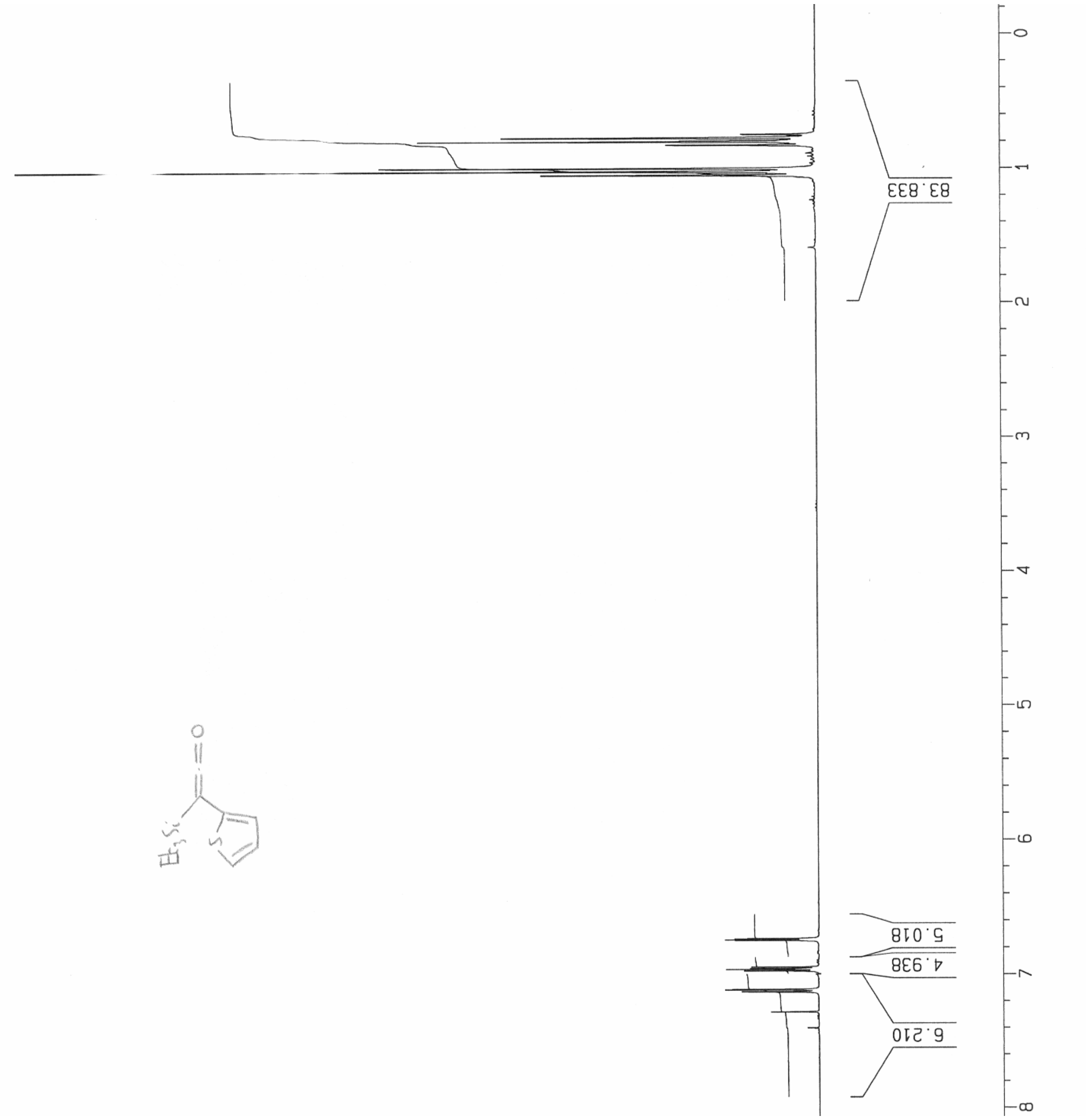

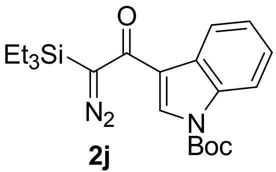

2j

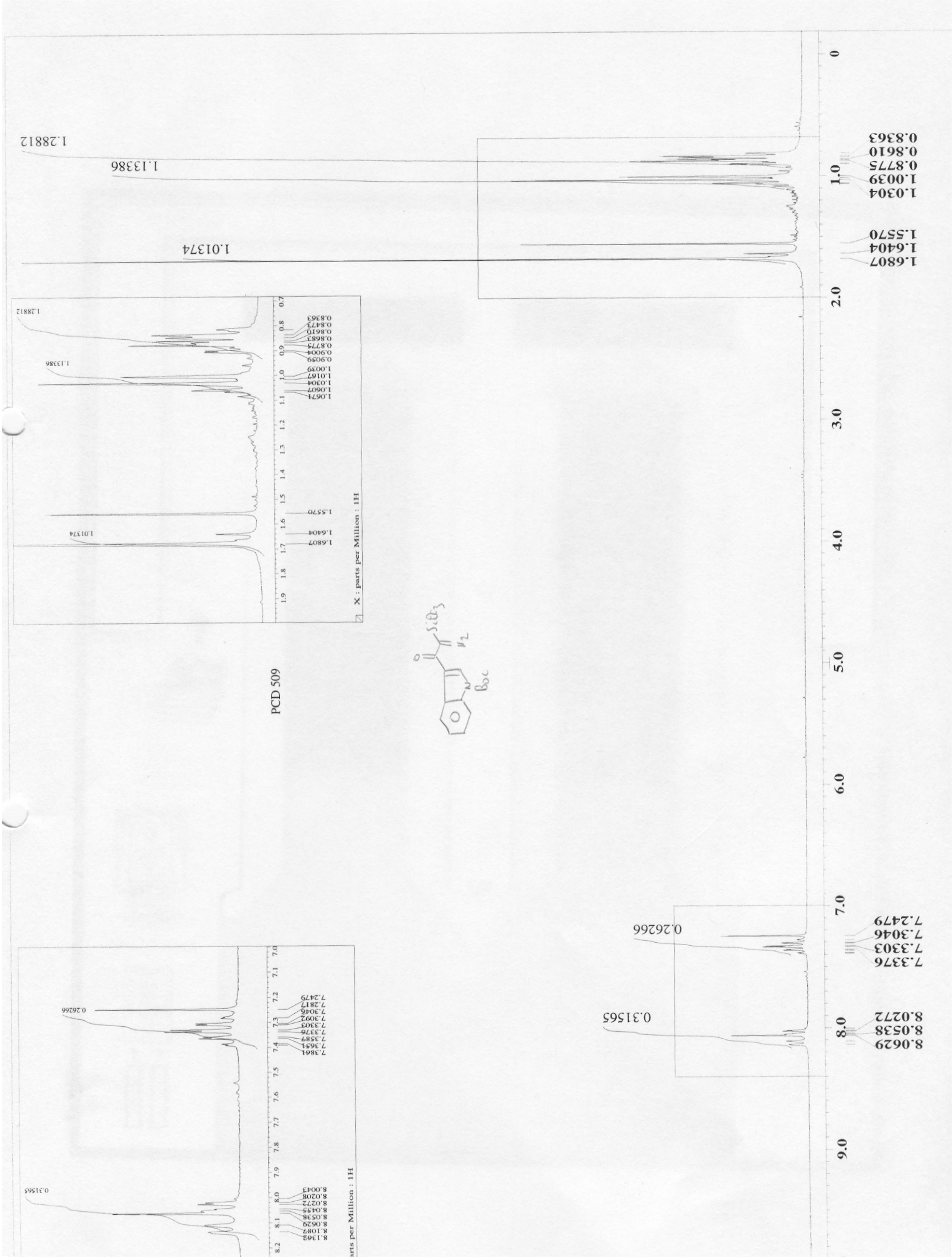

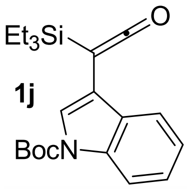

1j

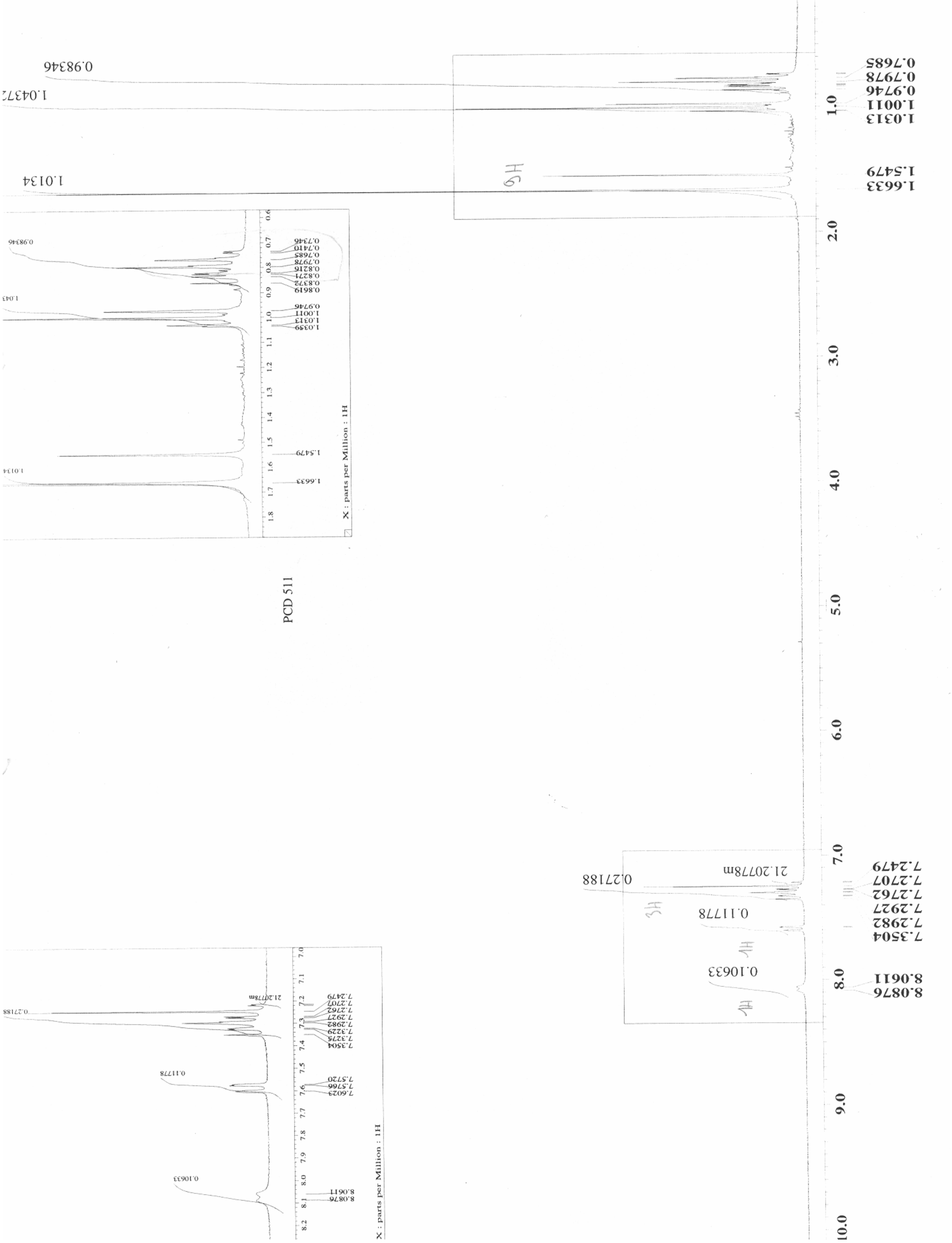

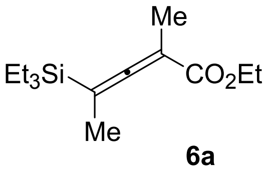

Me
Et3Si
CO2Et
Me

6a

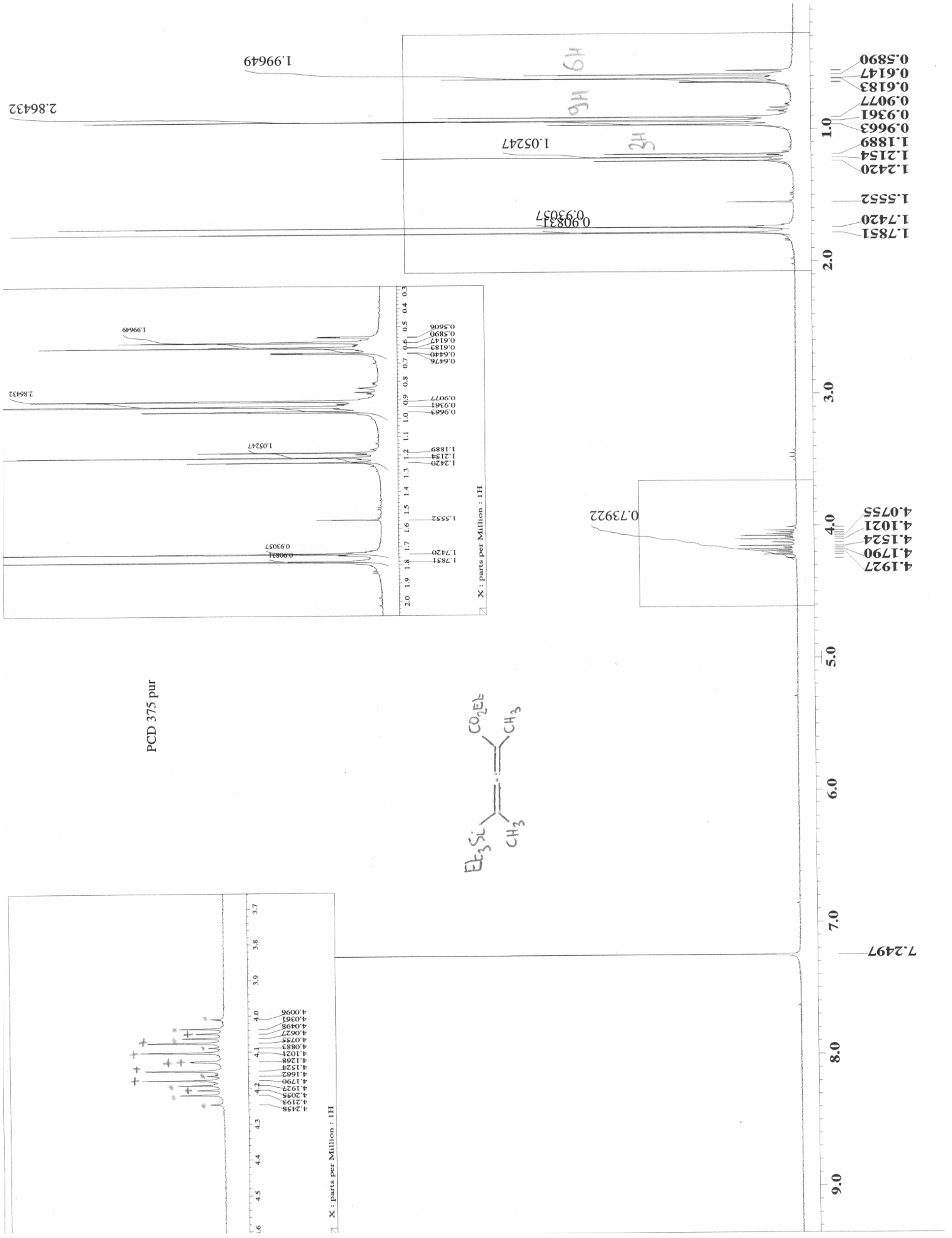

PCD 375 pur
1.99649
2.86432
1.05247
0.90831
0.93057
0.73922
0.5890
0.6147
0.6183
0.9077
0.9361
0.9663
1.1889
1.2154
1.2420
1.5552
1.7420
1.7851
4.0755
4.1021
4.1524
4.1790
4.1927
7.2497
X : parts per Million : 1H

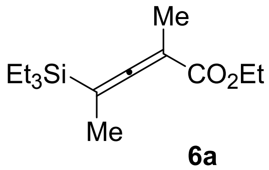
Me
Et3Si
CO2Et
Me

6a

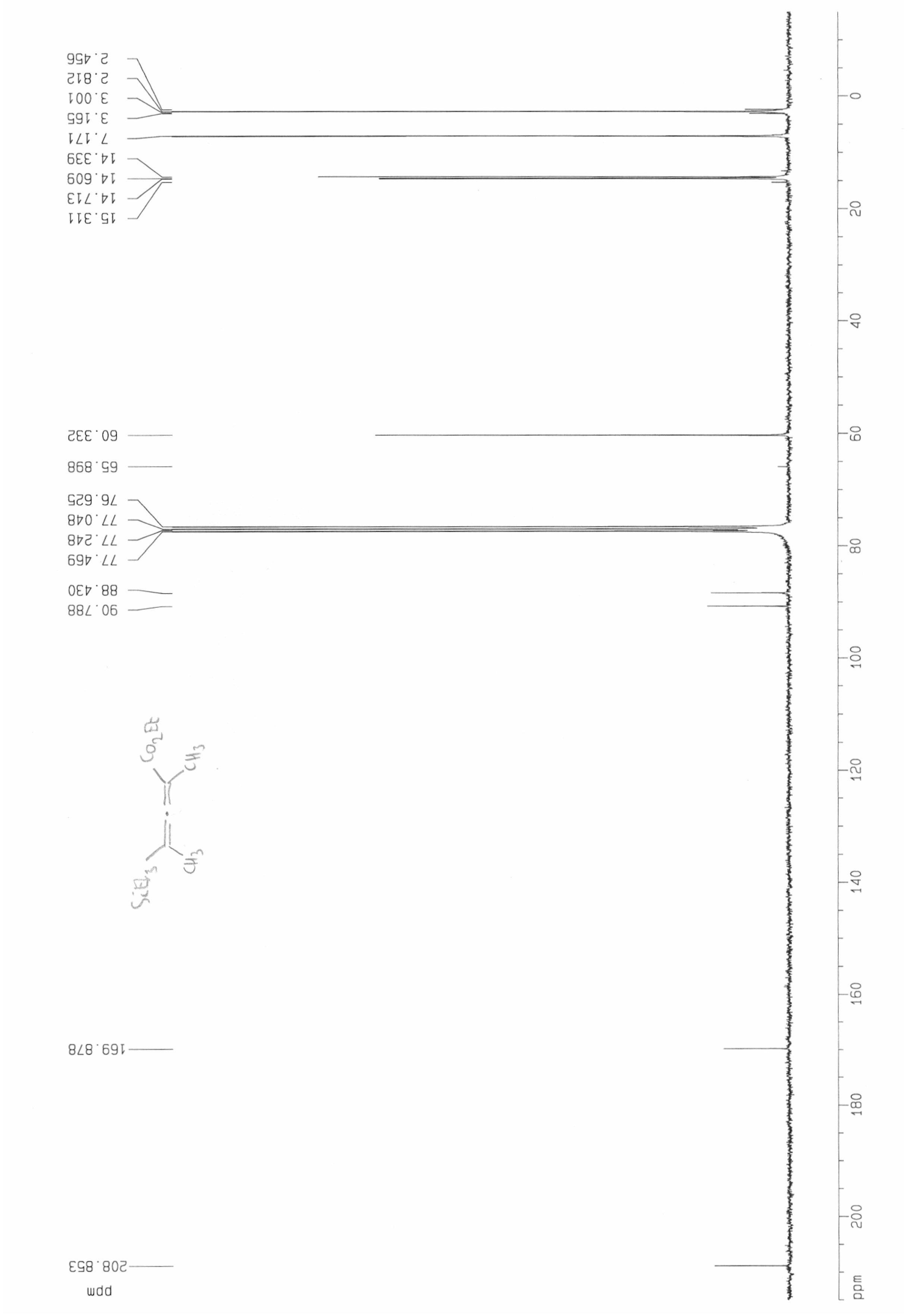
2.456
2.812
3.001
3.165
7.171
14.339
14.609
14.713
15.311
60.332
65.898
76.625
77.048
77.248
77.469
88.430
90.788
169.878
208.853
ppm
0
20
40
60
80
100
120
140
160
180
200
ppm

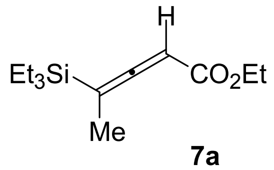

7a

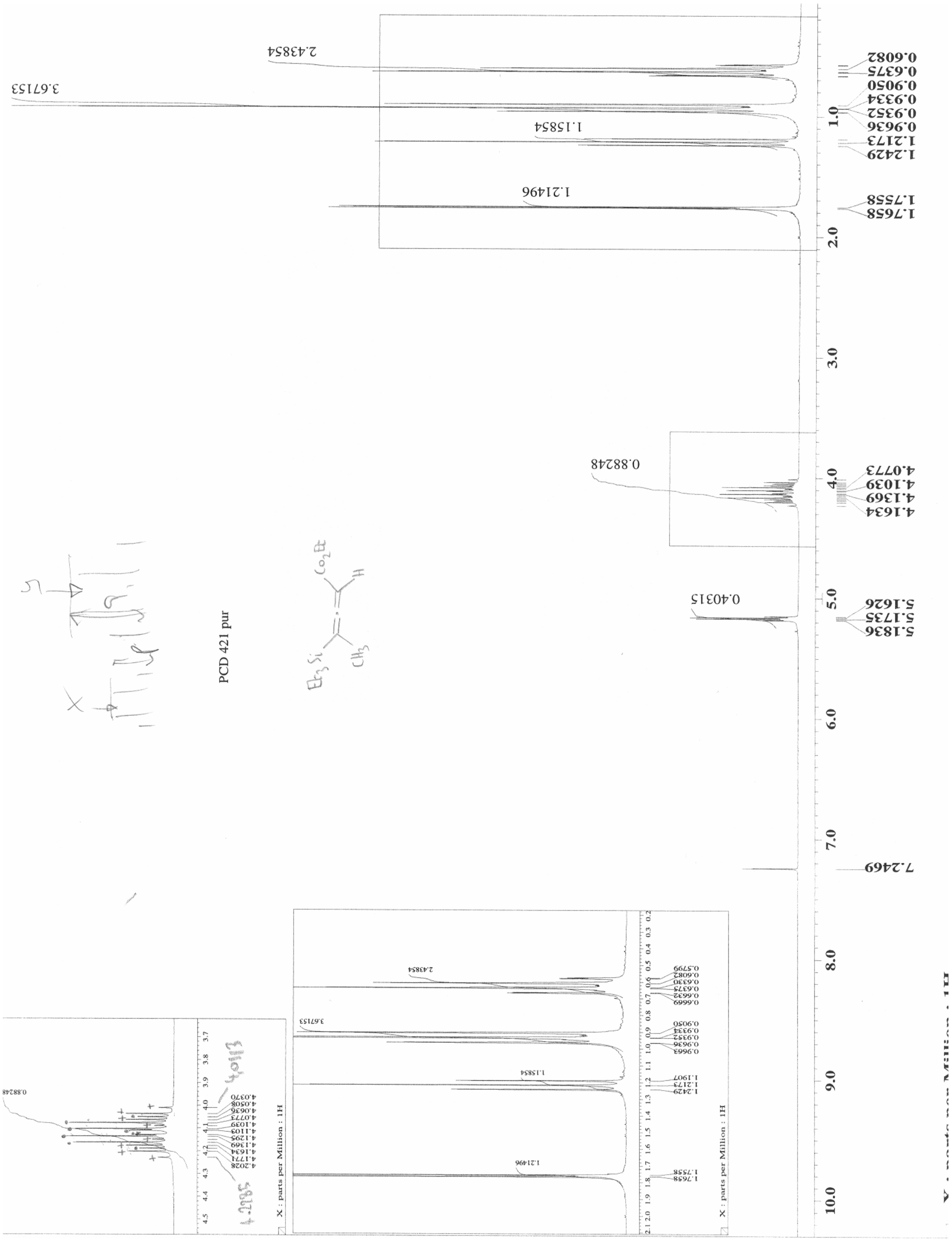

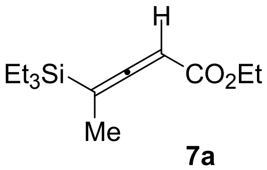
H
Et3Si
CO2Et
Me

7a

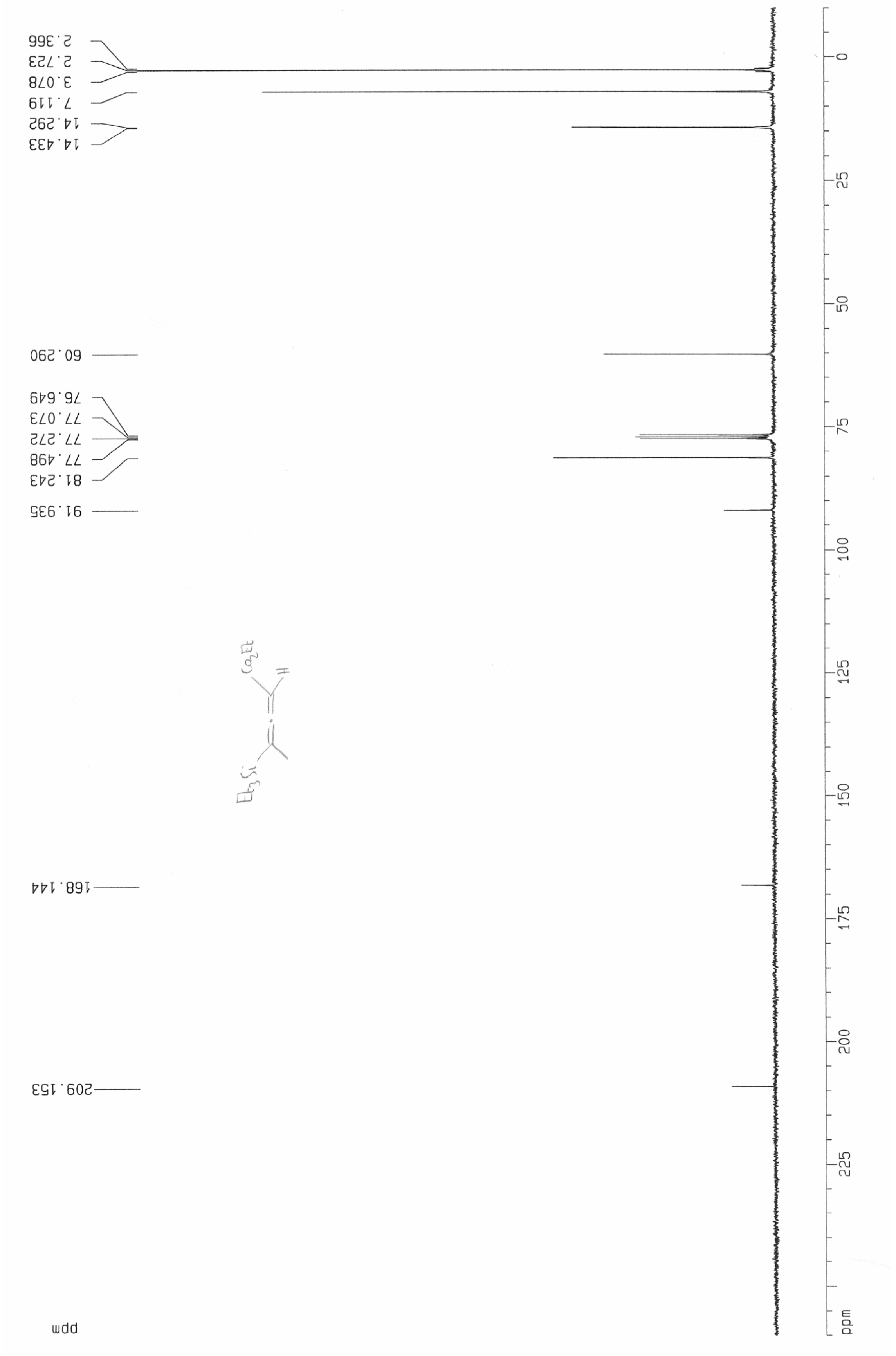
2.366
2.723
3.078
7.119
14.292
14.433
60.290
76.649
77.073
77.272
77.498
81.243
91.935
168.144
209.153
ppm
0
25
50
75
100
125
150
175
200
225
ppm

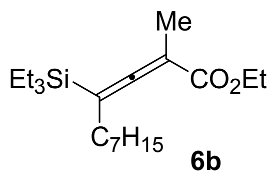

6b

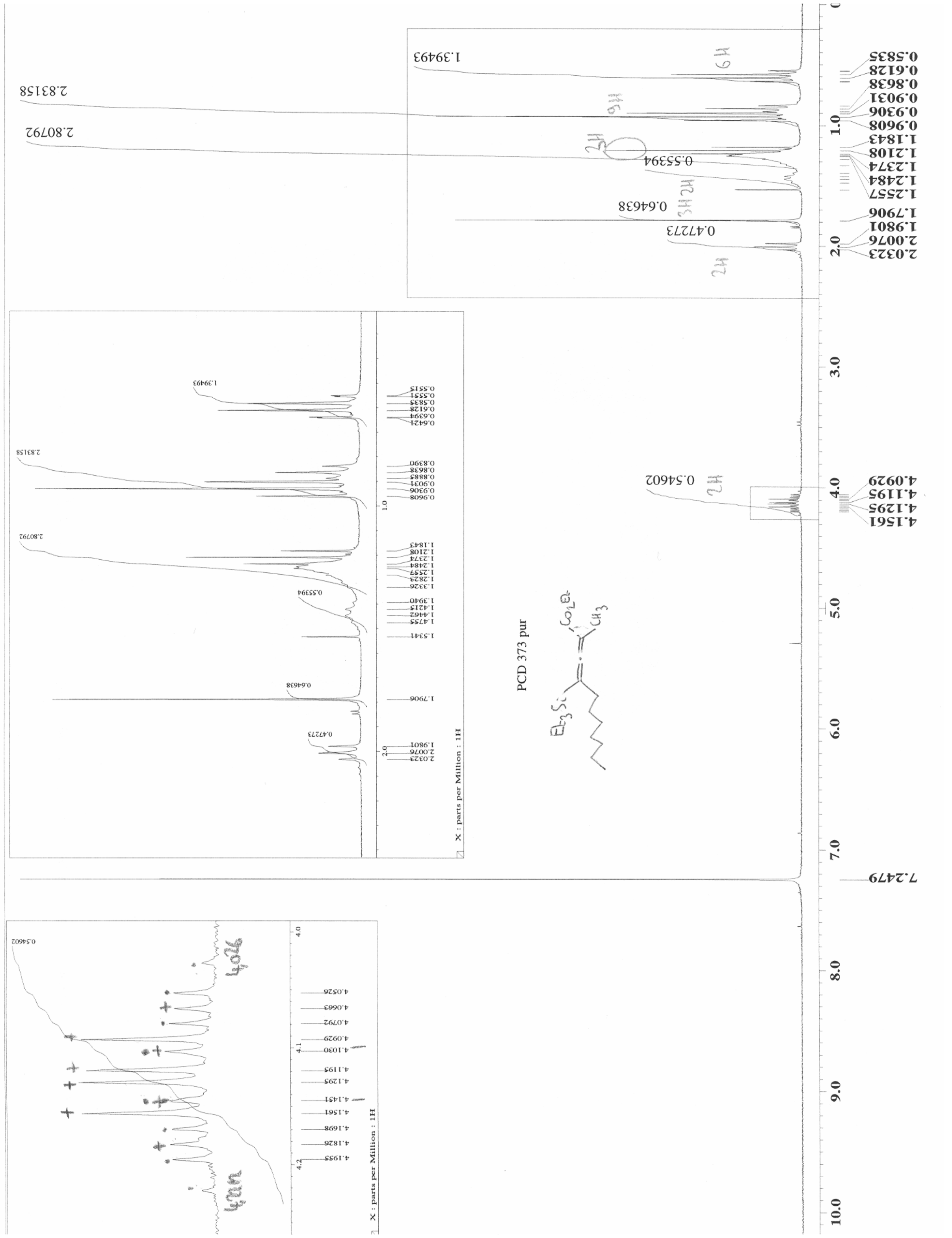

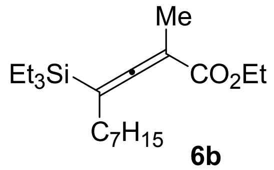
Me
Et3Si
CO2Et
C7H15

6b

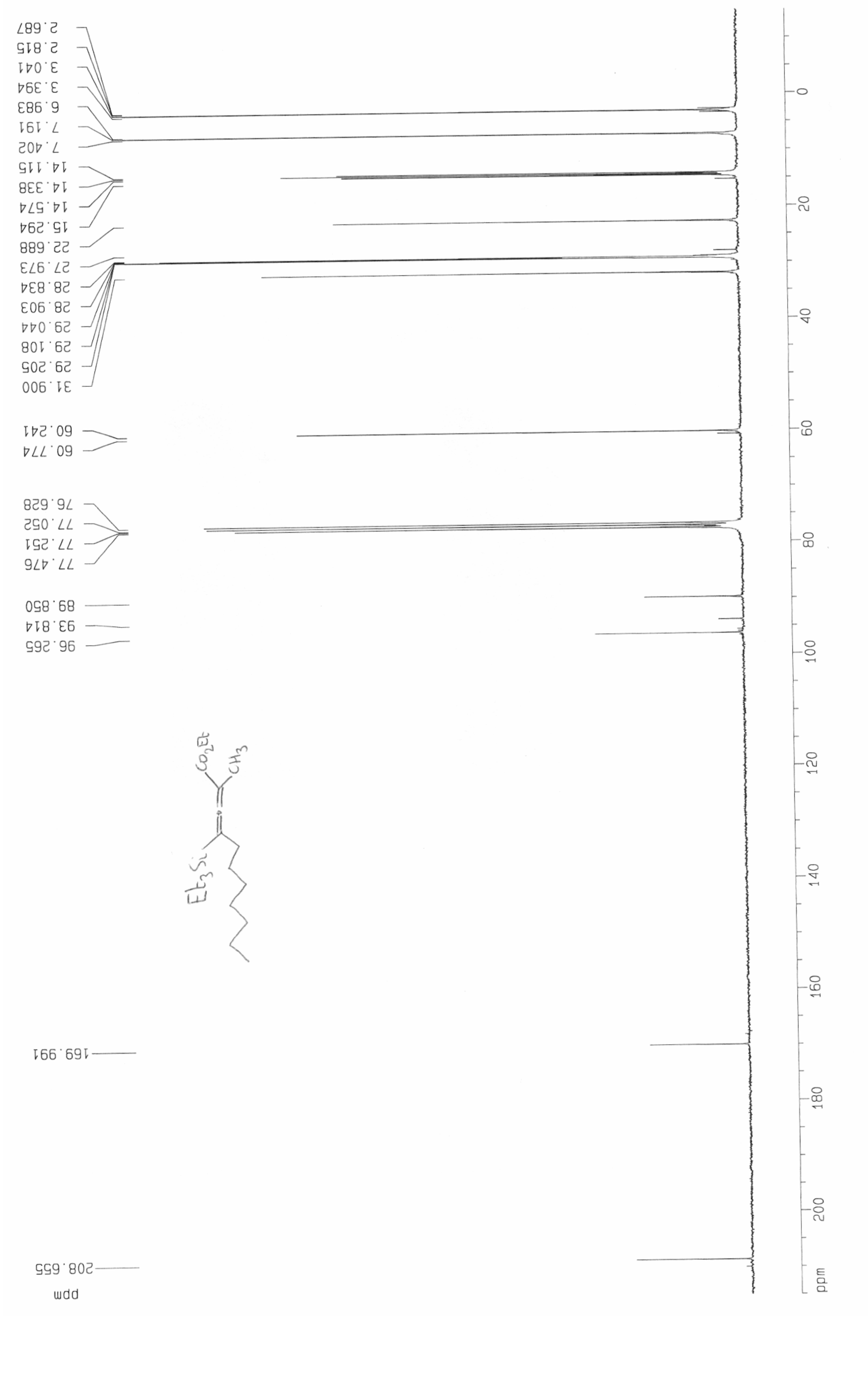
2.687
2.815
3.041
3.394
6.983
7.191
7.402
14.115
14.338
14.574
15.294
22.688
27.973
28.834
28.903
29.044
29.108
29.205
31.900
60.241
60.774
76.628
77.052
77.251
77.476
89.850
93.814
96.265
169.991
208.655
ppm
0
20
40
60
80
100
120
140
160
180
200
ppm

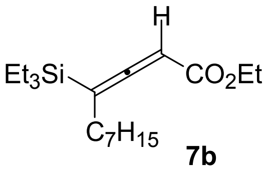
Et3Si
CO2Et
H
C7H15

7b

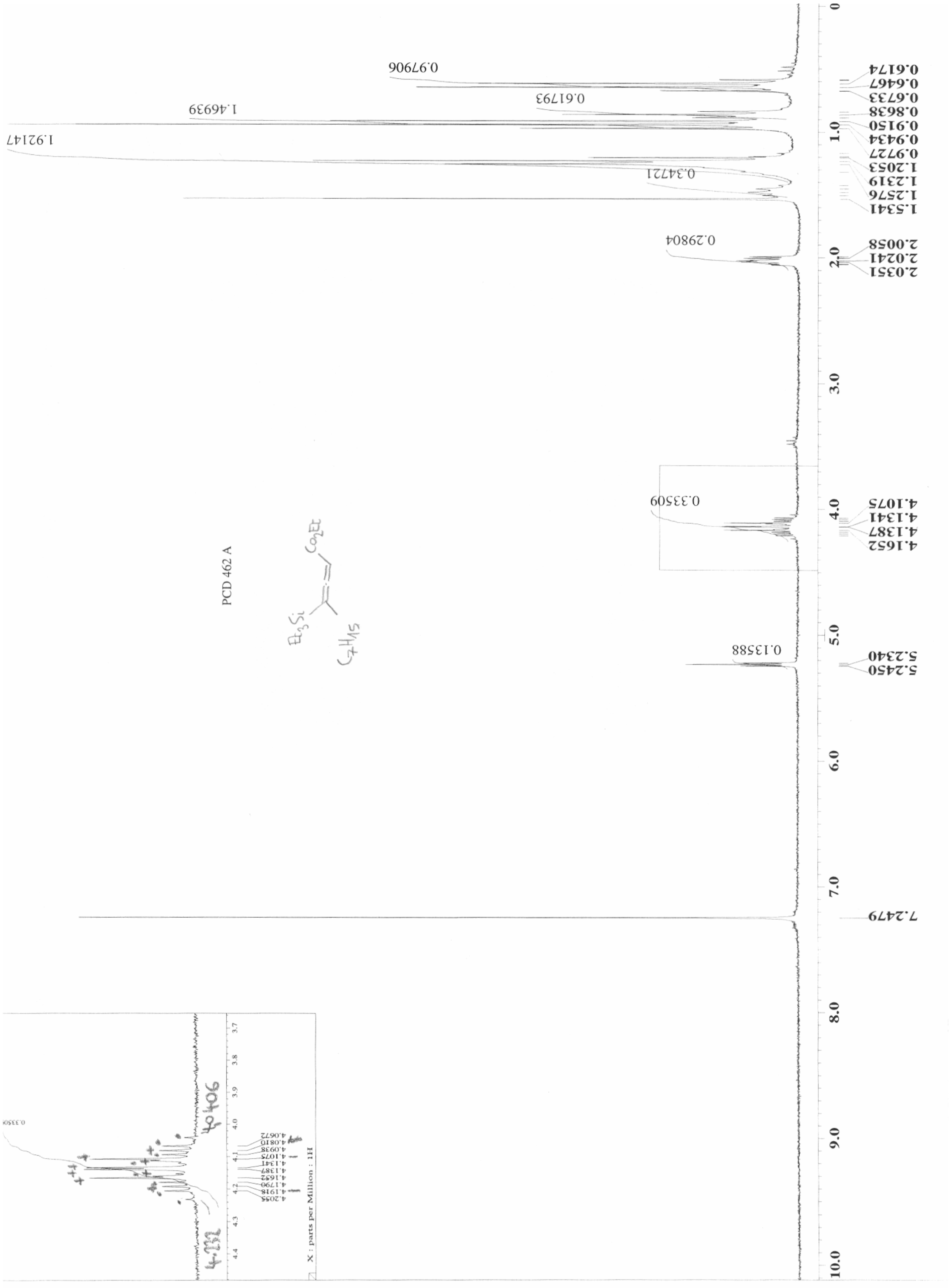
PCD 462 A
0.97906
1.46939
1.92147
0.61793
0.34721
0.29804
0.33509
0.13588
0.6174
0.6467
0.6733
0.8638
0.9150
0.9434
0.9727
1.2053
1.2319
1.2576
1.5341
2.0058
2.0241
2.0351
4.1075
4.1341
4.1387
4.1652
5.2340
5.2450
7.2479
0
1.0
2.0
3.0
4.0
5.0
6.0
7.0
8.0
9.0
10.0

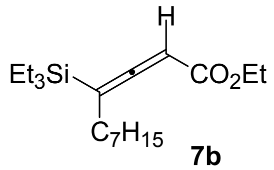
H
Et3Si
CO2Et
C7H15

7b

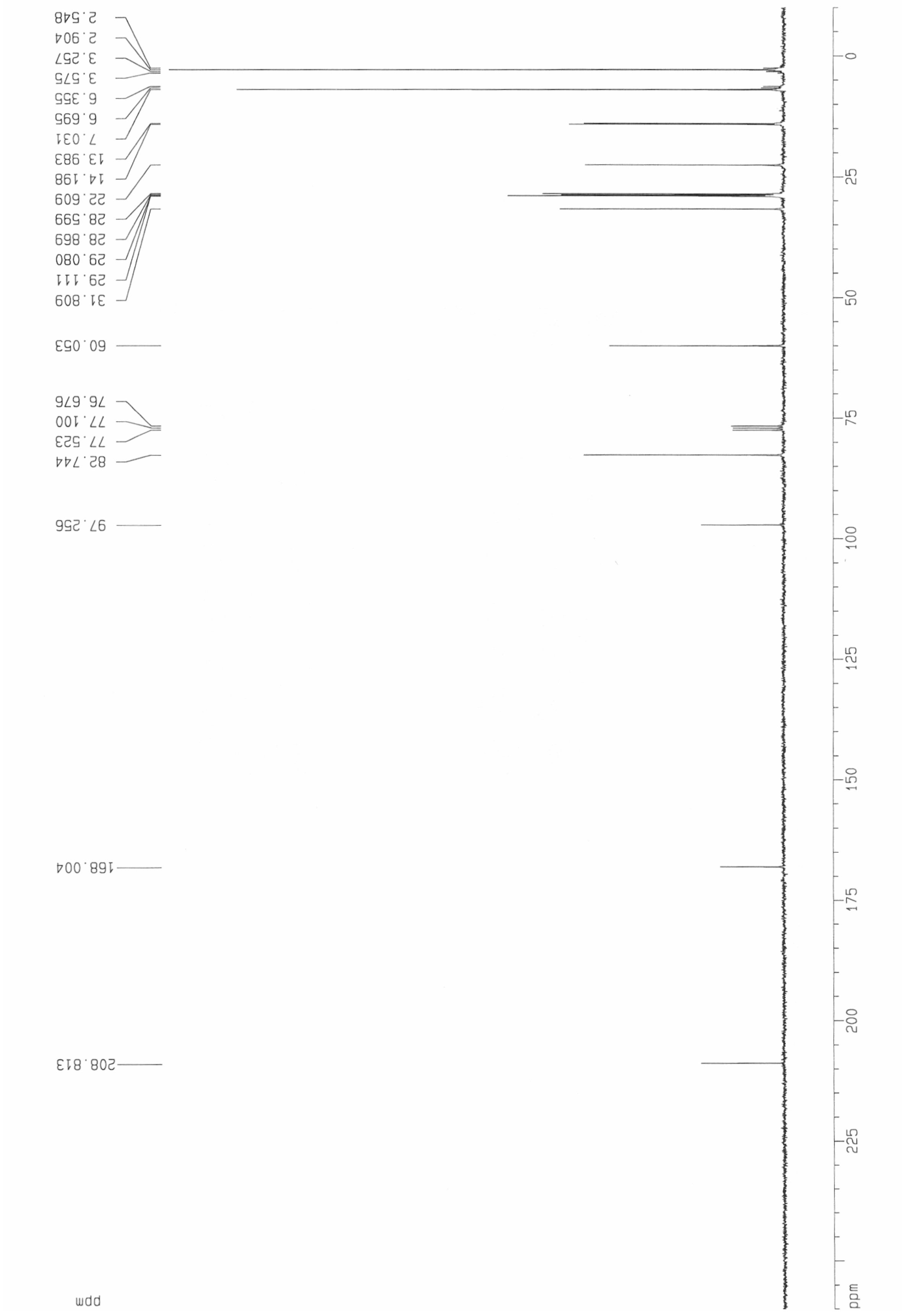
2.548
2.904
3.257
3.575
6.355
6.695
7.031
13.983
14.198
22.609
28.599
28.869
29.080
29.111
31.809
60.053
76.676
77.100
77.523
82.744
97.256
168.004
208.813
ppm
0
25
50
75
100
125
150
175
200
225
ppm

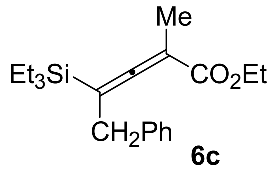

6c

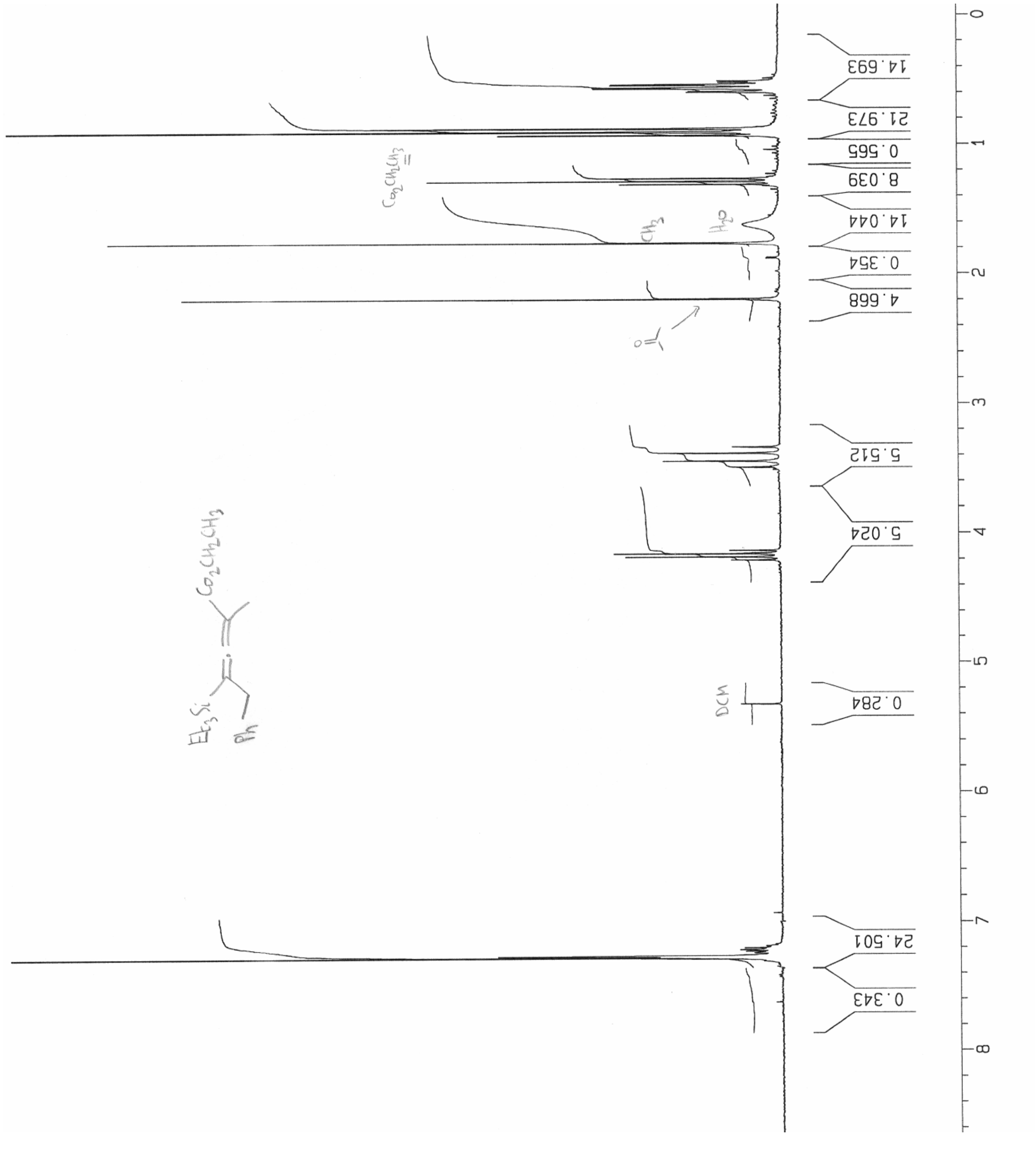

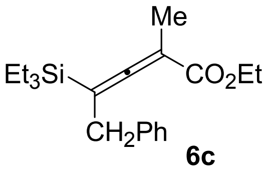

Me
Et_3Si
CO_2Et
CH_2Ph

6c

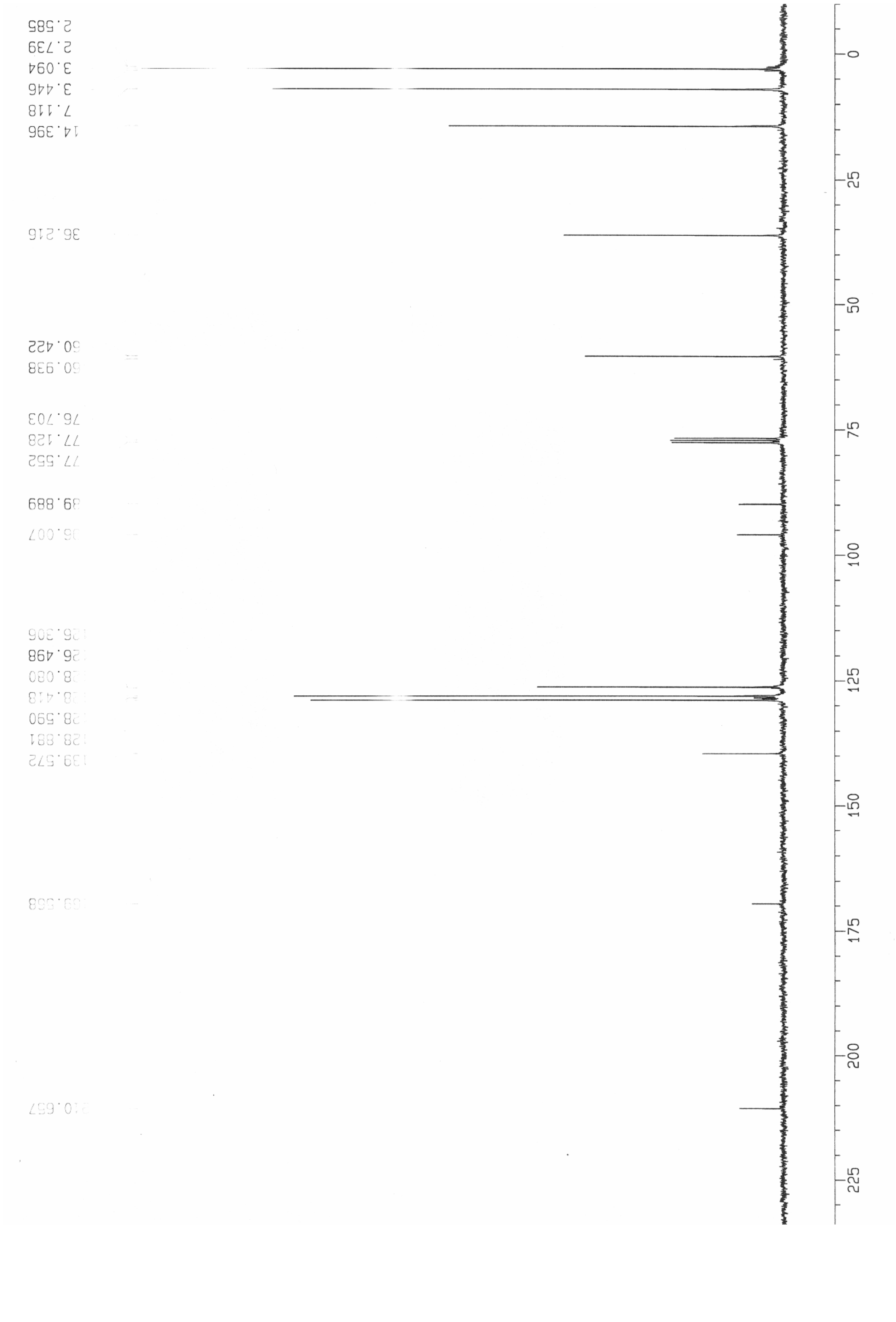

2.585
2.739
3.094
3.446
7.118
14.396
36.216
60.422
60.938
76.703
77.128
77.552
0
25
50
75
100
125
150
175
200
225

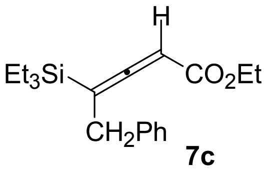
H
Et3Si
CO2Et
CH2Ph

7c

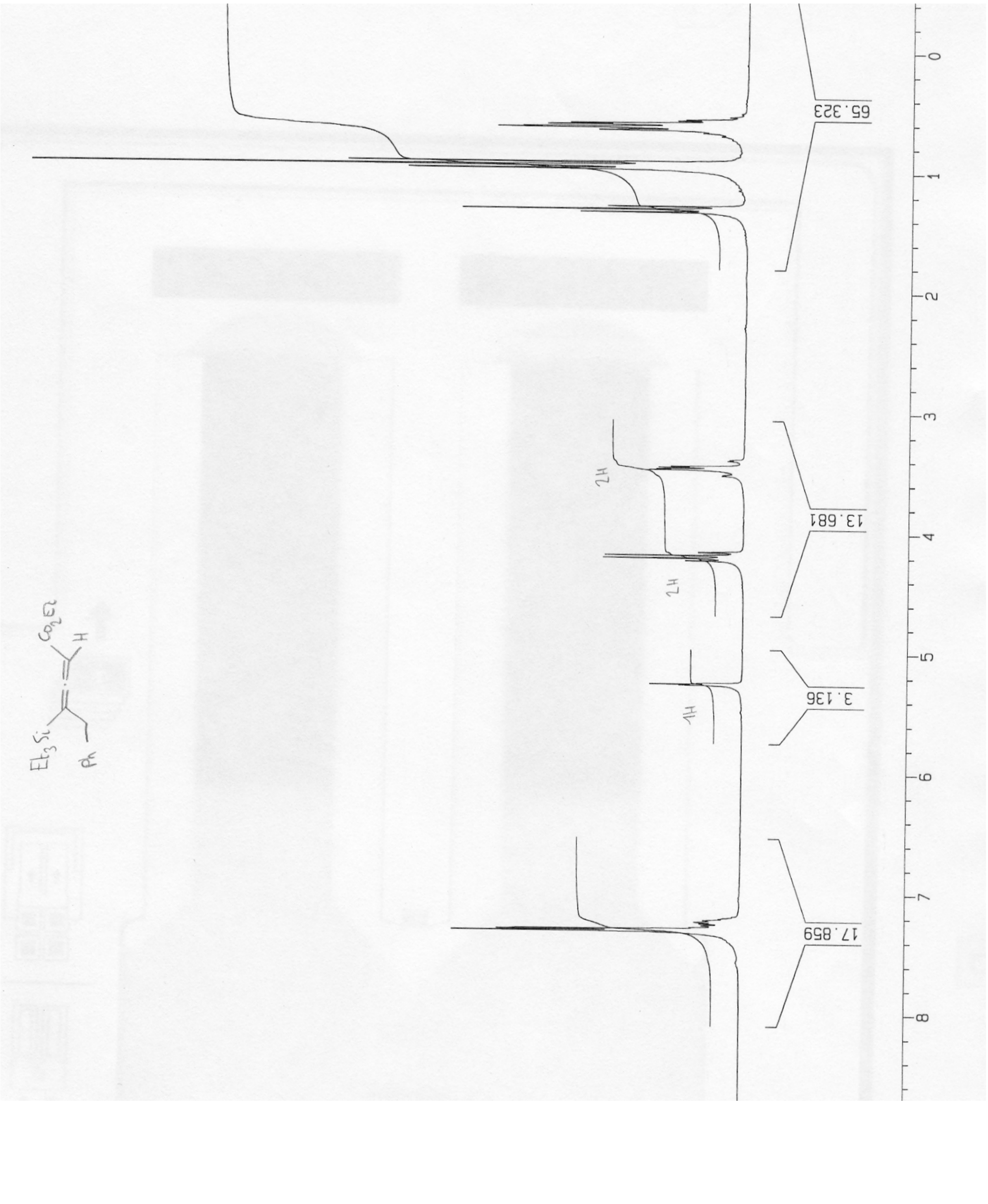
65.323
13.681
3.136
17.859
2H
2H
1H
0
1
2
3
4
5
6
7
8

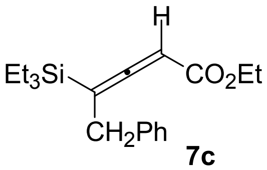

7c

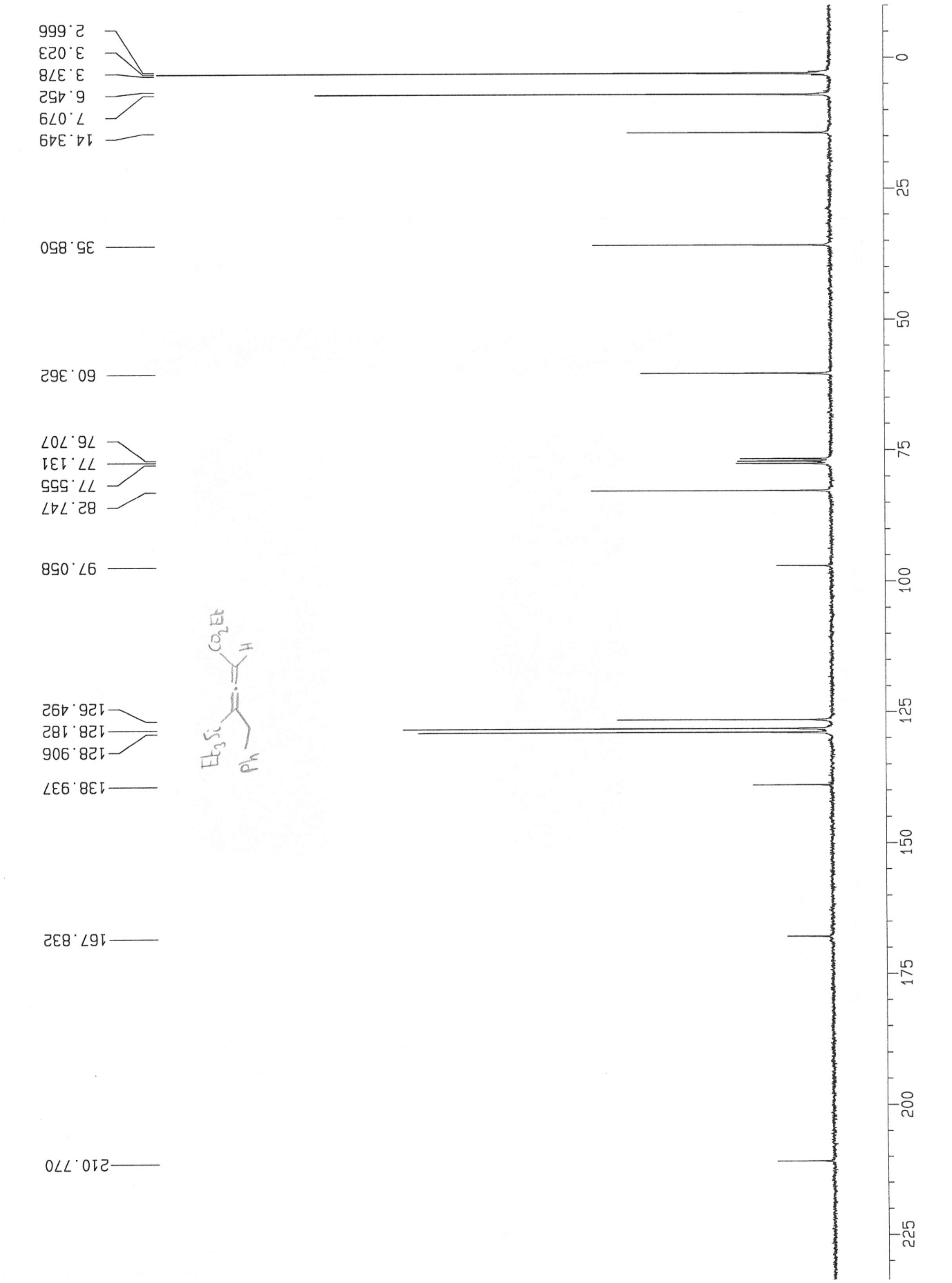
2.666
3.023
3.378
6.452
7.079
14.349
35.850
60.362
76.707
77.131
77.555
82.747
97.058
126.492
128.182
128.906
138.937
167.832
210.770
0
25
50
75
100
125
150
175
200
225

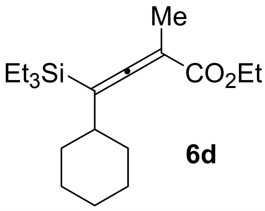

6d

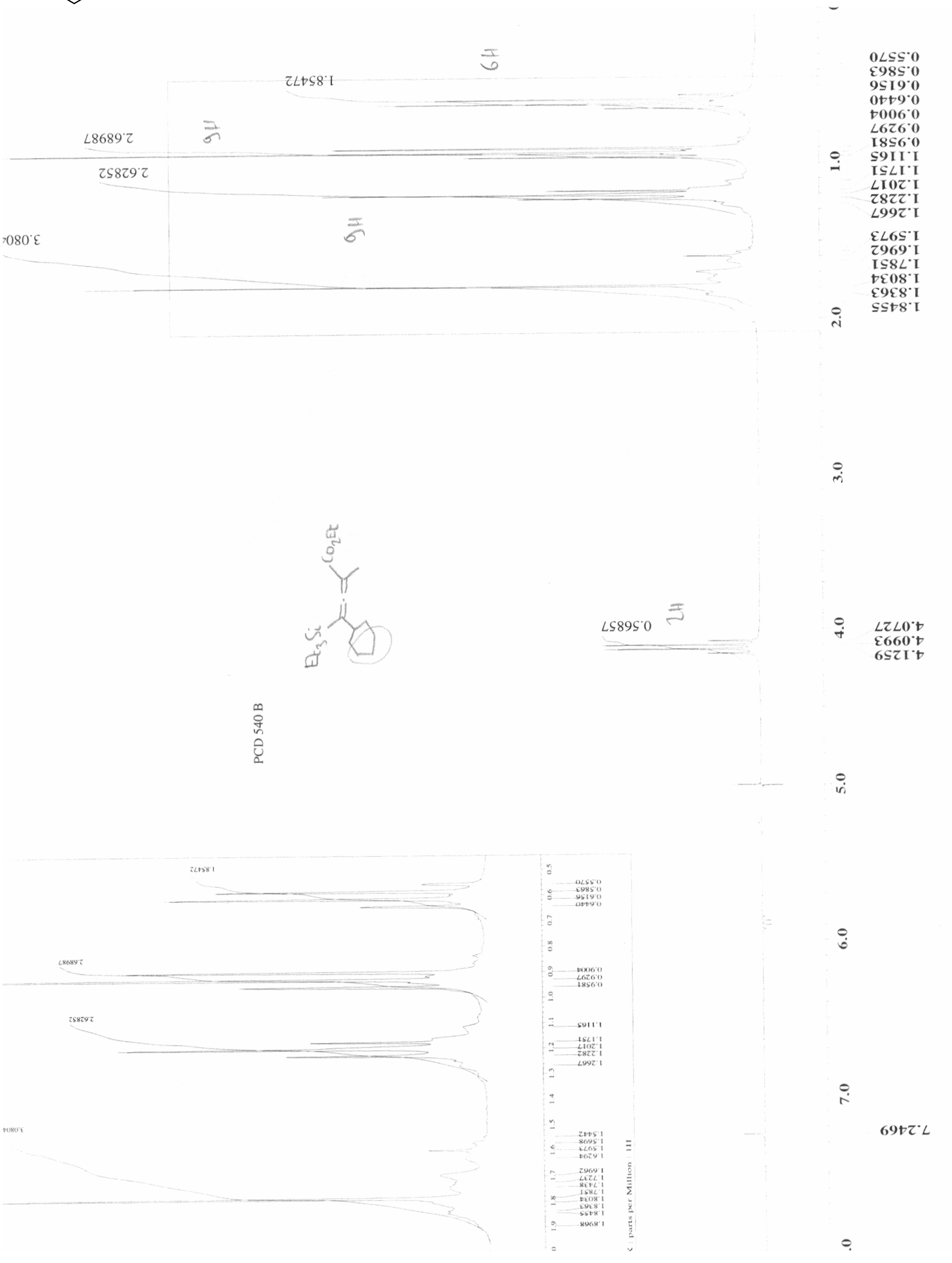

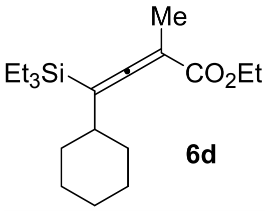
Me
Et3Si
CO2Et

6d

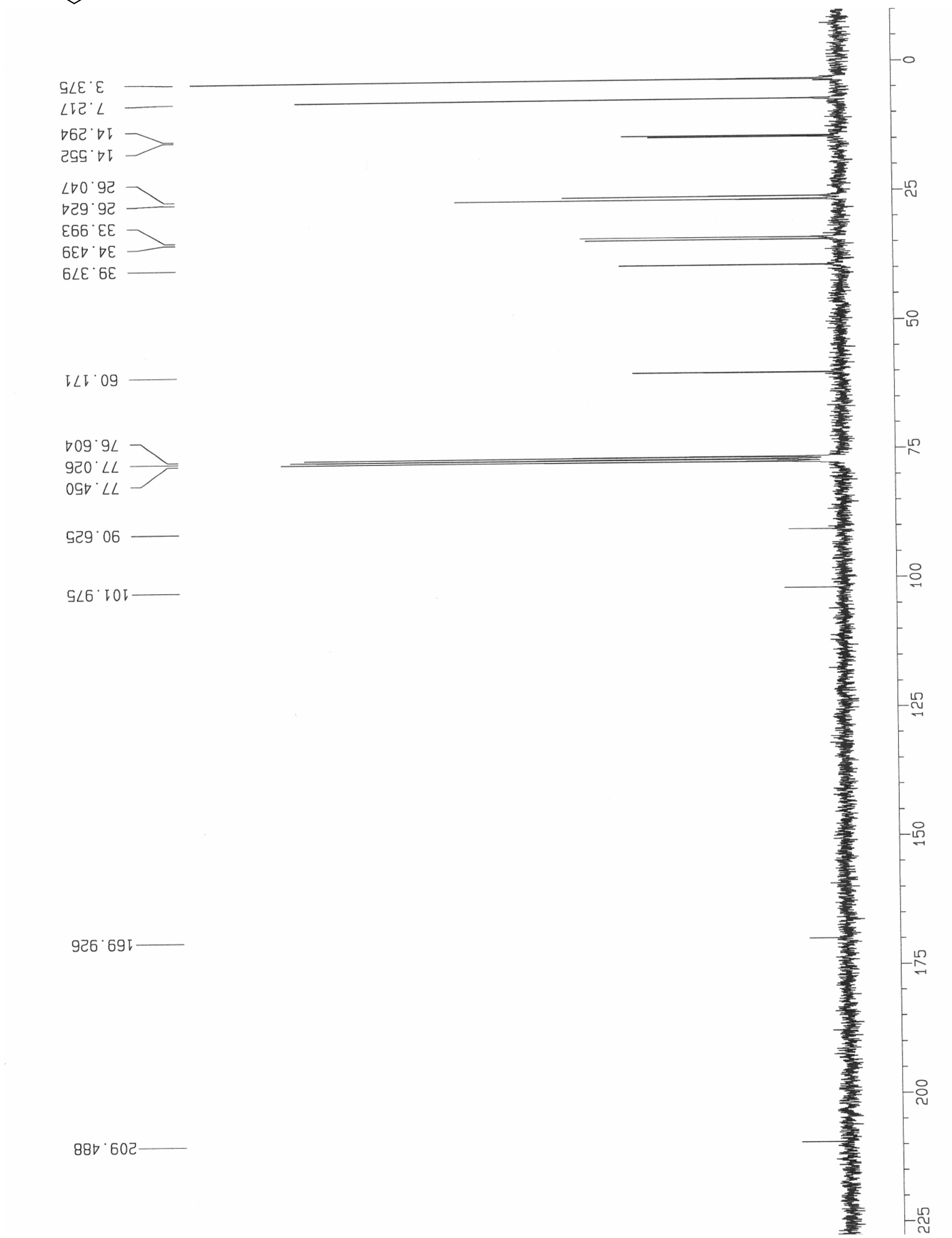
3.375
7.217
14.294
14.552
26.047
26.624
33.993
34.439
39.379
60.171
76.604
77.026
77.450
90.625
101.975
169.926
209.488
0
25
50
75
100
125
150
175
200
225

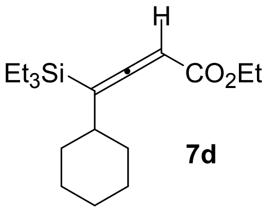

7d

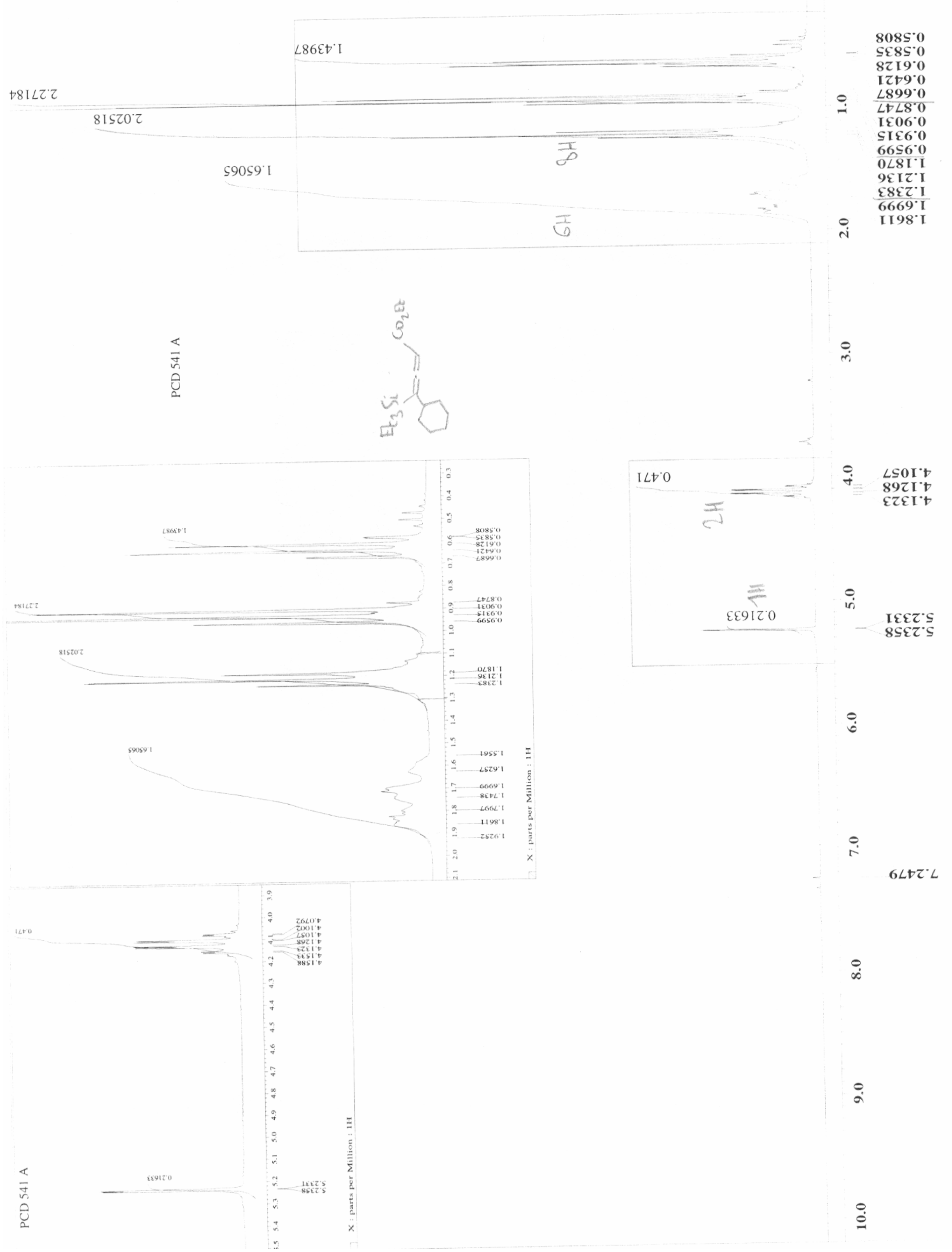

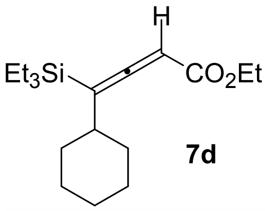
H
Et3Si
CO2Et

7d

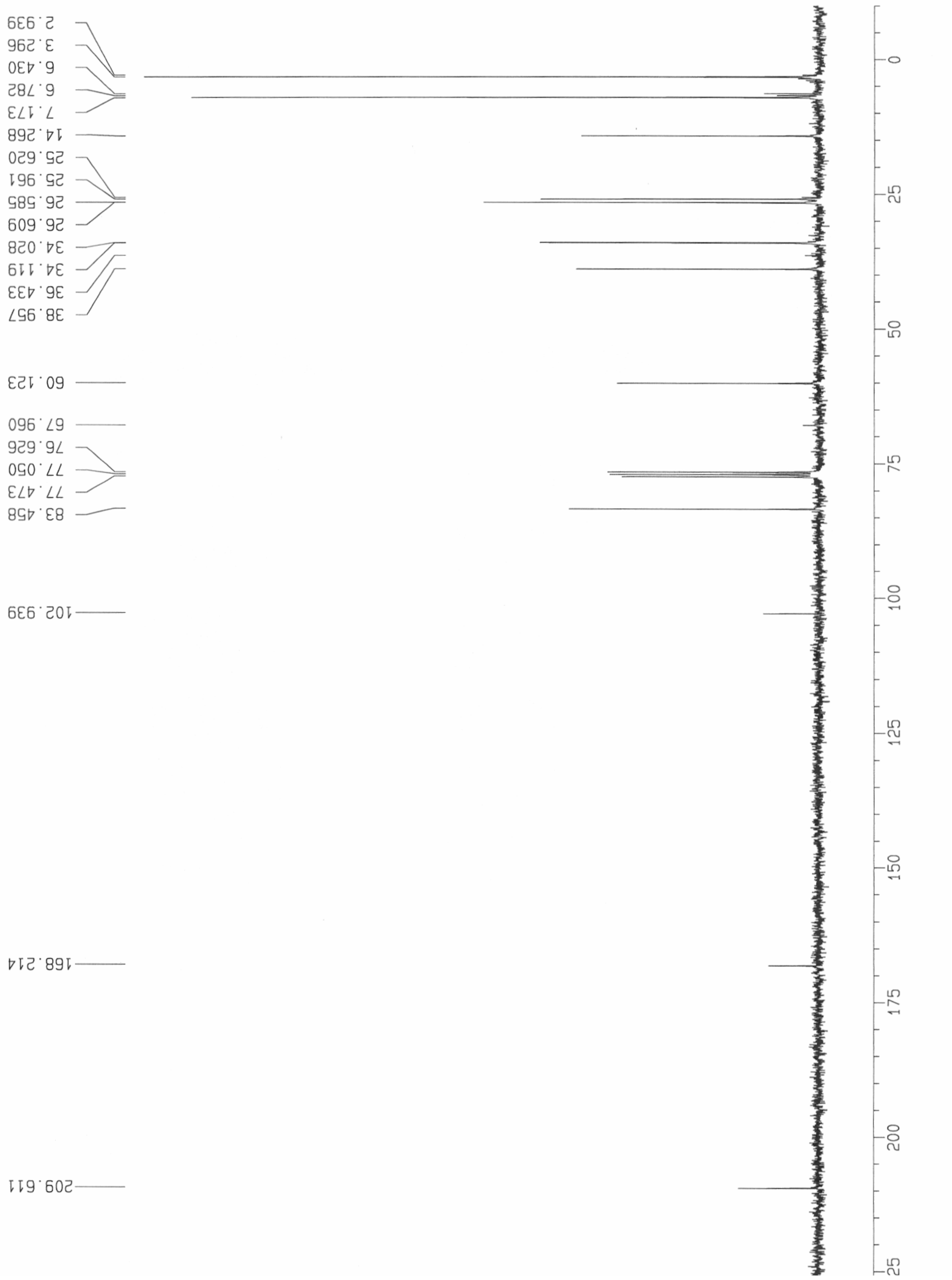
2.939
3.296
6.430
6.782
7.173
14.268
25.620
25.961
26.585
26.609
34.028
34.119
36.433
38.957
60.123
67.960
76.626
77.050
77.473
83.458
102.939
168.214
209.611
0
25
50
75
100
125
150
175
200

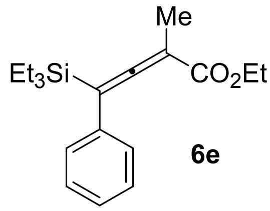
Me
Et_3Si
CO_2Et

6e

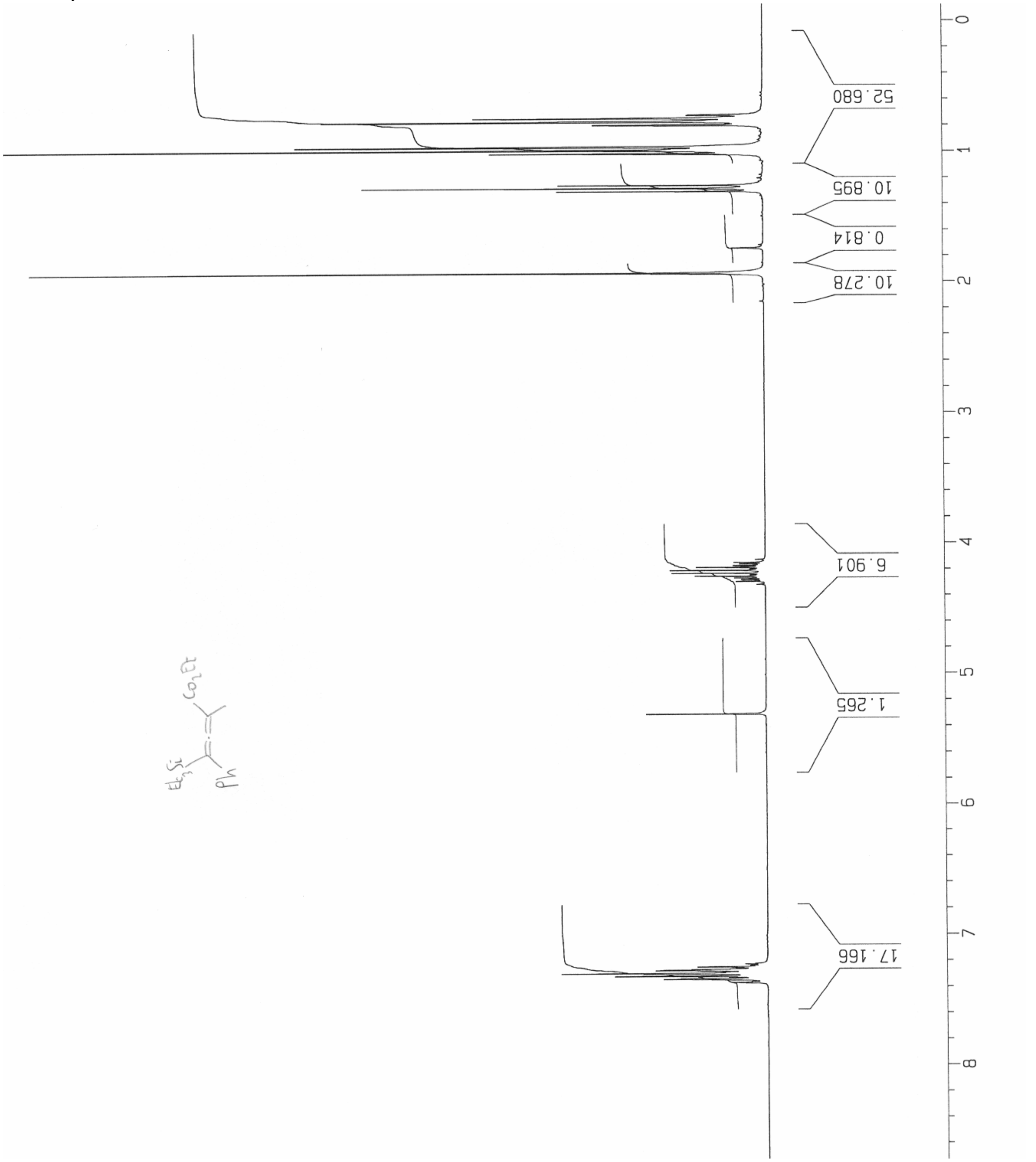
52.680
10.895
0.814
10.278
6.901
1.265
17.166
0
1
2
3
4
5
6
7
8

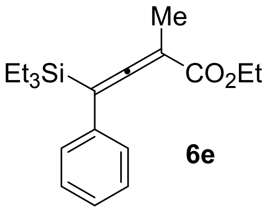
Me
Et3Si
CO2Et

6e

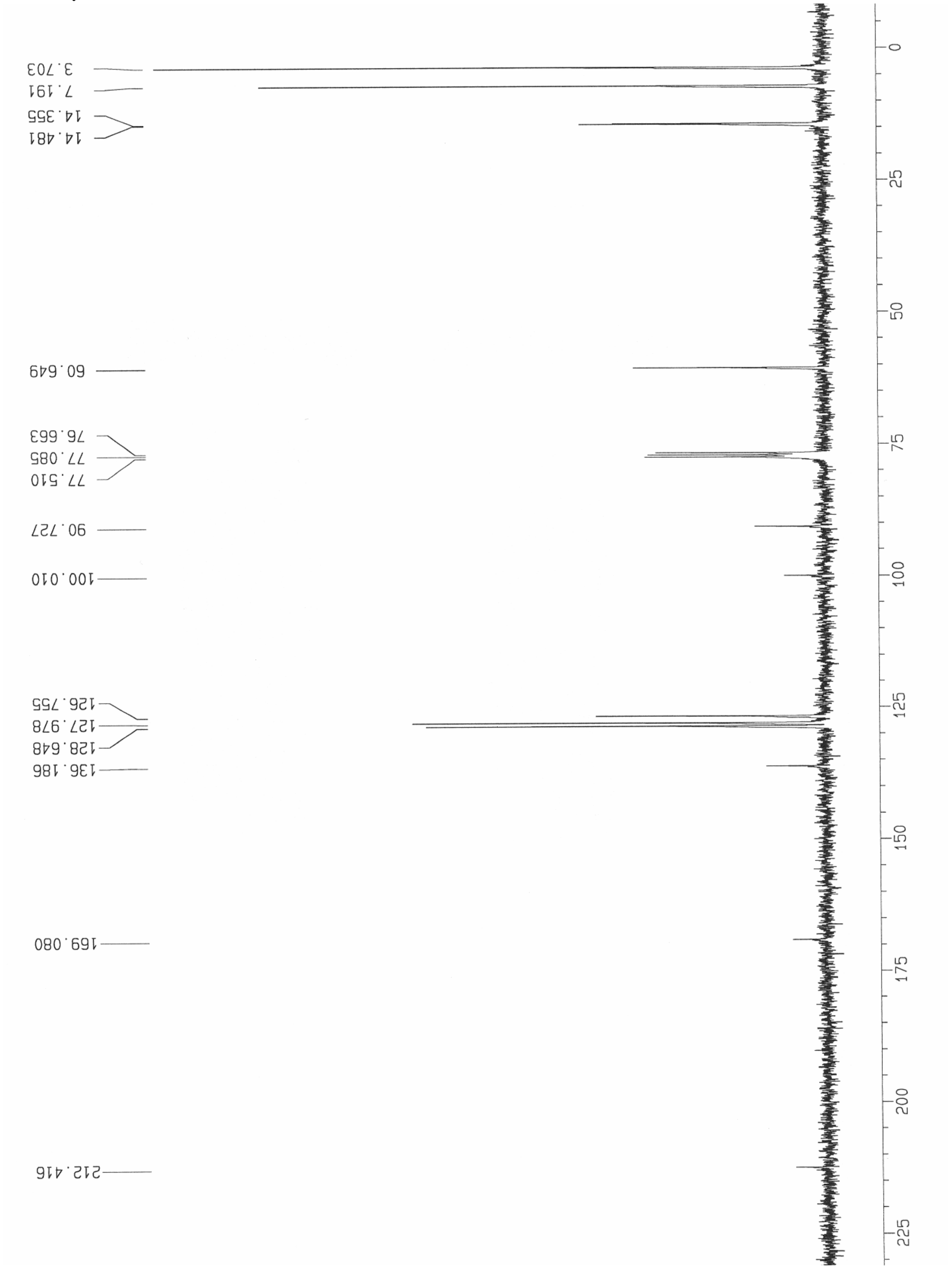
3.703
7.191
14.355
14.481
60.649
76.663
77.085
77.510
90.727
100.010
126.755
127.978
128.648
136.186
169.080
212.416
0
25
50
75
100
125
150
175
200
225

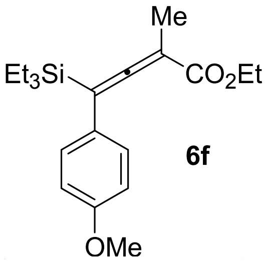

6f

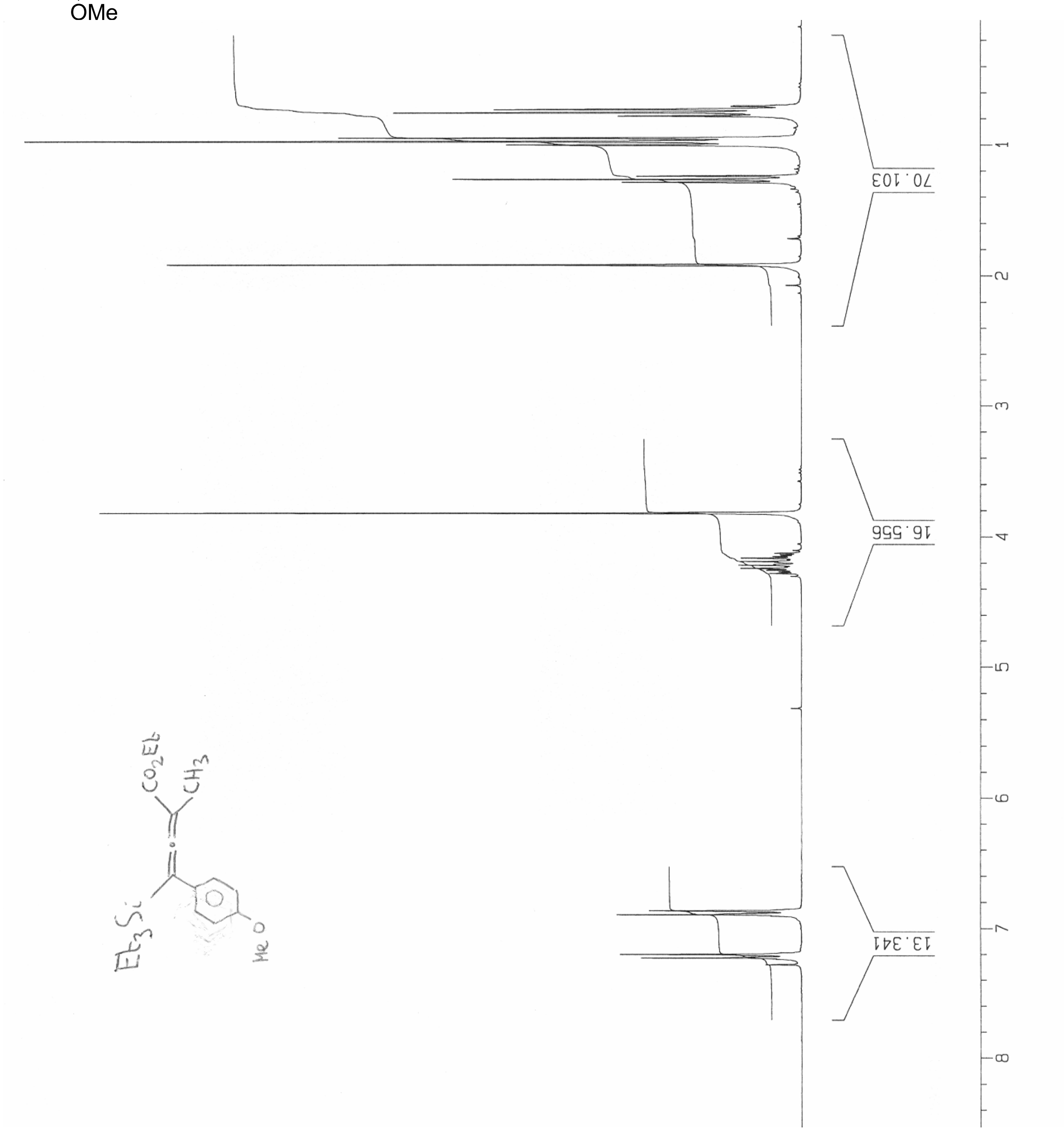

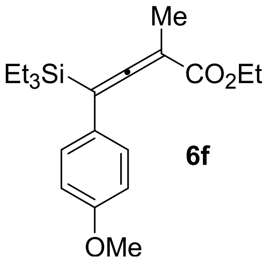

Me
Et3Si
CO2Et
OMe

6f

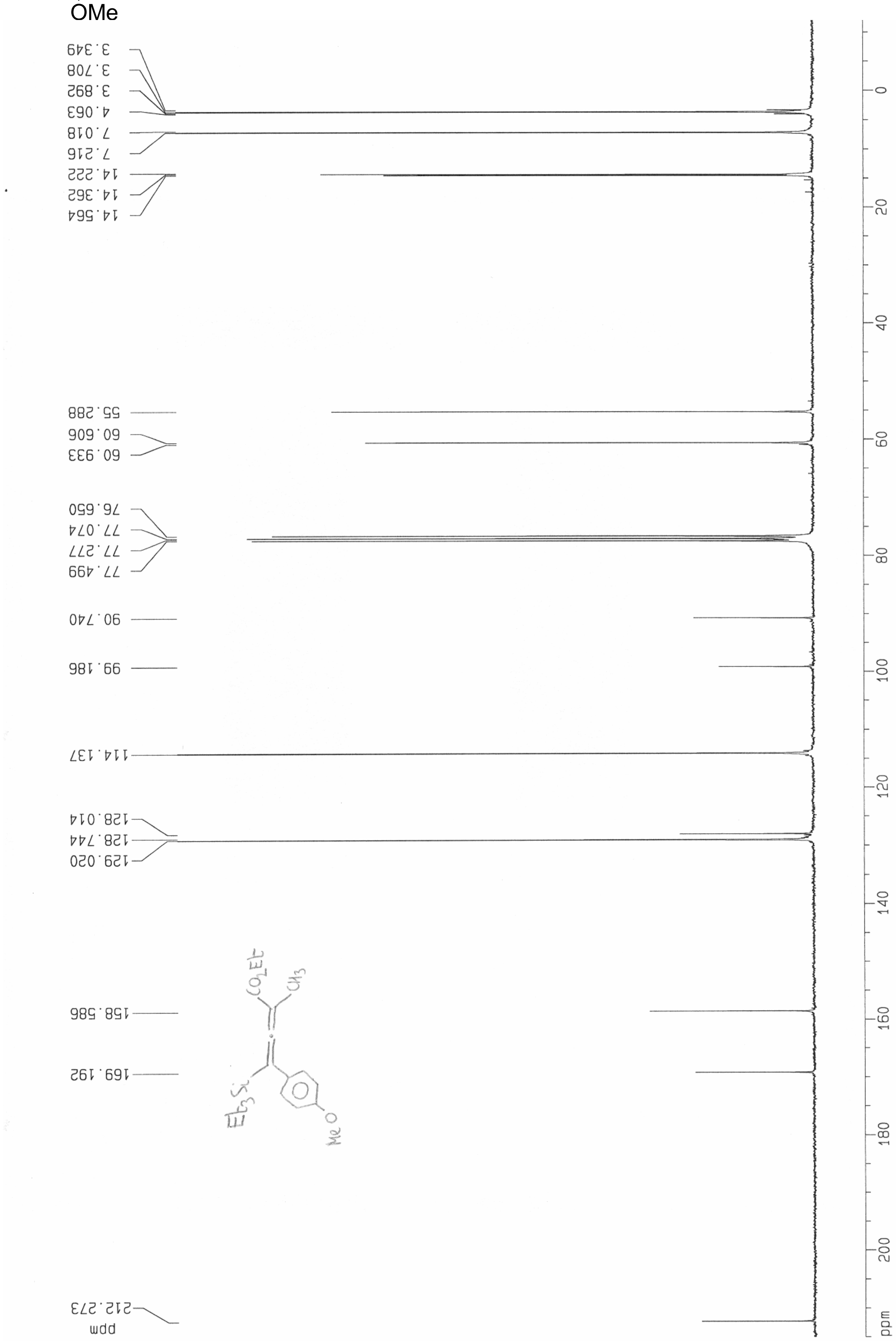

3.349
3.708
3.892
4.063
7.018
7.216
14.222
14.362
14.564
55.288
60.606
60.933
76.650
77.074
77.277
77.499
90.740
99.186
114.137
128.014
128.744
129.020
158.586
169.192
212.273
ppm
0
20
40
60
80
100
120
140
160
180
200
ppm

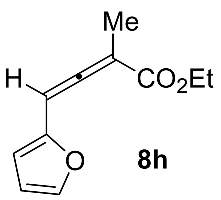

8h

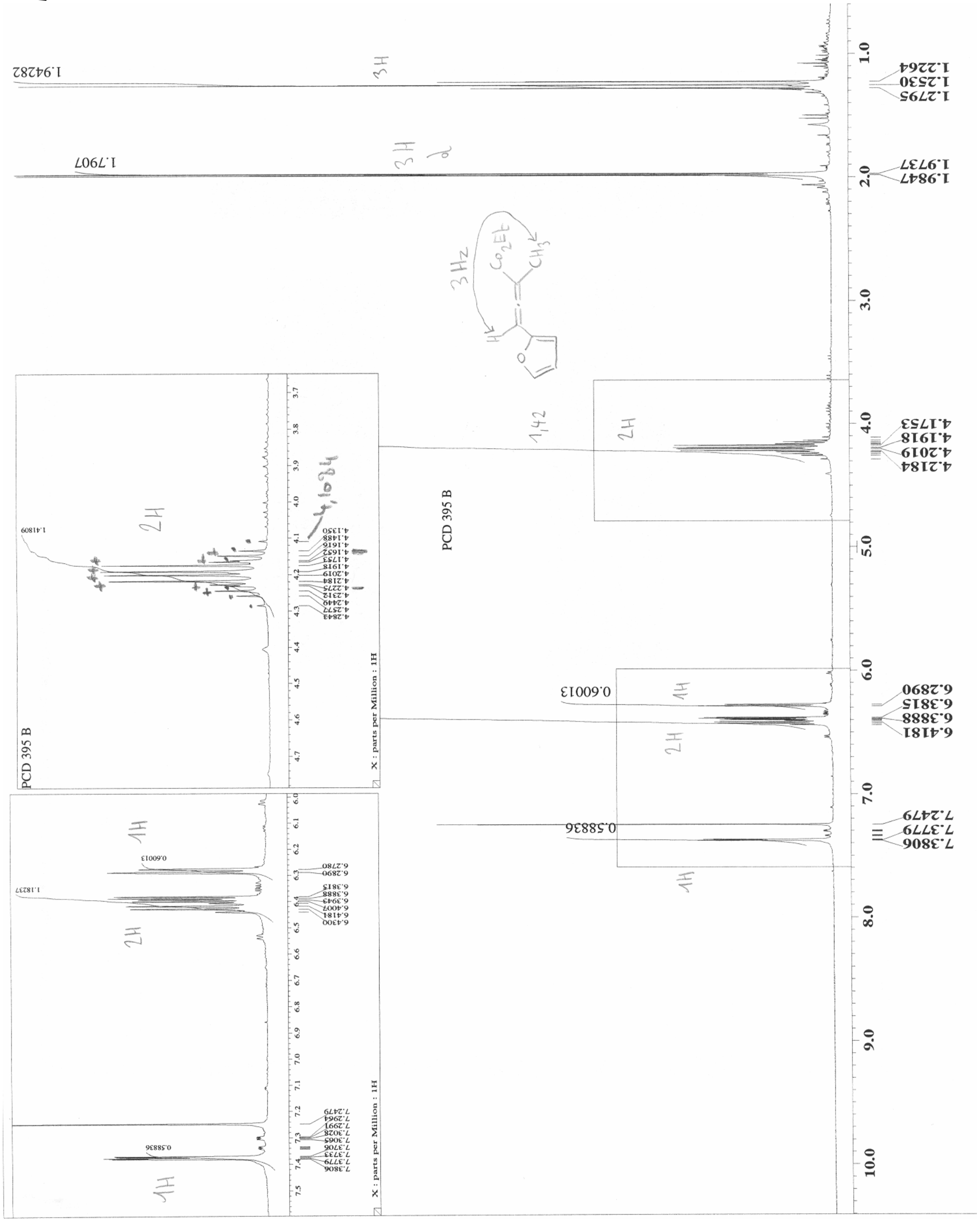

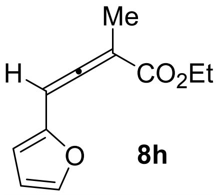
Me
H
CO2Et
O

8h

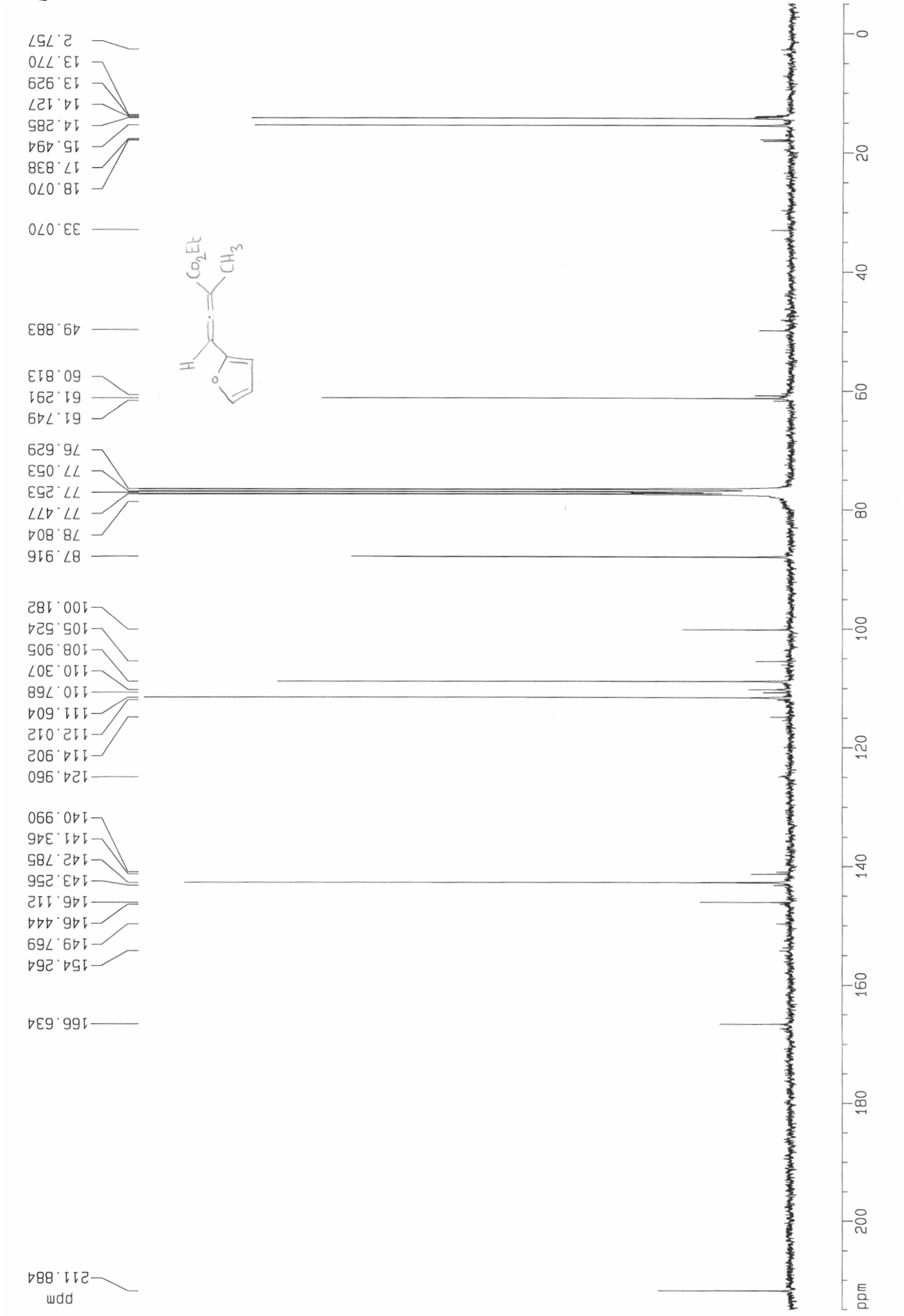
2.757
13.770
13.929
14.127
14.285
15.494
17.838
18.070
33.070
49.883
60.813
61.291
61.749
76.629
77.053
77.253
77.477
78.804
87.916
100.182
105.524
108.905
110.307
110.768
111.604
112.012
114.902
124.960
140.990
141.346
142.785
143.256
146.112
146.444
149.769
154.264
166.634
211.884
ppm
0
20
40
60
80
100
120
140
160
180
200
ppm

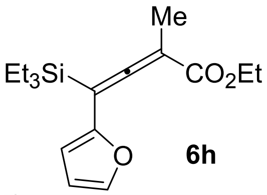

6h

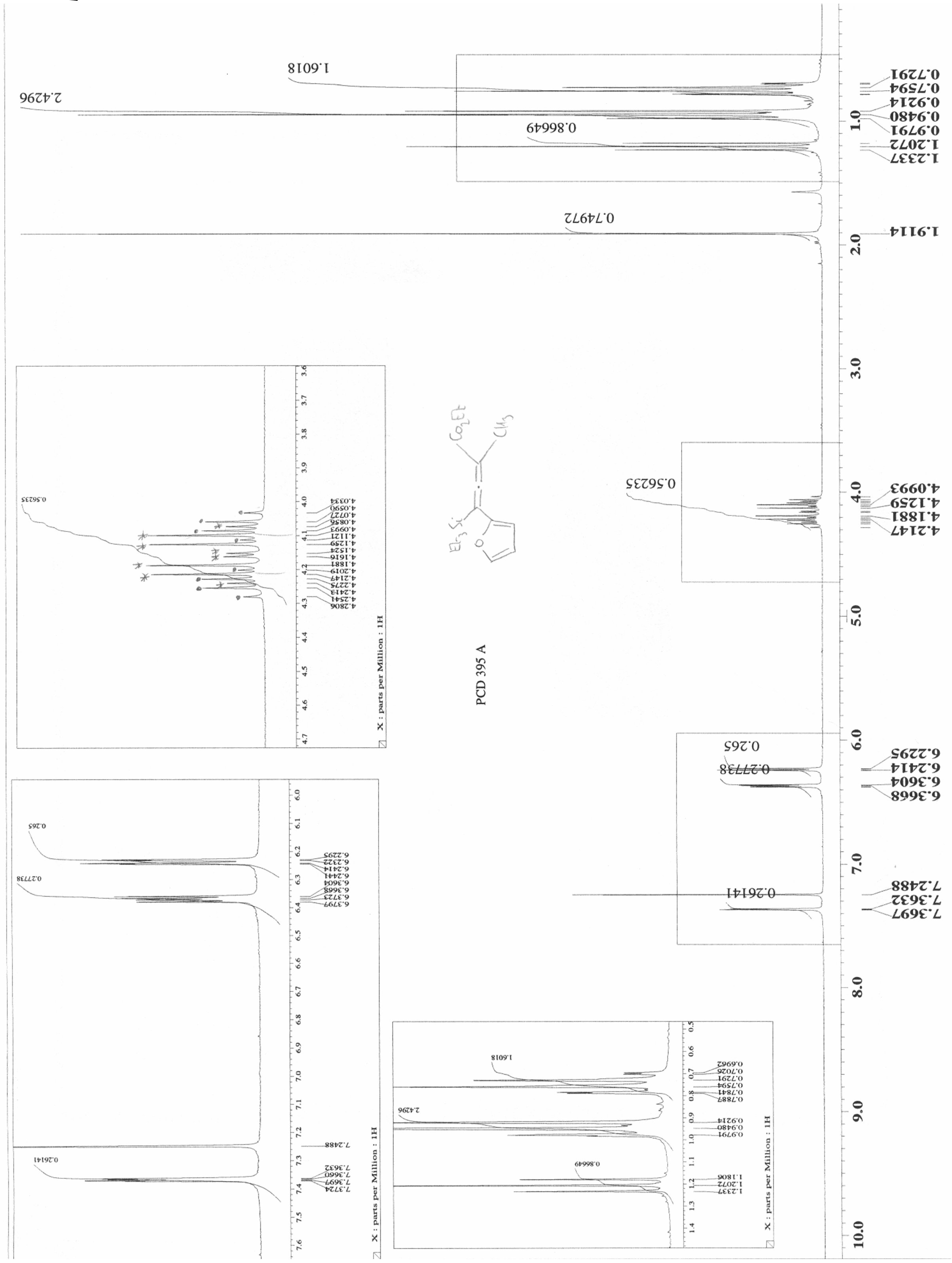

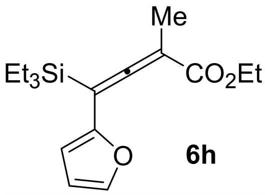
Me
Et3Si
CO2Et

6h

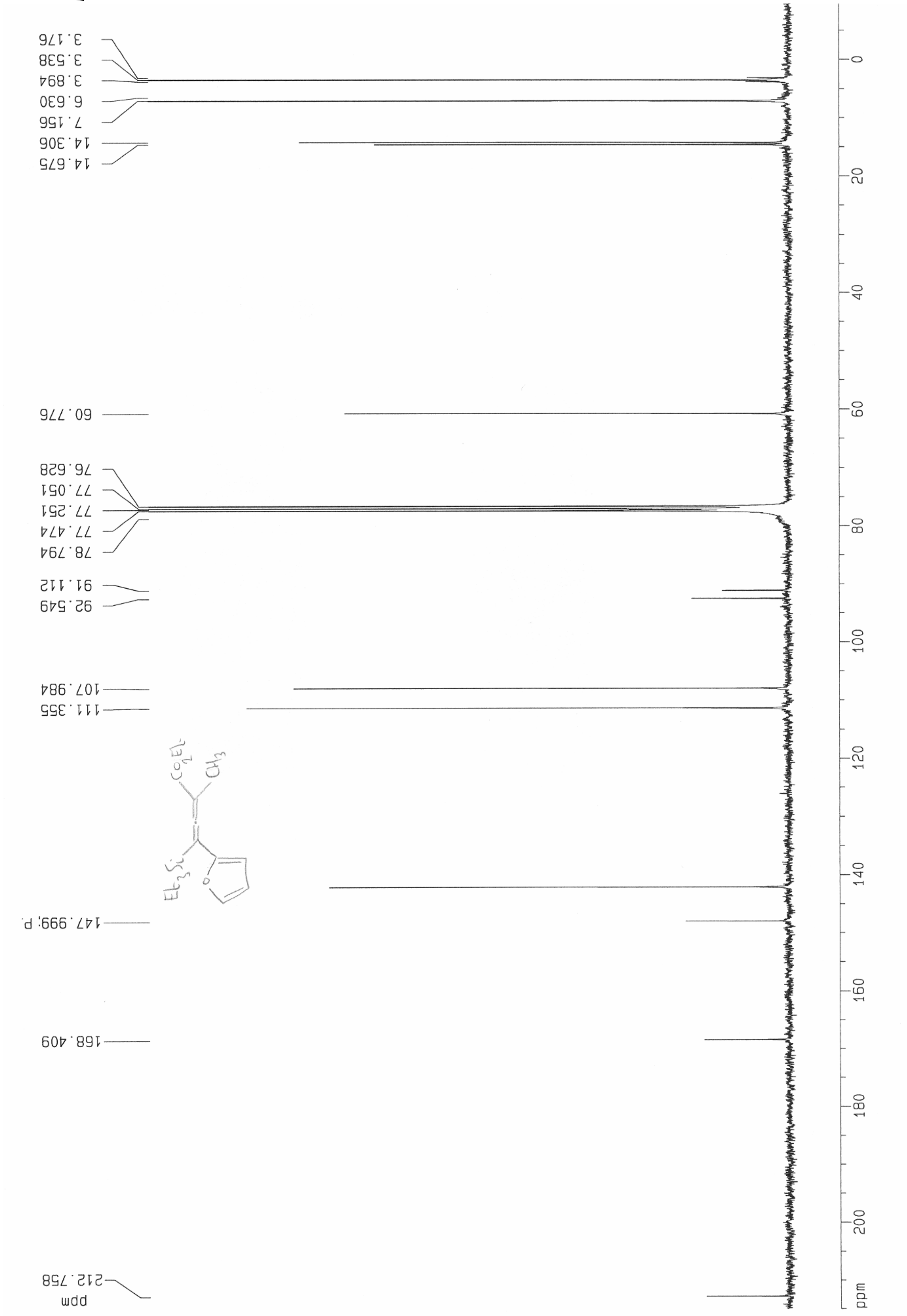
3.176
3.538
3.894
6.630
7.156
14.306
14.675
60.776
76.628
77.051
77.251
77.474
78.794
91.112
92.549
107.984
111.355
147.999
168.409
212.758
ppm
0
20
40
60
80
100
120
140
160
180
200
ppm

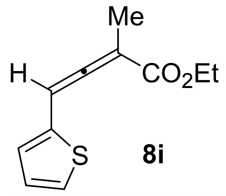
Me
H
CO2Et
S

8i

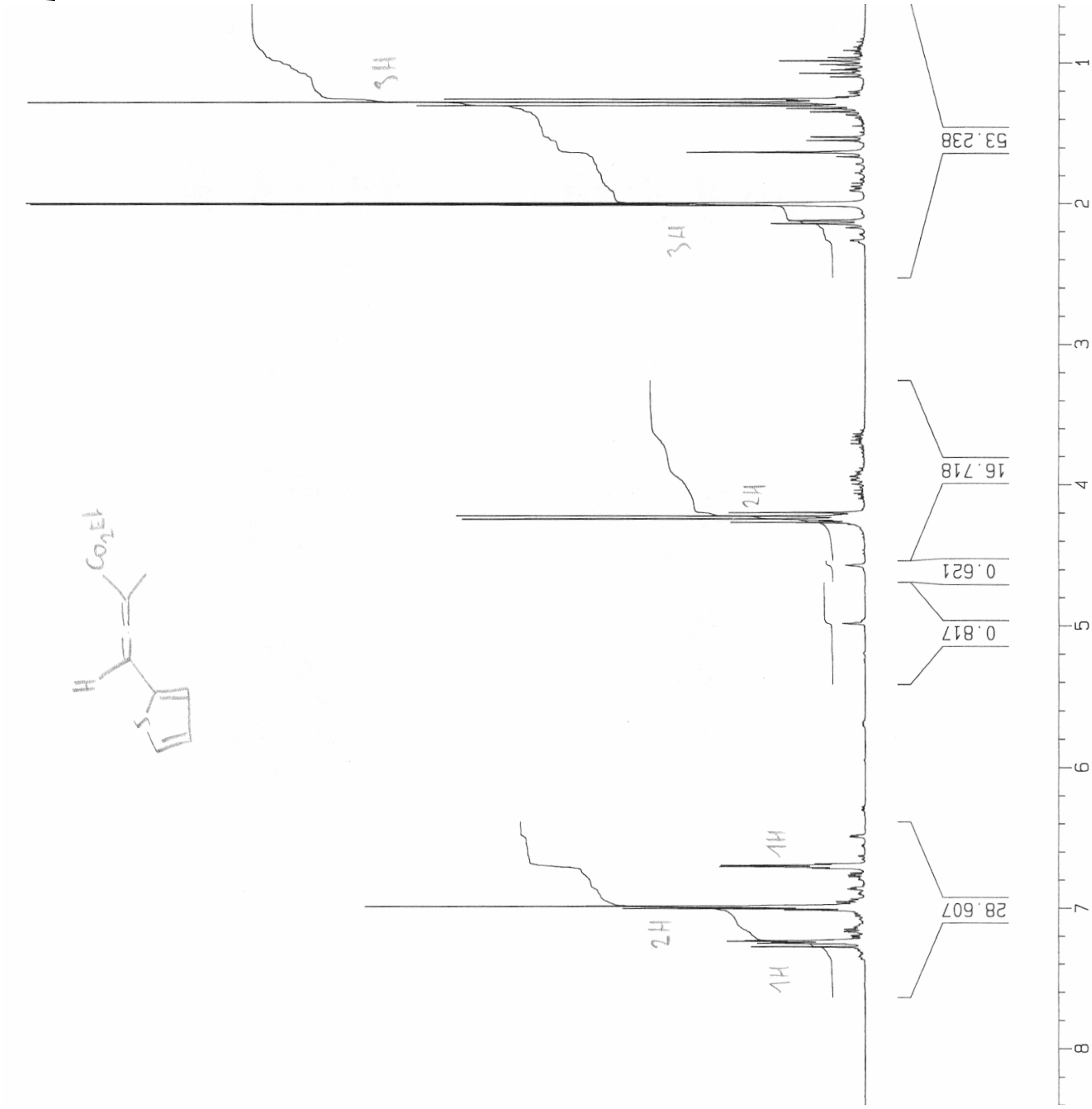
53.238
16.718
0.621
0.817
28.607
1
2
3
4
5
6
7
8
3H
3H
2H
1H
2H
1H

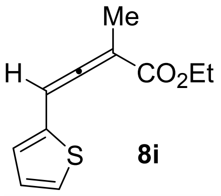
Me
H
CO2Et
S

8i

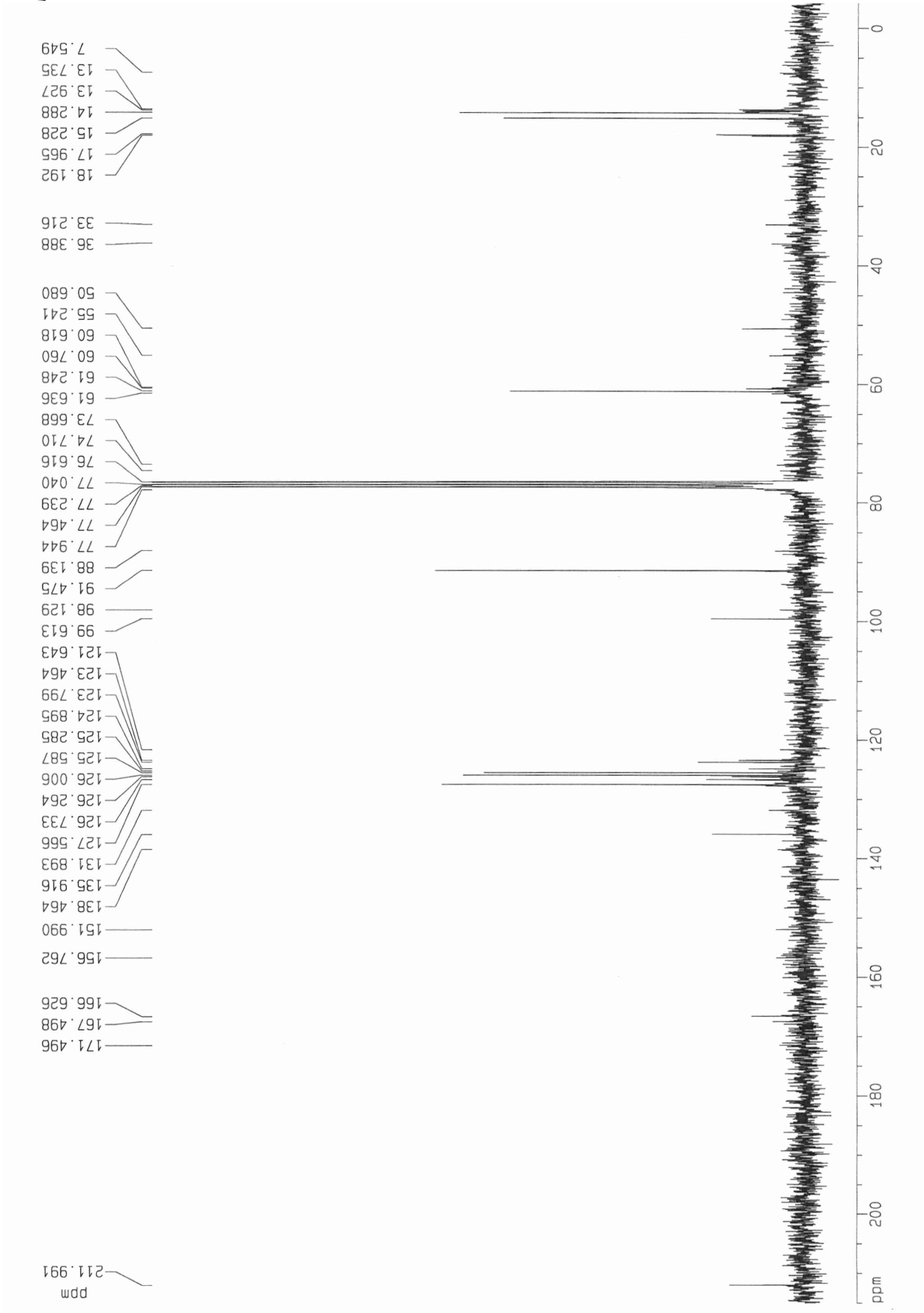
7.549
13.735
13.927
14.288
15.228
17.965
18.192
33.216
36.388
50.680
55.241
60.618
60.760
61.248
61.636
73.668
74.710
76.616
77.040
77.239
77.464
77.944
88.139
91.475
98.129
99.613
121.643
123.464
123.799
124.895
125.285
125.587
126.006
126.264
126.733
127.566
131.893
135.916
138.464
151.990
156.762
166.626
167.498
171.496
211.991
ppm
0
20
40
60
80
100
120
140
160
180
200
ppm

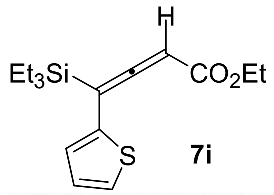
H
Et3Si
CO2Et
S

7i

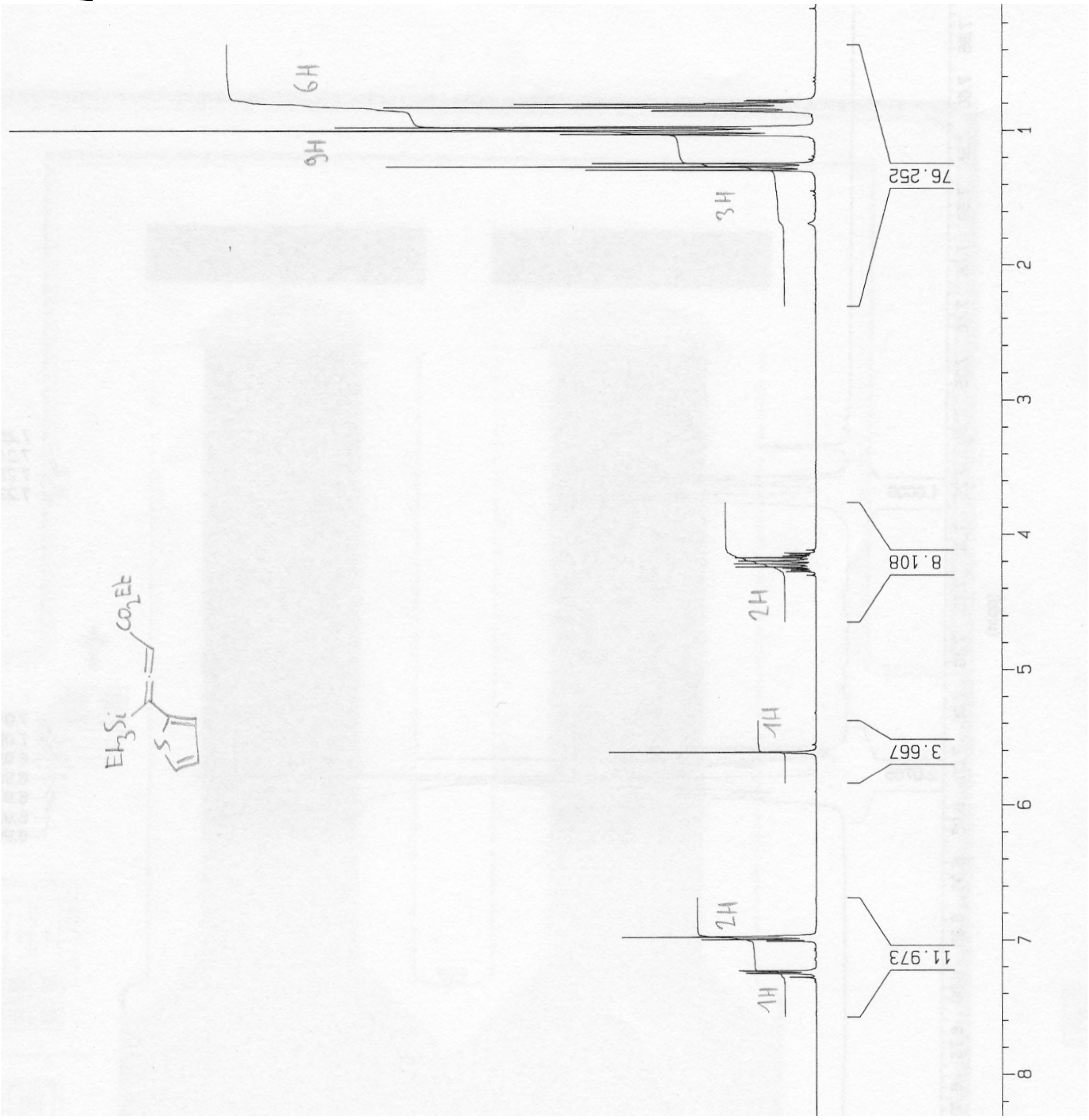
6H
9H
3H
2H
1H
2H
1H
76.252
8.108
3.667
11.973
1
2
3
4
5
6
7
8

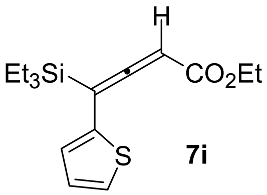
H
Et3Si
CO2Et
S

7i

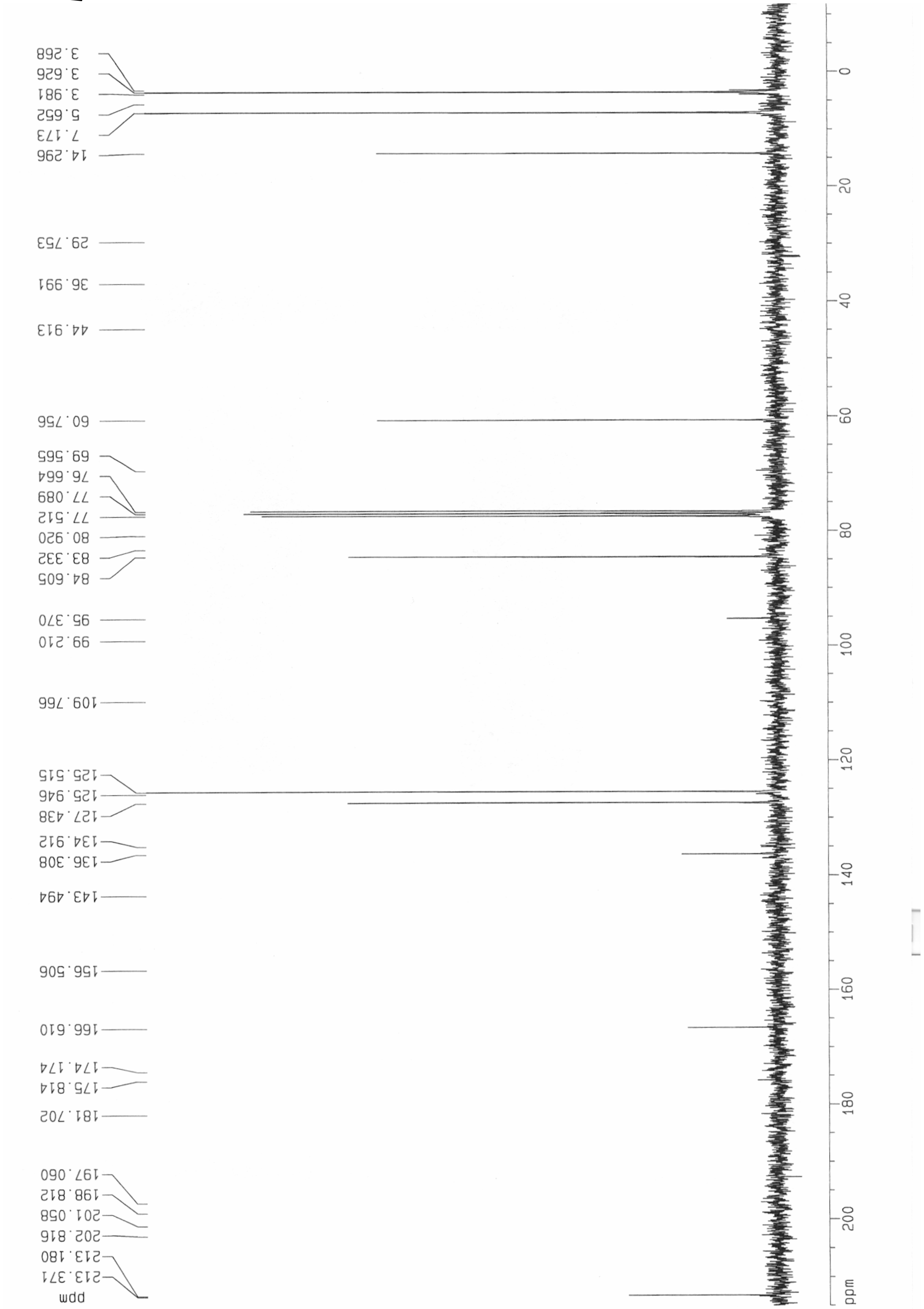
3.268
3.626
3.981
5.652
7.173
14.296
29.753
36.991
44.913
60.756
69.565
76.664
77.089
77.512
80.920
83.332
84.605
95.370
99.210
109.766
125.515
125.946
127.438
134.912
136.308
143.494
156.506
166.610
174.174
175.814
181.702
197.060
198.812
201.058
202.816
213.180
213.371
ppm
0
20
40
60
80
100
120
140
160
180
200
ppm

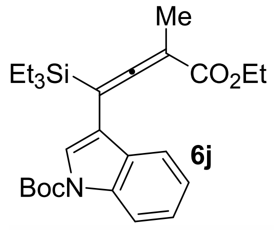

6j

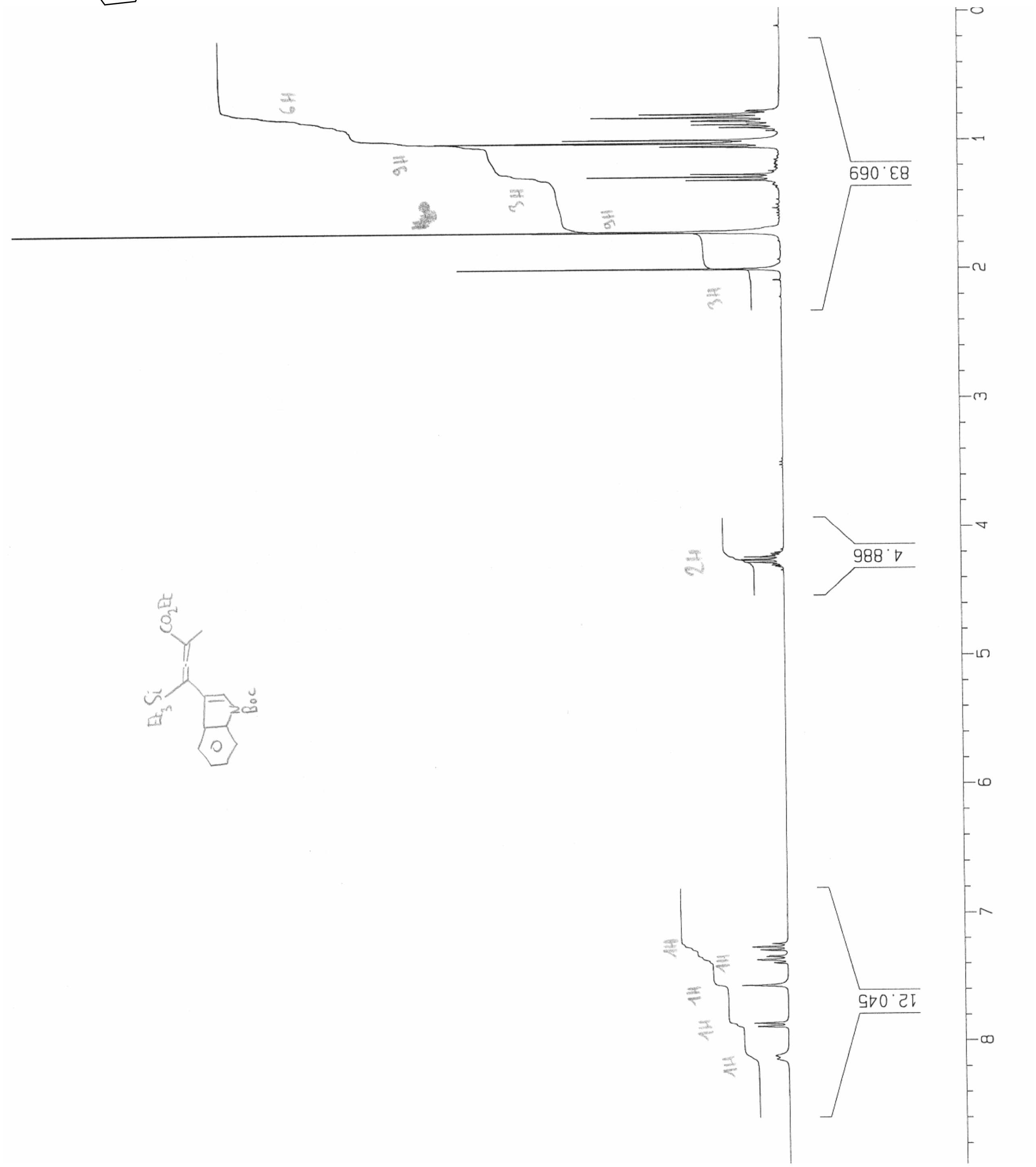

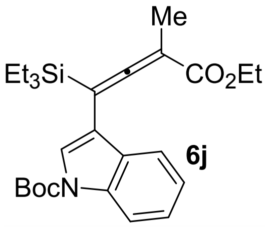
Me
Et3Si
CO2Et
BocN

6j

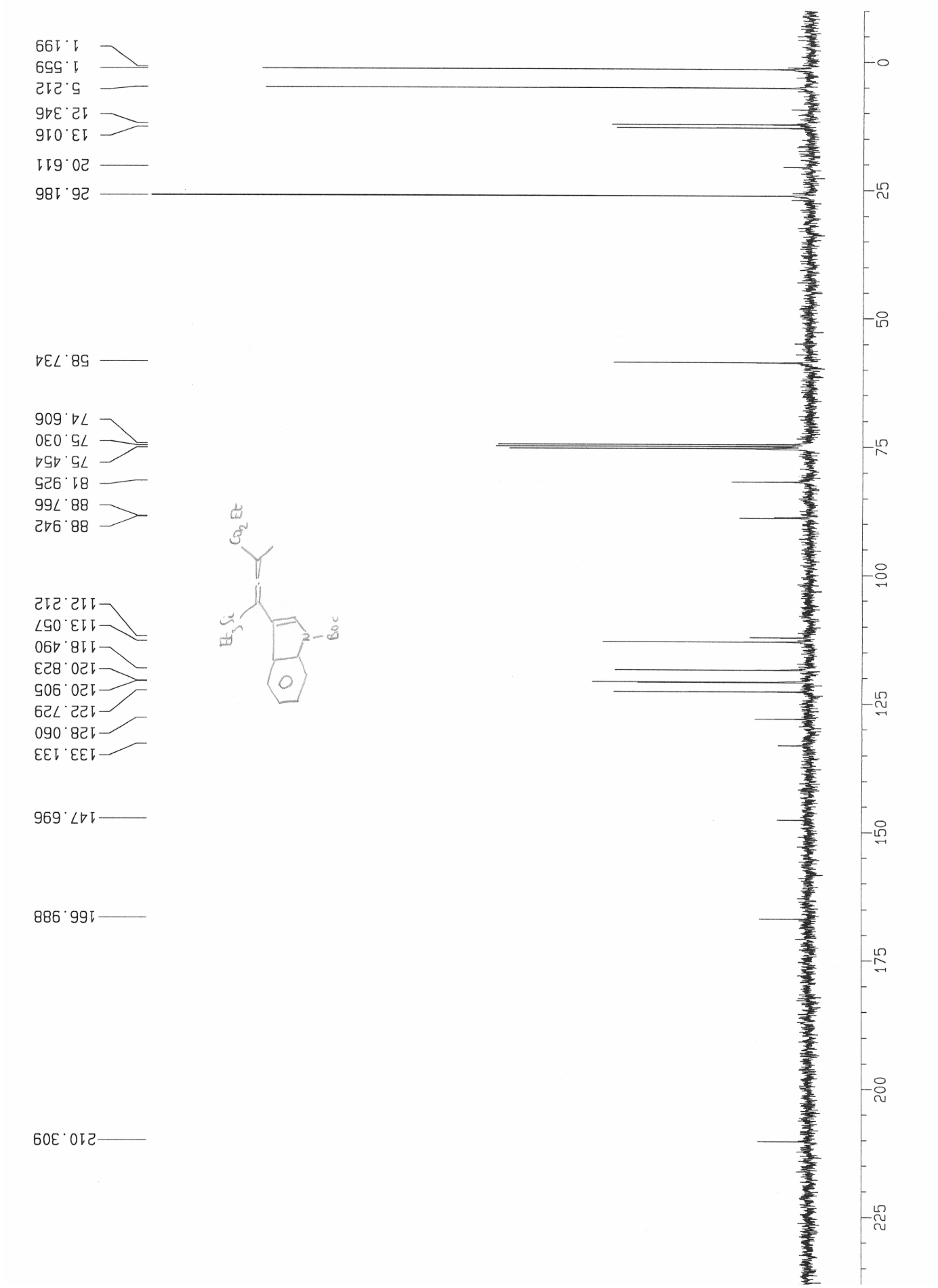
1.199
1.559
5.212
12.346
13.016
20.611
26.186
58.734
74.606
75.030
75.454
81.925
88.766
88.942
112.212
113.057
118.490
120.823
120.905
122.729
128.060
133.133
147.696
166.988
210.309
0
25
50
75
100
125
150
175
200
225
