# Supplementary material for: Synthesis of highly substituted allenylsilanes by alkylidenation of silylketenes
Source: Beilstein J Org Chem. 2005 Aug 26;1:5. doi: 10.1186/1860-5397-1-5 (PMC1399453; doi:10.1186/1860-5397-1-5)
Supplement: File 3 — NMR spectra [file Beilstein_J_Org_Chem-01-05-s003.pdf]

Synthesis of Highly Substituted Allenylsilanes by Alkylidenation of Silylketenes

Stephen P. Marsden* and Pascal C. Ducept

Supplementary information part 2

^{1}H and ^{13}C nmr spectra of allenylsilanes **14-19**.

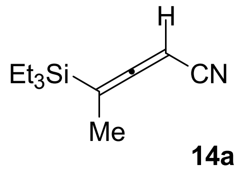

14a

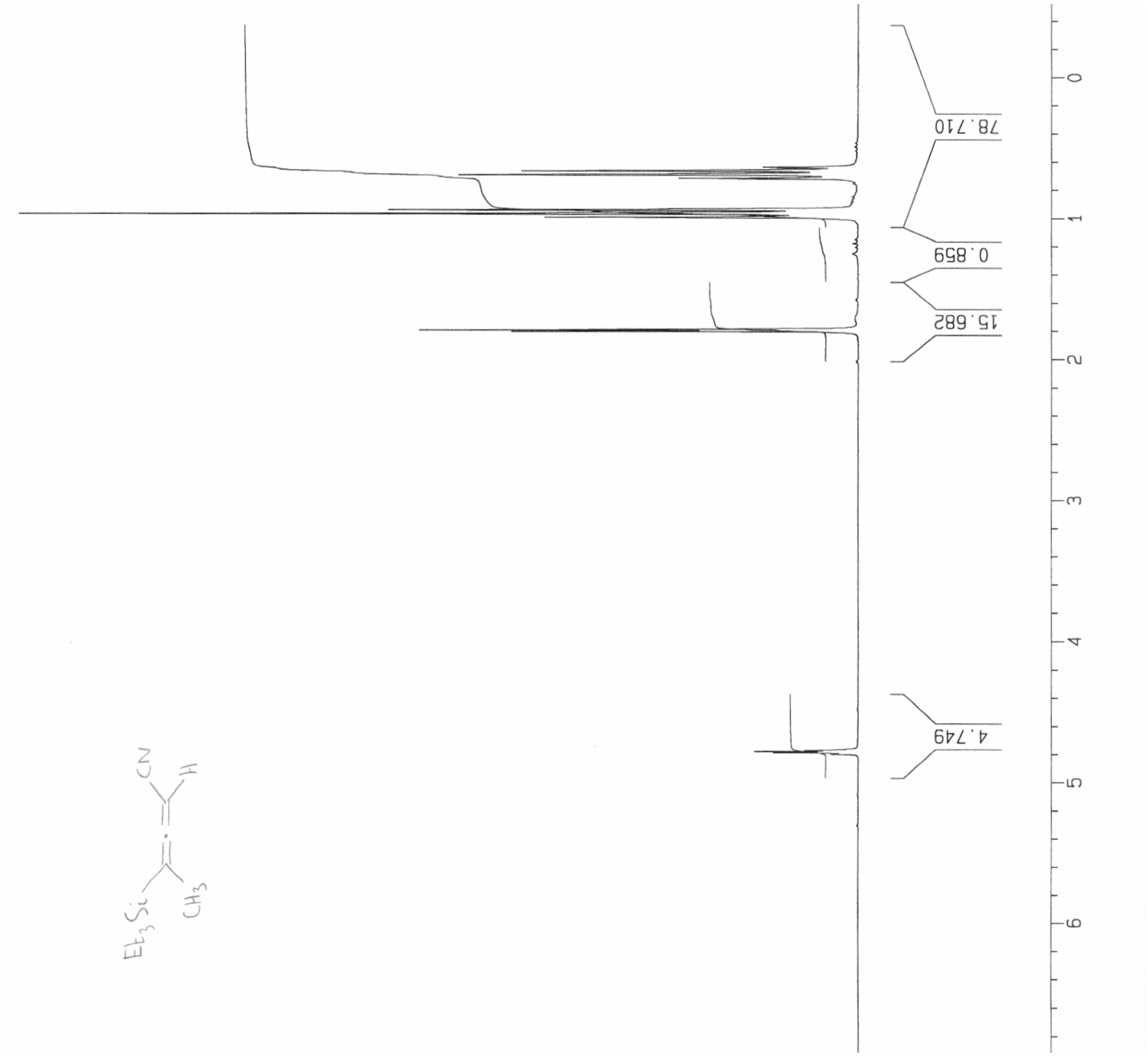

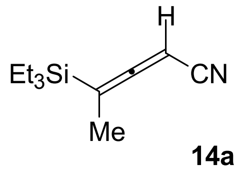

H
Et3Si
CN
Me

14a

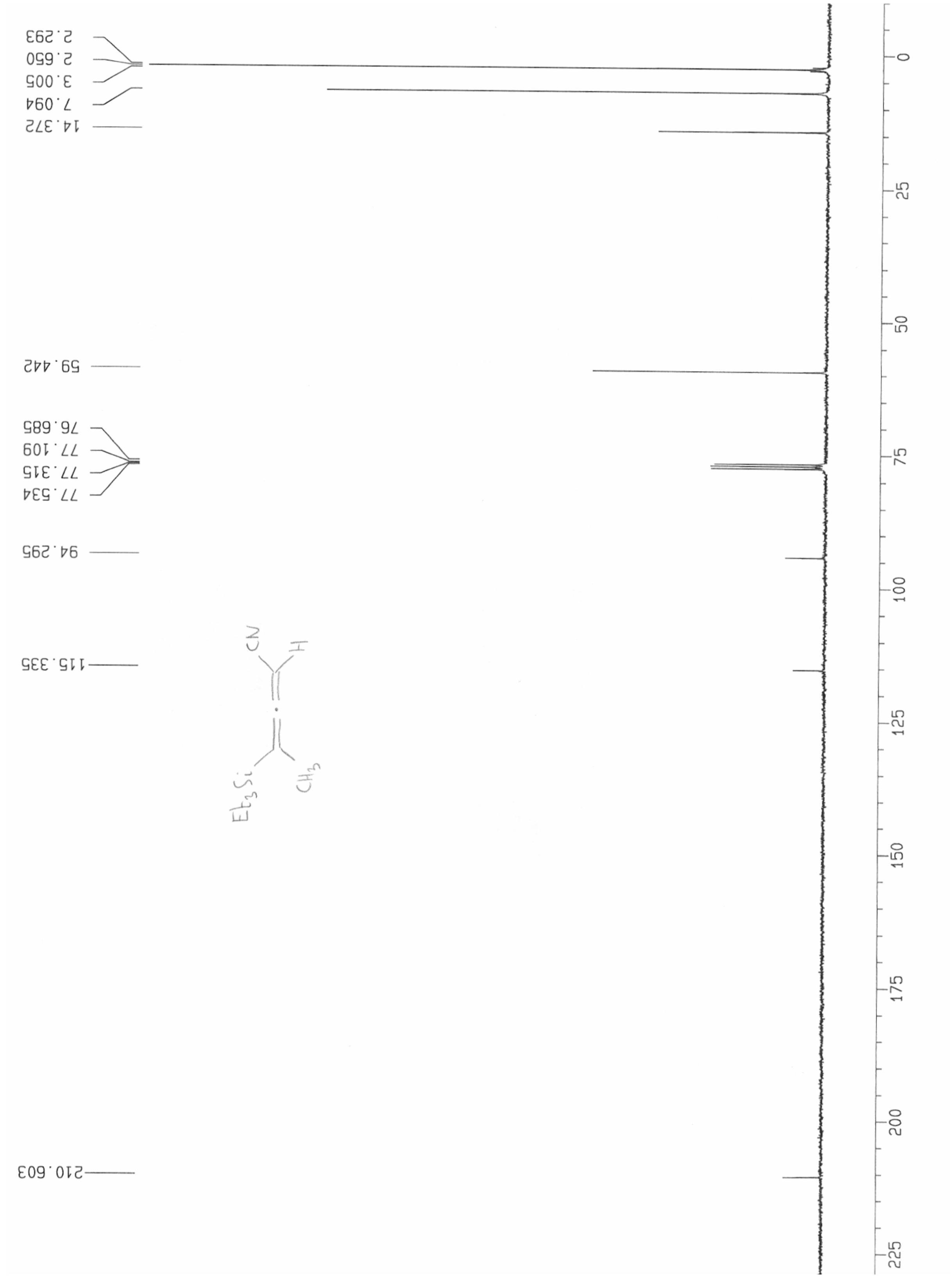

2.293
2.650
3.005
7.094
14.372
59.442
76.685
77.109
77.315
77.534
94.295
115.335
210.603
0
25
50
75
100
125
150
175
200
225

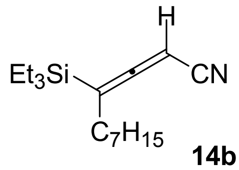

14b

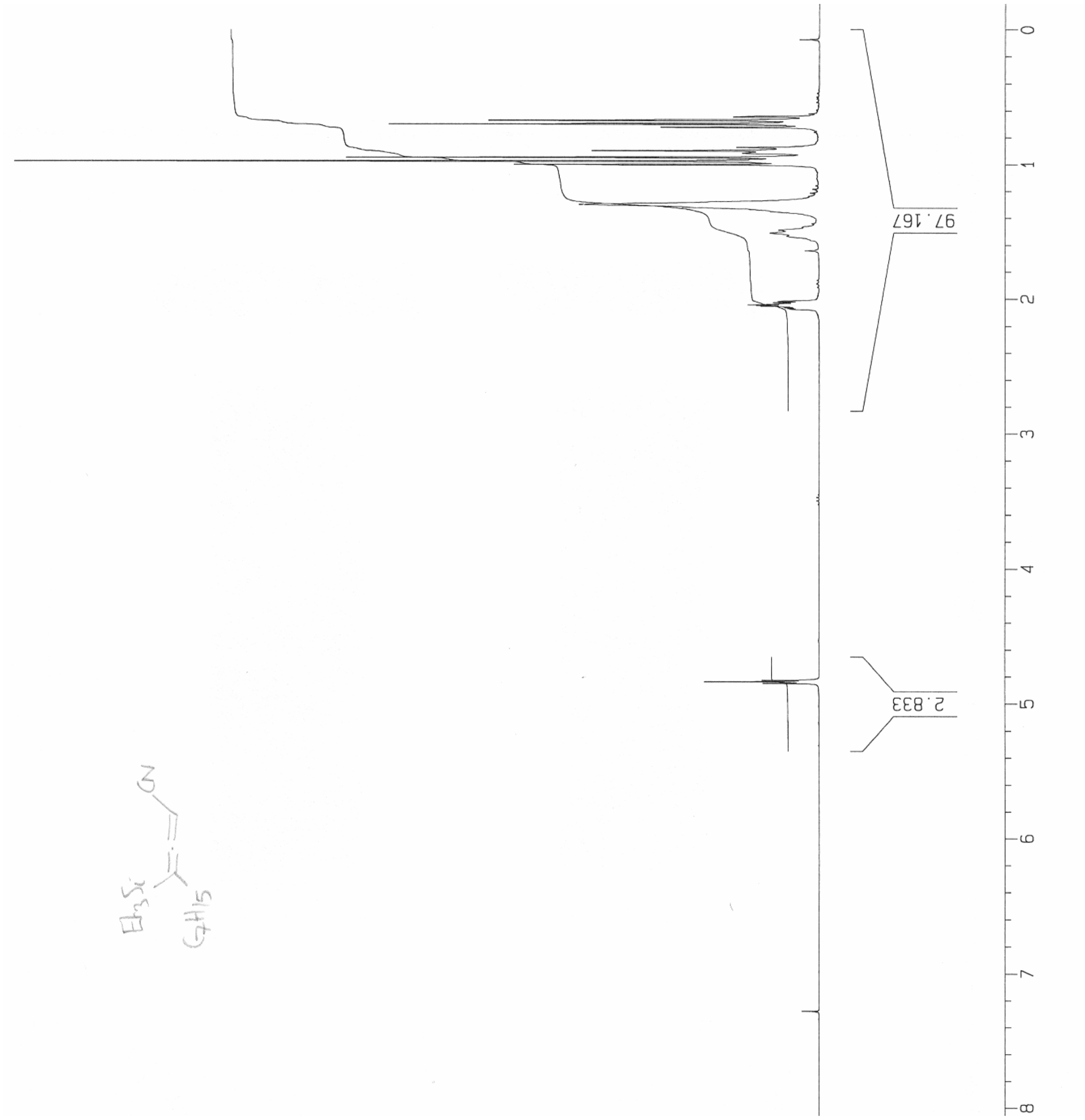

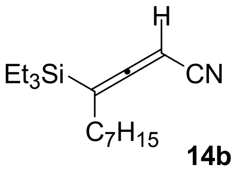
Et3Si
H
CN
C7H15

14b

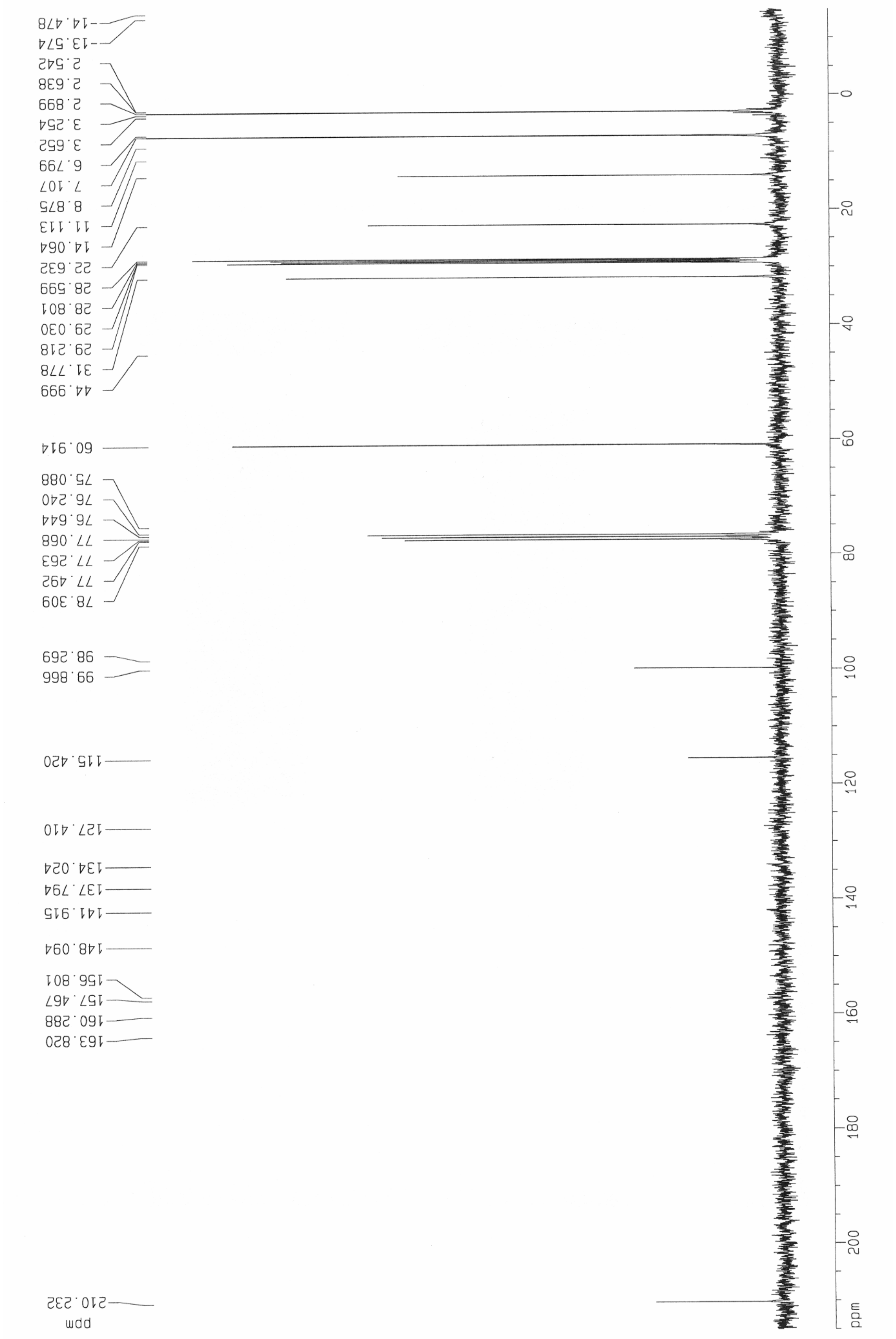
14.478
13.574
2.542
2.638
2.899
3.254
3.652
6.799
7.107
8.875
11.113
14.064
22.632
28.599
28.801
29.030
29.218
31.778
44.999
60.914
75.088
76.240
76.644
77.068
77.263
77.492
78.309
98.269
99.866
115.420
127.410
134.024
137.794
141.915
148.094
156.801
157.467
160.288
163.820
210.232
ppm
0
20
40
60
80
100
120
140
160
180
200
ppm

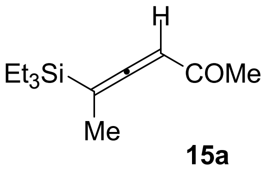

H
Et3Si
COMe
Me

15a

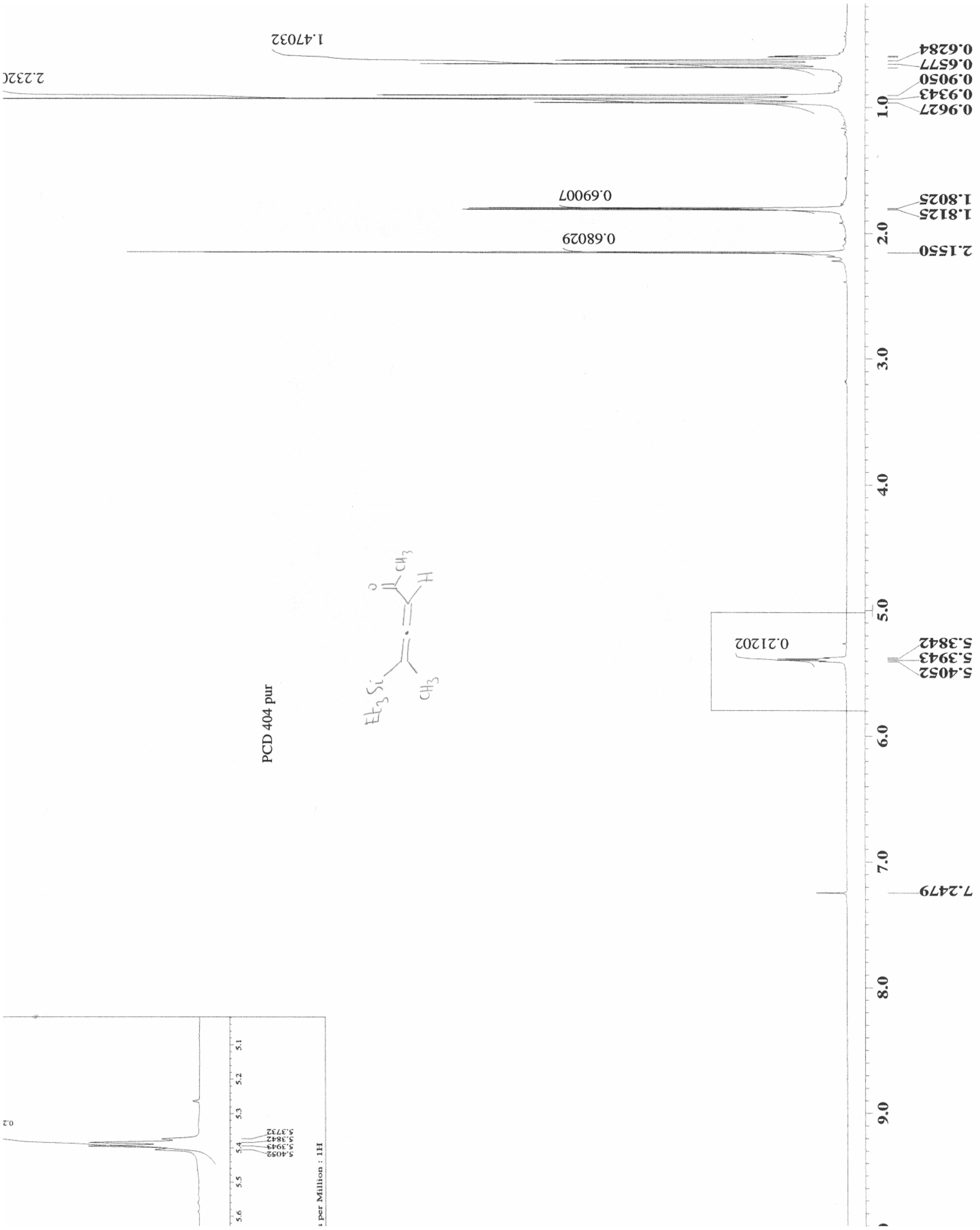

PCD 404 pur
0.6284
0.6577
0.9050
0.9343
0.9627
1.8025
1.8125
2.1550
5.3842
5.3943
5.4052
7.2479
1.47032
0.69007
0.68029
0.21202

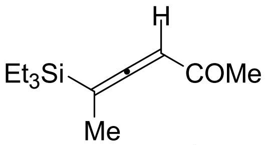
H
Et3Si
COMe
Me

15a

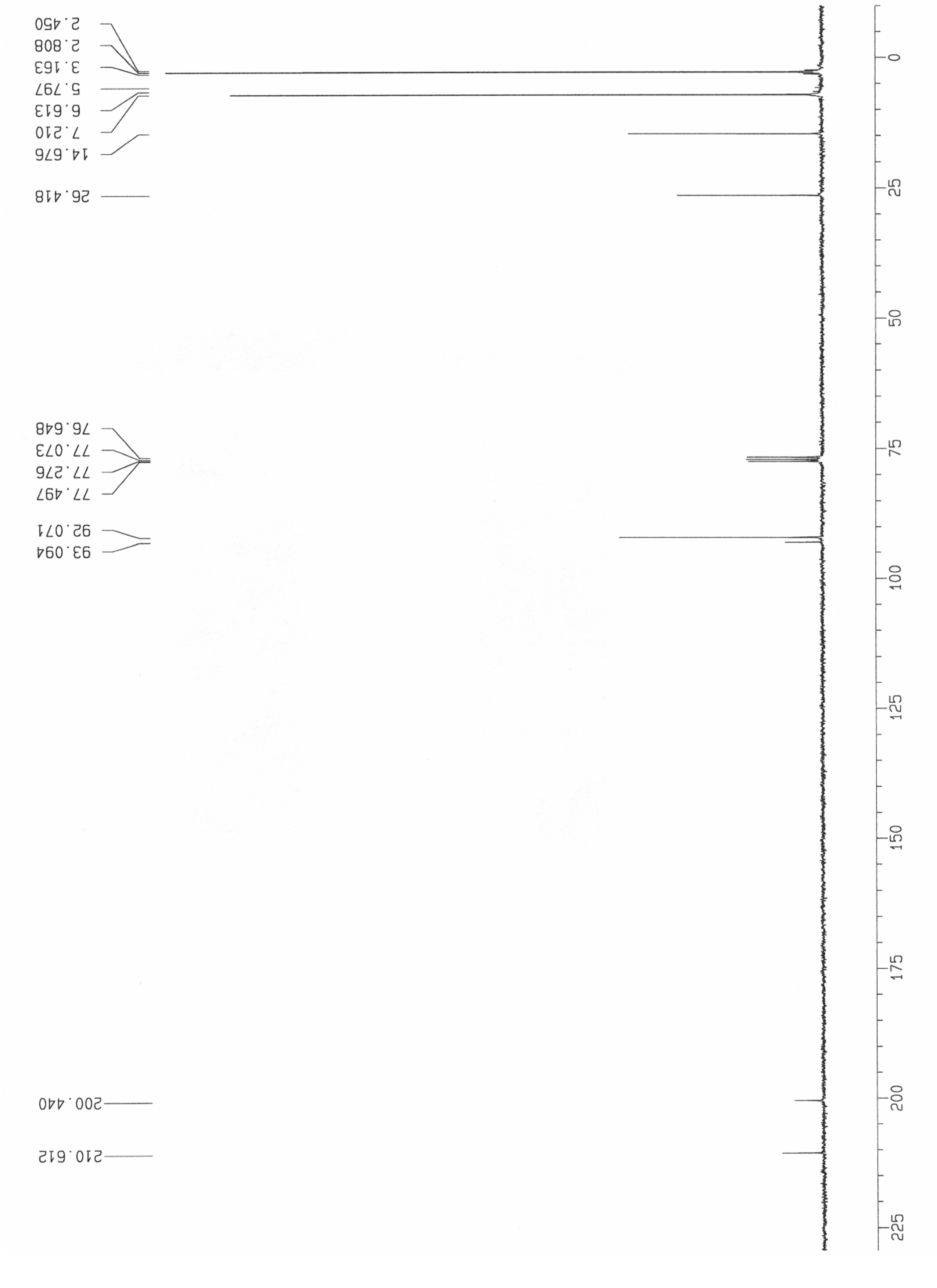
2.450
2.808
3.163
5.797
6.613
7.210
14.676
26.418
76.648
77.073
77.276
77.497
92.071
93.094
200.440
210.612
0
25
50
75
100
125
150
175
200
225

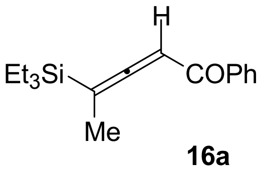

16a

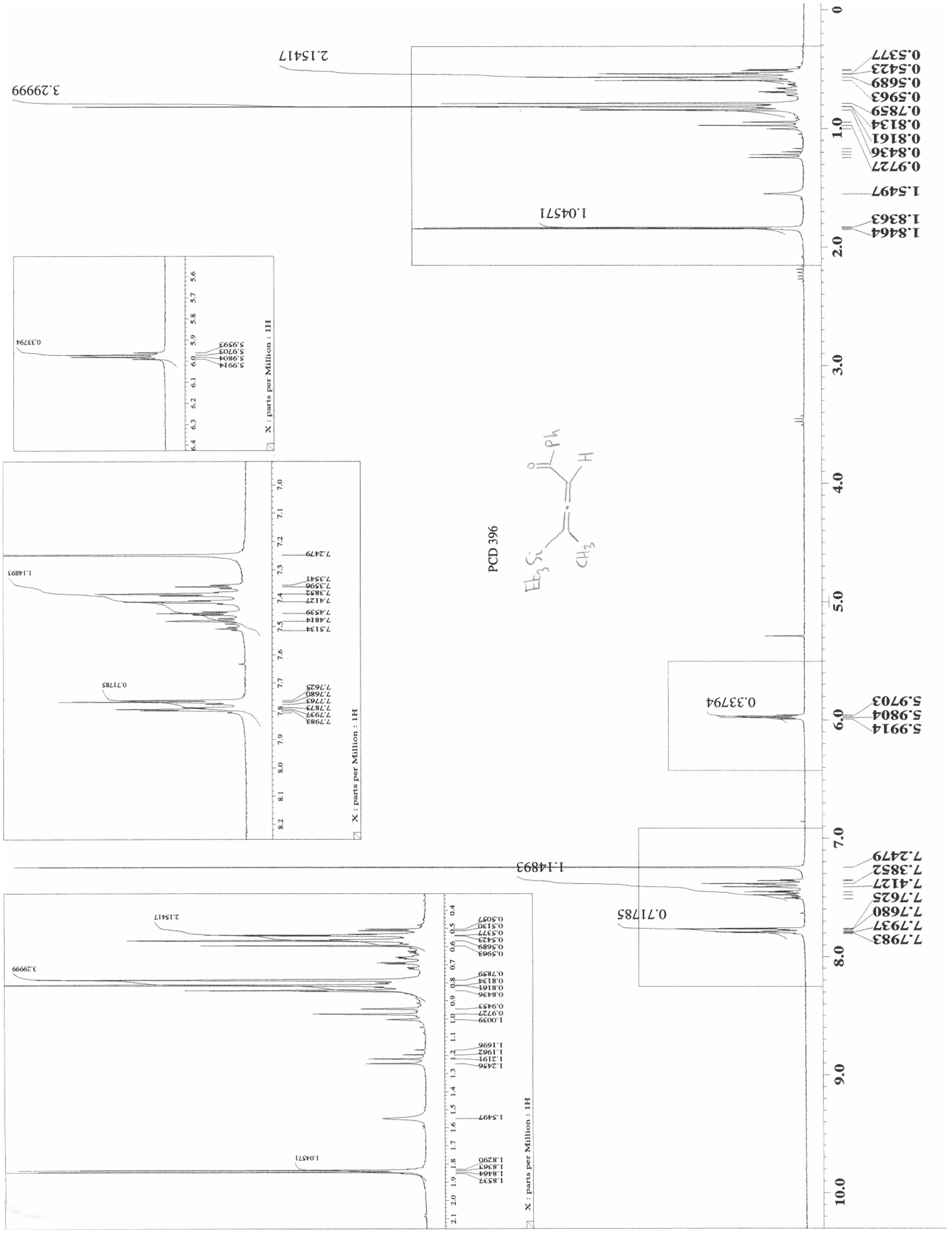

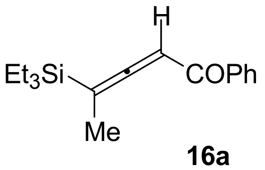
H
Et3Si
COPh
Me

16a

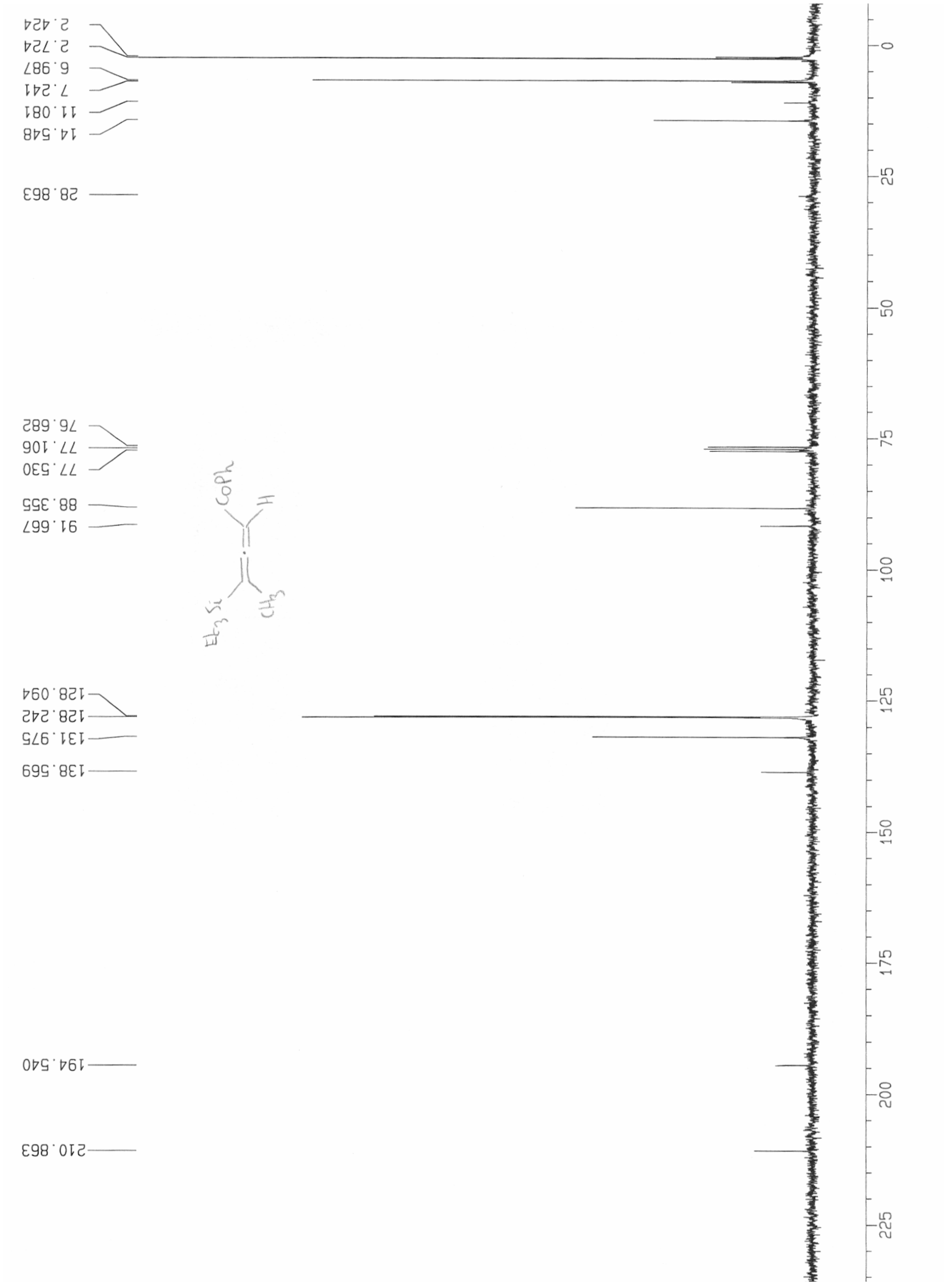
2.424
2.724
6.987
7.241
11.081
14.548
28.863
76.682
77.106
77.530
88.355
91.667
128.094
128.242
131.975
138.569
194.540
210.863
0
25
50
75
100
125
150
175
200
225

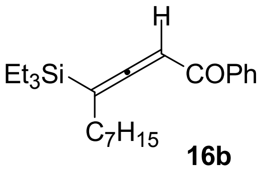
H
Et3Si
COPh
C7H15

16b

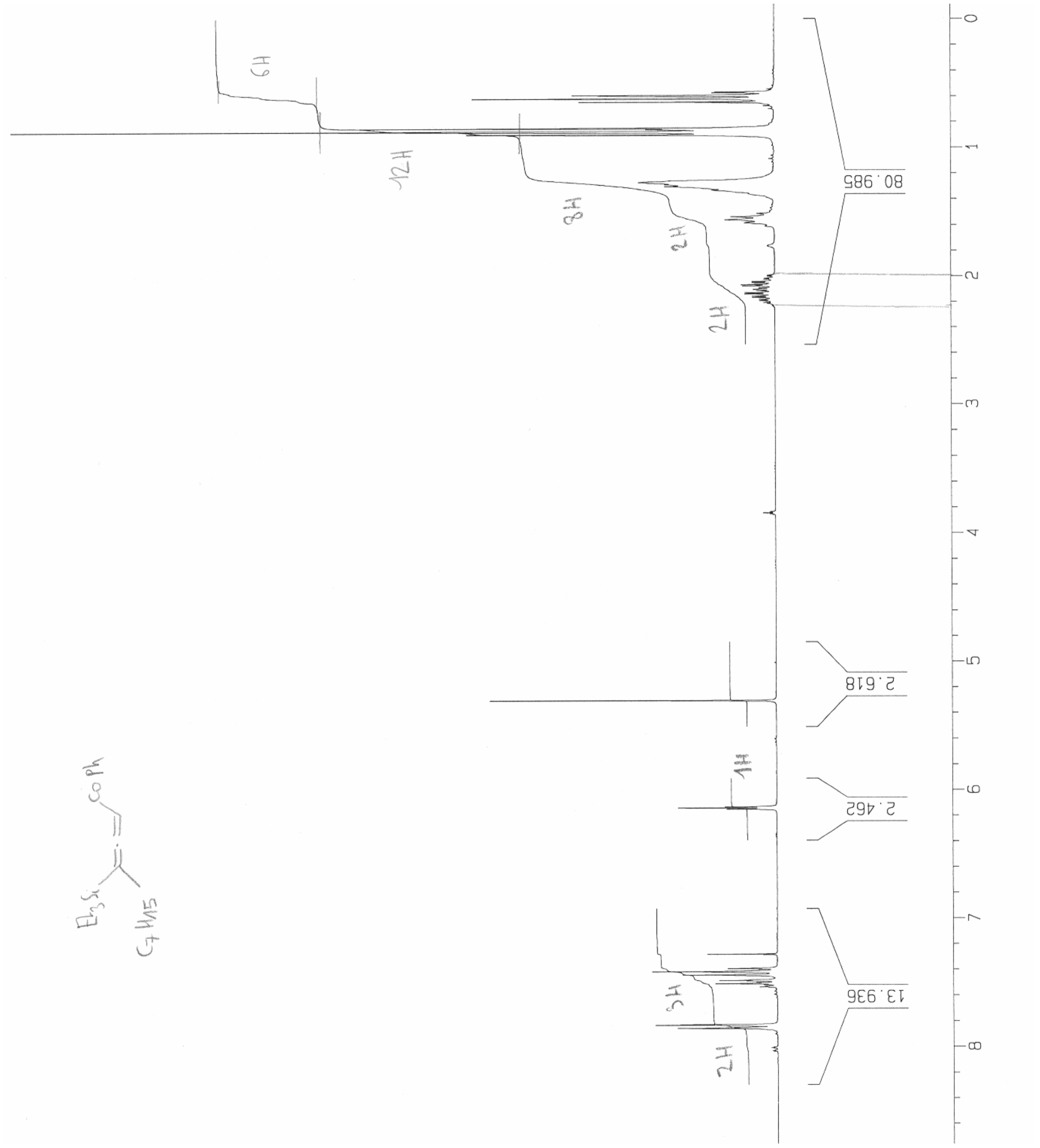
80.985
2.618
2.462
13.936
6H
12H
8H
2H
2H
1H
3H
2H
0
1
2
3
4
5
6
7
8

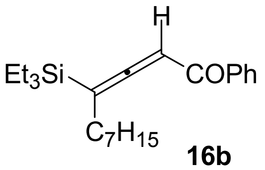
H
Et3Si
COPh
C7H15

16b

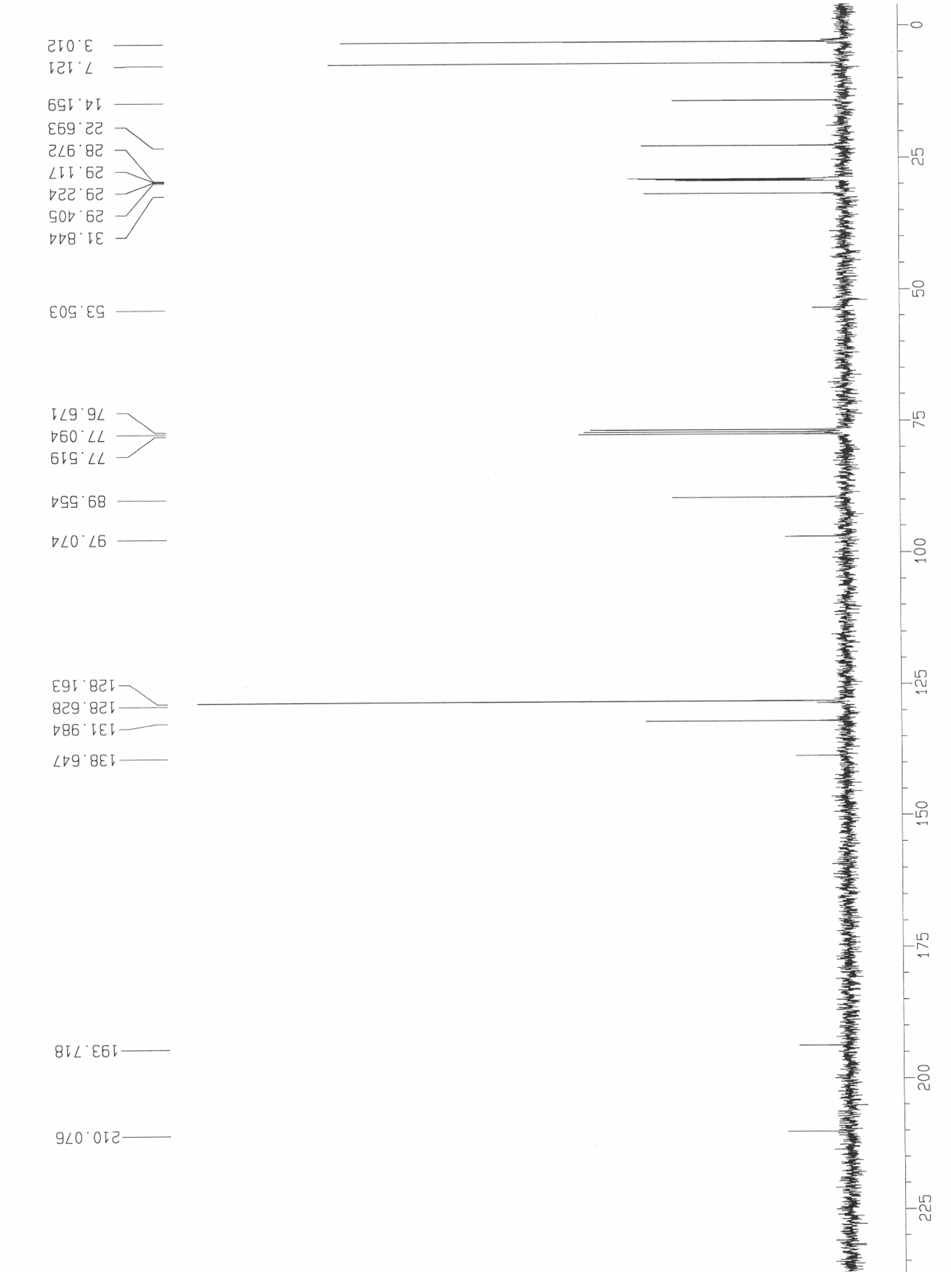
3.012
7.121
14.159
22.693
28.972
29.117
29.224
29.405
31.844
53.503
76.671
77.094
77.519
89.554
97.074
128.163
128.628
131.984
138.647
193.718
210.076
0
25
50
75
100
125
150
175
200
225

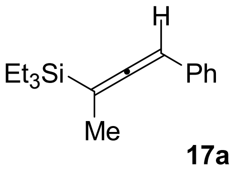

17a

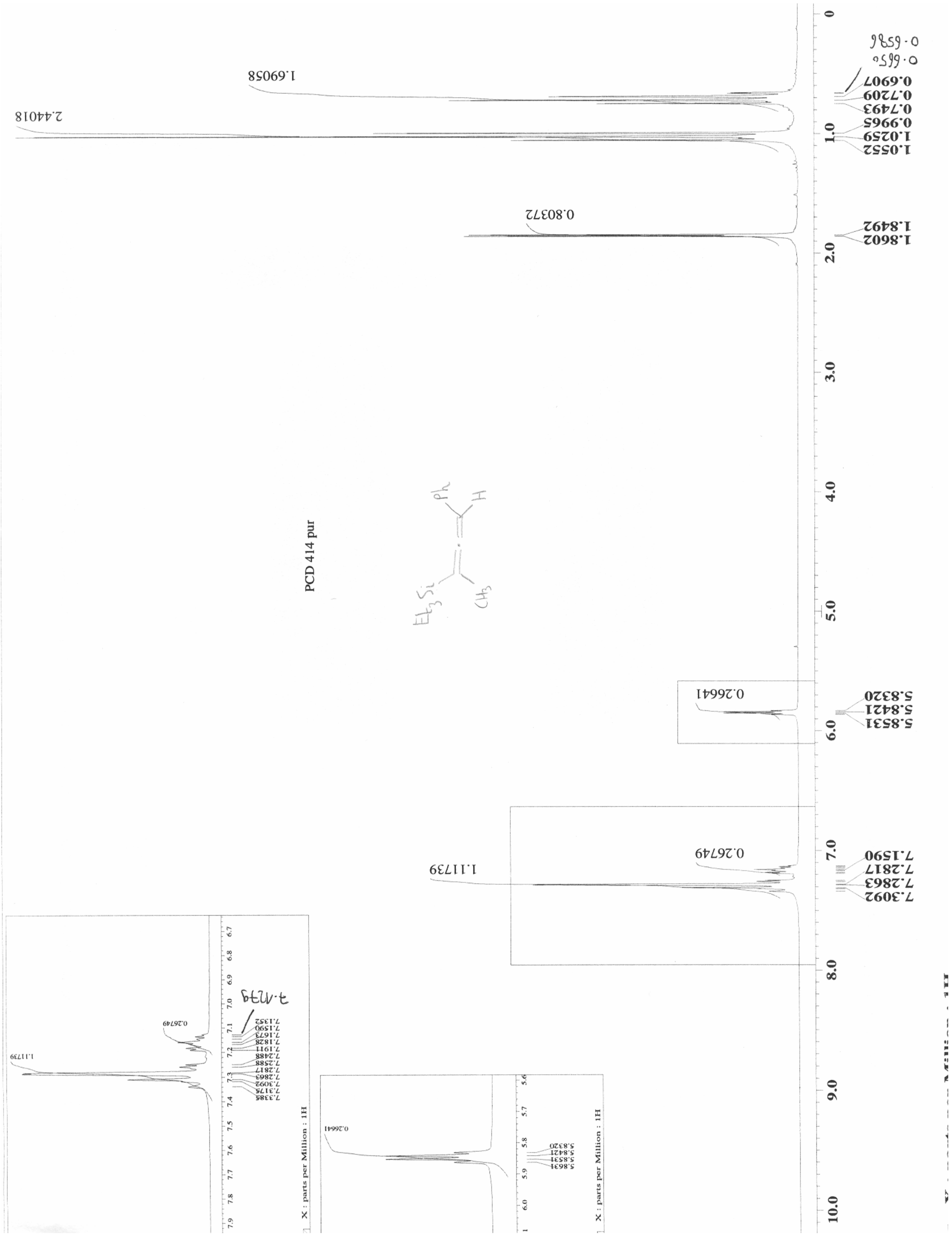

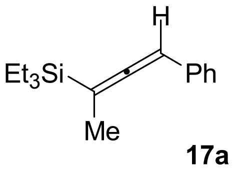
H
Et3Si
Ph
Me

17a

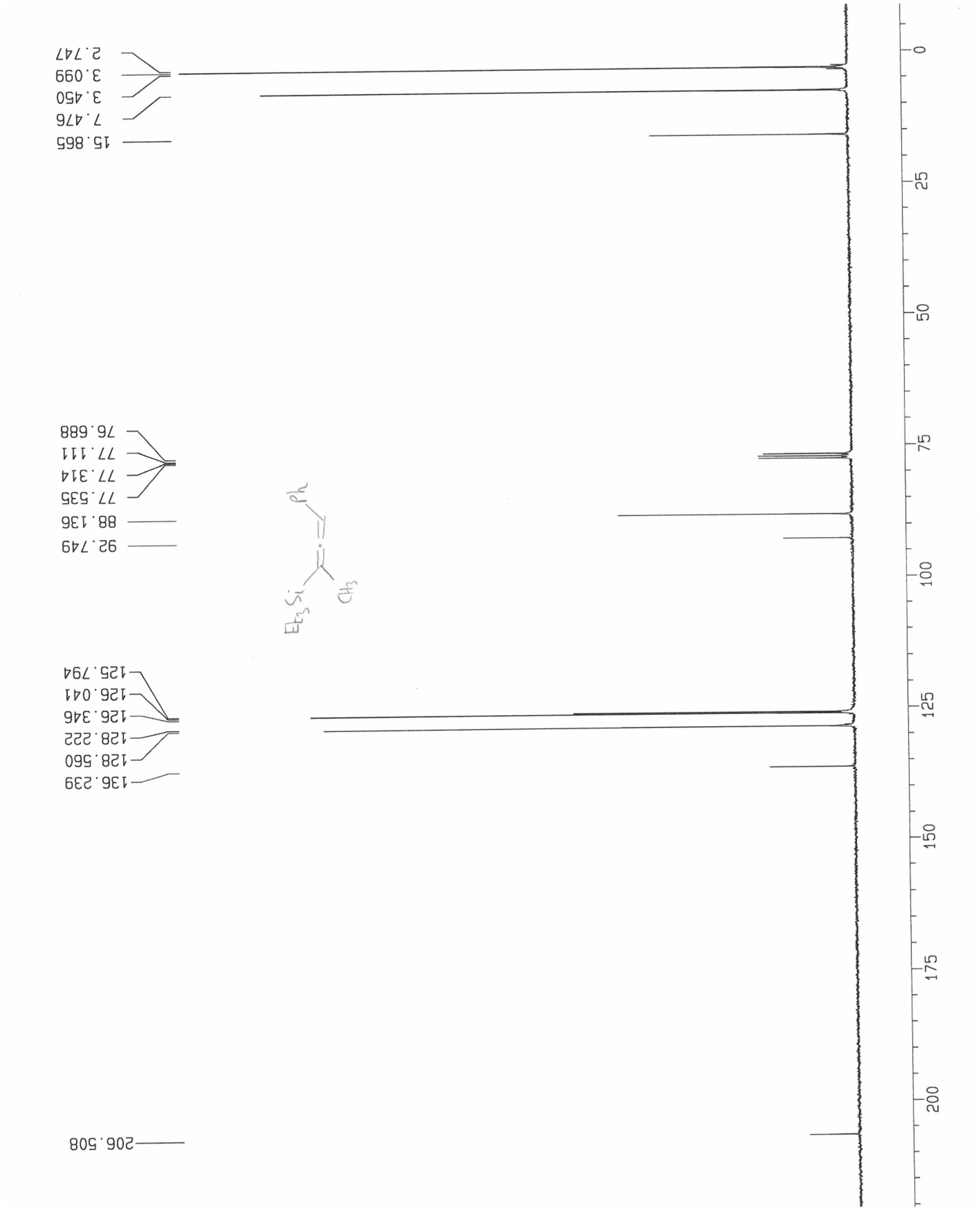
2.747
3.099
3.450
7.476
15.865
76.688
77.111
77.314
77.535
88.136
92.749
125.794
126.041
126.346
128.222
128.560
136.239
206.508
0
25
50
75
100
125
150
175
200

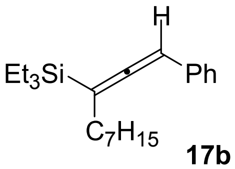

17b

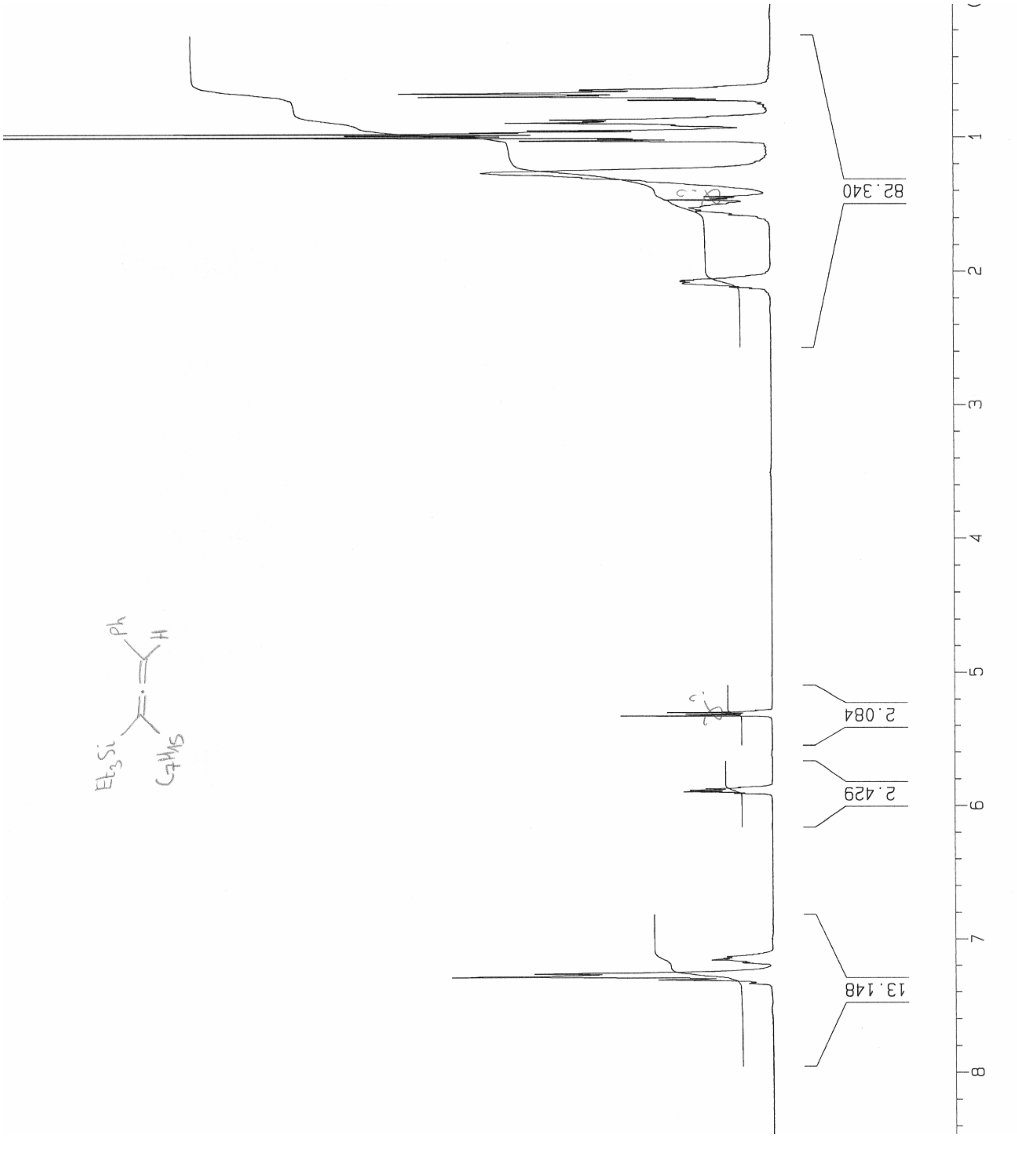

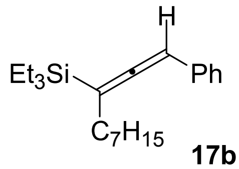

Et3Si
H
Ph
C7H15

17b

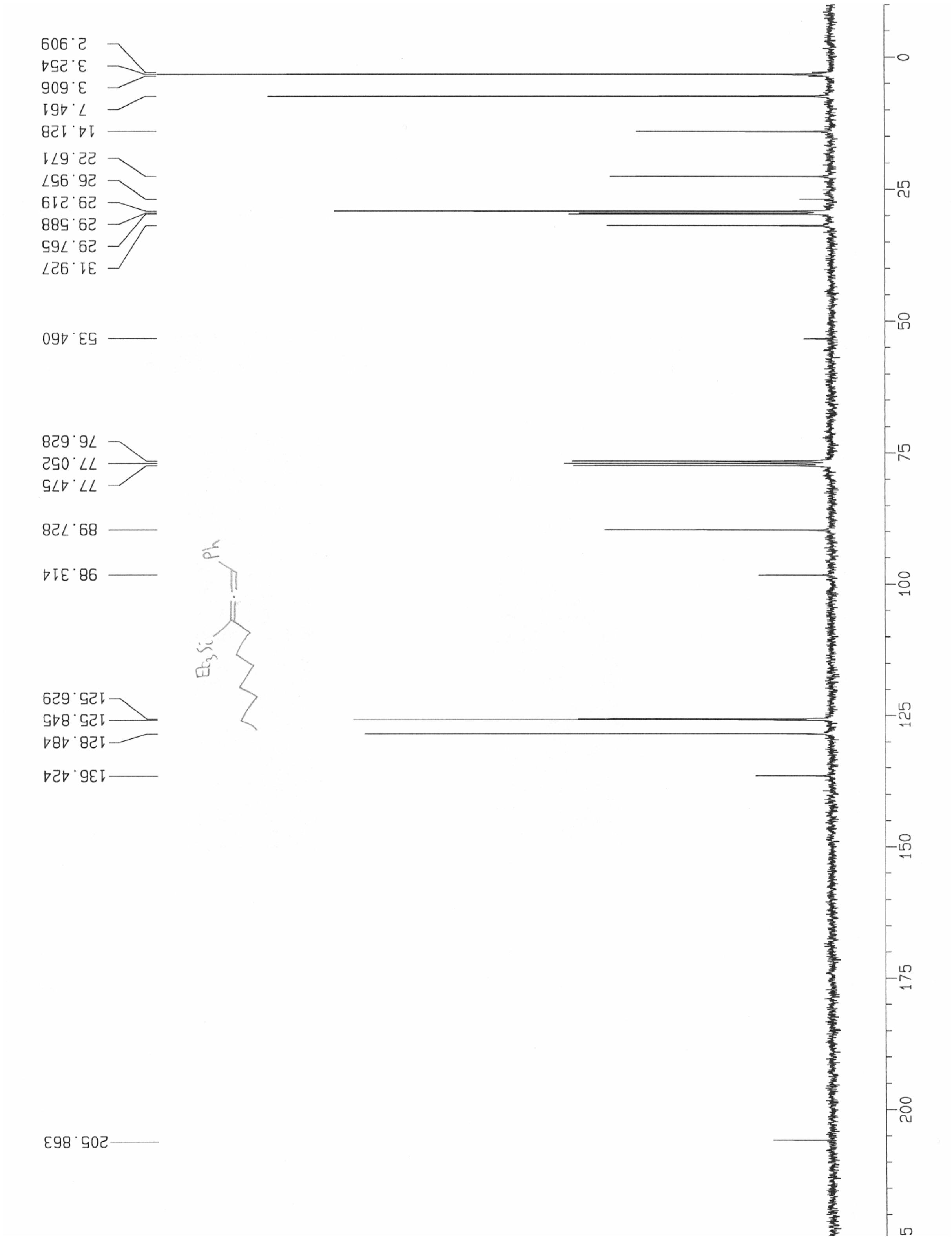

2.909
3.254
3.606
7.461
14.128
22.671
26.957
29.219
29.588
29.765
31.927
53.460
76.628
77.052
77.475
89.728
98.314
125.629
125.845
128.484
136.424
205.863
0
25
50
75
100
125
150
175
200

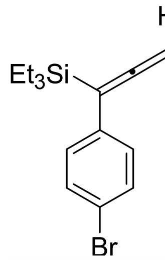

17g

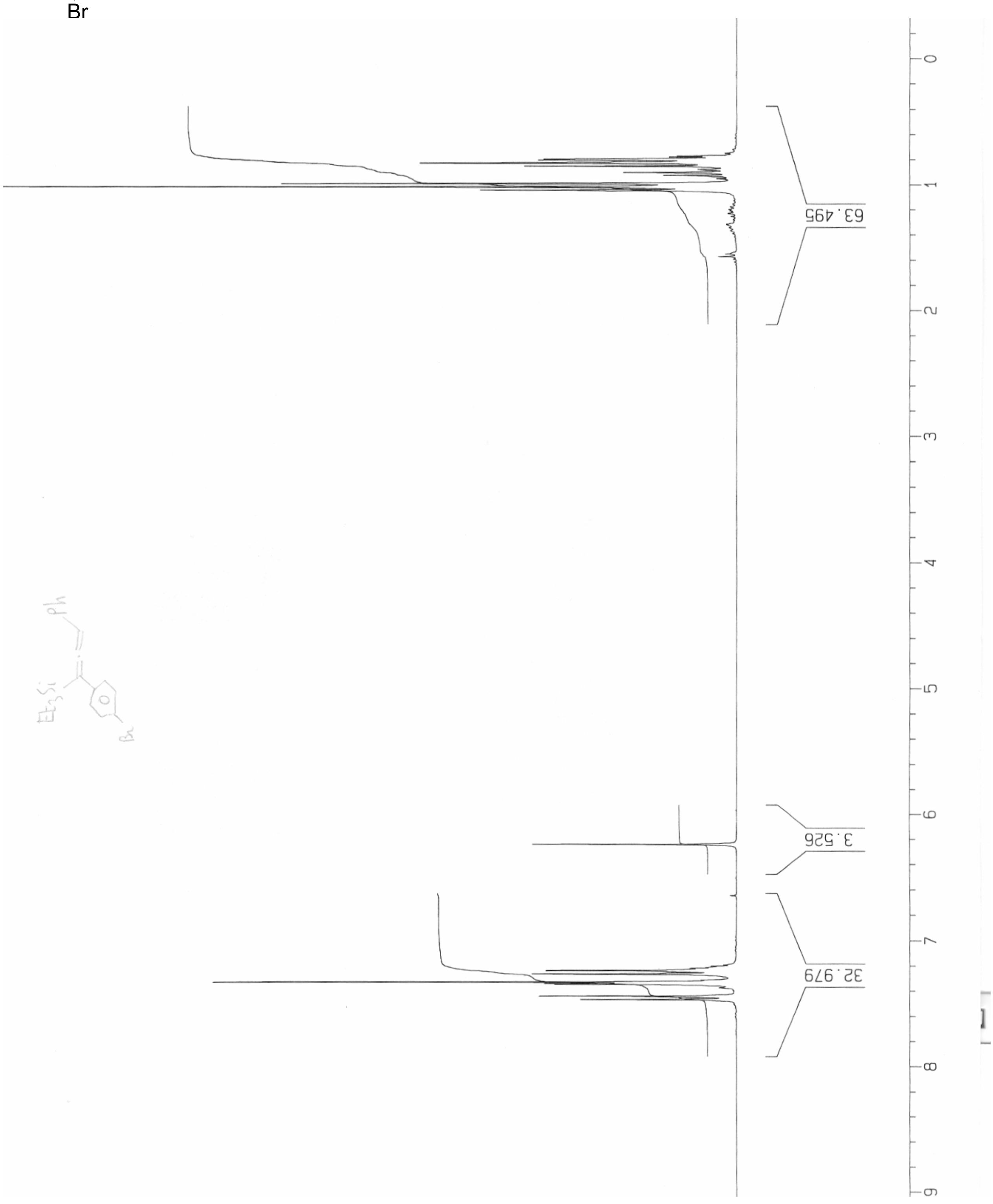

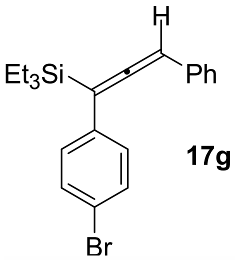
Et3Si
H
Ph
Br

17g

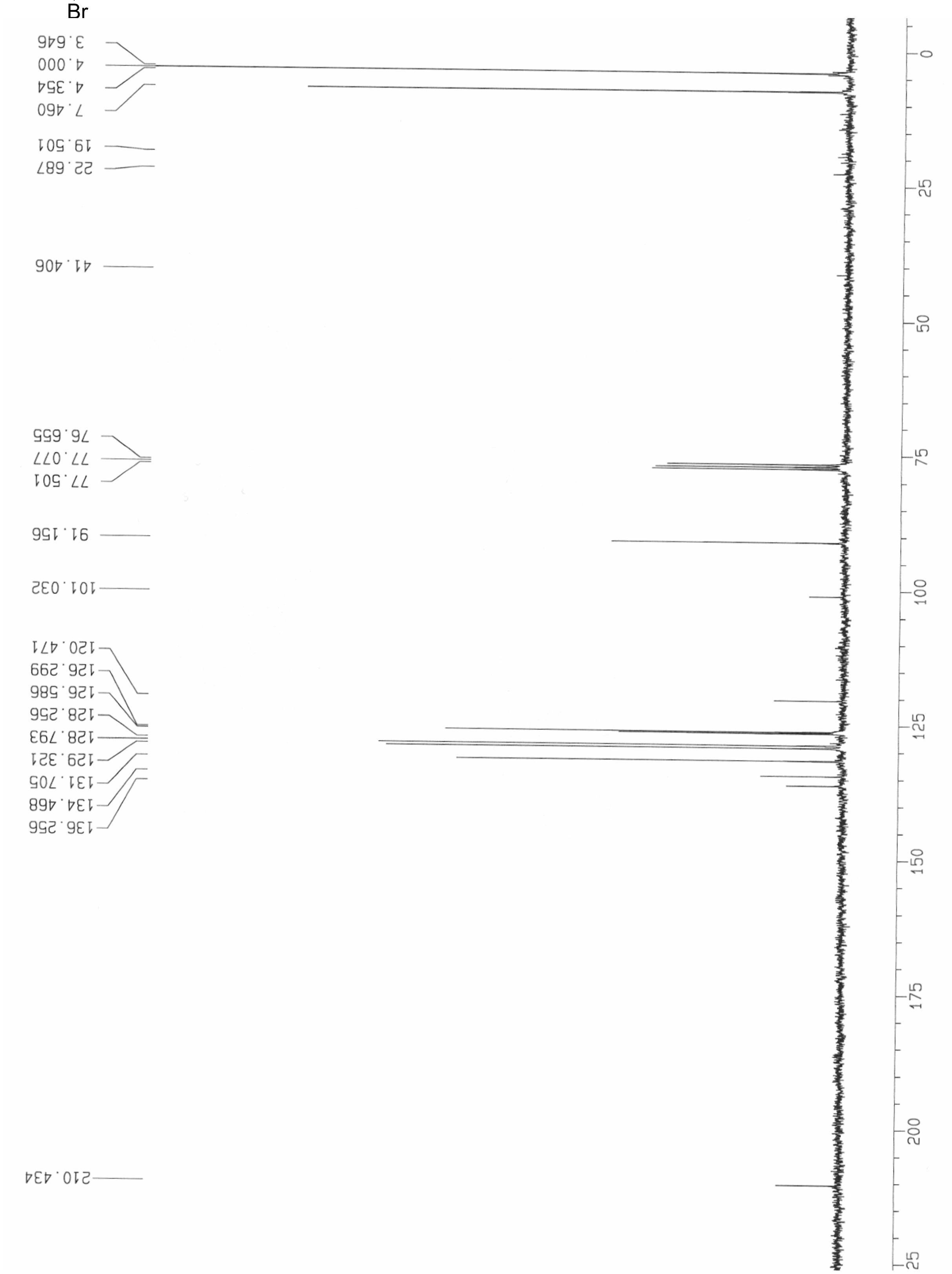
3.646
4.000
4.354
7.460
19.501
22.687
41.406
76.655
77.077
77.501
91.156
101.032
120.471
126.299
126.586
128.256
128.793
129.321
131.705
134.468
136.256
210.434
0
25
50
75
100
125
150
175
200

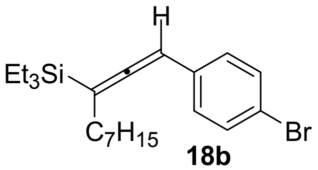

18b

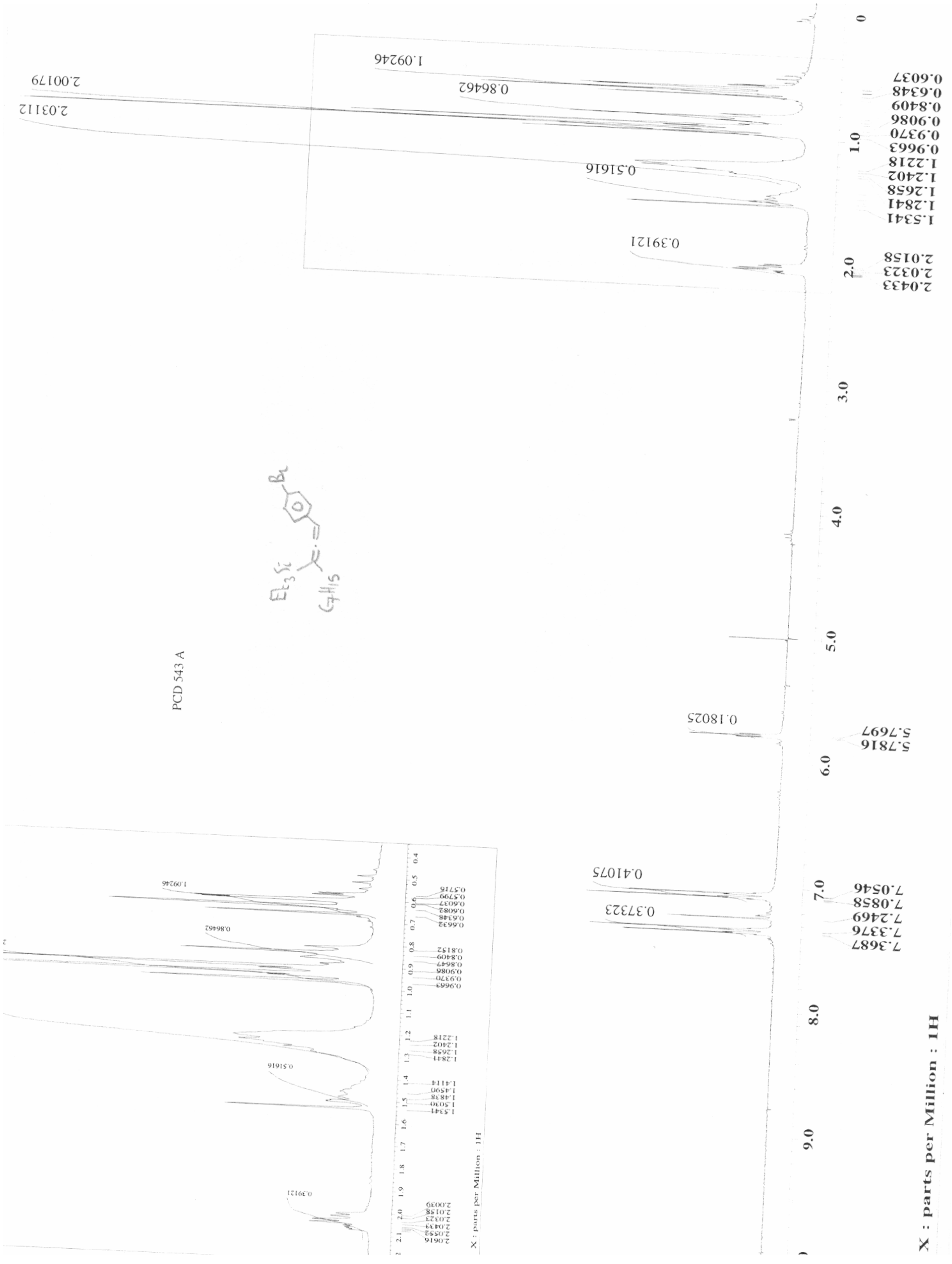

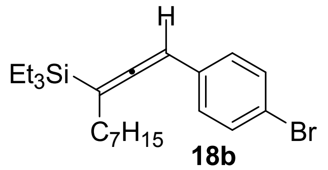
H
Et3Si
C7H15
Br

18b

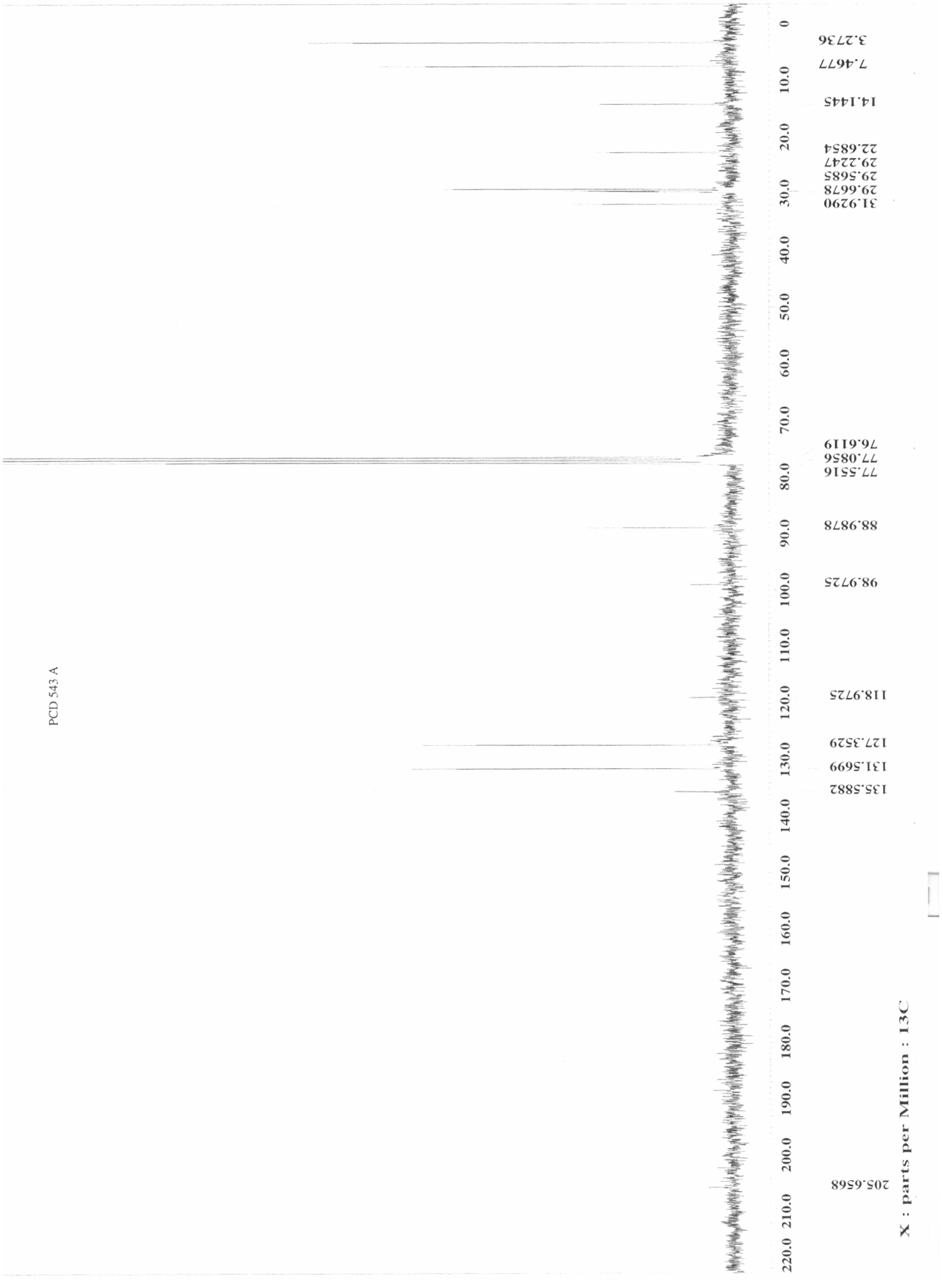
PCD 543 A
3.2736
7.4677
14.1445
22.6854
29.2247
29.5685
29.6678
31.9290
76.6119
77.0856
77.5516
88.9878
98.9725
118.9725
127.3529
131.5699
135.5882
205.6568
0
10.0
20.0
30.0
40.0
50.0
60.0
70.0
80.0
90.0
100.0
110.0
120.0
130.0
140.0
150.0
160.0
170.0
180.0
190.0
200.0
210.0
220.0
X : parts per Million : 13C

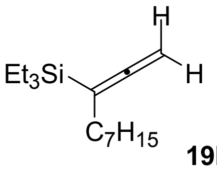

19b

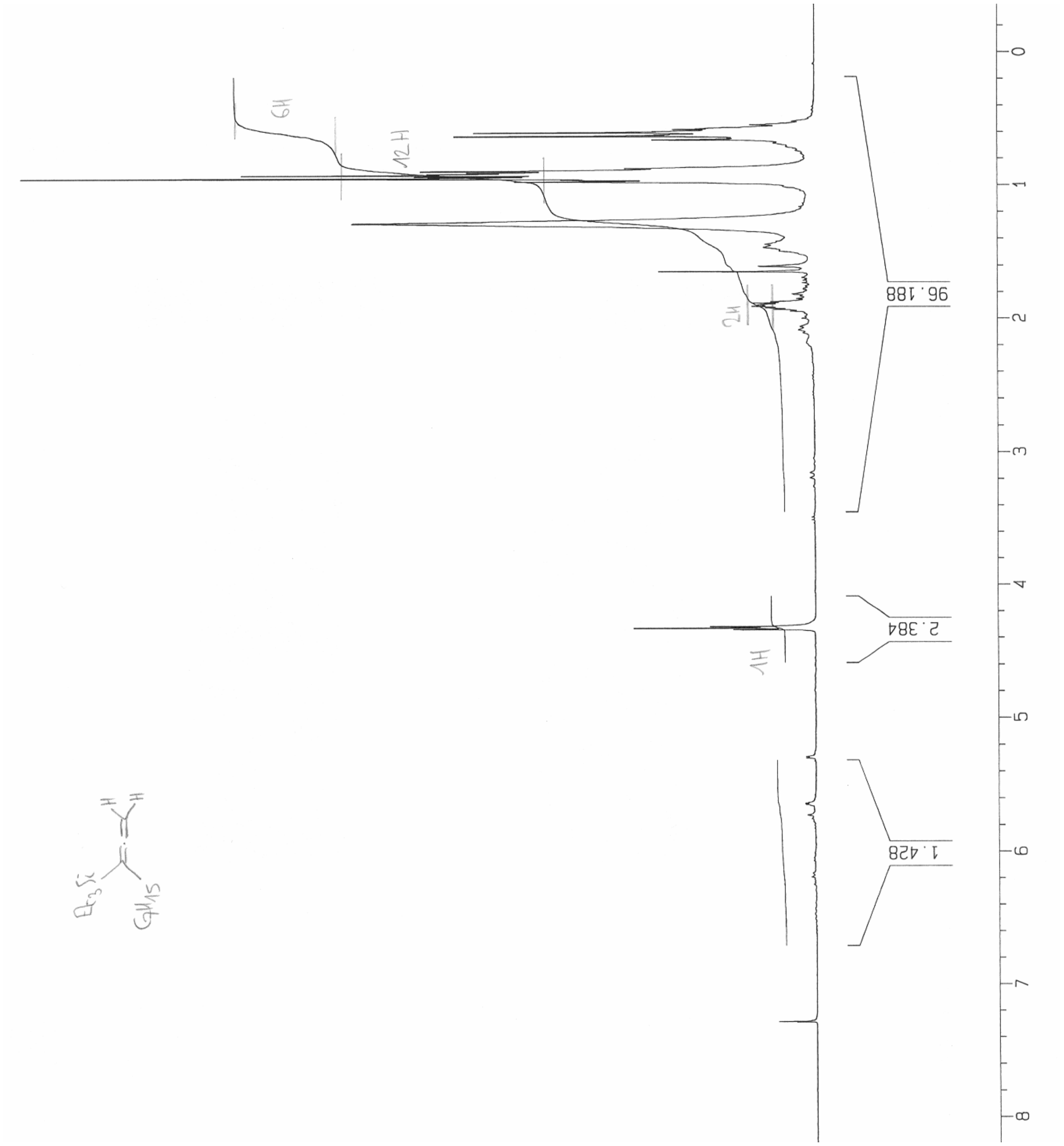

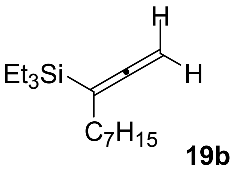
Et3Si
H
H
C7H15

19b

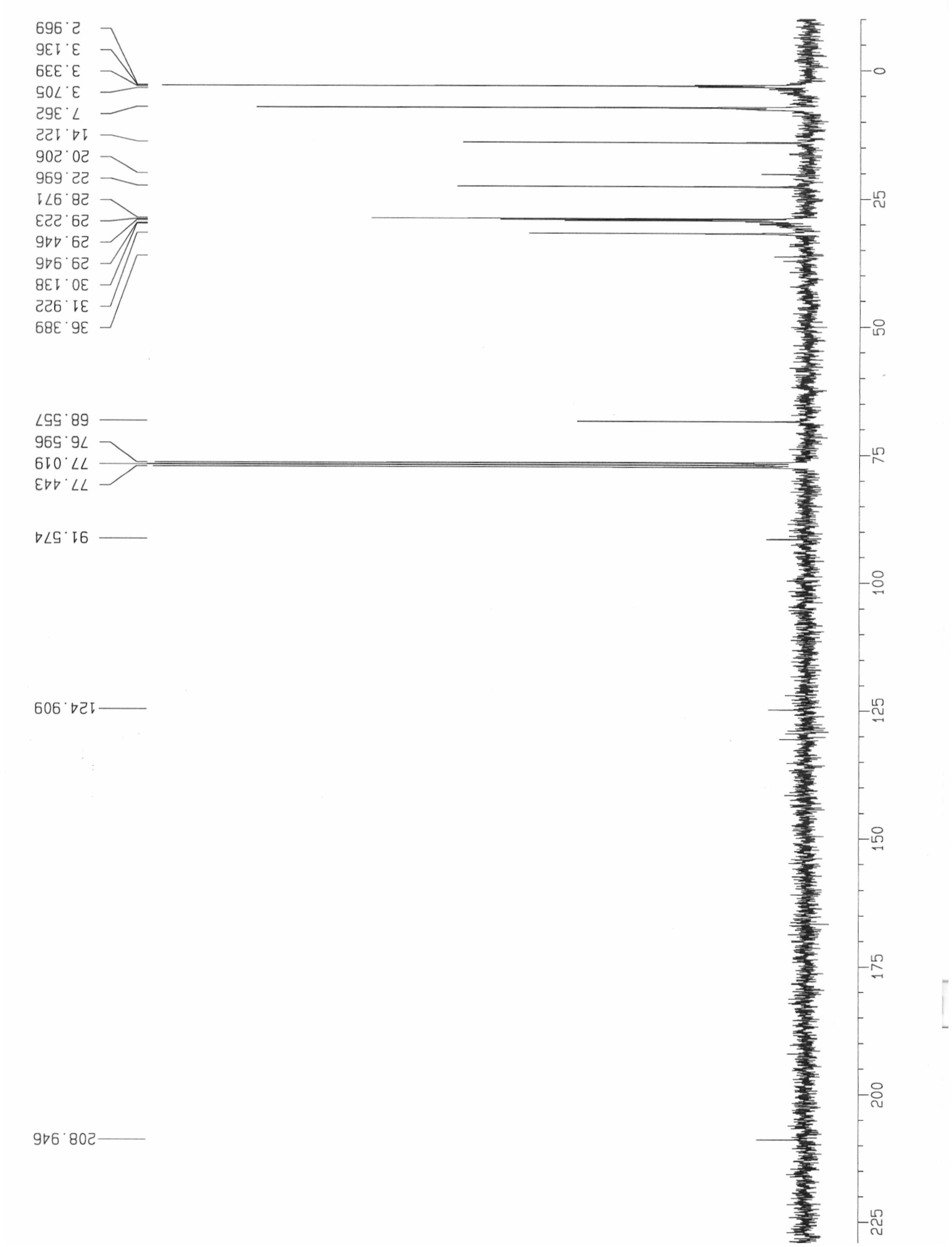
2.969
3.136
3.339
3.705
7.362
14.122
20.206
22.696
28.971
29.223
29.446
29.946
30.138
31.922
36.389
68.557
76.596
77.019
77.443
91.574
124.909
208.946
0
25
50
75
100
125
150
175
200
225

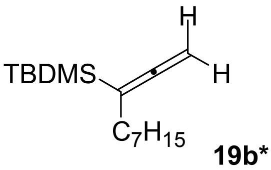

19b*

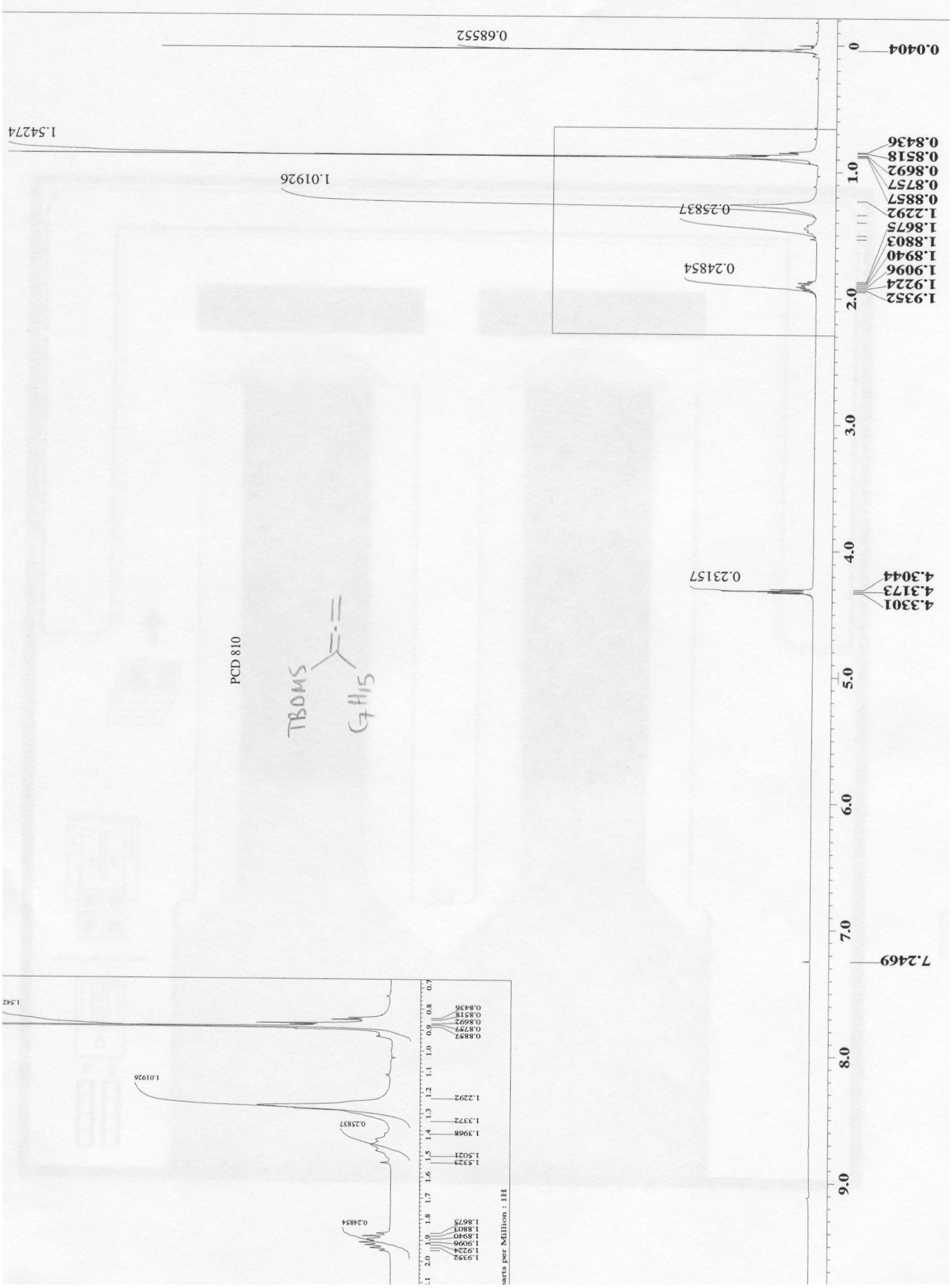

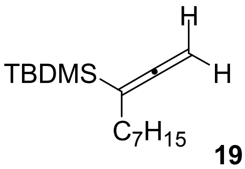
TBDMS
H
H
C_7H_{15}

19b*

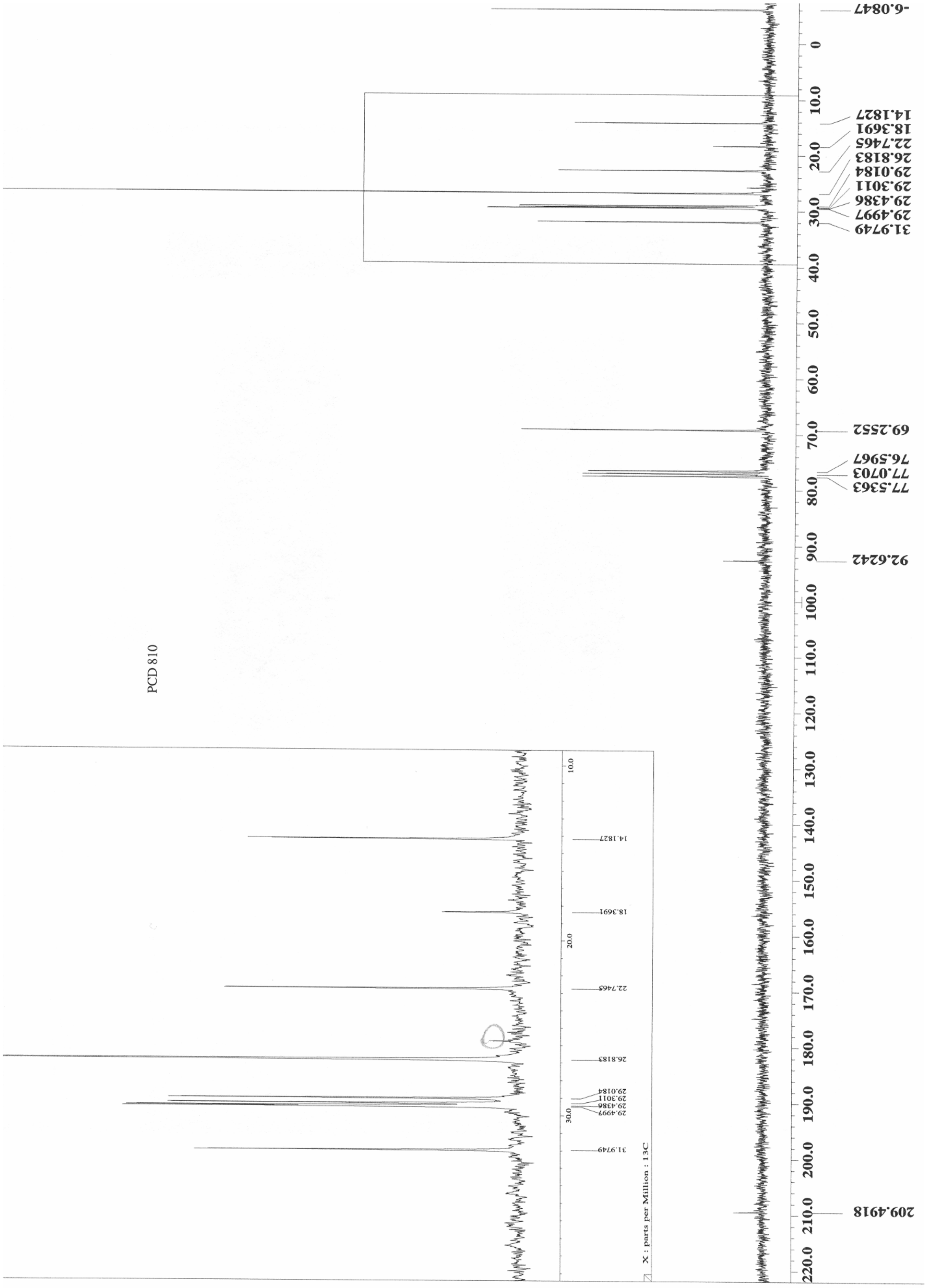
PCD 810
X : parts per Million : 13C
-6.0847
14.1827
18.3691
22.7465
26.8183
29.0184
29.3011
29.4386
29.4997
31.9749
69.2552
76.5967
77.0703
77.5363
92.6242
209.4918

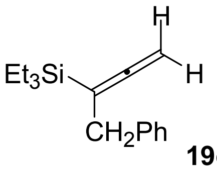

19c

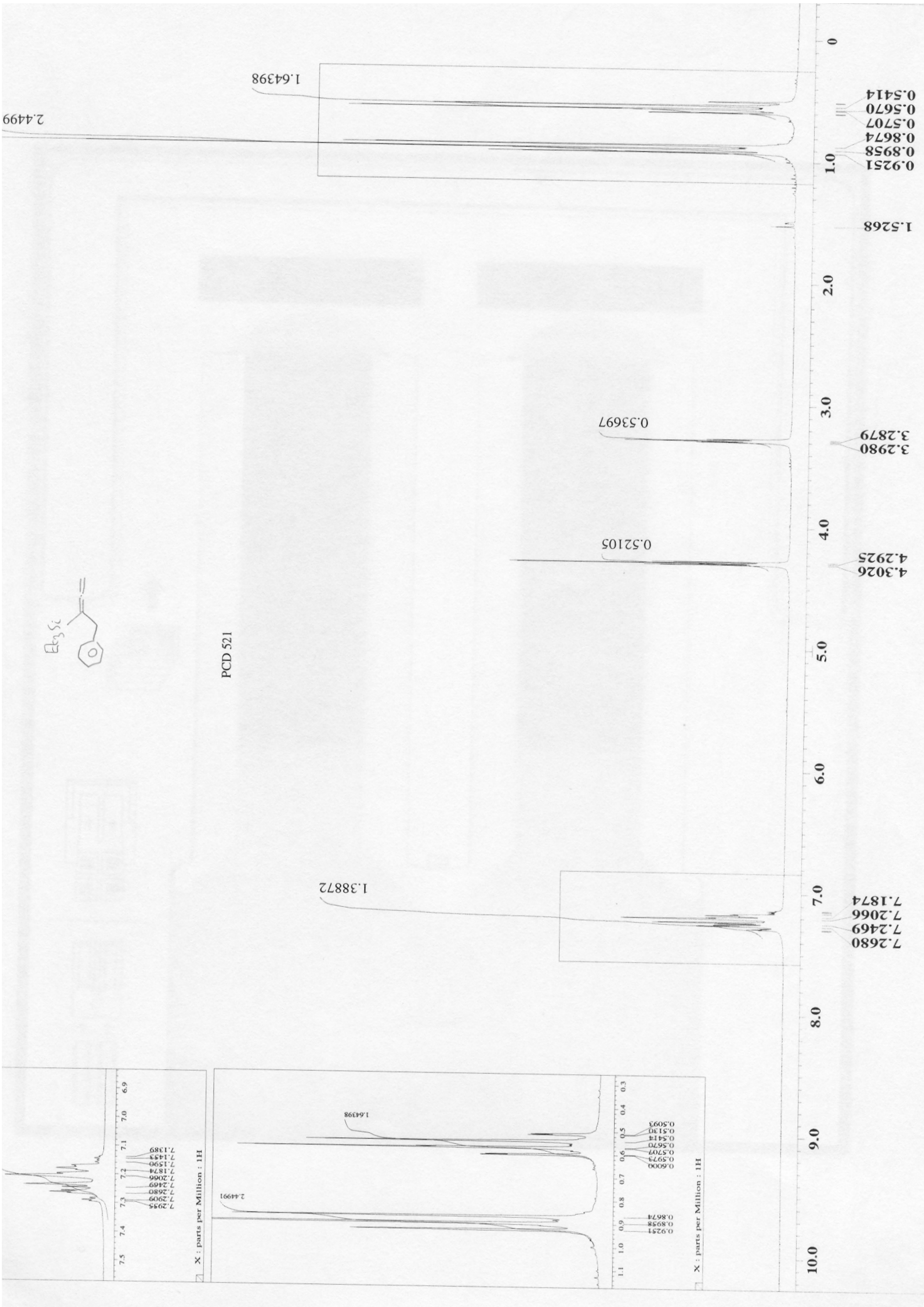

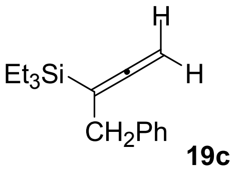
H
Et3Si
H
CH2Ph

19c

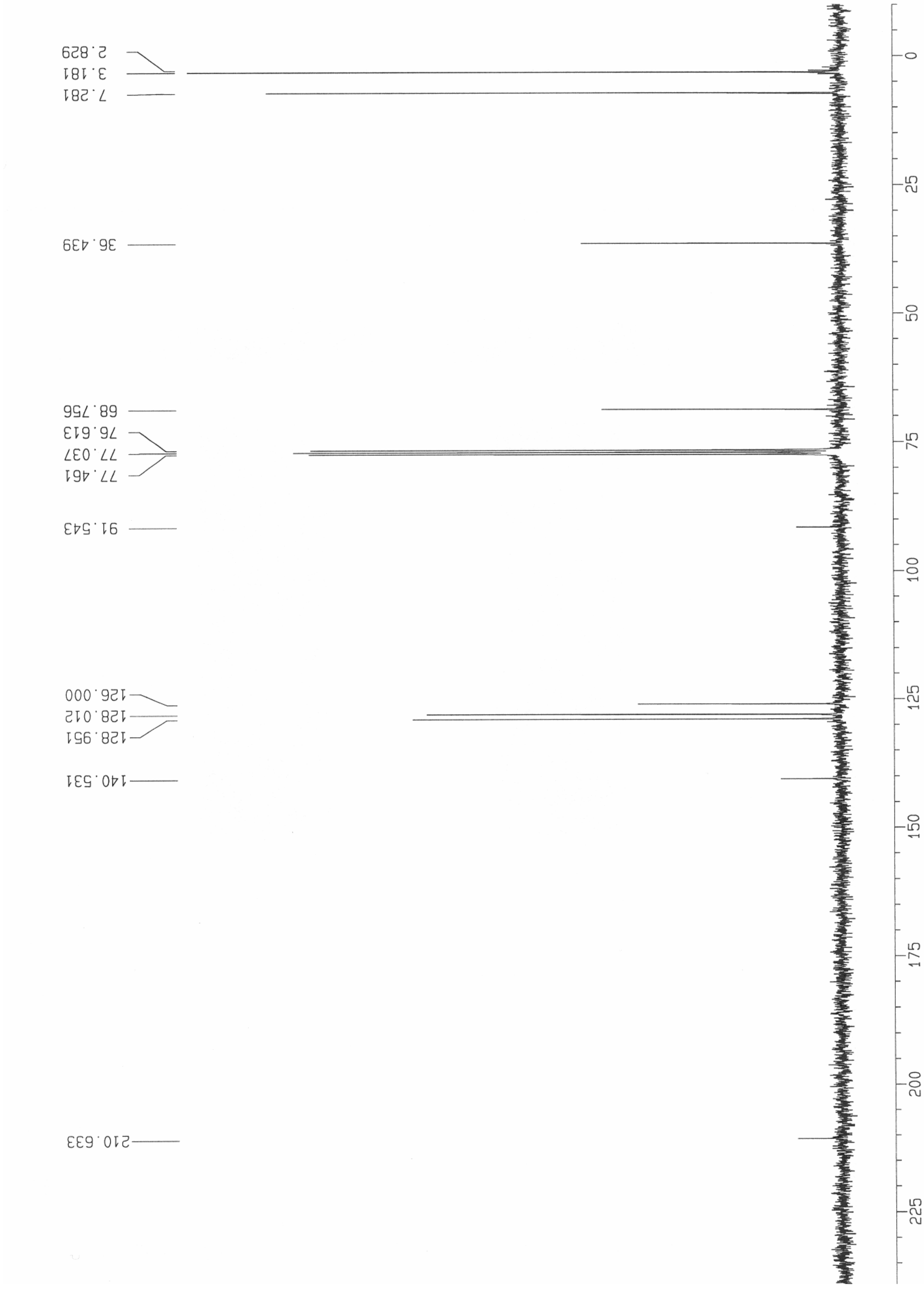
2.829
3.181
7.281
36.439
68.756
76.613
77.037
77.461
91.543
126.000
128.012
128.951
140.531
210.633
0
25
50
75
100
125
150
175
200
225

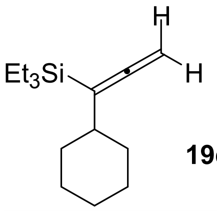

Et3Si
H
H

19d

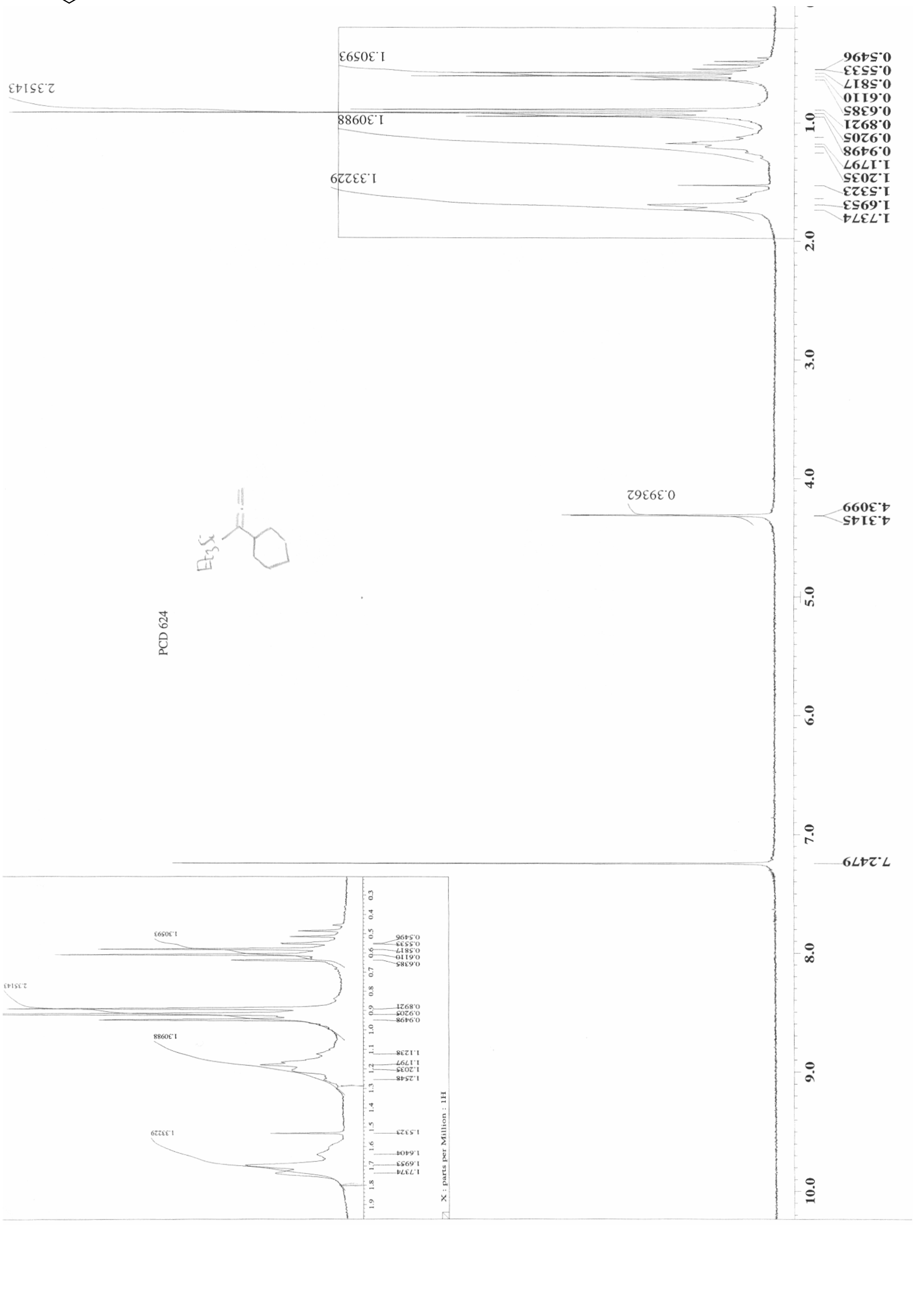

PCD 624
X : parts per Million : 1H
1.30593
1.30988
1.33229
2.35143
0.39362
0.5496
0.5533
0.5817
0.6110
0.6385
0.8921
0.9205
0.9498
1.1797
1.2035
1.5323
1.6953
1.7374
4.3099
4.3145
7.2479

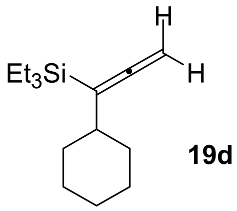
Et3Si
H
H

19d

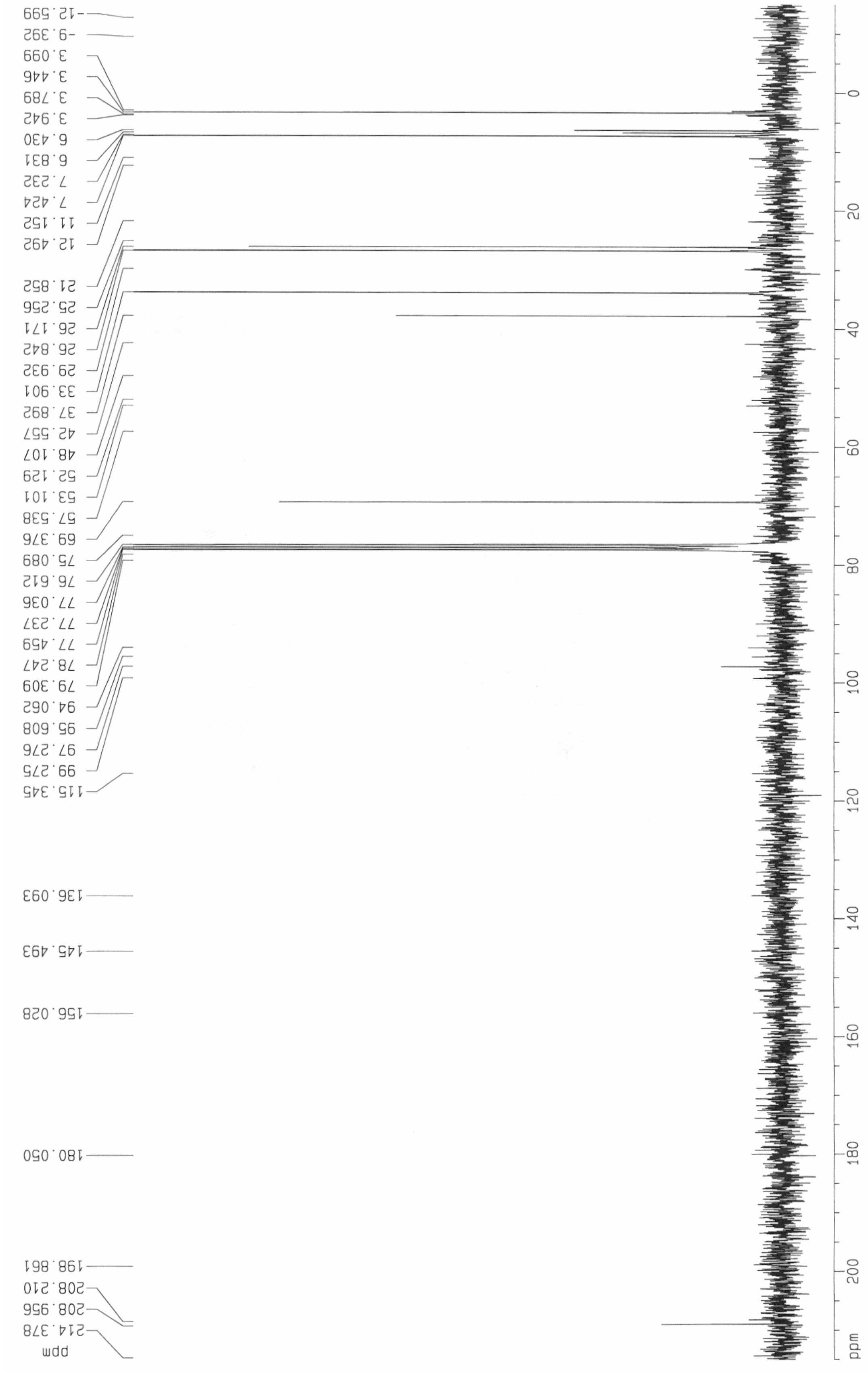
-12.599
-9.392
3.099
3.446
3.789
3.942
6.430
6.831
7.232
7.424
11.152
12.492
21.852
25.256
26.171
26.842
29.932
33.901
37.892
42.557
48.107
52.129
53.101
57.538
69.376
75.089
76.612
77.036
77.237
77.459
78.247
79.309
94.062
95.608
97.276
99.275
115.345
136.093
145.493
156.028
180.050
198.861
208.210
208.956
214.378
ppm
0
20
40
60
80
100
120
140
160
180
200
ppm

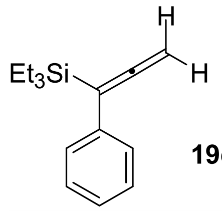

19e

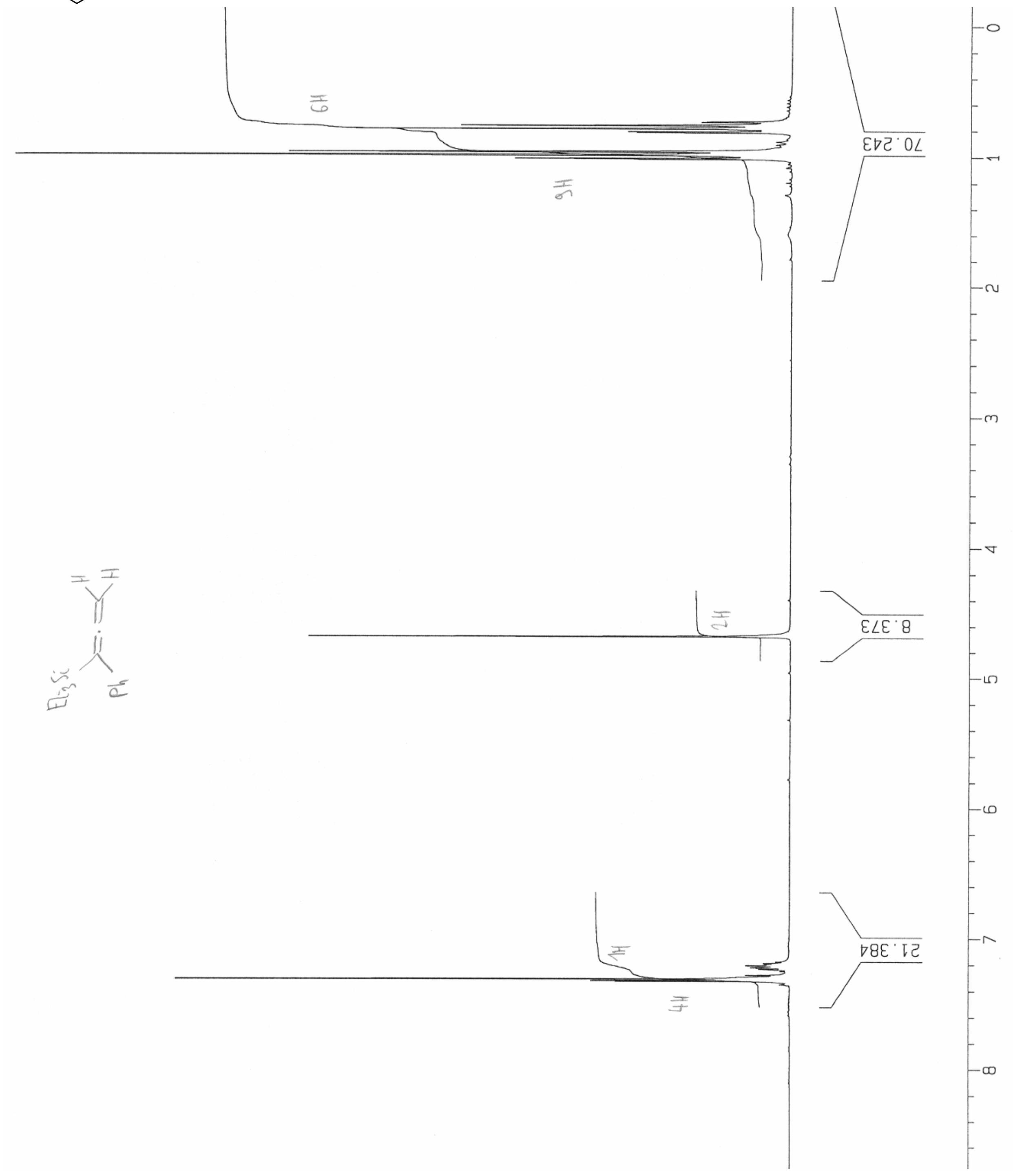

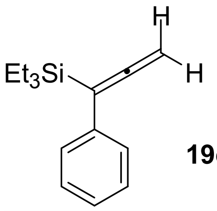

H
Et3Si
H

19e

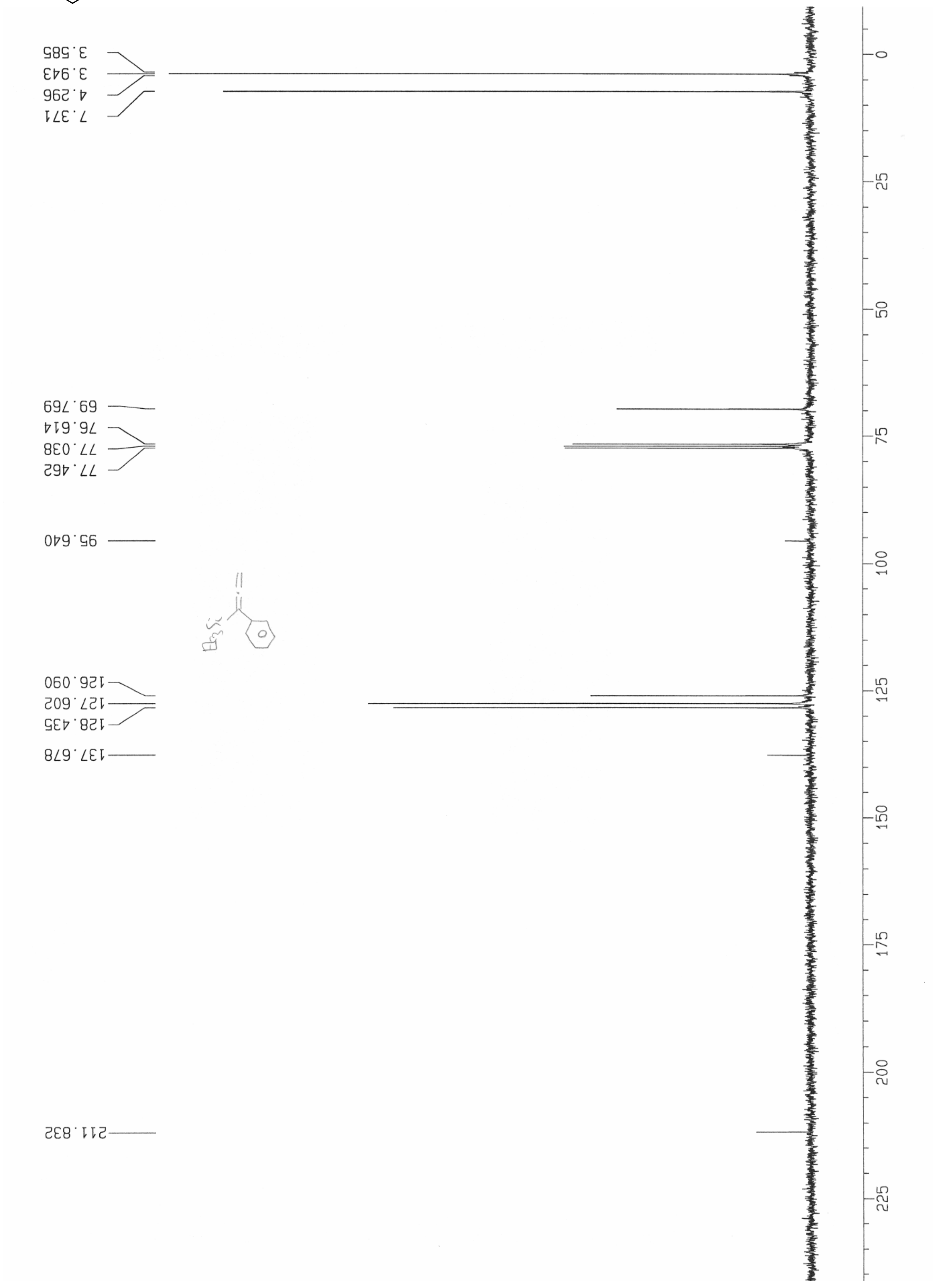

3.585
3.943
4.296
7.371
69.769
76.614
77.038
77.462
95.640
126.090
127.602
128.435
137.678
211.832
0
25
50
75
100
125
150
175
200
225
